# Oxygen–Oxygen Bond-Forming Reactions for the Synthesis of Cyclic Peroxides

**DOI:** 10.1021/jacs.6c04588

**Published:** 2026-05-08

**Authors:** Farhan A. Chowdhury, Elena J. Helm, K. A. Woerpel

**Affiliations:** Department of Chemistry, 5894 New York University, 100 Washington Square East, New York, New York 10003, United States

## Abstract

Treatment of hydroperoxides bearing pendant hydroxyl groups (OH groups) with sulfonyl chlorides under basic conditions formed cyclic peroxides. Mechanistic experiments indicated that these reactions proceeded by an oxygen–oxygen bond-forming substitution reaction, where the two oxygen atoms take opposite roles: one oxygen atom serves as an electrophile, and the other serves as a nucleophile. This mechanism was supported by ^17^O-labeling experiments and by the determination that the reaction proceeds with retention of configuration at the carbon atoms of the ring. The reaction was rapid at −78 °C, it was high-yielding, and it was general for a range of substrates, including the formation of five- and six-membered cyclic and bicyclic peroxides. This reaction, therefore, serves as a synthetic analogy to the oxygen–oxygen bond formation reaction used in nature to form dioxygen.

## Graphical abstract

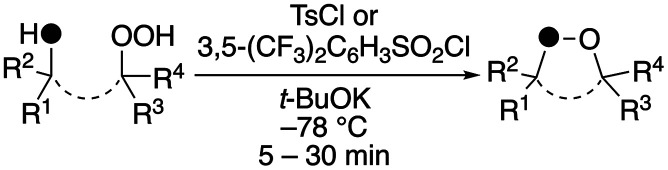

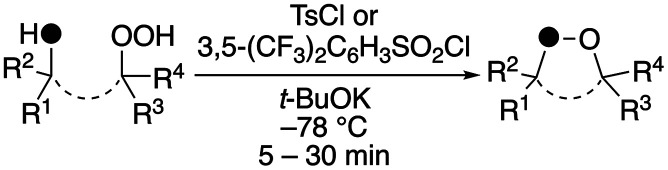

## Introduction

The formation of bonds between two oxygen atoms, a transformation performed by photosystem II, is a reaction that is considered central to life on Earth because it generates oxygen gas and enables the synthesis of ozone.^1–3^ Because of the importance of the oxygen-evolving complex in photosystem II, chemists have studied the metal complexes involved in O_2_ generation,^4^ developing model complexes containing metals such as manganese and calcium to form the oxygen–oxygen bonds in O_2_ and H_2_O_2_.^5–13^ The mechanisms of these reactions are complex, but one likely mechanism involves a nucleophilic oxygen atom attacking an electrophilic oxygen atom (outlined in Scheme 1A^14^).

**Scheme 1:**
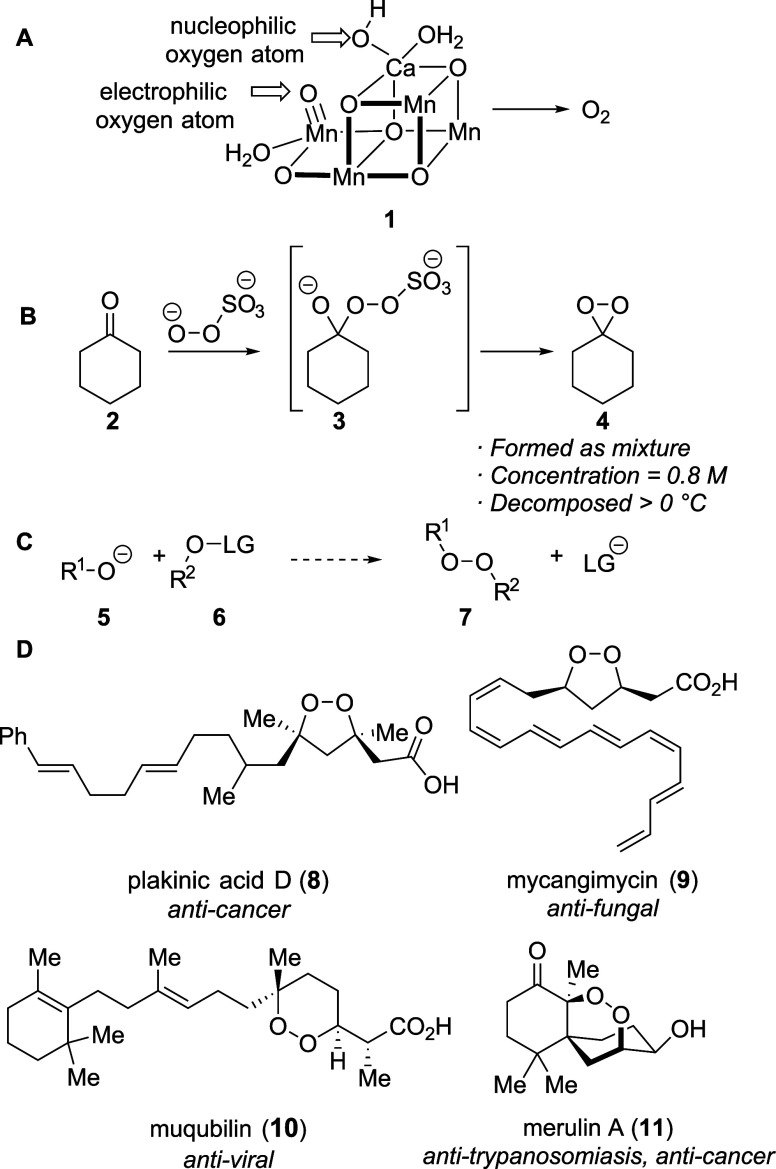
Oxygen–Oxygen Bond Formation and Products That Could Be Prepared Using That Reaction

The analogous formation of oxygen–oxygen bonds to prepare isolable organic peroxides, however, is much less developed. The connection of a nucleophilic oxygen atom with an electrophilic oxygen atom has been reported for the synthesis of dioxiranes, but these cyclic peroxides have short lifetimes (Scheme 1B).^15,16^ Coupling of alkoxy radicals was used to synthesize acyl peroxides,^17^ and this reaction may be involved in the electrocatalytic formation of cyclic peroxyimidates.^18^ Small amounts of cyclic peroxides (2%) can be formed in the reaction of F_2_ with a protected diol, but this reaction forms mixtures of products, not pure cyclic peroxides.^19^ Related reactions of HOF with H_2_O formed H_2_O_2_, but the product of the reaction reacted further, so steady-state concentrations of H_2_O_2_ are low (∼0.2 M).^20^

The limited success in achieving oxygen–oxygen bond formation, which requires the formation of a relatively weak bond,^21^ has caused chemists to synthesize peroxides using reagents where the oxygen–oxygen bond of the product is already present. This limitation has meant that synthetic methods rely upon the use of O_2_ (either in its triplet or singlet state), H_2_O_2_, or O_3_.^22^ Regardless of whether the peroxide is the target of a synthetic scheme^23–28^ or is a synthetic intermediate,^29–33^ O_2_, with the oxygen–oxygen bond intact, is the most common source of the peroxide functional group. Even in the biosynthesis of peroxide-containing natural products, enzymes do not form an oxygen–oxygen bond, instead incorporating the intact peroxide linkage as O_2_.^34,35^ As a result, the development of a practical, high-yielding oxygen–oxygen bond-forming reaction (Scheme 1C) would introduce a new fundamental reaction to synthetic chemistry. It would also provide a unique approach to the synthesis of cyclic peroxides, a family of compounds that exhibit potent and selective biological activity for the treatment of a number of diseases, as illustrated by the examples in Scheme 1D.^36–47^

In this paper, we report a method for the synthesis of oxygen–oxygen bonds for the synthesis of cyclic peroxides according to Scheme 1C. This reaction involves two oxygen atoms serving different roles, similar to what could be occurring in photosystem II (Scheme 1A): one oxygen atom is activated by installing a leaving group on it (as in **6**), and the resulting electrophilic oxygen atom is attacked by a second oxygen atom acting as a nucleophile (as in **5**). This substitution reaction, which we used for the synthesis of cyclic peroxides, is rapid (within minutes at −78 °C in most cases), operationally simple, and generally high-yielding. Experiments using ^17^O-labeled substrates verify that the reaction involves an oxygen–oxygen bond-forming step. Because of this mechanism, the stereochemistry of the starting hydroperoxide was retained in the product. This new oxygen–oxygen bond-forming reaction is general for the synthesis of five- and six-membered-ring peroxides with various substitution patterns.

## Results and Discussion

The formation of oxygen–oxygen bonds was established by developing methods to activate the OOH group of a hydroperoxide to make the internal oxygen atom electrophilic. The substrate **13** was readily prepared in one step by hydroperoxidation^48^ of the corresponding homoallylic alcohol **12** (eq 1), which was prepared in one step^49^ from the corresponding aryl methyl ketone. The resulting cyclization reaction was anticipated to form the five-membered cyclic peroxide **14** (eq 2) by formation of the oxygen–oxygen bond, similar to substitution reactions that form oxygen–carbon^50^ and oxygen–nitrogen bonds^51^ in cyclic products from peroxide-containing electrophiles.
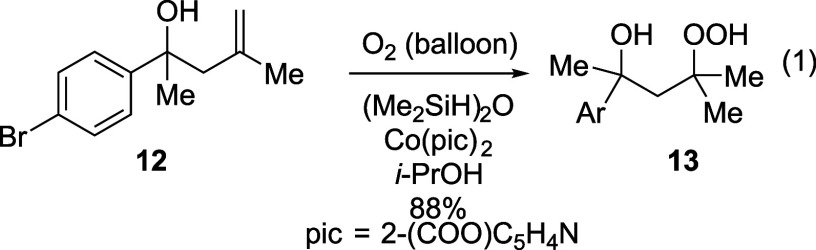
 
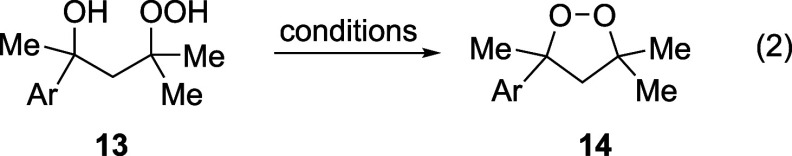


Despite the limited precedent for nucleophilic substitutions on an oxygen electrophile by an oxygen nucleophile, a number of conditions could be used to form cyclic peroxide **14** (eq 2). Table 1 shows the conditions that are effective for this transformation. Activation by sulfonyl chlorides was particularly efficient. Treatment of hydroperoxide **13** with 1.1 equiv of *p-*toluenesulfonyl chloride and 2.5 equiv of an alkoxide base in THF at −78 °C formed the product within 30 min, whereas other bases formed the product but in lower yields (entries 3–5 vs entries 1 and 2). The ^1^H and ^13^C­{^1^H} spectra of the unpurified reaction mixtures showed that the product was formed cleanly under the conditions of entries 4 and 5, although the product was purified by chromatography to obtain an analytically pure material (85% isolated yield). The reaction was not highly sensitive to the temperature of the reaction mixture (entry 6). Two equivalents of base were needed, which is consistent with the fact that two acidic protons would need to be removed in the reaction (entries 5 and 8 vs entry 7). Cyclization was also rapid, occurring in just minutes at −78 °C (entries 9 and 10). The use of ethereal solvents gave better yields than the use of toluene (entries 5 and 11 vs entry 12), which may reflect the solubility of the base at −78 °C. Other sulfonyl chlorides also promoted the cyclization (entries 13 and 14), and even Ac_2_O and benzoyl chloride were effective (entries 15 and 16). Overall, the reaction was robust, allowing products to be formed in high yields even with considerable variation of reaction conditions.^52–54^

**Table 1:** Optimization of the Reaction Conditions for Cyclization^a^

Entry	Activating Agent	Base	Equiv of Base	Temp	Time (min)	Solvent	% Yield (NMR)
1	TsCl	Et_3_N	2.5	–78 °C	30	THF	10
2	TsCl	NaH	2.5	–78 °C	30	THF	14
3	TsCl	*t*-BuONa	2.5	–78 °C	30	THF	65
4	TsCl	*t*-BuOLi	2.5	–78 °C	30	THF	89
5	TsCl	*t*-BuOK	2.5	–78 °C	30	THF	90 (85%)
6	TsCl	*t*-BuOK	2.5	22 °C	30	THF	73
7	TsCl	*t*-BuOK	1.0	–78 °C	30	THF	35
8	TsCl	*t*-BuOK	3.0	–78 °C	30	THF	91
9	TsCl	*t*-BuOK	2.5	–78 °C	5	THF	73
10	TsCl	*t*-BuOK	2.5	–78 °C	10	THF	83
11	TsCl	*t*-BuOK	2.5	–78 °C	30	Et_2_O	86
12	TsCl	*t*-BuOK	2.5	–78 °C	30	PhMe	36
13	3,5-(CF_3_)_2_C_6_H_3_SO_2_Cl	*t*-BuOK	2.5	–78 °C	30	THF	83
14	4-(MeO)C_6_H_4_SO_2_Cl	*t*-BuOK	2.5	–78 °C	30	THF	93
15	Ac_2_O	*t*-BuOK	2.5	22 °C	30	THF	59
16	PhCOCl	*t*-BuOK	2.5	22 °C	30	THF	88

a Conditions that give the cyclic peroxide **14** from precursor **13** (eq 2). Isolated yield is given in parentheses.

Although the reaction was initially envisioned to involve oxygen–oxygen bond formation, the observation of the cyclic peroxide was not itself proof that such a reaction had occurred. The fact that no preparative synthesis of a cyclic dialkyl peroxide (or even an acyclic peroxide) by oxygen–oxygen bond formation had been developed previously made it imperative to devise experiments to test the plausibility of this mechanism and alternative mechanisms. In addition to the oxygen–oxygen bond-forming reaction that had been envisioned (Scheme 2A), three other mechanisms were considered. Instead of the formation of the tosylated peroxide required for the oxygen–oxygen bond-forming process (Scheme 2A), tosylation could have occurred at the hydroxyl group to convert its pendant carbon atom into an electrophile, and cyclization would occur using the hydroperoxy group as the nucleophile (Scheme 2B). Considering that the tertiary sulfonate ester would reside at a benzylic position, this cyclization could occur by a dissociative mechanism involving a carbocationic intermediate (Scheme 2C). Alternatively, the reaction could proceed via alkoxyl radical intermediates (Scheme 2D), similar to the mechanism proposed for the formation of difluorodioxirane gas from gaseous FCO_2_F and ClF passed over solid CsF in a Teflon tube.^55^

**Scheme 2:**
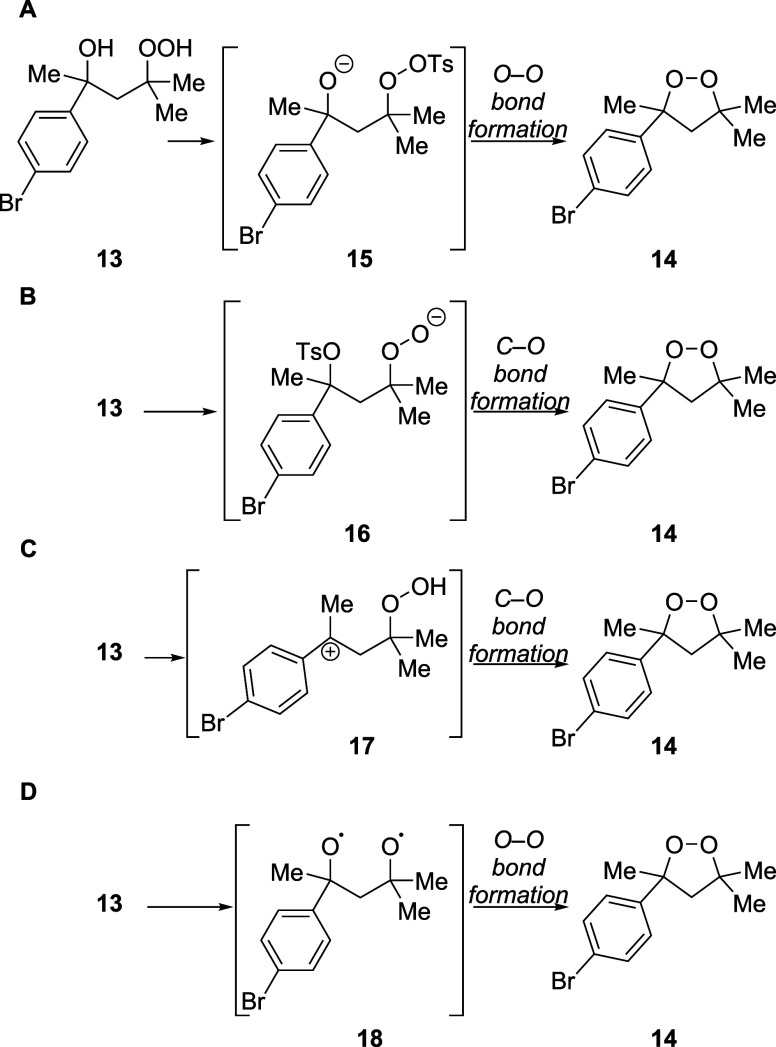
Possible Mechanisms for the Formation of the Cyclic Peroxide

Initial experiments argued against the two mechanisms that required tosylation of the hydroxyl group instead of the hydroperoxyl group (Schemes 2B and 2C). Under conditions similar to the reaction conditions (*t*-BuOK, TsCl, −78 °C), and even at elevated temperatures for longer periods of time, no tosylation of the structurally related tertiary homoallylic alcohol **12** occurred (Scheme 3A). Consequently, tosylation of the hydroxyl group would not be fast enough to enable the formation of the cyclic peroxide to be complete in 30 min at −78 °C (Table 1, entry 5).

**Scheme 3:**
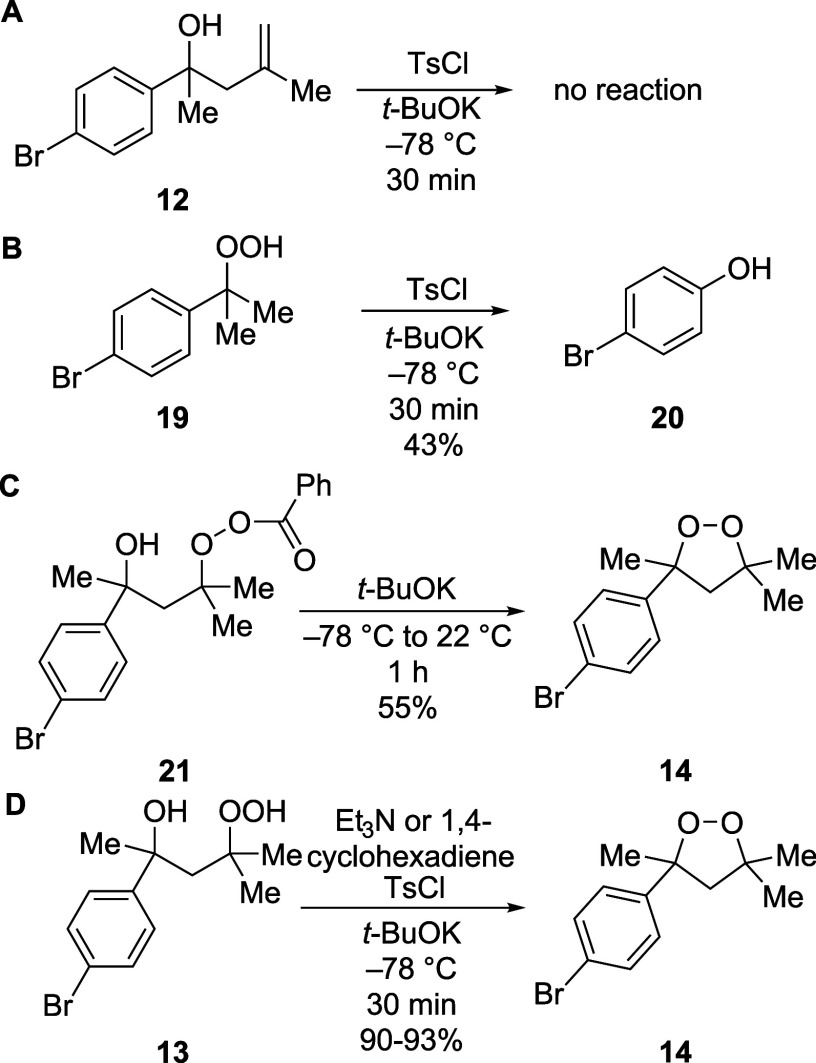
Mechanistic Experiments Suggesting that the Cyclic Peroxide was Formed by Making the Oxygen–Oxygen Bond, Not an Alternative Mechanism

By contrast, sulfonylation of an OOH group was rapid, and it likely reacts to form a product by reaction with an alkoxide ion (**15**, Scheme 2A). Treatment of hydroperoxide **19** with *t*-BuOK and TsCl at −78 °C led to the rapid formation of the corresponding phenol (**20**, Scheme 3B), indicating that an OOH group can be activated by sulfonylation and can undergo further reactions^56^ at low temperatures. The rapid reaction of this peroxysulfonate intermediate is consistent with the fact that peroxysulfonate esters are unstable at room temperature, exploding within minutes.^57^ All efforts to prepare and isolate sulfonylated derivatives of hydroperoxide **13** led to the formation of either cyclic peroxide **14** or decomposition products. Reactions with the corresponding acylated hydroperoxide, however, provide additional evidence that activation of the hydroperoxyl group was required for cyclization. The analogous peroxybenzoate ester **21**, which is likely to be the intermediate in the reaction in Table 1, entry 16 (Scheme 3C), could be isolated. Treatment of this peroxybenzoate with base formed cyclic peroxide **14** upon warming to room temperature. The slower cyclization is consistent with the fact that a benzoate ion is a poorer leaving group than a tosylate ion.^58^

Additional experiments show that a mechanism involving radical intermediates such as **18** ^59^ is unlikely to be involved in these oxygen–oxygen bond-forming reactions. The presence of triethylamine or 1,4-cyclohexadiene, which are known to trap alkoxyl radicals rapidly,^60–63^ did not affect the yield or the rate of the reaction (Scheme 3D). The absence of radical intermediates is consistent with earlier studies suggesting that peroxytosylates decompose at low temperatures (5 °C) by ionic pathways, not radical pathways.^57^ In addition, no fragmentation products were identified, which would have been characteristic of reactions involving alkoxyl radicals.^60,64–66^

Studies of the stereochemical course of the cyclization provided additional evidence that tosylates such as **16** (Scheme 2B) could not be reactive intermediates. These experiments involved chemical correlations of the products to establish the stereochemical course of the oxygen–oxygen bond-forming reaction (Scheme 4). The key starting material, silyl peroxide *trans*-**22**, was formed as a mixture of diastereomers upon peroxidation of an alkene using a Co­(II) catalyst,^67^ but the *trans* isomer shown could be isolated with only small amounts of *cis*-**22** (dr = 95:5). After the hydroperoxyl group was deprotected, *trans*-**23** was subjected to the reaction conditions, which formed the cyclic peroxide *trans*-**24**. The relative stereochemistry of this compound was established by NOE experiments. Reduction of the oxygen–oxygen bond of cyclic peroxide *trans*-**24** by palladium-catalyzed hydrogenation^68^ formed diol *trans*-**25**, whose relative stereochemistry would be unchanged from that of the 1,2-dioxolane. That same diol, *trans*-**25**, was formed by hydrogenation of silyl peroxide *trans*-**22**, demonstrating that all of these compounds had the same relative stereochemistry. To establish the stereospecificity of the reaction, the same experiments were conducted on a sample of *trans*-**22** containing substantial amounts of *cis*-**22** (dr = 55:45). All subsequent products were obtained as mixtures of diastereomers (dr = 55:45). These results are not consistent with the mechanisms shown in Schemes 2B and C. The stereospecific retention of configuration, however, is consistent with the direct displacement mechanism involving activation of the OOH group into an electrophilic entity that reacts with the nucleophilic hydroxyl group (Scheme 2A).^69^

**Scheme 4:**
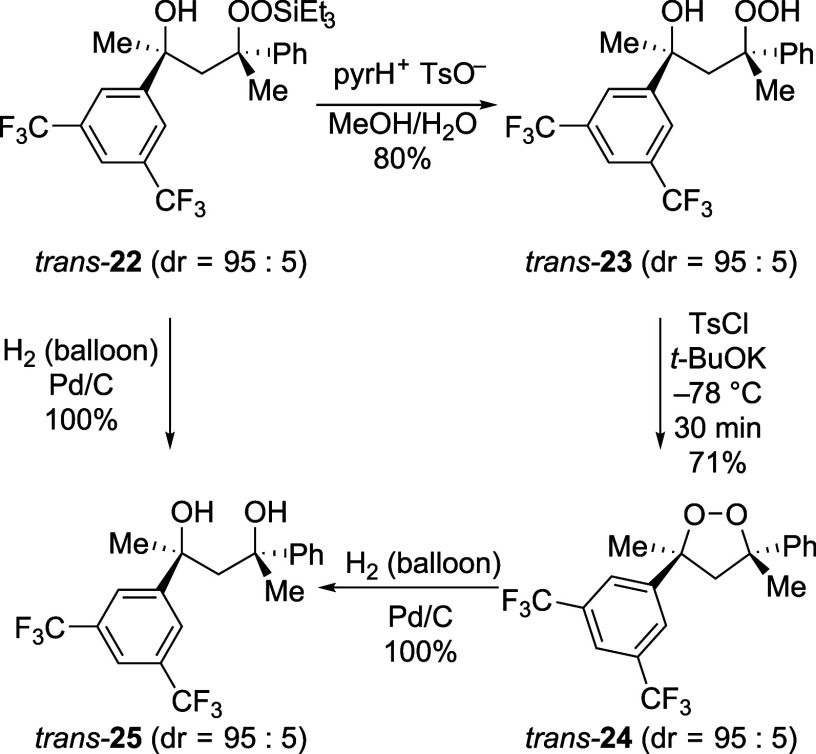
Formation of the Cyclic Peroxide Occurred with Retention of Configuration

An isotopic labeling experiment using ^17^O-labeled starting materials and analysis of the reaction mixture by ^17^O NMR spectroscopy^70^ provided further evidence that the reaction proceeded by oxygen–oxygen bond formation (eq 3). The ^17^O-labeled hydroperoxide **13-** ^**17**^ **O** was prepared with 2% enrichment in the heavy isotope.^71^ Treatment of this labeled alcohol (whose ^17^O chemical shift was δ 40 ppm) with TsCl and *t*-BuOK at −78 °C formed cyclic peroxide **14-** ^**17**^ **O**. This compound also showed significant isotopic labeling above background (^17^O represents only 0.04% of oxygen atoms at natural abundance^70^) with a chemical shift of δ 296 ppm, consistent with the chemical shift of an oxygen atom in a cyclic peroxide.^72–74^ The position of the oxygen atom is inferred by considering the stoichiometry and the other mechanistic results, although it cannot be assigned unambiguously. Nevertheless, the fact that the oxygen atom of the hydroxyl group is retained in the product is inconsistent with mechanisms involving tosylation at the hydroxyl group, where the hydroxyl group, and thus the ^17^O label, would be absent in the final cyclic peroxide (Schemes 2B and C). The results are also inconsistent with any mechanism where the labeled oxygen atom of the hydroxyl group is not converted to one of the oxygen atoms of the cyclic peroxide. Instead, the labeling is consistent with one oxygen atom of the cyclic peroxide being derived from the hydroxyl group of **13-** ^**17**^ **O**, and one oxygen atom being derived from the hydroperoxyl group. Taken together, the results shown in Scheme 4 and eq 3 suggest that the cyclic peroxide was formed by an oxygen–oxygen bond formation (Scheme 2A).
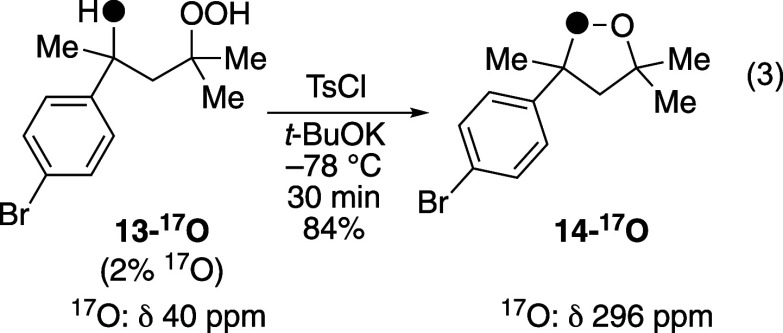


The base-mediated cyclization to form oxygen–oxygen bonds was general for a number of substrates (Chart [Fig chart1]). In addition to the substrates shown in earlier schemes and equations, a range of five-membered cyclic peroxides was formed from substrates in good yields within 5–30 min at −78 °C (the compounds are drawn so their regioselectivities correspond to structures **26** and **27**). The structure of cyclic peroxide **36** was established unambiguously by X-ray crystallography.^71^ The reaction was general with respect to the substitution near the hydroxyl group, with primary, secondary, and tertiary hydroxyl groups serving as effective nucleophiles. The reactions were general as long as the substrate was a tertiary hydroperoxide. With a secondary hydroperoxide, more decomposition products were observed (likely by elimination reactions to form carbonyl-containing products,^75^ which were detected by NMR spectroscopic analysis in reaction mixtures containing cyclic peroxide **37**). Successful applications of this reaction with secondary and primary hydroperoxides will likely depend upon the development of conditions that are less basic. The synthesis of spirobicyclic five-membered cyclic peroxide **38** suggests that this reaction can be applied to the synthesis of a family of ferroptosis-inducing cyclic peroxides.^44,68,76^ The synthesis of cyclic peroxide **39** from a primary alcohol underscores how much more rapid the sulfonylation of the hydroperoxide functional group is compared to that of a hydroxyl group under these conditions. The synthesis of *trans*-fused bicyclic peroxide **40** provides additional evidence that the cyclization proceeds with retention of configuration at the carbon atoms, which is consistent with oxygen–oxygen bond formation. The reaction could also be extended to form a six-membered-ring cyclic peroxide (**41**).

**1 chart1:**
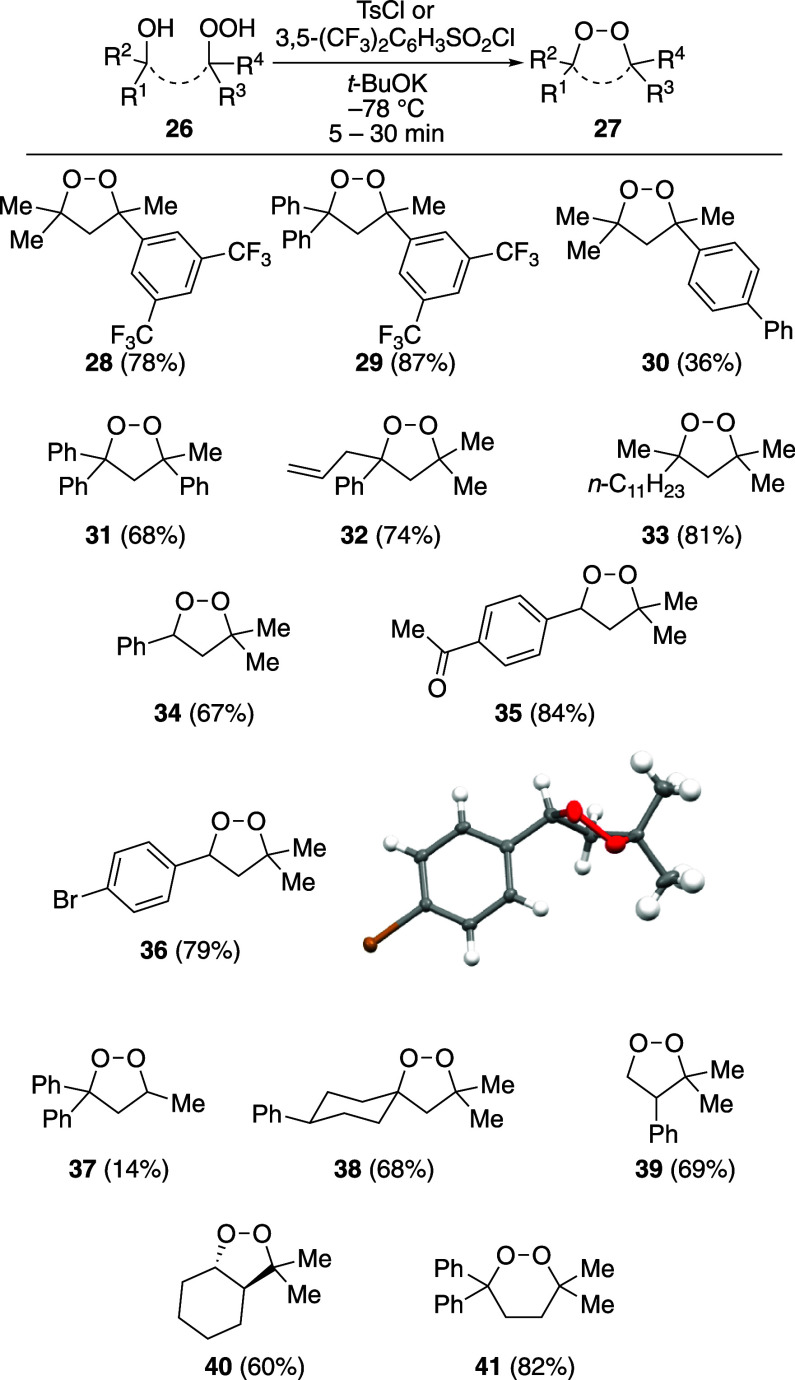
Scope of Oxygen–Oxygen Bond-Forming Reaction[Fn chart1-fn1]

a Reactions of substrates **26** to form endoperoxides **27**. The products are oriented as depicted in the equation; details of the reaction conditions are provided as Supporting Information.

## Conclusion

In conclusion, we demonstrate a new method for the formation of oxygen–oxygen bonds, a bond-forming process that has not been preparatively useful for the synthesis of alkyl peroxides. The reaction, which involves oxygen atoms acting as both nucleophile and electrophile, is fast, robust, stereospecific, and general for the synthesis of cyclic peroxides. The results of mechanistic studies are inconsistent with three alternative mechanisms, and stereochemical studies and labeling experiments are consistent with the oxygen–oxygen bond-forming reaction proceeding via intermediates that are not radicals. Considering that cyclic peroxides exhibit a range of useful and selective biological activities, as illustrated in Scheme 1D, the synthesis of peroxides by formation of the oxygen–oxygen bond complements other methods that have this bond already intact in the starting materials.^22^

## Supplementary Material



## Notes

Experimental details; characterization; stereochemical and structural proofs; computational details; crystallographic information for compound **36**, and ^1^H, ^13^C­{^1^H}, ^19^F­{^1^H}, and ^17^O spectra (PDF)
