# Supplementary material for: Oxygen–Oxygen Bond-Forming Reactions for the Synthesis of Cyclic Peroxides
Source: J Am Chem Soc. 2026 May 8;148(19):20122–8. doi: 10.1021/jacs.6c04588 (PMC13195650; doi:10.1021/jacs.6c04588)

Oxygen–Oxygen Bond-Forming Reactions for the Synthesis of Cyclic Peroxides

Authors: Farhan A. Chowdhury[1], Elena J. Helm[1], and K. A. Woerpel[1]*

[1]Department of Chemistry, New York University, 100 Washington Square East, New York, NY 10003 USA
Email: kwoerpel@nyu.edu

Supporting Information

Table of Contents

I. General Information S2
II. Synthesis of Substrates S3
A. Synthesis of Alkenes S3
B. Synthesis of Peroxides S12
C. Synthesis of Endoperoxides S21
III. Stereochemical Correlations and Proofs S30
A. Synthesis of Substrates for Stereochemical Proof for O–O Bond Formation S30
B. Assignment of Relative Stereochemical Configuration for Endoperoxide 24 .... S37
C. Assignment of Relative Stereochemical Configuration for Endoperoxide 40 .... S38
D. Assignment of Relative Stereochemical Configuration for Alkene S5 .... S38
IV. Structural Proofs S39
A. Synthesis for Structural Proof for Benzoylated Peroxide 21 .... S39
B. Structural Proof for Benzoylated Peroxide 21 .... S42
V. Synthesis for ^{17}O-labeled Substrates S42
VI. Radical Quenching Experiments S44
VII. Miscellaneous Mechanistic Experiments S45
VIII. Crystallographic Data of Endoperoxide 36 .... S47
IX. Computational Investigations of the Cyclization Reaction S48
X. References S55
XI. NMR Spectra S56

I. General Information

^{1}H NMR and $^{13}C\{^{1}H\}$ NMR spectra were obtained at room temperature using Bruker AVIII-400 (400 and 100 MHz, respectively) and Bruker AVIIIHD-400 (400 and 100 MHz, respectively) spectrometers. $^{19}F\{^{1}H\}$ NMR spectra and ^{17}O NMR spectra were obtained at room temperature using an AVIII-400 (377 MHz and 54 MHz, respectively) spectrometer. All spectroscopic data are reported as follows: chemical shifts are reported in ppm and are referenced to residual solvent on the δ scale (^{1}H NMR: $CDCl_3$ δ 7.26 ppm, C_6D_6 δ 7.16 ppm, $(CD_3)_2SO)$ δ 2.50 ppm; ^{13}C NMR: $CDCl_3$ δ 77.16 ppm, C_6D_6 δ 128.06 ppm, $(CD_3)_2SO)$ δ 39.52 ppm), multiplicity (s = singlet, br = broad, d = doublet, t = triplet, q = quartet, sept = septet, m = multiplet), coupling constants (Hz), and integration. $^{19}F\{^{1}H\}$ NMR spectra are externally referenced to trifluoroacetic acid ($^{19}F\{^{1}H\}$ NMR: $CDCl_3$ δ – 76.55). ^{17}O NMR spectra are externally referenced to water (^{17}O NMR: $CDCl_3$ δ 0.00 ppm). Ratios of products were derived from $^{13}C\{^{1}H\}$ NMR integrations using diagnostic peaks in the crude reaction mixture.[1] Multiplicity of carbon peaks were defined using HSQC experiments. Infrared (IR) spectra were recorded using a Thermo Nicolet AVATAR Fourier Transform IR spectrometer using attenuated total reflectance (ATR). High-resolution mass spectra were acquired on an Agilent 6224 Accurate-Mass time-of-flight spectrometer and were obtained using peak matching. The ionization sources used was electrospray ionization (ESI). Analytical thin layer chromatography was performed on silica get 60 Å F254 plates. Liquid chromatography was performed using automated flash column chromatography of the indicated solvent system on silica gel (SiO_2) 60 (230–400 mesh). All reactions were run under a nitrogen atmosphere in glassware that had been flame-dried under vacuum. Non-deuterated solvents were purified via the Pure Solv-MD Standard Design Solvent Purification System before use. Aqueous solutions were prepared from nanopore water with a resistivity over 18 MΩ-cm. Unless otherwise stated, all reagents and substrates were commercially available.

II. Synthesis of Substrates

A. Synthesis of Alkenes

Standard procedure A for alkene formation:

To a solution of the ketone (1.0 equiv) in dry THF (0.2 M) was added the bromide (1.5 equiv), then sodium iodide (1.5 equiv). After 5 min, indium powder was added (1.1 equiv, 99.9% purity). The reaction mixture was stirred vigorously at 22 ºC for 18 h then was diluted with a saturated solution of aqueous NH_4Cl (1 × 10 mL/mmol of ketone). The layers were separated, and the aqueous layer was extracted with EtOAc (3 × 10 mL/mmol of ketone). The combined organic extracts were washed with brine (1 ×10 mL/mmol of ketone), dried over Na_2SO_4, filtered, and concentrated under reduced pressure to afford the crude product. Purification by automated column chromatography provided the desired alkene.

Standard procedure B for alkene formation:

A solution of the ketone (1.0 equiv) in THF/H_2O (1:1 v/v, 0.1 M) was heated to 60 °C in an oil bath. Methallyl bromide (1.0 equiv) was added dropwise, followed by rapid addition of indium powder (1.1 equiv, 99.9% purity). The reaction mixture was heated at reflux for 48 h. Progress of the reaction was monitored by TLC until full consumption of the starting ketone was observed. The mixture was cooled to 22 ºC and diluted with EtOAc (10 mL/mmol of ketone). The layers were separated, and the aqueous layer was extracted with EtOAc (3 × 10 mL/mmol of ketone). The combined organic extracts were washed with brine (1 × 10 mL/mmol of ketone), dried over Na_2SO_4, filtered, and concentrated under reduced pressure to afford the crude product. Purification by automated column chromatography provided the desired alkene.

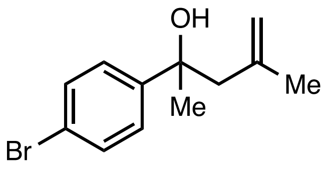

2-(4-Bromophenyl)-4-methylpent-4-en-2-ol (12). 1-(4-Bromophenyl)ethan-1-one (1.99 g, 10.0 mmol) was subjected to the standard procedure A for alkene formation using dry THF (50 mL), methallyl bromide (1.51 mL, 15.0 mmol), sodium iodide (2.25 g, 15.0 mmol), and indium powder (1.26 g, 11.0 mmol). Purification by automated column chromatography (0-20% EtOAc in hexanes) provided the desired alkene **12** as a clear oil (2.24 g, 88%):

^{1}H NMR (400 MHz, $CDCl_3$) δ 7.45 (d, J = 8.6 Hz, 2H), 7.32 (d, J = 8.6 Hz, 2H), 4.90 (s, 1H), 4.74 (s, 1H), 2.61 (d, J = 13.4 Hz, 1H), 2.49 (d, J = 13.4 Hz, 1H), 2.31 (s, 1H), 1.53 (s, 3H), 1.42 (s, 3H);

$^{13}C\{^1H\}$ NMR (100 MHz, $CDCl_3$) δ 147.2 (C), 142.3 (C), 131.3 (CH), 126.9 (CH), 120.6 (C), 116.2 (CH_2), 73.1 (C), 51.9 (CH_2), 30.9 (CH_3), 24.4 (CH_3);

IR (ATR) 3458, 2975, 2930, 1487, 1008, 822 cm^{-1};

HRMS (ESI) *m/z:* $[(M + H)]^+$ Calcd for $C_{12}H_{16}BrO$ 255.0379; Found 255.0367.

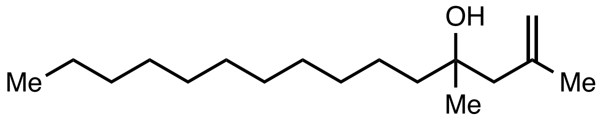

2,4-Dimethylpentadec-1-en-4-ol (S1). Tridecan-2-one (0.595 g, 3.00 mmol) was subjected to the standard procedure A for alkene formation using dry THF (15 mL), methallyl bromide (454 µL, 4.50 mmol), sodium iodide (0.675 g, 4.50 mmol), and indium powder (0.344 g, 3.00 mmol). Purification by automated column chromatography (0-20% EtOAc in hexanes) provided the desired alkene **S1** as a clear oil (0.666 g, 87%):

^1H NMR (400 MHz, $CDCl_3$) δ 4.93 (s, 1H), 4.79 (s, 1H), 2.22 (d, J = 13.3 Hz, 1H), 2.16 (d, J = 13.3 Hz, 1H), 1.84 (s, 3H), 1.54 (s, 1H), 1.50–1.42 (m, 2H), 1.40–1.20 (m, 18H), 1.17 (s, 3H), 0.88 (t, J = 6.7 Hz, 3H);

$^{13}C\{^1H\}$ NMR (100 MHz, $CDCl_3$) δ 143.1 (C), 114.9 (CH_2), 72.5 (C), 49.4 (CH_2), 42.8 (CH_2), 32.1 (CH_2), 30.4 (CH_2), 29.81 (CH_2, broad), 29.78 (CH_2, broad), 29.5 (CH_2), 27.1 (CH_3), 25.2 (CH_3), 24.3 (CH_2), 22.8 (CH_2), 14.3 (CH_3);

IR (ATR) 3446, 2922, 2852, 1465, 1375, 888 cm^{-1};

HRMS (ESI) *m/z:* $[(M + Na)]^+$ Calcd for $C_{17}H_{34}NaO$ 277.2507; Found 277.2507.

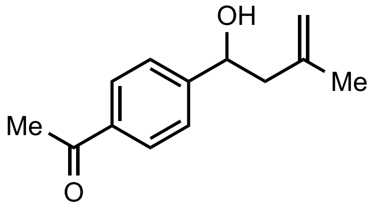

1-(4-(1-Hydroxy-3-methylbut-3-en-1-yl)phenyl)ethan-1-one (S2). 4-Acetylbenzaldehyde (1.00 g, 6.76 mmol) was subjected to the standard procedure B for alkene formation using THF/H_2O (1:1 v/v, 34 mL THF and 34 mL H_2O), methallyl bromide (702 µL, 6.76 mmol), indium powder (0.855 g, 7.44 mmol). The reaction mixture was heated at reflux for 48 h. Purification by automated column chromatography (0-20% EtOAc in hexanes) provided alkene **S2** as a clear oil (0.608 g, 44%). The spectroscopic data are consistent with the data reported:[2]

^{1}H NMR (400 MHz, $CDCl_3$) δ 7.90–7.85 (m, 2H), 7.41–7.39 (m, 2H), 4.88 (s, 1H), 4.79 (s, 1H), 2.53 (s, 3H), 1.74 (s, 3H);

$^{13}C\{^{1}H\}$ NMR (100 MHz, $CDCl_3$) δ 197.7 (C), 149.5 (C), 141.8 (C), 136.3 (C), 128.8 (CH), 125.9 (CH), 114.6 (CH_2), 70.8 (CH), 48.4 (CH_2), 31.6 (CH_2), 26.6 (CH_2), 21.0 (CH_3), 14.2 (CH_3).

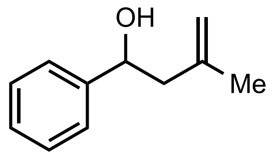

3-Methyl-1-phenylbut-3-en-1-ol (S3). Benzaldehyde (640 μL, 6.03 mmol) was subjected to the standard procedure B for alkene formation using THF/H_2O (1:1 v/v, 30 mL THF and 30 mL H_2O), methallyl bromide (482 μL, 6.03 mmol), indium powder (0.756 g, 6.63 mmol). The reaction mixture was heated at reflux for 48 h. Purification by automated column chromatography (0-20% EtOAc in hexanes) provided alkene **S3** as a clear oil (0.969 g, 99%). The spectroscopic data are consistent with the data reported:[3]

^{1}H NMR (400 MHz, $CDCl_3$) δ 7.40–7.35 (m, 4H), 7.30–7.28 (m, 1H), 4.94–4.81 (m, 3H), 2.45–2.43 (m, 2H), 2.24 (br s, 1H), 1.81 (s, 3H);

$^{13}C\{^{1}H\}$ NMR (100 MHz, $CDCl_3$) δ 144.2 (C), 142.5 (C), 128.5 (CH), 127.6 (CH), 125.9 (CH), 114.2 (CH_2), 71.6 (CH), 48.5 (CH_2), 22.5 (CH_3).

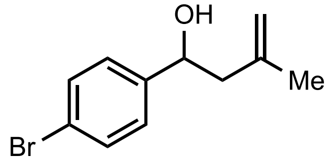

1-(4-Bromophenyl)-3-methylbut-3-en-1-ol (S4). 4-Bromobenzaldehyde (1.41 g, 7.62 mmol) was subjected to the standard procedure B for alkene formation using THF/H_2O (1:1 v/v, 38 mL THF and 38 mL H_2O), methallyl bromide (792 μL, 7.62 mmol), indium powder (0.955 g, 8.38 mmol). The reaction mixture was heated at reflux for 48 h. Purification by automated column chromatography (0-20% EtOAc in hexanes) provided alkene **S4** as a clear oil (1.76 g, 95%). The spectroscopic data are consistent with the data reported:[4]

^{1}H NMR (400 MHz, $CDCl_3$) δ 7.47–7.45 (m, 2H), 7.26–7.23 (m, 2H), 4.93–4.92 (m, 1H), 4.84–4.83 (m, 1H), 4.77–4.74 (m, 1H), 2.38–2.36 (m, 2H), 1.78 (s, 3H);

$^{13}C\{^{1}H\}$ NMR (100 MHz, $CDCl_3$) δ 143.3 (C), 142.2 (C), 131.7 (C), 127.7 (CH), 121.4 (CH), 114.6 (CH_2), 71.0 (CH), 48.5 (CH_2), 22.5 (CH_3).

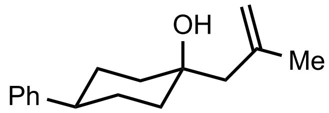

(1S,4S)-1-(2-Methylallyl)-4-phenylcyclohexan-1-ol (S5). 4-phenylcyclohexanone (0.500 g, 2.87 mmol) was subjected to the standard procedure B for alkene formation using THF/H_2O (1:1 v/v, 14 mL THF and 14 mL H_2O), methallyl bromide (447 µL, 4.31 mmol), indium powder (0.359 g, 3.16 mmol). The reaction mixture was heated at reflux for 48 h. Purification by automated column chromatography (0-20% EtOAc in hexanes) provided one diastereomer of alkene **S5** as a clear oil (0.345 g, 52%). The spectroscopic data are consistent with the data reported.[5] The stereochemistry of the major diastereomer was assigned based on the coupling values of the methylene protons, and in comparison to the allyl variant of the substrate (described in Section III, D of the Supporting Information):

^{1}H NMR (400 MHz, $CDCl_3$) δ 7.31–7.16 (m, 5H), 4.96 (br s, 1H), 4.79 (br s, 1H), 2.50–2.44 (m, 1H), 2.22 (br s, 2H), 1.92–1.71 (m, 8H), 1.58–1.50 (m, 3H);

^{13}C{^{1}H} NMR (100 MHz, $CDCl_3$) δ 147.3 (C), 142.4 (C), 128.3 (CH), 126.9 (CH), 126.0 (CH), 114.9 (CH_2), 70.0 (C), 51.7 (CH), 44.0 (CH_2), 37.8 (CH_2), 29.4 (CH_2), 25.5 (CH_3).

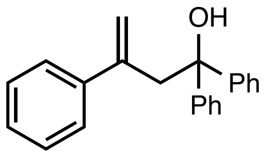

1,1,3-Triphenylbut-3-en-1-ol (S6). 1,3-Diphenylbut-3-en-1-one was prepared according to a reported procedure.[6] To a cooled (0 ºC) solution of 1,3-diphenylbut-3-en-1-one (0.323 g, 1.45 mmol, 1.00 equiv) in dry THF (10 mL) was added PhMgCl (3.0 M, 727 µL, 2.2 mmol, 1.5 equiv). The reaction mixture was stirred and warmed to 22 ºC for 1 h. Excess reagent was quenched with a saturated solution of aqueous NH_4Cl (10 mL). The layers were separated, and the aqueous layer was extracted with EtOAc (3 × 10 mL). The combined organic extracts were washed with brine (1 × 10 mL), dried over Na_2SO_4, filtered, and concentrated under reduced pressure to afford the crude product. Purification by automated column chromatography (0-20% EtOAc in hexanes) provided the desired alkene **S6** as a clear oil (0.370 g, 85%):

^{1}H NMR (400 MHz, $CDCl_3$) δ 7.42–7.36 (m, 4H), 7.25–7.11 (m, 11H), 5.25 (d, J = 1.5 Hz, 1H), 4.97 (m, 1H), 3.58 (d, J = 0.9 Hz, 2H), 2.55 (br s, 1H);

^{13}C{^{1}H} NMR (100 MHz, $CDCl_3$) δ 146.6 (C), 145.0 (C), 142.7 (C), 128.3 (CH), 128.1 (CH), 127.4 (CH), 126.9 (CH), 126.7 (CH), 126.2 (CH), 118.5 (CH_2), 78.0 (C), 47.4 (CH_2);

IR (ATR) 3561, 3083, 3056, 3024, 1447, 697 cm^{-1};

HRMS (ESI) *m/z:* $[(M + Na)]^+$ Calcd for $C_{22}H_{20}NaO$ 323.1412; Found 323.1408.

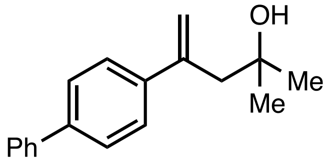

4-([1,1'-Biphenyl]-4-yl)-2-methylpent-4-en-2-ol (S7). 3-Bromoprop-1-en-2-yl)-1,1'-biphenyl was prepared according to a reported procedure.[7] To a solution of 3-bromoprop-1-en-2-yl)-1,1'-biphenyl (0.641 g, 2.35 mmol, 1.00 equiv) and acetone (174 µL, 2.35 mmol, 1.00 equiv) in THF (8 mL) and H_2O (2 mL) was added was indium powder (0.270 g, 2.35 mmol, 1.00 equiv). The reaction mixture was stirred vigorously for 18 h at 22 ºC then was diluted with a saturated solution of aqueous NH_4Cl (10 mL). The layers were separated, and the aqueous layer was extracted with EtOAc (3 × 10 mL). The combined organic extracts were washed with brine (1 × 10 mL), dried over Na_2SO_4, filtered, and concentrated under reduced pressure to afford the crude product. Purification by automated column chromatography (0-20% EtOAc in hexanes) provided the desired alkene **S7** as a white solid (0.232 g, 39%):

mp = 72 ºC–74 ºC;

^{1}H NMR (400 MHz, $CDCl_3$) δ 7.64–7.54 (m, 4H), 7.53–7.48 (m, 2H), 7.47–7.40 (m, 2H), 7.37–7.31 (m, 1H), 5.46 (d, *J* = 1.7 Hz, 1H), 5.17 (s, 1H), 2.79 (s, 2H), 1.17 (s, 6H);

$^{13}C\{^1H\}$ NMR (100 MHz, $CDCl_3$) δ 145.5 (C), 141.3 (C), 140.7 (C), 140.5 (C), 128.9 (CH), 127.5 (CH), 127.3 (CH), 127.1 (CH), 127.0 (CH), 117.4 (CH_2), 71.0 (C), 48.7 (CH_2), 29.9 (CH_3);

IR (ATR) 3373, 2978, 2966, 2928, 1153, 769 cm^{-1};

HRMS (ESI) *m/z:* $[(M + Na)]^+$ Calcd for $C_{18}H_{20}NaO$ 275.1412; Found 275.1412.

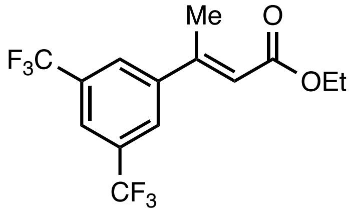

Ethyl-3-(3,5-bis(trifluoromethyl)phenyl)but-2-enoate (S8). To a cooled solution (0 ºC) of ethyl 2-(diethoxyphosphoryl)acetate (5.69 mL, 28.7 mmol, 1.50 equiv) in dry THF (20 mL) was added NaH (1.15 g, 29 mmol, 1.5 equiv, 60% w/w). The reaction mixture was warmed to 22 ºC and stirred for 30 min. 1-(3,5-Bis(trifluoromethyl)phenyl)ethan-1-one (3.52 mL, 19.1 mmol, 1.00 equiv) was added to the reaction mixture, which was then stirred for 18 h. A saturated solution of aqueous NH_4Cl (20 mL) was added. The organic layer was separated. The aqueous

layer was extracted with EtOAc (3 × 20 mL). The combined organic extracts were washed with brine (1 × 20 mL), dried over Na_2SO_4, filtered, and concentrated under reduced pressure to afford the crude product. The crude product was purified by automated column chromatography (0-20% EtOAc in hexanes) to yield the desired ester **S8** as a mixture of isomers (*E:Z* = 91:9) as a clear oil (5.63 g, 90%). The spectroscopic data are consistent with the data reported:[8]

E isomer:

^{1}H NMR (400 MHz, $CDCl_3$) δ 7.88 (s, 2H), 7.86 (s, 1H), 6.89 (q, *J* = 1.4 Hz, 1H), 4.24 (q, *J* = 7.1 Hz, 2H), 2.60 (d, *J* = 1.4 Hz, 3H), 1.32 (t, *J* = 7.1 Hz, 3H);

^{13}C{^{1}H} NMR (100 MHz, $CDCl_3$) δ 166.0 (C), 151.9 (C), 144.5 (C), 132.2 (C, q, $^2J_{C\text{–}F}$ = 33.3 Hz), 126.6 (CH, m), 123.3 (CF_3, q, $^1J_{C\text{–}F}$ = 272.8 Hz), 122.5 (CH, m), 120.3 (CH), 60.4 (CH_2), 17.9 (CH_3), 14.3 (CH_3);

^{19}F{^{1}H} NMR (377 MHz, $CDCl_3$) δ –64.1.

Z isomer:

^{1}H NMR (400 MHz, $CDCl_3$) δ 7.81 (s, 1H), 7.64 (s, 2H), 6.02 (q, *J* = 1.4 Hz, 1H), 3.98 (q, *J* = 7.1 Hz, 2H), 2.21 (d, *J* = 1.4 Hz, 3H), 1.05 (t, *J* = 7.1 Hz, 3H);

^{13}C{^{1}H} NMR (100 MHz, $CDCl_3$) δ 165.1 (C), 151.8 (C), 143.1 (C), 131.4 (C, q, $^2J_{C\text{–}F}$ = 33.3 Hz), 127.5 (CH, m), 123.4 (CF_3, q, $^1J_{C\text{–}F}$ = 272.8 Hz), 121.5 (CH, m), 120.4 (CH), 60.3 (CH_2), 26.8 (CH_3), 13.8 (CH_3);

^{19}F{^{1}H} NMR (377 MHz, $CDCl_3$) δ –64.0.

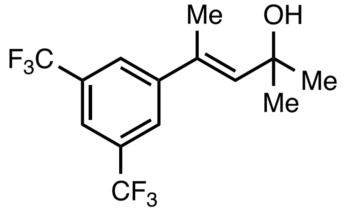

4-(3,5-Bis(trifluoromethyl)phenyl)-2-methylpent-3-en-2-ol (S9). To a cooled solution (0 ºC) of ester **S8** (4.13 g, 12.6 mmol, 1.00 equiv) in dry THF (20 mL) was added MeMgCl (3.0 M, 16.9 mL, 51 mmol, 4.0 equiv). The reaction mixture was warmed to 22 ºC and stirred for 18 h. A saturated solution of aqueous NH_4Cl (20 mL) was added. The organic layer was separated. The aqueous layer was extracted with EtOAc (3 × 20 mL). The combined organic extracts were washed with brine (1 × 20 mL), dried over Na_2SO_4, filtered, and concentrated under reduced pressure to afford the crude product. The crude product was purified by automated column

chromatography (0-20% EtOAc in hexanes) to yield the desired alcohol **S9** as a mixture of isomers (*E*:*Z* = 94:6) as a clear oil (2.39 g, 61%):

IR (ATR) 3446, 2922, 2852, 1465, 1375, 888 cm^{-1};

HRMS (ESI) *m/z:* $[(M + Na)]^+$ Calcd for $C_{14}H_{14}F_6NaO$ 335.0847; Found 335.0835.

E isomer:

^{1}H NMR (400 MHz, $CDCl_3$) δ 7.77 (s, 2H), 7.64 (s, 1H), 5.92 (q, *J* = 1.3 Hz, 1H), 2.33 (d, *J* = 1.3 Hz, 3H), 1.50 (s, 6H);

$^{13}C\{^1H\}$ NMR (100 MHz, $CDCl_3$) δ 146.9 (C), 138.5 (CH), 135.1 (C), 131.6 (C, q, $^2J_{C–F}$ = 33.0 Hz), 126.2 (CH), 123.6 (CF_3, q, $^1J_{C–F}$ = 273.5 Hz), 120.7 (CH, m), 71.5 (C), 31.2 (CH_3), 16.8 (CH_3);

$^{19}F\{^1H\}$ NMR (377 MHz, $CDCl_3$) δ –63.9.

Characteristic peaks for Z isomer:

^{1}H NMR (400 MHz, $CDCl_3$) δ 5.72 (q, *J* = 1.3 Hz, 1H), 2.01 (d, *J* = 1.3 Hz, 3H), 1.21 (s, 6H);

$^{19}F\{^1H\}$ NMR (377 MHz, $CDCl_3$) δ –64.0.

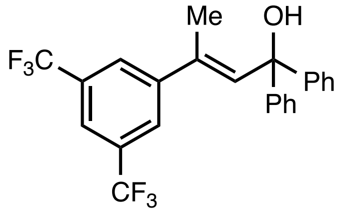

3-(3,5-Bis(trifluoromethyl)phenyl)-1,1-diphenylbut-2-en-1-ol (S10). To a cooled solution (0 ºC) of ester **S8** (1.00 g, 3.07 mmol, 1.00 equiv) in dry THF (10 mL) was added PhMgCl (2.0 M, 6.14 mL, 12 mmol, 4.0 equiv). The reaction mixture was warmed to 22 ºC and stirred for 18 h. A saturated solution of aqueous NH_4Cl (10 mL) was added. The organic layer was separated. The aqueous layer was extracted with EtOAc (3 × 10 mL). The combined organic extracts were washed with brine (1 × 10 mL), dried over Na_2SO_4, filtered, and concentrated under reduced pressure to afford the crude product. The crude product was purified by automated column chromatography (0-20% EtOAc in hexanes) to yield the desired alcohol **S10** as a mixture of isomers (*E*:*Z* = 92:8) as a pale yellow oil (1.22 g, 91%):

IR (ATR) 3456, 3088, 3061, 3028, 1275, 1126 cm^{-1};

HRMS (ESI) *m/z:* $[(M + Na)]^+$ Calcd for $C_{24}H_{18}F_6NaO$ 459.1160; Found 459.1159.

E isomer:

^{1}H NMR (400 MHz, $CDCl_3$) δ 7.82 (s, 2H), 7.78 (s, 1H), 7.51–7.44 (m, 4H), 7.40–7.33 (m, 4H), 7.31–7.27 (m, 2H), 6.62 (s, 1H), 2.50 (s, 1H), 2.04 (s, 3H);

$^{13}C\{^1H\}$ NMR (100 MHz, $CDCl_3$) δ 147.3 (C), 146.3 (C), 138.7 (C), 137.9 (CH), 132.0 (C, q, $^2J_{C–F}$ = 33.3 Hz), 128.6 (CH), 127.5 (CH), 126.4 (CH), 126.2 (CH, m), 123.5 (CF_3, q, $^1J_{C–F}$ = 272.5 Hz), 121.2 (CH, m), 79.0 (C), 18.0 (CH_3);

$^{19}F\{^1H\}$ NMR (377 MHz, $CDCl_3$) δ –63.8.

Characteristic peaks for Z isomer:

^{1}H NMR (400 MHz, $CDCl_3$) δ 6.45 (s, 1H), 2.15 (s, 3H);

$^{19}F\{^1H\}$ NMR (377 MHz, $CDCl_3$) δ –64.1.

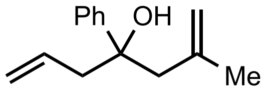

2-Methyl-4-phenylhepta-1,6-dien-4-ol (S11). 3-Methyl-1-phenylbut-3-en-1-one was prepared from **S3** according to a reported procedure.[6] To a cooled (0 °C) solution of 3-methyl-1-phenylbut-3-en-1-one (0.601 g, 3.71 mmol) in Et_2O (37 mL, 0.1 M) was added allylmagnesium bromide solution (1.0 M in Et_2O, 10 mmol) dropwise over 5 min. After 20 min, the mixture was quenched with NH_4Cl (50 mL). The layers were separated, and the aqueous layer was extracted with CH_2Cl_2 (3 x 50 mL). The combined organic layers were washed with brine (1 x 100 mL), dried over Na_2SO_4, filtered, and concentrated in vacuo. Alkene **S11** was isolated as a colorless oil (0.271 g, 36%) and used without further purification:

^{1}H NMR (400 MHz, $CDCl_3$) δ 7.34–7.32 (m, 2H), 7.27–7.23 (m, 2H), 7.16–7.13 (m, 1H), 5.59–5.48 (m, 1H), 5.04–4.95 (m, 2H), 4.78–4.77 (m, 1H), 4.62 (s, 1H), 2.67–2.62 (m, 1H), 2.57–2.42 (m, 3H), 1.32 (s, 3H);

$^{13}C\{^1H\}$ NMR (100 MHz, $CDCl_3$) δ 146.2 (C), 142.4 (C), 133.6 (CH), 128.0 (CH_2), 126.5 (CH), 125.4 (CH_2), 118.9 (CH), 115.6 (CH), 74.8 (C), 50.5 (CH_2), 47.6 (CH_2), 24.3 (CH_3);

IR (ATR) 3587, 2966, 1665, 1378, 930, 826 cm^{-1};

HRMS (ESI) *m/z* calcd for $C_4H_{19}O$ $[M + H]^+$: 202.1358, found 202.1360.

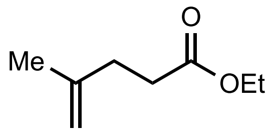

Ethyl 4-methyl-4-pentenoate (S12). *t*-BuOK (9.65 g, 86.2 mmol, 1.50 equiv) was added to methyltriphenylphosphonium bromide (30.77 g, 86.13 mmol, 1.500 equiv) in THF (200 mL, 0.43 M) at 0 °C. After stirring for 10 min, ethyl levulinate (8.28 g, 57.4 mmol, 1.00 equiv) was added dropwise. The reaction mixture was stirred for 16 h at 22 ºC and then concentrated in vacuo to afford a yellow solid. The residue was suspended in Et_2O (1000 mL) and filtered to remove insoluble phosphine oxide. The filtrate was concentrated in vacuo to give a yellow oil. Purification by automated column chromatography (0-5% EtOAc in hexanes) afforded alkene **S12** as a colorless oil (5.11 g, 63%). The spectroscopic data are consistent with the data reported:[9]

^{1}H NMR (400 MHz, $CDCl_3$) δ 4.76 (s, 1H), 4.71 (s, 1H), 4.15 (q, *J* = 7.05, 2H), 2.49–2.45 (m, 2H), 2.37–2.33 (m, 2H), 1.76 (s, 3H), 1.27 (t, *J* = 7.05, 3H);

^{13}C{^{1}H} NMR (100 MHz, $CDCl_3$) δ 173.4 (C), 144.2 (C), 110.3 (CH_2), 60.3 (CH_2), 32.7 (CH_2), 32.6 (CH_2), 22.5 (CH_3), 14.2 (CH_3).

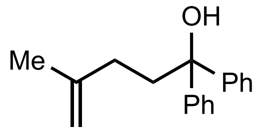

4-Methyl-1,1-diphenylpent-4-en-1-ol (S13). To a cooled (0 °C) solution of alkene **S12** (2.00 g, 14.1 mmol) in Et_2O (200 mL, 0.07 M) was added PhMgBr (28.2 mL, 1.0 M in Et_2O, 28 mmol) dropwise over 5 min. After 20 min, the mixture was quenched with NH_4Cl (200 mL). The layers were separated, and the aqueous layer was extracted with CH_2Cl_2 (3 x 100 mL). The combined organic layers were washed with brine (1 x 400 mL), dried over Na_2SO_4, filtered, and concentrated in vacuo. Purification by flash chromatography (0-2% EtOAc in hexanes) afforded alkene **S13** as a colorless oil (2.44 g, 86%). The spectroscopic data are consistent with the data reported:[10]

^{1}H NMR (400 MHz, $CDCl_3$) δ 7.47–7.43 (m, 4H), 7.36–7.32 (m, 4H), 7.27–7.23 (m, 2H), 4.75 (s, 1H), 4.72 (s, 1H), 2.48–2.44 (m, 2H), 2.06–2.02 (m, 2H), 1.76 (s, 3H);

^{13}C{^{1}H} NMR (100 MHz, $CDCl_3$) δ 146.9 (C), 146.2 (C), 128.2 (CH), 127.2 (CH), 125.9 (CH), 109.9 (CH_2), 78.3 (C), 39.8 (CH_2), 32.1 (CH_2), 22.8 (CH_3).

B. Synthesis of Peroxides

Standard procedure for peroxidation:

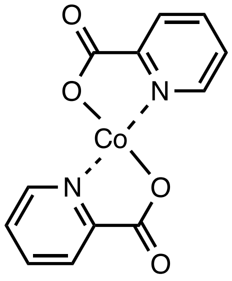

Co(pic)$_2$

Co(pic)$_2$ was prepared according to a reported procedure.[11] A solution of alkene (1.0 equiv) and Co(pic)$_2$ (10 mol%) in *i*-PrOH (0.03 M or 0.2 M) was sonicated for 5 min until the mixture was homogeneous. 1,1,3,3-Tetramethyldisiloxane (2.2 equiv) and *t*-BuOOH (1.0 M in CH_2Cl_2, 0.20 equiv) were added sequentially to the reaction mixture at 22 °C. The solution was purged with an oxygen balloon for 5 min to provide an O_2 atmosphere. The reaction mixture was brought to 35 ºC or 45 °C and allowed to stir for 18 h. The reaction mixture was concentrated in vacuo to yield a purple oil. Filtration of the crude material over a silica plug (50:50 EtOAc:hexanes) facilitated the removal of the cobalt catalyst, affording the hydroperoxide as a clear oil. This material was then subjected to further purification.

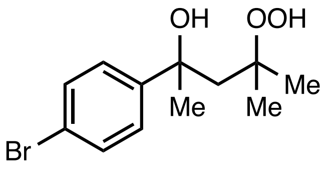

2-(4-Bromophenyl)-4-hydroperoxy-4-methylpentan-2-ol (13). Alkene **12** (0.706 g, 2.77 mmol) was subjected to the standard procedure for peroxidation at 35 ºC using Co(pic)$_2$ (0.084 g, 0.28 mmol), 1,1,3,3-tetramethyldisiloxane (1.08 mL, 6.09 mmol), *t*-BuOOH (0.553 mL, 1.0 M, 0.55 mmol), and *i*-PrOH (28 mL). Purification by automatic column chromatography (0-50% EtOAc in hexanes) afforded hydroperoxide **13** as a clear oil (0.700 g, 88%). The peroxide would sometimes solidify into a white solid in vacuo:

mp = 93 – 95 ºC;

^{1}H NMR (400 MHz, $CDCl_3$) δ 8.90 (s, 1H), 7.46 (d, J = 8.7 Hz, 2H), 7.35 (d, J = 8.7 Hz, 2H), 3.22 (s, 1H), 2.31 (d, J = 15.5 Hz, 1H), 2.10 (d, J = 15.5 Hz, 1H), 1.59 (s, 3H), 1.28 (s, 3H), 0.79 (s, 3H);

^{13}C{^{1}H} NMR (100 MHz, $CDCl_3$) δ 147.4 (C), 131.4 (CH), 126.8 (CH), 120.7 (C), 83.6 (C), 75.2 (C), 49.3 (CH_2), 33.3 (CH_3), 27.6 (CH_3), 25.4 (CH_3);

IR (ATR) 3293, 2977, 2929, 1740, 1487, 1394, 1365, 1077, 1008 cm^{-1};

HRMS (ESI) *m/z:* [(M + H)]$^+$ Calcd for $C_{12}H_{18}BrO_3$ 311.0259; Found 311.0268.

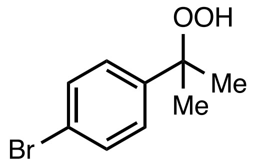

1-Bromo-4-(2-hydroperoxypropan-2-yl)benzene (19). 1-Bromo-4-(prop-1-en-2-yl)benzene was prepared according to a reported procedure.[12] 1-Bromo-4-(prop-1-en-2-yl)benzene (0.143 g, 0.723 mmol) was subjected to the standard procedure for peroxidation at 45 ºC using Co(pic)$_2$ (0.022 g, 0.072 mmol), 1,1,3,3-tetramethyldisiloxane (281 µL, 1.59 mmol), *t*-BuOOH (145 µL, 1.0 M, 0.15 mmol), and *i*-PrOH (7 mL). Purification by automatic column chromatography (0-50% EtOAc in hexanes) afforded hydroperoxide **19** as a clear oil (0.104 g, 62%):

^{1}H NMR (400 MHz, CDCl$_3$) δ 7.50 (d, J = 8.6 Hz, 2H), 7.34 (d, J = 8.6 Hz, 2H), 7.29 (br s, 1H), 1.59 (s, 6H);

^{13}C{^{1}H} NMR (100 MHz, CDCl$_3$) δ 143.9 (C), 131.7 (CH), 127.4 (CH), 121.6 (C), 83.8 (C), 26.2 (CH$_3$);

IR (ATR) 3384, 2982, 1489, 1395, 1265, 1108, 1097, 1009, 820 cm^{-1};

HRMS (ESI) m/z: [(M + Na)]$^+$ Calcd for $C_9H_{11}BrNaO_2$ 252.9840; Found 252.9844.

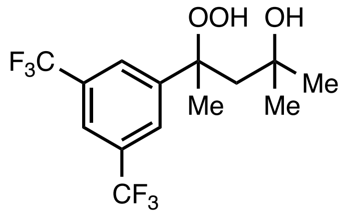

4-(3,5-Bis(trifluoromethyl)phenyl)-4-hydroperoxy-2-methylpentan-2-ol (S14). Alkene **S9** (0.618 g, 1.98 mmol) was subjected to the standard procedure for peroxidation at 45 ºC using Co(pic)$_2$ (0.060 g, 0.20 mmol), 1,1,3,3-tetramethyldisiloxane (770 µL, 4.35 mmol), *t*-BuOOH (396 µL, 1.0 M, 0.40 mmol), and *i*-PrOH (10 mL). Purification by automatic column chromatography (0-50% EtOAc in hexanes) afforded hydroperoxide **S14** as a clear oil (0.442 g, 65%):

^{1}H NMR (400 MHz, CDCl$_3$) δ 10.62 (br s, 1H), 7.88 (s, 2H), 7.77 (s, 1H), 2.39 (d, J = 15.6 Hz, 1H), 2.34 (d, J = 15.6 Hz, 1H), 1.93 (br s, 1H), 1.60 (s, 3H), 1.46 (s, 3H), 1.13 (s, 3H);

^{13}C{^{1}H} NMR (100 MHz, CDCl$_3$) δ 149.5 (C), 131.7 (C, q, $^2J_{C–F}$ = 33.1 Hz), 125.5 (CH, m), 123.5 (CF$_3$, q, $^1J_{C–F}$ = 272.7 Hz), 120.8 (CH, m), 84.8 (C), 72.6 (C), 48.3 (CH$_2$), 31.5 (CH$_3$), 31.4 (CH$_3$), 29.2 (CH$_3$);

$^{19}F\{^{1}H\}$ (377 MHz, $CDCl_3$) δ –63.8;

IR (ATR) 3335, 2976, 2923, 2851, 1278, 1132 cm^{-1};

HRMS (ESI) *m/z:* $[(M + Na)]^+$ Calcd for $C_{14}H_{16}F_6NaO_3$ 369.0901; Found 369.0885.

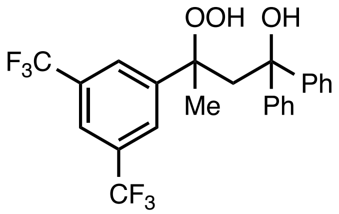

3-(3,5-Bis(trifluoromethyl)phenyl)-3-hydroperoxy-1,1-diphenylbutan-1-ol (S15). Alkene **S10** (0.770 g, 2.46 mmol) was subjected to the standard procedure for peroxidation at 45 ºC using Co(pic)$_2$ (0.075 g, 0.25 mmol), 1,1,3,3-tetramethyldisiloxane (958 µL, 5.42 mmol), *t*-BuOOH (493 µL, 1.0 M, 0.49 mmol), and *i*-PrOH (12 mL). Purification by automatic column chromatography (0-50% EtOAc in hexanes) afforded hydroperoxide **S15** as a clear oil (0.179 g, 21%):

^{1}H NMR (400 MHz, $CDCl_3$) δ 9.66 (s, 1H), 7.57–7.51 (m, 3H), 7.43–7.37 (m, 2H), 7.35–7.22 (m, 3H), 7.18–7.12 (m, 2H), 7.08–6.98 (m, 3H), 3.28 (d, *J* = 15.1 Hz, 1H), 3.09 (d, *J* = 15.1 Hz, 1H), 3.00 (s, 1H), 1.47 (s, 3H);

$^{13}C\{^{1}H\}$ NMR (100 MHz, $CDCl_3$) δ 147.6 (C), 146.6 (C), 144.4 (C), 131.1 (C, q, $^{2}J_{C\text{–}F}$ = 33.0 Hz), 128.7 (CH), 128.0 (CH), 127.9 (CH), 127.4 (CH), 126.4 (CH), 126.2 (CH), 125.3 (CH, m), 123.4 (CF_3, q, $^{1}J_{C\text{–}F}$ = 272.3 Hz) 120.5 (CH, m), 85.4 (C), 78.5 (C), 48.2 (CH_2), 27.8 (CH_3);

$^{19}F\{^{1}H\}$ (377 MHz, $CDCl_3$) δ –63.8;

IR (ATR) 3309, 2976, 2926, 2853, 1278, 1132 cm^{-1};

HRMS (ESI) *m/z:* $[(M + Na)]^+$ Calcd for $C_{24}H_{20}F_6NaO_3$ 493.1214; Found 493.1218.

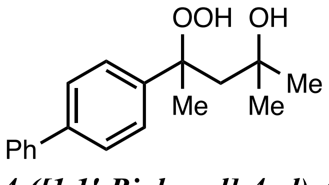

4-([1,1'-Biphenyl]-4-yl)-4-hydroperoxy-2-methylpentan-2-ol (S16). Alkene **S7** (0.232 g, 0.918 mmol) was subjected to the standard procedure for peroxidation at 35 ºC using Co(pic)$_2$ (0.028 g,

0.092 mmol), 1,1,3,3-tetramethyldisiloxane (357 μL, 2.02 mmol), *t*-BuOOH (184 μL, 1.0 M, 0.18 mmol), and *i*-PrOH (5 mL). Purification by automatic column chromatography (0-50% EtOAc in hexanes) afforded hydroperoxide **S16** as a clear oil (0.0851 g, 32%):

^{1}H NMR (400 MHz, $CDCl_3$) δ 10.03 (br s, 1H), 7.64–7.54 (m, 4H), 7.52–7.39 (m, 4H), 7.38–7.30 (m, 1H), 2.39 (d, *J* = 15.6 Hz, 1H), 2.33 (d, *J* = 15.6 Hz, 1H), 1.94 (br s, 1H), 1.65 (s, 3H), 1.39 (s, 3H), 1.11 (s, 3H);

^{13}C{^{1}H} NMR (100 MHz, $CDCl_3$) δ 145.2 (C), 140.8 (C), 139.7 (C), 128.9 (CH), 127.4 (CH), 127.2 (CH), 127.1 (CH), 125.3 (CH), 85.4 (C), 72.7 (C), 48.7 (CH_2), 32.2 (CH_3), 30.7 (CH_3), 29.1 (CH_3);

IR (ATR) 3215, 2966, 2937, 2880, 1487, 1246 cm^{-1};

HRMS (ESI) *m/z:* [(M + Na)]$^+$ Calcd for $C_{18}H_{22}NaO_3$ 309.1467; Found 309.1466.

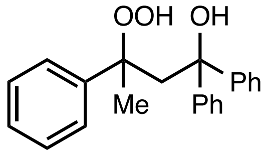

3-Hydroperoxy-1,1,3-triphenylbutan-1-ol (S17). Alkene **S6** (0.370 g, 1.23 mmol) was subjected to the standard procedure for peroxidation at 35 ºC using Co(pic)$_2$ (0.037 g, 0.12 mmol), 1,1,3,3-tetramethyldisiloxane (479 μL, 2.71 mmol), *t*-BuOOH (246 μL, 1.0 M, 0.25 mmol), and *i*-PrOH (6 mL). Purification by automatic column chromatography (0-50% EtOAc in hexanes) afforded hydroperoxide **S17** as a clear oil (0.238 g, 58%):

^{1}H NMR (400 MHz, $CDCl_3$) δ 8.44 (br s, 1H), 7.60–7.50 (m, 2H), 7.41–7.10 (m, 13H), 3.62 (br s, 1H), 3.13 (d, *J* = 15.6 Hz, 1H), 3.08 (d, *J* = 15.6 Hz, 1H), 1.27 (s, 3H);

^{13}C{^{1}H} NMR (100 MHz, $CDCl_3$) δ 147.9 (C), 147.0 (C), 145.5 (C), 128.7 (CH), 128.4 (CH), 128.3 (CH), 127.4 (CH), 127.03 (CH), 126.99 (CH), 126.1 (CH), 125.7 (CH), 125.1 (CH), 87.4 (C), 78.0 (C), 48.5 (CH_2), 25.3 (CH_3);

IR (ATR) 3397, 3087, 3058, 3030, 1447, 698 cm^{-1};

HRMS (ESI) *m/z:* [(M + Na)]$^+$ Calcd for $C_{22}H_{22}NaO_3$ 357.1467; Found 357.1468.

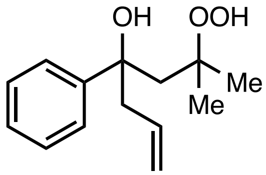

6-Hydroperoxy-6-methyl-4-phenylhept-1-en-4-ol (S18). Alkene **S11** (0.270 g, 1.34 mmol) was subjected to the standard procedure for peroxidation at 45 ºC using Co(pic)$_2$ (0.041 g, 0.14 mmol), 1,1,3,3-tetramethyldisiloxane (515 µL, 2.91 mmol), *t*-BuOOH (266 µL, 1.0 M, 0.27 mmol), and *i*-PrOH (45 mL). Purification by automatic column chromatography (0-50% EtOAc in hexanes) afforded hydroperoxide **S18** as a clear oil (0.088 g, 28%):

^{1}H NMR (400 MHz, CDCl$_3$) δ 7.29–7.26 (m, 2H), 7.21–7.16 (m, 2H), 7.11–7.06 (m, 1H), 5.36–5.24 (m, 1H), 5.04–4.96 (m, 2H), 2.67–2.60 (m, 1H), 2.39 (dd, *J* = 13.5, 9.3, 1H), 2.22 (d, *J* = 15.4, 1H), 1.94 (d, *J* = 15.4, 1H), 1.10 (s, 3H), 0.51 (s, 3H);

^{13}C{^{1}H} NMR (100 MHz, CDCl$_3$) δ 145.3 (C), 132.8 (CH), 128.2 (CH), 126.8 (CH), 125.4 (CH), 120.6 (CH$_2$), 82.8 (C), 76.0 (C), 49.3 (CH$_2$), 48.7 (CH$_2$), 27.9 (CH$_3$), 25.1 (CH$_3$);

IR (ATR) 3483, 3394, 1675, 1421, 1117, 655 cm^{-1};

HRMS (ESI) *m/z* calcd for $C_{14}H_{21}O_3$ [M + H]$^+$: 237.1412, found 237.1410.

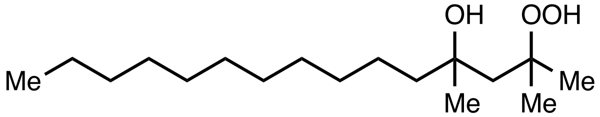

2-Hydroperoxy-2,4-dimethylpentadecan-4-ol (S19). Alkene **S1** (0.503 g, 1.98 mmol) was subjected to the standard procedure for peroxidation at 35 ºC using Co(pic)$_2$ (0.060 g, 0.20 mmol), 1,1,3,3-tetramethyldisiloxane (768 µL, 4.35 mmol), *t*-BuOOH (395 µL, 1.0 M, 0.40 mmol), and *i*-PrOH (10 mL). Purification by automatic column chromatography (0-50% EtOAc in hexanes) afforded hydroperoxide **S19** as a clear oil (0.410 g, 72%):

^{1}H NMR (400 MHz, CDCl$_3$) δ 1.92 (d, *J* = 15.5 Hz, 1H), 1.69 (d, *J* = 15.5 Hz, 1H), 1.59–1.51 (m, 2H), 1.37 (s, 3H), 1.33 (s, 3H), 1.32 (s, 3H), 1.31–1.21 (m, 18H), 0.88 (t, *J* = 6.7 Hz, 3H);

^{13}C{^{1}H} NMR (100 MHz, CDCl$_3$) δ 82.5 (C), 74.7 (C), 47.4 (CH$_2$), 44.9 (CH$_2$), 32.1 (CH$_2$), 30.2 (CH$_2$), 29.9 (CH$_2$), 29.79 (CH$_2$), 29.76 (CH$_2$), 29.74 (CH$_2$), 29.5 (CH$_2$), 27.9 (CH$_3$), 27.8 (CH$_3$), 27.1 (CH$_3$), 24.3 (CH$_2$), 22.8 (CH$_2$), 14.3 (CH$_3$);

IR (ATR) 3374, 2965, 2926, 2855, 1467, 1240 cm^{-1};

HRMS (ESI) *m/z:* $[(M + Na)]^+$ Calcd for $C_{17}H_{36}NaO_3$ 311.2562; Found 311.2569.

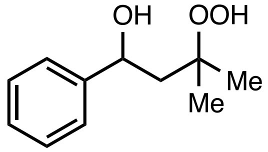

3-Hydroperoxy-3-methyl-1-phenylbutan-1-ol (S20). Alkene **S3** (0.200 g, 1.02 mmol) was subjected to the standard procedure for peroxidation at 45 ºC using $Co(pic)_2$ (0.031 g, 0.10 mmol), 1,1,3,3-tetramethyldisiloxane (395 µL, 2.24 mmol), *t*-BuOOH (204 µL, 1.0 M, 0.204 mmol), and *i*-PrOH (34 mL). Purification by automatic column chromatography (0-50% EtOAc in hexanes) afforded hydroperoxide **S20** as a white solid (0.147 g, 73%):

mp = 98 °C–101°C;

^{1}H NMR (400 MHz, $CDCl_3$) δ 7.35–7.21 (m, 5H), 4.96 (br d, *J* = 9.8, 1H), 2.32 (dd, *J* = 15.5, 9.8, 1H), 2.36 (dd, *J* = 15.5, 1.2, 1H), 1.28 (s, 3H), 1.26 (s, 3H);

$^{13}C\{^1H\}$ NMR (100 MHz, $CDCl_3$) δ 144.8 (C), 128.7 (CH), 127.8 (CH), 125.5 (CH), 81.8 (C), 71.4 (CH), 47.1 (CH_2), 26.2 (CH_3), 23.7 (CH_3);

IR (ATR) 3334, 1650, 1211, 1304, 1133, 1064 cm^{-1};

HRMS (ESI) *m*/*z* calcd for $C_{11}H_{17}O_3$ $[M + H]^+$: 197.1099, found 197.1102.

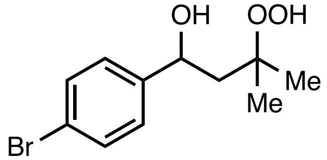

1-(4-Bromophenyl)-3-hydroperoxy-3-methylbutan-1-ol (S21). Alkene **S4** (0.480 g, 1.93 mmol) was subjected to the standard procedure for peroxidation at 45 ºC using $Co(pic)_2$ (0.060 g, 0.20 mmol), 1,1,3,3-tetramethyldisiloxane (771 µL, 4.36 mmol), *t*-BuOOH (398 µL, 1.0 M, 0.40 mmol), and *i*-PrOH (66 mL). Purification by automatic column chromatography (0-50% EtOAc in hexanes) afforded hydroperoxide **S21** as a white solid (0.338 g, 62%):

mp = 64 °C–66 °C;

^{1}H NMR (400 MHz, $CDCl_3$) δ 7.52–7.49 (m, 2H), 7.28–7.23 (m, 2H), 5.02 (br d, *J* = 9.8, 1H), 2.35 (dd, *J* = 15.4, 9.9, 1H), 1.63 (dd, *J* = 15.4, 1.4, 1H), 1.37 (s, 3H), 1.35 (s, 3H);

$^{13}C\{^1H\}$ NMR (100 MHz, $CDCl_3$) δ 143.9 (C), 131.9 (CH), 127.2 (CH), 121.5 (C), 81.9 (C), 70.8 (CH), 47.1 (CH_2), 26.1 (CH_3), 23.6 (CH_3);

IR (ATR) 3333, 3197, 1614, 1450, 1057, 704 cm^{-1};

HRMS (ESI) *m/z* calcd for $C_{11}H_{16}BrO_3$ $[M + H]^+$: 275.0205, found 275.0210.

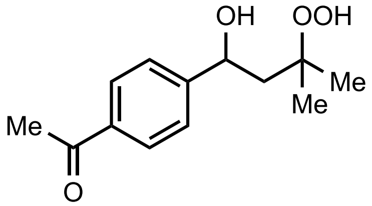

1-(4-(3-Hydroperoxy-1-hydroxy-3-methylbutyl)phenyl)ethan-1-one (S22). Alkene **S2** (0.408 g, 2.04 mmol) was subjected to the standard procedure for peroxidation at 45 ºC using $Co(pic)_2$ (0.061 g, 0.20 mmol), 1,1,3,3-tetramethyldisiloxane (770 µL, 4.49 mmol), *t*-BuOOH (408 µL, 1.0 M, 0.41 mmol), and *i*-PrOH (70 mL). Purification by automatic column chromatography (0-50% EtOAc in hexanes) afforded hydroperoxide **S22** as a clear oil (0.345 g, 72%):

^1H NMR (400 MHz, $CDCl_3$) δ 7.87 (d, J = 8.3, 2H), 7.38 (d, J = 8.3, 2H), 5.02 (br d, J = 9.7, 1H), 2.53 (s, 3H), 2.26 (dd, J = 15.2, 9.8, 1H), 1.57 (dd, J = 15.3, 1.3, 1H), 1.28 (s, 6H);

$^{13}C\{^1H\}$ NMR (100 MHz, $CDCl_3$) δ 198.1 (C), 150.1 (C), 136.5 (C), 128.8 (CH), 125.6 (CH), 82.1 (C), 70.9 (CH), 47.1 (CH_2), 26.6 (CH_3), 26.1 (CH_3), 23.6 (CH_3);

IR (ATR) 3511, 3390, 2810, 1282, 1126, 634 cm^{-1};

HRMS (ESI) *m/z* calcd for $C_{13}H_{19}O_4$ $[M + H]^+$: 239.1205, found 239.1211.

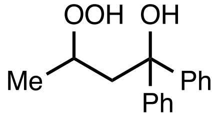

3-Hydroperoxy-1,1-diphenylbutan-1-ol (S23). 1,1-Diphenylbut-3-en-1-ol was prepared according to a reported procedure.[13] 1,1-Diphenylbut-3-en-1-ol (0.791 g, 3.53 mmol) was subjected to the standard procedure for peroxidation at 35 ºC using $Co(pic)_2$ (0.107 g, 0.353 mmol), 1,1,3,3-tetramethyldisiloxane (1.37 mL, 7.76 mmol), *t*-BuOOH (0.705 mL, 1.0 M, 0.71 mmol), and *i*-PrOH (18 mL). Purification by automatic column chromatography (0-50% EtOAc in hexanes) afforded hydroperoxide **S23** as a white solid (0.397 g, 44%):

mp = 104 ºC–106 ºC;

^{1}H NMR (400 MHz, $CDCl_3$) δ 8.36 (br s, 1H), 7.50–7.18 (m, 10H), 4.18–4.08 (m, 1H), 3.56 (br s, 1H), 2.63 (dd, *J* = 15.2, 9.1 Hz, 1H), 2.51 (dd, *J* = 15.2 Hz, 2.9 Hz, 1H), 1.27 (d, *J* = 6.3 Hz, 3H);

^{13}C{^{1}H} NMR (100 MHz, $CDCl_3$) δ 147.4 (C), 145.7 (C), 128.5 (CH), 128.4 (CH), 127.3 (CH), 127.1 (CH), 126.2 (CH), 125.9 (CH), 79.8 (CH), 78.2 (C), 45.9 (CH_2), 20.0 (CH_3);

IR (ATR) 3415, 3248, 1499, 1413, 1160, 891 cm^{-1};

HRMS (ESI) *m/z:* $[(M + Na)]^+$ Calcd for $C_{16}H_{18}NaO_3$ 281.1148; Found 281.1121.

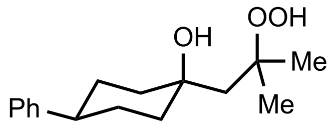

(1S,4S)-1-(2-Hydroperoxy-2-methylpropyl)-4-phenylcyclohexan-1-ol (S24). Alkene **S5** (0.332 g, 1.44 mmol) was subjected to the standard procedure for peroxidation at 45 ºC using $Co(pic)_2$ (0.044 g, 0.14 mmol), 1,1,3,3-tetramethyldisiloxane (0.560 mL, 3.17 mmol), *t*-BuOOH (288 µL, 1.0 M, 0.29 mmol), and *i*-PrOH (48 mL). Purification by automatic column chromatography (0-30% EtOAc in hexanes) afforded hydroperoxide **S24** as a clear oil (0.127 g, 33%):

^{1}H NMR (400 MHz, $CDCl_3$) δ 7.37–7.20 (m, 5H), 2.56–2.46 (m, 1H), 2.15–2.07 (m, 2H), 1.89 (s, 2H), 1.83–1.77 (m, 4H), 1.61–1.55 (m, 5H), 1.39 (s, 6H);

^{13}C{^{1}H} NMR (100 MHz, $CDCl_3$) δ 146.7 (C), 128.4 (CH), 126.8 (CH), 126.2 (CH), 82.6 (C), 72.2 (CH), 50.3 (CH), 43.5 (CH_2), 38.3 (CH_2), 29.1 (CH_2), 27.4 (CH_3);

IR (ATR) 3359, 2190, 1223, 1045, 976, 504 cm^{-1};

HRMS (ESI) *m*/*z* calcd for $[M + Na]^+$: 287.16854, found 287.16857.

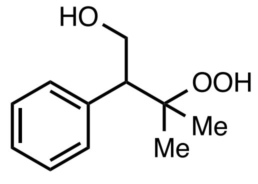

3-Hydroperoxy-3-methyl-2-phenylbutan-1-ol (S25). 3-Methyl-2-phenylbut-3-en-1-ol was prepared according to a reported procedure.[14] 3-Methyl-2-phenylbut-3-en-1-ol (0.388 g, 2.39 mmol) was subjected to the standard procedure for peroxidation at 45 ºC using $Co(pic)_2$ (0.074 g, 0.24 mmol), 1,1,3,3-tetramethyldisiloxane (952 µL, 5.39 mmol), *t*-BuOOH (480 µL, 1.0 M, 0.48 mmol), and *i*-PrOH (80 mL). Hydroperoxide **S25** was isolated as a clear oil (0.297 g, 63%) and used without further purification:

^{1}H NMR (400 MHz, $CDCl_3$) δ 7.23–7.21 (m, 5H), 4.18 (dd, *J* = 10.9, 7.6, 1H), 3.85 (dd, *J* = 10.9, 5.0, 1H), 3.26 (dd, *J* = 7.6, 5.1, 1H), 1.21 (s, 3H), 1.07 (s, 3H);

$^{13}C\{^1H\}$ NMR (100 MHz, $CDCl_3$) δ 139.4 (C), 129.5 (CH), 128.4 (CH), 127.1 (CH), 84.9 (C), 63.6 (CH_2), 53.9 (CH), 24.5 (CH_3), 20.8 (CH_3);

IR (ATR) 3514, 3254, 1448, 1383, 1105, 1055 cm^{-1};

HRMS (ESI) *m/z* calcd for $C_{11}H_{17}O_3$ $[M + H]^+$: 197.2460, found 197.2462.

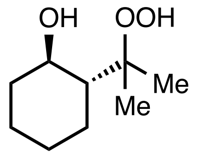

(1R,2R)-2-(2-Hydroperoxypropan-2-yl)cyclohexan-1-ol (S26). (1*R*,2*S*)-2-(Prop-1-en-2-yl)cyclohexan-1-ol was prepared according to a reported procedure.[15] (1*R*,2*S*)-2-(Prop-1-en-2-yl)cyclohexan-1-ol (0.509 g, 3.60 mmol) was subjected to the standard procedure for peroxidation at 45 ºC using Co(pic)$_2$ (0.109 g, 0.360 mmol), 1,1,3,3-tetramethyldisiloxane (1300 µL, 7.9 mmol), *t*-BuOOH (720 µL, 1.0 M, 0.72 mmol), and *i*-PrOH (120 mL). Purification by automatic column chromatography (0-40% EtOAc in hexanes) afforded hydroperoxide **S26** as a clear oil (0.403 g, 64%):

^{1}H NMR (400 MHz, $CDCl_3$) δ 3.51–3.43 (m, 1H), 1.68–1.61 (m, 1H), 1.56–1.48 (m, 4H), 1.15 (s, 3H), 1.11–1.07 (m, 4H), 1.01 (s, 3H);

$^{13}C\{^1H\}$ NMR (100 MHz, $CDCl_3$) δ 87.3 (C), 72.4 (CH), 48.7 (CH), 34.9 (CH_2), 31.6 (CH_2), 27.2 (CH_2), 25.9 (CH_2), 24.7 (CH_3), 24.6 (CH_3);

IR (ATR) 3498, 3112, 2714, 1601, 1111, 811 cm^{-1};

HRMS (ESI) *m/z* calcd for $C_9H_{18}O_3Na$ $[M + Na]^+$: 197.1154, found 197.1149.

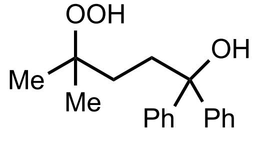

4-Hydroperoxy-4-methyl-1,1-diphenylpentan-1-ol (S27). Alkene **S13** (1.76 g, 6.97 mmol) was subjected to the standard procedure for peroxidation at 45 ºC using Co(pic)$_2$ (0.209 g, 0.689 mmol), 1,1,3,3-tetramethyldisiloxane (2.67 mL, 15.2 mmol), *t*-BuOOH (1.36 mL, 1.0 M, 1.4

mmol), and *i*-PrOH (230 mL). Hydroperoxide **S27** was isolated as a white solid (1.60 g, 80%) and used without further purification:

mp = 86 °C– 88 °C;

^{1}H NMR (400 MHz, $CDCl_3$) δ 7.45–7.41 (m, 4H), 7.37–7.32 (m, 4H), 7.29–7.24 (m, 2H), 2.44–2.39 (m, 2H), 1.62–1.57 (m, 2H), 1.21 (s, 6H);

$^{13}C\{^1H\}$ NMR (100 MHz, $CDCl_3$) δ 146.7 (C), 128.3 (CH), 127.1 (CH), 126.1 (CH), 82.4 (C), 78.9 (C), 35.1 (CH_2), 31.8 (CH_2), 24.2 (CH_3);

IR (ATR) 3491, 3209, 2763, 1630, 1008, 894 cm^{-1};

HRMS (ESI) *m*/*z* calcd for $C_{18}H_{22}O_3Na$ $[M + Na]^+$: 310.1445, found 310.1499.

C. Synthesis of Endoperoxides

Standard procedure A for cyclization: To a cooled (−78 °C) solution of the corresponding hydroperoxide (1.0 equiv) and tosyl chloride (1.1 equiv) in THF (0.1 M) was added *t*-BuOK (2.5 equiv). After 30 min, saturated aqueous NH_4Cl (10 mL/mmol of hydroperoxide) was added. The solution was warmed to 22 °C and allowed to stir for an additional 5 min. The layers were then separated, and the aqueous layer was extracted with CH_2Cl_2 (3 × 10 mL/mmol of hydroperoxide). The combined organic layers were dried over Na_2SO_4, filtered, and concentrated in vacuo. Purification by automated column chromatography afforded the endoperoxide.

Standard procedure B for cyclization: To a cooled (−78 °C) solution of the corresponding hydroperoxide (1.0 equiv) and 3,5-bis(trifluoromethyl)benzenesulfonyl chloride (1.1 equiv) in THF (0.1 M) was added *t*-BuOK (2.5 equiv). After 5 min, saturated aqueous NH_4Cl (10 mL/mmol of hydroperoxide) was added. The solution was warmed to 22 °C and allowed to stir for an additional 5 min. The layers were then separated, and the aqueous layer was extracted with CH_2Cl_2 (3 × 10 mL/mmol of hydroperoxide) The combined organic layers were dried over Na_2SO_4, filtered, and concentrated in vacuo. Purification by automated column chromatography afforded the endoperoxide.

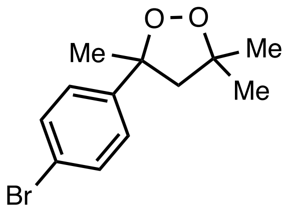

3-(4-Bromophenyl)-3,5,5-trimethyl-1,2-dioxolane (14). Hydroperoxide **13** (0.038 g, 0.13 mmol) was subjected to standard procedure A for cyclization using tosyl chloride (0.028 g, 0.14 mmol) and *t*-BuOK (0.037 g, 0.33 mmol) in THF (1 mL). Purification by automatic column chromatography (0-15% EtOAc in hexanes) afforded the endoperoxide **14** as a clear oil (0.030 g, 85%):

^{1}H NMR (400 MHz, $CDCl_3$) δ 7.46 (d, J = 8.6 Hz, 2H), 7.30 (d, J = 8.6 Hz, 2H), 2.70 (d, J = 11.9 Hz, 1H), 2.555 (d, J = 11.9 Hz, 1H), 1.59 (s, 3H), 1.44 (s, 3H), 1.19 (s, 3H);

^{13}C{^{1}H} NMR (100 MHz, $CDCl_3$) δ 145.3 (C), 131.5 (CH), 126.8 (CH), 120.9 (C), 87.2 (C), 84.6 (C), 59.7 (CH_2), 27.7 (CH_3), 27.4 (CH_3), 25.9 (CH_3);

^{17}O NMR (54 MHz, $CDCl_3$) δ 296;

IR (ATR) 2977, 2931, 1485, 1381, 1009, 822 cm^{-1};

HRMS (ESI) *m/z:* $[(M + H)]^+$ Calcd for $C_{12}H_{16}BrO$ 271.0328; Found 271.0329.

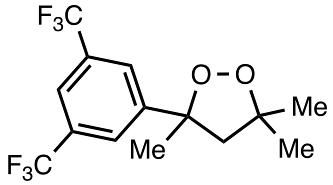

3-(3,5-Bis(trifluoromethyl)phenyl)-3,5,5-trimethyl-1,2-dioxolane (28). Hydroperoxide **S14** (0.179 g, 0.516 mmol) was subjected to standard procedure A for cyclization using tosyl chloride (0.110 g, 0.567 mmol) and *t*-BuOK (0.145 g, 1.29 mmol) in THF (5 mL). Purification by automatic column chromatography (0-15% EtOAc in hexanes) afforded the endoperoxide **28** as a clear oil (0.132 g, 78%):

^{1}H NMR (400 MHz, $CDCl_3$) δ 7.89 (s, 2H), 7.78 (s, 1H), 2.72 (d, J = 12.1 Hz, 1H), 2.65 (d, J = 12.1 Hz, 1H), 1.65 (s, 3H), 1.48 (s, 3H), 1.19 (s, 3H);

^{13}C{^{1}H} NMR (100 MHz, $CDCl_3$) δ 149.4 (C), 131.8 (C, q, $^{2}J_{C-F}$ = 33.3 Hz), 125.4 (CH, m), 123.5 (CF_3, q, $^{1}J_{C-F}$ = 272.7 Hz), 121.2 (CH, m), 86.9 (C), 85.0 (C), 59.9 (CH_2), 27.6 (CH_3), 27.5 (CH_3), 25.5 (CH_3);

$^{19}F\{^1H\}$ NMR (377 MHz, $CDCl_3$) δ –63.8;

IR (ATR) 2982, 2937, 1276, 1173, 1126, 682 cm^{-1};

HRMS (ESI) *m/z:* $[(M + Na)]^+$ Calcd for $C_{14}H_{14}F_6NaO_2$ 351.0796; Found 351.0786.

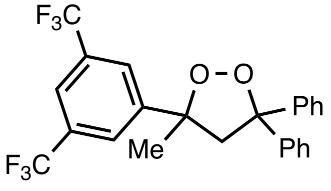

3-(3,5-Bis(trifluoromethyl)phenyl)-3-methyl-5,5-diphenyl-1,2-dioxolane (29). Hydroperoxide **S15** (0.080 g, 0.17 mmol) was subjected to standard procedure A for cyclization using tosyl chloride (0.037 g, 0.19 mmol) and *t*-BuOK (0.048 g, 0.43 mmol) in THF (2 mL). Purification by automatic column chromatography (0-15% EtOAc in hexanes) afforded the endoperoxide **29** as a white solid (0.067 g, 87%):

mp = 70 ºC–72 ºC;

^1H NMR (400 MHz, $CDCl_3$) δ 7.85 (s, 2H), 7.75 (s, 1H), 7.57–7.28 (m, 5H), 7.24–7.12 (m, 5H), 3.66 (d, J = 12.3 Hz, 1H), 3.57 (d, J = 12.3 Hz, 1H), 1.62 (s, 3H);

$^{13}C\{^1H\}$ NMR (100 MHz, $CDCl_3$) δ 148.8 (C), 143.1 (C), 141.6 (C), 131.9 (C, q, $^2J_{C-F}$ = 33.1 Hz), 128.6 (CH), 128.5 (CH), 128.0 (CH), 127.8 (CH), 126.5 (CH), 126.4 (CH), 125.5 (CH, m), 123.4 (CF_3, q, $^1J_{C-F}$ = 272.8 Hz), 121.3 (CH, m), 92.3 (C), 87.4 (C), 61.0 (CH_2), 27.2 (CH_3);

$^{19}F\{^1H\}$ NMR (377 MHz, $CDCl_3$) δ –63.9;

IR (ATR) 3063, 1449, 1378, 1275, 1126, 699 cm^{-1};

HRMS (ESI) *m/z:* $[(M + Na)]^+$ Calcd for $C_{24}H_{18}F_6NaO_2$ 475.1109; Found 475.1102.

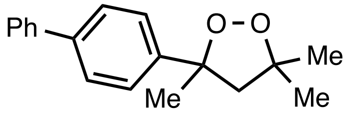

3-([1,1'-Biphenyl]-4-yl)-3,5,5-trimethyl-1,2-dioxolane (30). Hydroperoxide **S16** (0.085 g, 0.30 mmol) was subjected to standard procedure A for cyclization using tosyl chloride (0.064 g, 0.33 mmol) and *t*-BuOK (0.083 g, 0.74 mmol) in THF (3 mL). Purification by automatic column chromatography (0-15% EtOAc in hexanes) afforded the endoperoxide **30** as a white solid (0.029 g, 36%):

mp = 68 ºC–70 ºC;

^{1}H NMR (400 MHz, $CDCl_3$) δ 7.62–7.55 (m, 4H), 7.52–7.48 (m, 2H), 7.47–7.40 (m, 2H), 7.37–7.31 (m, 1H), 2.80 (d, J = 11.9 Hz, 1H), 2.59 (d, J = 11.9 Hz, 1H), 1.67 (s, 3H), 1.47 (s, 3H), 1.25 (s, 3H);

^{13}C{^{1}H} NMR (100 MHz, $CDCl_3$) δ 145.1 (C), 140.9 (C), 139.9 (C), 128.9 (CH), 127.4 (CH), 127.21 (CH), 127.17 (CH), 125.4 (CH), 87.4 (C), 84.5 (C), 59.8 (CH_2), 28.0 (CH_3), 27.3 (CH_3), 26.2 (CH_3);

IR (ATR) 3029, 2976, 2930, 1486, 765, 697 cm^{-1};

HRMS (ESI) *m/z:* $[(M + Na)]^+$ Calcd for $C_{18}H_{20}NaO_2$ 291.1361; Found 291.1361.

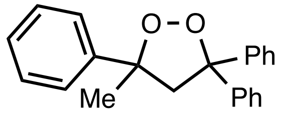

3-Methyl-3,5,5-triphenyl-1,2-dioxolane (31). Hydroperoxide **S17** (0.238 g, 0.712 mmol) was subjected to standard procedure A for cyclization using tosyl chloride (0.152 g, 0.783 mmol) and *t*-BuOK (0.200 g, 1.78 mmol) in THF (7 mL). Purification by automatic column chromatography (0-15% EtOAc in hexanes) afforded the endoperoxide **31** as a clear oil (0.152 g, 68%):

^{1}H NMR (400 MHz, $CDCl_3$) δ 7.58–7.52 (m, 2H), 7.46–7.13 (m, 13H), 3.62 (d, J = 12.4 Hz, 1H), 3.59 (d, J = 12.4 Hz, 1H), 1.58 (s, 3H);

^{13}C{^{1}H} NMR (100 MHz, $CDCl_3$) δ 146.0 (C), 143.9 (C), 142.6 (C), 128.51 (CH), 128.49 (CH), 128.3 (CH), 127.7 (CH), 127.5 (CH), 127.1 (CH), 126.9 (CH), 126.5 (CH), 125.0 (CH), 92.1 (C), 88.3 (C), 61.3 (CH_2), 27.5 (CH_3);

IR (ATR) 3059, 3025, 2979, 1447, 756, 697 cm^{-1};

HRMS (ESI) *m/z:* $[(M + Na)]^+$ Calcd for $C_{22}H_{20}NaO_2$ 339.1361; Found 339.1356.

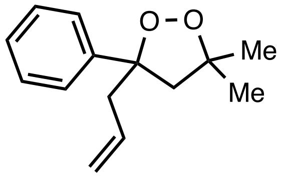

3-Allyl-5,5-dimethyl-3-phenyl-1,2-dioxolane (32). Hydroperoxide **S18** (0.070 g, 0.30 mmol) was subjected to standard procedure B for cyclization using 3,5-bis(trifluoromethyl)benzenesulfonyl chloride (0.112 g, 0.359 mmol) and *t*-BuOK (0.084 g, 0.75 mmol) in THF (3 mL). Purification

by automatic column chromatography (0-10% EtOAc in hexanes) afforded endoperoxide **32** as a light brown oil (0.444 g, 74%):

^{1}H NMR (400 MHz, $CDCl_3$) δ 7.31–7.24 (m, 4H), 7.19–7.15 (m, 1H), 5.61–5.50 (m, 1H), 4.97–4.91 (m, 2H), 2.65–2.55 (m, 4H), 1.36 (s, 3H), 1.12 (s, 3H);

^{13}C{^{1}H} NMR (100 MHz, $CDCl_3$) δ 144.3 (C), 139.3 (CH), 128.1 (CH), 126.9 (CH), 125.4 (CH), 118.5 (CH_2), 89.3 (C), 84.2 (C), 57.1 (CH_2), 45.2 (CH_2), 26.9 (CH_3), 26.2 (CH_3);

IR (ATR) 3083, 2926, 1475, 1299, 1209, 666 cm^{-1};

HRMS (ESI) *m*/*z* calcd for $C_{14}H_{19}O_2$ [M + H]$^+$: 219.1307, found 219.1310.

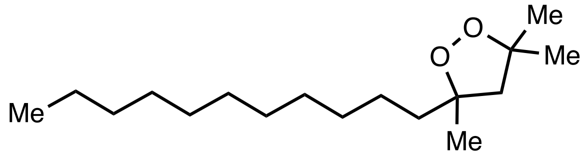

3,3,5-Trimethyl-5-undecyl-1,2-dioxolane (33). Hydroperoxide **S19** (0.410 g, 1.42 mmol) was subjected to standard procedure A for cyclization using tosyl chloride (0.304 g, 1.56 mmol) and *t*-BuOK (0.399 g, 3.56 mmol) in THF (14 mL). Purification by automatic column chromatography (0-15% EtOAc in hexanes) afforded the endoperoxide **33** as a clear oil (0.310 g, 81%):

^{1}H NMR (400 MHz, $CDCl_3$) δ 2.20 (d, J = 11.8 Hz, 1H), 2.09 (d, J = 11.8 Hz, 1H), 1.71–1.48 (m, 2H), 1.35 (s, 3H), 1.33 (s, 3H), 1.32–1.22 (m, 18H), 1.30 (s, 3H), 0.88 (t, J = 6.7 Hz, 3H);

^{13}C{^{1}H} NMR (100 MHz, $CDCl_3$) δ 86.5 (C), 83.7 (C), 57.4 (CH_2), 39.5 (CH_2), 32.1 (CH_2), 30.3 (CH_2), 29.80 (CH_2), 29.77 (CH_2), 29.74 (CH_2), 29.72 (CH_2), 29.5 (CH_2), 26.8 (CH_3), 26.6 (CH_3), 24.9 (CH_2), 24.3 (CH_3), 22.8 (CH_2), 14.3 (CH_3);

IR (ATR) 2972, 2955, 2923, 2853, 1457, 1365 cm^{-1};

HRMS (ESI) *m/z:* [(M + Na)]$^+$ Calcd for $C_{17}H_{34}NaO_2$ 293.2456; Found 293.2452.

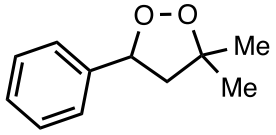

3,3-Dimethyl-5-phenyl-1,2-dioxolane (34). Hydroperoxide **S20** (0.134 g, 0.683 mmol) was subjected to standard procedure B for cyclization using 3,5-bis(trifluoromethyl)benzenesulfonyl chloride (0.235 g, 0.752 mmol) and *t*-BuOK (0.190 g, 1.69 mmol) in THF (7 mL). Purification

by automatic column chromatography (0-10% EtOAc in hexanes) afforded the endoperoxide **34** as a clear oil (0.081 g, 67%):

^{1}H NMR (400 MHz, $CDCl_3$) δ 7.35–7.21 (m, 5H), 5.23 (t, *J* = 7.6, 1H), 2.75 (dd, *J* = 12.0, 7.8, 1H), 2.36 (dd, *J* = 11.9, 7.6, 1H), 1.39 (s, 3H), 1.37 (s, 3H);

^{13}C{^{1}H} NMR (100 MHz, $CDCl_3$) δ 139.1 (C), 128.6 (CH), 128.2 (CH), 126.6 (CH), 83.9 (C), 83.3 (CH), 54.9 (CH_2), 26.5 (CH_3), 25.9 (CH_3);

IR (ATR) 3080, 1465, 1392, 1216, 1113, 712 cm^{-1};

HRMS (ESI) *m*/*z* calcd for $C_{11}H_{14}O_2Na$ $[M + Na]^+$: 201.0884, found 201.0885.

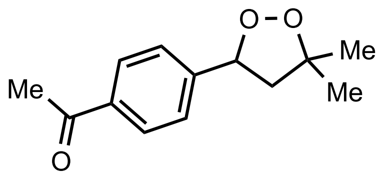

1-(4-(5,5-Dimethyl-1,2-dioxolan-3-yl)phenyl)ethan-1-one (35). Hydroperoxide **S22** (0.160 g, 0.670 mmol) was subjected to standard procedure B for cyclization using 3,5-bis(trifluoromethyl)benzenesulfonyl chloride (0.230 g, 0.736 mmol) and *t*-BuOK (0.188 g, 1.68 mmol) in THF (7 mL). Purification by automatic column chromatography (0-15% EtOAc in hexanes) afforded the endoperoxide **35** as a clear oil (0.134 g, 84%):

^{1}H NMR (400 MHz, $CDCl_3$) δ 7.96 (d, *J* = 8.4, 2H), 7.49 (d, *J* = 8.3, 2H), 5.38 (t, *J* = 7.5, 1H), 2.91 (dd, *J* = 11.9, 8.2, 1H), 2.61 (s, 3H), 2.43–2.37 (m, 1H), 1.47 (s, 3H), 1.44 (s, 3H);

^{13}C{^{1}H} NMR (100 MHz, $CDCl_3$) δ 197.7 (C), 145.4 (C), 136.7 (CH), 128.7 (CH), 126.3 (CH), 83.9 (C), 82.3 (CH), 54.9 (CH_2), 31.6 (CH_3), 26.7 (CH_3), 25.9 (CH_3);

IR (ATR) 1641, 1447, 1380, 1366, 1280, 1267 cm^{-1};

HRMS (ESI) *m*/*z* calcd for $C_{13}H_{17}O_3$ $[M + H]^+$: 221.1099, found 221.1101.

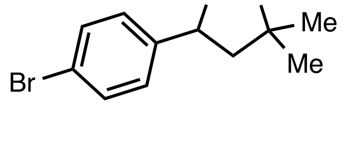

5-(4-Bromophenyl)-3,3-dimethyl-1,2-dioxolane (36). Hydroperoxide **S21** (0.140 g, 0.509 mmol) was subjected to standard procedure B for cyclization using 3,5-bis(trifluoromethyl)benzenesulfonyl chloride (0.175 g, 0.560 mmol) and *t*-BuOK (0.142 g, 1.27

mmol) in THF (5 mL). Purification by automatic column chromatography (0-10% EtOAc in hexanes) afforded the endoperoxide **36** as a white solid (0.103 g, 79%):

mp = 55 °C–57 °C;

^{1}H NMR (400 MHz, $CDCl_3$) δ 7.41−7.38 (m, 2H), 7.20-7.16 (m, 2H), 5.18 (t, *J* = 8.0, 1H), 2.75 (dd, *J* = 8.0, 11.9, 1H), 2.28 (dd, *J* = 12.0, 7.2, 1H), 1.36 (s, 3H), 1.35 (s, 3H);

$^{13}C\{^1H\}$ NMR (100 MHz, $CDCl_3$) δ 138.6 (C), 131.9 (CH), 128.1 (CH), 121.9 (C), 83.9 (C), 82.4 (CH), 54.8 (CH_2), 26.2 (CH_3), 25.9 (CH_3);

IR (ATR) 3088, 2807, 1499, 1300, 1213, 742 cm^{-1};

HRMS (ESI) *m*/*z* calcd for $C_{11}H_{13}BrO_2Na$ $[M + Na]^+$: 278.9997, found 279.0008.

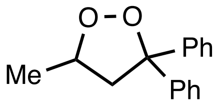

5-Methyl-3,3-diphenyl-1,2-dioxolane (37). Hydroperoxide **S23** (0.390 g, 1.51 mmol) was subjected to standard procedure B for cyclization using 3,5-bis(trifluoromethyl)benzenesulfonyl chloride (0.530 g, 1.66 mmol) and *t*-BuOK (0.424 g, 3.78 mmol) in THF (15 mL). Purification by automatic column chromatography (0-50% CH_2Cl_2 in hexanes) afforded the endoperoxide **37** as a white solid (0.051 g, 14%):

mp = 69 ºC–71 ºC;

^{1}H NMR (400 MHz, $CDCl_3$) δ), 7.44–7.39 (m, 4H), 7.36–7.29 (m, 4H), 7.28–7.22 (m, 2H), 4.65–4.55 (m, 1H), 3.43 (dd, *J* = 12.0 Hz, 6.9 Hz, 1H), 2.91 (dd, *J* = 12.0 Hz, 7.5 Hz, 1H), 1.32 (d, *J* = 6.1 Hz, 3H);

$^{13}C\{^1H\}$ NMR (100 MHz, $CDCl_3$) δ 143.4 (C), 143.3 (C), 128.5 (CH), 127.7 (CH), 127.6 (CH), 126.58 (CH), 126.55 (CH), 90.7 (CH), 78.2 (C), 54.9 (CH_2), 18.7 (CH_3);

HSQC indicates that there are 2 overlapping carbon peaks at 128.5 ppm;

IR (ATR) 1493, 1403, 1350, 1184, 1000, 916 cm^{-1};

HRMS (ESI) *m/z:* $[(M + Na)]^+$ Calcd for $C_{16}H_{16}NaO_2$ 263.1042; Found 263.1043.

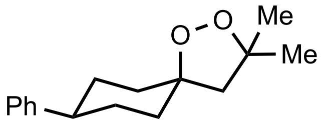

(5S,8S)-3,3-Dimethyl-8-phenyl-1,2-dioxaspiro[4.5]decane (38). Hydroperoxide **S24** (0.050 g, 0.19 mmol) was subjected to standard procedure B for cyclization using 3,5-bis(trifluoromethyl)benzenesulfonyl chloride (0.065 g, 0.21 mmol) and *t*-BuOK (0.053 g, 0.48 mmol) in THF (2 mL). Purification by automatic column chromatography (0-7% EtOAc in hexanes) afforded endoperoxide **38** as a clear oil (0.031 g, 68%):

^{1}H NMR (400 MHz, $CDCl_3$) δ 7.32–7.20 (m, 5H), 2.55–2.47 (m, 1H), 2.17 (s, 2H), 1.89–1.74 (m, 4H), 1.66–1.57 (m, 2H), 1.39 (s, 6H);

^{13}C{^{1}H} NMR (100 MHz, $CDCl_3$) δ 146.9 (C), 128.8 (CH), 127.2 (CH), 126.0 (CH), 84.2 (C), 83.1 (C), 58.5 (CH), 43.3 (CH_2), 35.7 (CH_2), 30.5 (CH_2), 26.5 (CH_3);

IR (ATR) 3030, 2812, 1444, 1210, 776, 690 cm^{-1};

HRMS (ESI) *m*/*z* calcd for $C_{16}H_{22}O_2Na$ [M + Na]$^+$: 269.1518, found 269.1513.

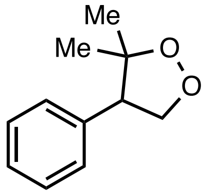

3,3-Dimethyl-4-phenyl-1,2-dioxolane (39). Hydroperoxide **S25** (0.100 g, 0.510 mmol) was subjected to standard procedure B for cyclization using 3,5-bis(trifluoromethyl)benzenesulfonyl chloride (0.175 g, 0.560 mmol) and *t*-BuOK (0.142 g, 1.26 mmol) in THF (5 mL). Purification by automatic column chromatography (0-10% EtOAc in hexanes) afforded the endoperoxide **39** as a clear oil (0.062 g, 69%):

^{1}H NMR (400 MHz, $CDCl_3$) δ 7.29– 7.19 (m, 5H), 4.51 (t, *J* = 7.8, 1H), 4.30 (t, *J* = 6.9, 1H), 3.68 (dd, *J* = 8.8, 6.8, 1H), 1.37 (s, 3H), 0.87 (s, 3H);

^{13}C{^{1}H} NMR (100 MHz, $CDCl_3$) δ 138.4 (C), 128.6 (CH), 128.3 (CH), 127.4 (CH), 85.2 (C), 75.8 (CH_2), 62.9 (CH), 26.5 (CH_3), 21.7 (CH_3);

IR (ATR) 3012, 2940, 1215, 1191, 1096, 652 cm^{-1};

HRMS (ESI) *m*/*z* calcd for $C_{11}H_{13}O$ [M + H – H_2O]$^+$: 161.0889, found 161.0888.

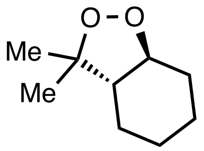

(3aS,7aS)-3,3-Dimethylhexahydro-3H-benzo[c][1,2]dioxole (40). Hydroperoxide **S26** (0.050 g, 0.29 mmol) was subjected to standard procedure B for cyclization using 3,5-bis(trifluoromethyl)benzenesulfonyl chloride (0.098 g, 0.32 mmol) and *t*-BuOK (0.081 g, 0.72 mmol) in THF (3 mL). Purification by automatic column chromatography (0-10% EtOAc in hexanes) afforded hydroperoxide **40** as a clear oil (0.029 g, 60%). The relative stereochemistry of the endoperoxide was assigned based on the coupling values of the protons on the ring (described in Section III, C of the Supporting Information):

^{1}H NMR (400 MHz, $CDCl_3$) δ 3.75 (td, *J*= 10.4, 10.4, 3.8, 1H), 2.20–2.14 (m, 1H), 1.93–1.79 (m, 4H), 1.38 (s, 3H), 1.35–1.26 (m, 4H), 1.18 (s, 3H);

^{13}C{^{1}H} NMR (100 MHz, $CDCl_3$) δ 82.86 (C), 82.83 (CH), 62.3 (CH), 29.1 (CH_2), 26.1 (CH_3), 25.0 (CH_2), 24.6 (CH_2), 23.2 (CH_2), 22.7 (CH_3);

IR (ATR) 2953, 1262, 1466, 1375, 1206, 724 cm^{-1};

HRMS (ESI) *m*/*z* calcd for $C_9H_{16}O_2Na$ [M + Na]$^+$: 179.1048, found 179.1051.

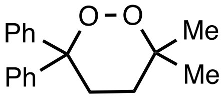

3,3-Dimethyl-6,6-diphenyl-1,2-dioxane (41). Hydroperoxide **S27** (0.185 g, 0.589 mmol) was subjected to standard procedure B for cyclization using 3,5-bis(trifluoromethyl)benzenesulfonyl chloride (0.202 g, 0.648 mmol) and *t*-BuOK (0.162 g, 1.45 mmol) in THF (6 mL). Purification by automatic column chromatography (0-10% EtOAc in hexanes) afforded endoperoxide **41** as a white solid (0.127 g, 82%):

mp = 77 °C–79° C;

^{1}H NMR (400 MHz, $CDCl_3$) ^{1}H NMR (400 MHz, $CDCl_3$) δ 7.33 (br s, 3H), 7.24 (br t, 4H), 7.15 (br t, 3H), 2.50 (br s, 2H), 1.61 (br s, 2H), 1.22 (br s, 6H);

^{13}C{^{1}H} NMR (100 MHz, $CDCl_3$) δ 128.2 (CH), 127.1 (CH), 126.4 (CH), 84.9 (C), 77.9 (C), 31.7 (CH_3), 29.9 (CH_3);

^{1}H NMR (400 MHz, DMSO-d_6, 70 °C) 7.45–7.42 (m, 4H), 7.39–7.34 (m, 4H), 7.29–7.24 (m, 2H), 2.66 (b t, *J* = 6.4, 2H), 2.62 (b t, *J* = 6.4, 2H), 1.25 (s, 6H);

$^{13}C\{^1H\}$ NMR (100 MHz, DMSO-d_6, 70 °C) δ 144.4 (C), 128.5 (CH), 127.4 (CH), 126.5 (CH), 84.9 (C), 77.9 (C), 31.7 (CH_2), 29.7 (CH_2), 25.4 (CH_3);

IR (ATR) 3173, 2937, 2853, 1347, 1061, 674 cm^{-1};

HRMS (ESI) *m/z* calcd for $C_{18}H_{21}O_2$ $[M + H]^+$: 269.1463, found 269.1463.

III. Stereochemical Correlations and Proofs

A. Synthesis of Substrates for Stereochemical Proof for O–O Bond Formation

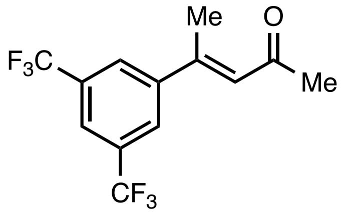

(E)-4-(3,5-Bis(trifluoromethyl)phenyl)pent-3-en-2-one (S28). To a cooled solution (0 ºC) of KOH (0.673 g, 12.0 mmol, 1.20 equiv) in a mixture of EtOH and water (4:1, 10 mL) was added 1-(3,5-bis(trifluoromethyl)phenyl)ethan-1-one (1.80 mL, 10.0 mmol, 1.00 equiv) and dimethyl (2-oxopropyl)phosphonate (1.66 mL, 12.0 mmol, 1.20 equiv). The reaction mixture was warmed to 22 ºC and stirred for 18 h. The reaction mixture was concentrated in vacuo, then partitioned between EtOAc (10 mL) and a saturated solution of ammonium chloride (10 mL). The organic layer was separated. The aqueous layer was extracted with EtOAc (3 × 10 mL). The combined organic layers were then washed with brine (1 × 10 mL), dried with sodium sulfate, filtered, then concentrated in vacuo. The crude product was purified by automated column chromatography (0-15% EtOAc in hexanes) to yield the desired ketone **S28** as a mixture of isomers (*E:Z* = 74:26) as a clear oil (1.98 g, 67% yield):

IR (ATR) 1689, 1604, 1376, 1275, 1123, 682 cm^{-1};

HRMS (ESI) *m/z:* $[(M + H)]^+$ Calcd for $C_{13}H_{11}F_6O$ 297.0709; Found 297.0723.

E isomer:

^1H NMR (400 MHz, $CDCl_3$) δ 7.88 (s, 3H), 6.54–6.52 (m, 1H), 2.55 (d, J = 1.3 Hz, 3H), 2.34 (s, 3H);

$^{13}C\{^1H\}$ NMR (100 MHz, $CDCl_3$) δ 198.5 (C), 150.2 (C), 144.8 (C), 132.2 (C, q, $^2J_{C–F}$ = 33.3 Hz), 127.0 (CH), 126.7 (CH, m), 123.3 (CF_3, q, $^1J_{C–F}$ = 273.0 Hz), 122.7 (CH, sept, $^3J_{C–F}$ = 3.7 Hz), 32.4 (CH_3), 18.4 (CH_3);

$^{19}F\{^1H\}$ NMR (377 MHz, $CDCl_3$) δ –63.94.

Characteristic peaks for *Z* isomer:

^1H NMR (400 MHz, $CDCl_3$) δ 6.36–6.32 (m, 1H), 2.20 (d, *J* = 1.5 Hz, 3H), 2.07 (s, 3H);

$^{13}C\{^1H\}$ NMR (100 MHz, $CDCl_3$) δ 197.2 (C), 149.6 (C), 143.0 (C), 131.6 (C, q, $^2J_{C–F}$ = 33.3 Hz), 127.9 (CH), 127.6 (CH, m), 123.4 (CF_3, q, $^1J_{C–F}$ = 273.0 Hz), 121.9 (CH, sept, $^3J_{C–F}$ = 3.7 Hz), 31.2 (CH_3), 27.2 (CH_3);

$^{19}F\{^1H\}$ NMR (377 MHz, $CDCl_3$) δ –63.89.

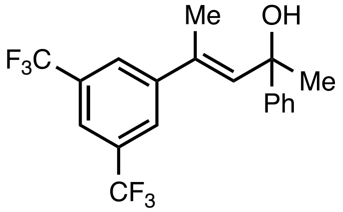

(E)-4-(3,5-Bis(trifluoromethyl)phenyl)-2-phenylpent-3-en-2-ol (S29). To a cooled solution (0 ºC) of ketone **S28** (1.98 g, 6.69 mmol, 1.0 equiv) in dry THF (10 mL) was added PhMgCl (3.0 M, 3.35 mL, 10 mmol, 1.5 equiv). The reaction mixture was warmed to 22 ºC and stirred for 18 h. A saturated solution of ammonium chloride (10 mL) was added. The organic layer was separated. The aqueous layer was extracted with EtOAc (3 × 10 mL). The combined organic layers were then washed with brine (1 × 10 mL), dried with sodium sulfate, filtered, then concentrated in vacuo. The crude product was purified by automated column chromatography (0-50% EtOAc in hexanes) to yield the desired alcohol **S29** as a mixture of isomers (*E*:*Z* = 82:12) as a clear oil (2.10 g, 84% yield):

IR (ATR) 3402, 2979, 1274, 1169, 1124, 682 cm^{-1};

HRMS (ESI) *m/z:* $[(M + Na)]^+$ Calcd for $C_{19}H_{16}F_6NaO$ 397.1003; Found 397.1009.

E isomer:

^1H NMR (400 MHz, $CDCl_3$) δ 7.79 (s, 2H), 7.77 (s, 1H), 7.56–7.50 (m, 2H), 7.42–7.34 (m, 2H), 7.32–7.29 (m, 1H), 6.36–6.32 (m, 1H), 1.998 (br s, 1H), 1.96 (d, *J* = 1.3 Hz, 3H), 1.79 (s, 3H);

$^{13}C\{^1H\}$ NMR (100 MHz, $CDCl_3$) δ 147.6 (C), 146.2 (C), 138.5 (CH), 136.9 (C), 131.7 (C, q, $^2J_{C–F}$ = 33.3 Hz), 128.6 (CH), 127.2 (CH), 126.2 (CH, m), 125.2 (CH), 123.5 (CF_3, q, $^1J_{C–F}$ = 273.0 Hz), 121.0 (CH, sept, $^3J_{C–F}$ = 3.74 Hz), 74.5 (C), 33.16 (CH_3), 17.2 (CH_3);

$^{19}F\{^1H\}$ NMR (377 MHz, $CDCl_3$) δ –63.9.

Z isomer:

^1H NMR (400 MHz, $CDCl_3$) δ 7.23–7.08 (m, 5H), 6.15–6.12 (m, 1H), 2.04 (d, *J* = 1.5 Hz, 3H), 2.001 (br s, 1H), 1.60 (s, 3H);

$^{13}C\{^1H\}$ NMR (100 MHz, $CDCl_3$) δ 147.5 (C), 144.0 (C), 136.8 (CH), 135.8 (C), 130.9 (C, q, $^2J_{C–F}$ = 33.3 Hz), 128.2 (CH, m), 127.0 (CH), 125.9 (CH), 125.1 (CH), 123.4 (CF_3, q, $^1J_{C–F}$ = 273.0 Hz), 120.4 (CH, m), 74.1 (C), 33.19 (CH), 27.7 (CH_3);

$^{19}F\{^1H\}$ NMR (377 MHz, $CDCl_3$) δ –64.0.

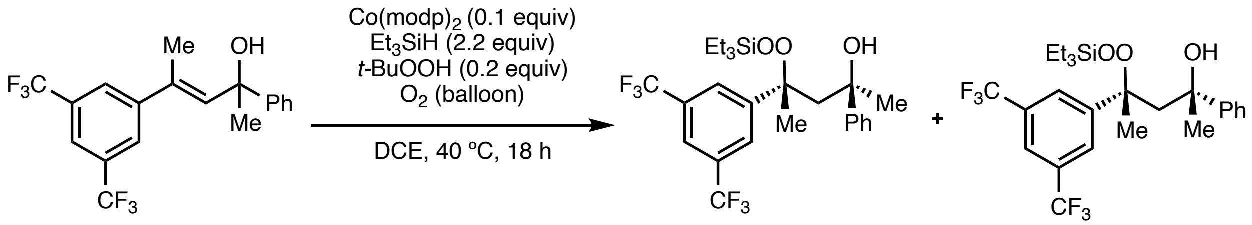

S29 ***trans*-22** ***cis*-22**

4-(3,5-Bis(trifluoromethyl)phenyl)-2-phenyl-4-((triethylsilyl)peroxy)pentan-2-ol (22). To a solution of alcohol **S29** (1.50 g, 4.00 mmol, 1.00 equiv) dissolved in DCE (10 mL) was added $Co(modp)_2$ (0.216 g, 0.400 mmol, 0.100 equiv). The reaction mixture was sonicated for 1 min. Next, Et_3SiH (1.41 mL, 8.80 mmol, 2.20 equiv) and *t*-BuOOH (1.0 M, 0.800 mL, 0.80 mmol, 0.20 equiv) was added. The reaction mixture was sparged with O_2(g) for 1 min, then stirred under O_2(g) at 40 ºC for 18 h. The reaction mixture was cooled to 22 ºC, then concentrated in vacuo. The crude product was purified by column chromatography (0-25% EtOAc in hexanes) to yield the desired silyl peroxide **22** as a clear oil (1.03 g, 49% yield, 58:42 *trans*:*cis*):

IR (ATR) 3567, 2960, 2880, 1275, 1171, 1129, 682 cm^{-1};

HRMS (ESI) *m/z:* $[(M + Na)]^+$ Calcd for $C_{25}H_{32}F_6NaO_3Si$ 545.1923; Found 545.1924.

Major (*Trans*) Diastereomer:

^{1}H NMR (400 MHz, $CDCl_3$) δ 7.71 (s, 2H), 7.69 (s, 1H), 7.38–7.14 (m, 5H), 3.15 (s, 1H), 2.49 (d, J = 15.4 Hz, 1H), 2.45 (d, J = 15.4 Hz, 1H), 1.46 (s, 3H), 1.44 (s, 3H), 0.97 (t, J = 8.0 Hz, 9H), 0.70 (q, J = 8.0 Hz, 6H);

^{13}C{^{1}H} NMR (100 MHz, $CDCl_3$) δ 148.3 (C), 147.7 (C), 131.2 (C, q, $^2J_{C–F}$ = 33.0 Hz), 128.3 (CH), 126.7 (CH), 126.2 (CH, m), 124.9 (CH), 123.6 (CF_3, q, $^1J_{C–F}$ = 272.7 Hz), 120.9 (CH, sept, $^3J_{C–F}$ = 3.7 Hz), 87.2 (C), 74.3 (C), 53.52 (CH_2), 33.7 (CH_3), 24.0 (CH_3), 6.66 (CH_2), 3.8 (CH_3);

^{19}F{^{1}H} NMR (377 MHz, $CDCl_3$) δ –63.8.

Minor (*Cis*) Diastereomer:

^{1}H NMR (400 MHz, $CDCl_3$) δ 7.45 (s, 1H), 7.36 (s, 2H), 7.03–6.82 (m, 5H), 3.75 (s, 1H), 2.74 (d, J = 15.3 Hz, 1H), 2.63 (d, J = 15.3 Hz, 1H), 1.66 (s, 3H), 1.45 (s, 3H), 1.04 (t, J = 8.0 Hz, 9H), 0.77 (q, J = 8.0 Hz, 6H);

^{13}C{^{1}H} NMR (100 MHz, $CDCl_3$) δ 146.2 (C), 145.9 (C), 130.7 (C, q, $^2J_{C–F}$ = 33.0 Hz), 127.7 (CH), 126.2 (CH), 125.8 (CH, m), 125.0 (CH), 123.5 (CF_3, q, $^1J_{C–F}$ = 272.7 Hz), 120.3 (CH, sept, $^3J_{C–F}$ = 3.7 Hz), 87.3 (C), 74.2 (C), 53.49 (CH_2), 33.8 (CH_3), 28.0 (CH_3), 6.70 (CH_2), 3.9 (CH_3);

^{19}F{^{1}H} NMR (377 MHz, $CDCl_3$) δ –63.9.

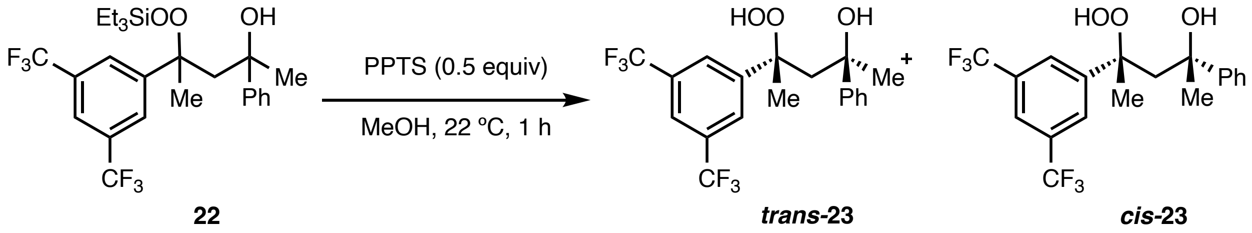

4-(3,5-Bis(trifluoromethyl)phenyl)-4-hydroperoxy-2-phenylpentan-2-ol (23). To a solution of silyl peroxide **22** (0.0455 g, 0.0871 mmol, 1.00 equiv, 95:5 *trans*:*cis*) dissolved in dry MeOH (3 mL) was added PPTS (0.0109 g, 0.0435 mmol, 0.5 equiv). The reaction mixture was stirred at 22 ºC for 1 h. The reaction mixture was concentrated in vacuo then purified by automated column chromatography (0-50% EtOAc in hexanes) to yield the desired peroxy-alcohol **23** as a clear oil (0.029 g, 80% yield, 95:5 *trans*:*cis*):

IR (ATR) 3305, 2982, 1376, 1276, 1171, 1129 cm^{-1};

HRMS (ESI) *m/z:* [(M + Na)]$^+$ Calcd for $C_{19}H_{18}F_6NaO_3$ 431.1058; Found 431.1066.

Major (*Trans*) Diastereomer:

^{1}H NMR (400 MHz, $CDCl_3$) δ 10.02 (br s, 1H), 7.83 (s, 2H), 7.75 (s, 1H), 7.50–7.23 (m, 5H), 2.65 (d, J = 15.4 Hz, 1H), 2.60 (d, J = 15.4 Hz, 1H), 1.56 (s, 3H), 1.09 (s, 3H);

^{13}C{^{1}H} NMR (100 MHz, $CDCl_3$) δ 149.3 (C), 146.7 (C), 131.7 (C, q, $^2J_{C–F}$ = 33.0 Hz), 128.7 (CH), 127.4 (CH), 125.5 (CH, m), 124.8 (CH), 123.5 (CF_3, q, $^1J_{C–F}$ = 272.8 Hz), 121.0 (CH, sept, $^3J_{C–F}$ = 3.8 Hz), 85.5 (C), 75.67 (C), 49.5 (CH_2), 32.8 (CH_3), 27.0 (CH_3);

^{19}F{^{1}H} NMR (377 MHz, $CDCl_3$) δ –63.8.

Minor (*Cis*) Diastereomer:

^{1}H NMR (400 MHz, $CDCl_3$) δ 10.15 (br s, 1H), 7.46 (s, 1H), 7.39 (s, 2H), 7.13–6.90 (m, 5H), 2.79 (d, J = 15.5 Hz, 1H), 2.57 (d, J = 15.5 Hz, 1H), 1.68 (s, 3H), 1.60 (s, 3H);

^{13}C{^{1}H} NMR (100 MHz, $CDCl_3$) δ 147.2 (C), 144.7 (C), 130.8 (C, q, $^2J_{C–F}$ = 33.0 Hz), 128.1 (CH), 127.1 (CH), 125.1 (CH, m), 125.0 (CH), 123.4 (CF_3, q, $^1J_{C–F}$ = 272.8 Hz), 120.2 (CH, sept, $^3J_{C–F}$ = 3.8 Hz), 84.7 (C), 75.71 (C), 50.5 (CH_2), 33.0 (CH_3), 29.0 (CH_3);

^{19}F{^{1}H} NMR (377 MHz, $CDCl_3$) δ –63.9.

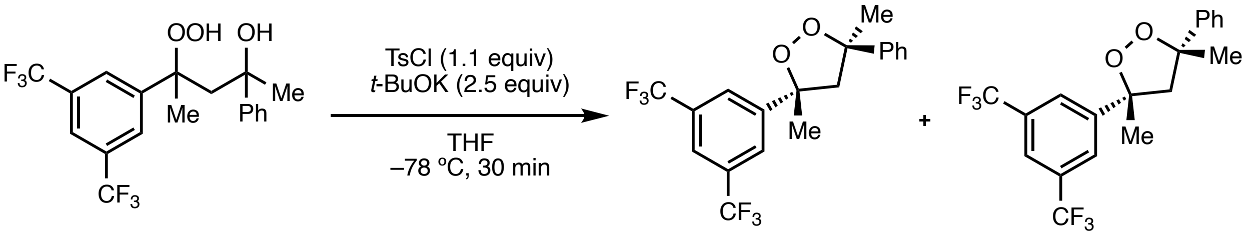

23 ***trans*-24** ***cis*-24**

3-(3,5-Bis(trifluoromethyl)phenyl)-3,5-dimethyl-5-phenyl-1,2-dioxolane (24). To a cooled (–78 ºC) solution of peroxy-alcohol **23** (0.0245 g, 0.0600 mmol, 1.00 equiv, 95:5 *trans*:*cis*) in dry THF (1 mL) were added tosyl chloride (0.0128 g, 0.0660 mmol, 1.10 equiv) then *t*-BuOK (0.0168 g, 0.150 mmol, 2.50 equiv). The reaction mixture was stirred at –78 ºC for 30 min. A saturated solution of ammonium chloride (1 mL) was added, then the mixture was warmed to 22 ºC. The organic layer was separated. The aqueous layer was extracted with EtOAc (3 × 1 mL). The combined organic layers were then washed with brine (1 × 1 mL), dried with sodium sulfate, filtered, then concentrated in vacuo. The crude mixture was purified by automated column chromatography (0-15% EtOAc in hexanes) to yield the desired endoperoxide **24** as a clear oil (0.017 g, 71% yield, 95:5 *trans*:*cis*):

IR (ATR) 2983, 2935, 1379, 1278, 1177, 1133 cm^{-1};

HRMS (ESI) *m/z:* $[(M + Na)]^+$ Calcd for $C_{19}H_{16}F_6NaO_2$ 413.0952; Found 413.0952.

Major (*Trans*) Diastereomer:

^{1}H NMR (400 MHz, $CDCl_3$) δ 7.97 (s, 2H), 7.81 (s, 1H), 7.53–7.36 (m, 4H), 7.33–7.15 (m, 1H), 3.28 (d, J = 12.1 Hz, 1H), 3.09 (d, J = 12.1 Hz, 1H), 1.45 (s, 3H), 1.43 (s, 3H);

^{13}C{^{1}H} NMR (100 MHz, $CDCl_3$) δ 149.7 (C), 145.9 (C), 132.0 (C, q, $^{2}J_{C\text{–}F}$ = 33.0 Hz), 128.7 (CH), 127.3 (CH), 125.4 (CH, m), 124.7 (CH), 123.5 (CF_3, q, $^{1}J_{C\text{–}F}$ = 272.8 Hz), 121.3 (CH, sept, $^{3}J_{C\text{–}F}$ = 3.8 Hz), 88.4 (C), 87.4 (C), 61.0 (CH_2), 27.2 (CH_3), 27.0 (CH_3);

^{19}F{^{1}H} NMR (377 MHz, $CDCl_3$) δ –63.8;

^{1}H NMR (400 MHz, C_6D_6) δ 7.94 (s, 2H), 7.70 (s, 1H), 7.45–7.39 (m, 2H), 7.25–7.18 (m, 2H), 7.11–7.06 (m, 1H), 2.60 (d, J = 12.3 Hz, 1H), 2.37 (d, J = 12.3 Hz, 1H), 1.05 (s, 3H), 0.90 (s, 3H);

^{13}C{^{1}H} NMR (100 MHz, C_6D_6) δ 150.4 (C), 146.5 (C), 132.1 (C, q, $^{2}J_{C\text{–}F}$ = 33.2 Hz), 128.7 (CH), 127.3 (CH), 125.6 (CH, m), 124.7 (CH), 124.0 (CF_3, q, $^{1}J_{C\text{–}F}$ = 272.8 Hz), 121.2 (CH, m), 88.2 (C), 87.1 (C), 60.9 (CH_2), 26.9 (CH_3), 26.1 (CH_3);

^{19}F{^{1}H} NMR (377 MHz, C_6D_6) δ –63.5.

Minor (*Cis*) Diastereomer ($CDCl_3$):

^{1}H NMR (400 MHz, $CDCl_3$) δ 7.72 (s, 2H), 7.68 (s, 1H), 7.53–7.36 (m, 4H), 7.33–7.15 (m, 1H), 3.19 (d, J = 12.2 Hz, 1H), 2.97 (d, J = 12.2 Hz, 1H), 1.78 (s, 3H), 1.77 (s, 3H);

^{13}C{^{1}H} NMR (100 MHz, $CDCl_3$) δ 148.1 (C), 143.7 (C), 131.7 (C, q, $^{2}J_{C\text{–}F}$ = 33.0 Hz), 128.5 (CH), 127.5 (CH), 125.7 (CH, m), 124.9 (CH), 123.4 (CF_3, q, $^{1}J_{C\text{–}F}$ = 272.8 Hz), 121.2 (CH, sept, $^{3}J_{C\text{–}F}$ = 3.8 Hz), 88.3 (C), 87.0 (C), 61.5 (CH_2), 27.8 (CH_3), 27.5 (CH_3);

^{19}F{^{1}H} NMR (377 MHz, $CDCl_3$) δ –63.9.

Characteristic Peaks for Minor (*Cis*) Diastereomer (C_6D_6):

^{1}H NMR (400 MHz, C_6D_6) δ 7.67 (s, 2H), 7.54 (s, 1H), 7.03–6.89 (m, 4H), 7.13–7.10 (m, 1H), 2.65 (d, J = 12.3 Hz, 1H), 2.2.24 (d, J = 12.3 Hz, 1H), 1.44 (s, 3H), 1.26 (s, 3H);

^{13}C{^{1}H} NMR (100 MHz, C_6D_6) δ 128.6 (CH), 127.4 (CH), 125.9 (CH, m), 125.0 (CH), 120.5 (CH, m), 88.1 (C), 86.6 (C), 61.2 (CH_2), 27.6 (CH_3), 26.9 (CH_3);

$^{19}F\{^1H\}$ NMR (377 MHz, C_6D_6) δ –63.7.

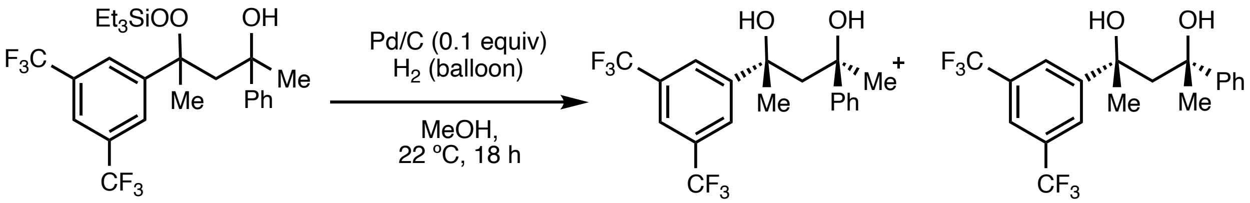

22 ***trans*-25** ***cis*-25**

2-(3,5-Bis(trifluoromethyl)phenyl)-4-phenylpentane-2,4-diol (25). To a solution of silyl peroxide **22** (0.0239 g, 0.0457 mmol, 1.00 equiv, 95:5 *trans*:*cis*) dissolved in dry MeOH (3 mL) was added Pd/C (10% w/w, 0.0049 g, 0.0046 mmol, 0.10 equiv). The reaction mixture was sparged with H_2(g) for 1 min. The reaction mixture was then stirred under H_2(g) at 22 ºC for 18 h. Then, the reaction mixture was purged with N_2(g) for 5 min and filtered through Celite with EtOAc (10 mL). The crude mixture was concentrated in vacuo to yield the desired diol **25** as a clear oil (0.0179 g, quant. yield, 95:5 *trans*:*cis* mixture):

IR (ATR) 3329, 2970, 2936, 1373, 1278, 1174, 1132 cm^{-1};

HRMS (ESI) *m/z:* $[(M + Na)]^+$ Calcd for $C_{19}H_{18}F_6NaO_2$ 415.1109; Found 415.1105.

Major (*Trans*) Diastereomer:

^1H NMR (400 MHz, $CDCl_3$) δ 7.95 (s, 2H), 7.76 (s, 1H), 7.48–7.25 (m, 5H), 4.78 (br s, 1H), 2.92 (br s, 1H), 2.45 (d, J = 15.1 Hz, 1H), 2.37 (d, J = 15.1 Hz, 1H), 1.29 (s, 3H), 1.28 (s, 3H);

$^{13}C\{^1H\}$ NMR (100 MHz, $CDCl_3$) δ 152.4 (C), 148.3 (C), 131.6 (C, q, $^2J_{C\text{–}F}$ = 33.0 Hz), 128.7 (CH), 127.3 (CH), 125.3 (CH, m), 124.4 (CH), 123.6 (CF_3, q, $^1J_{C\text{–}F}$ = 273.2 Hz), 120.7 (CH, sept, $^3J_{C\text{–}F}$ = 3.7 Hz), 76.6 (C), 75.3 (C), 53.7 (CH_2), 32.1 (CH_3), 32.0 (CH_3);

$^{19}F\{^1H\}$ NMR (377 MHz, $CDCl_3$) δ –63.7.

Characteristic Peaks for Minor (*Cis*) Diastereomer:

^1H NMR (400 MHz, $CDCl_3$) 2.65 (d, J = 14.8 Hz, 1H), 2.41 (d, J = 14.8 Hz, 1H), 1.57 (s, 3H), 1.48 (s, 3H);

$^{13}C\{^1H\}$ NMR (100 MHz, $CDCl_3$) δ 149.5 (C), 145.0 (C), 130.5 (C, q, $^2J_{C\text{–}F}$ = 33.0 Hz), 128.0 (CH), 126.5 (CH), 125.5 (CH, m), 124.4 (CH), 123.5 (CF_3, q, $^1J_{C\text{–}F}$ = 273.2 Hz), 119.9 (CH, sept, $^3J_{C\text{–}F}$ = 3.7 Hz), 76.7 (C), 75.2 (C), 53.5 (CH_2), 34.7 (CH_3), 34.1 (CH_3);

$^{19}F\{^1H\}$ NMR (377 MHz, $CDCl_3$) δ –63.9.

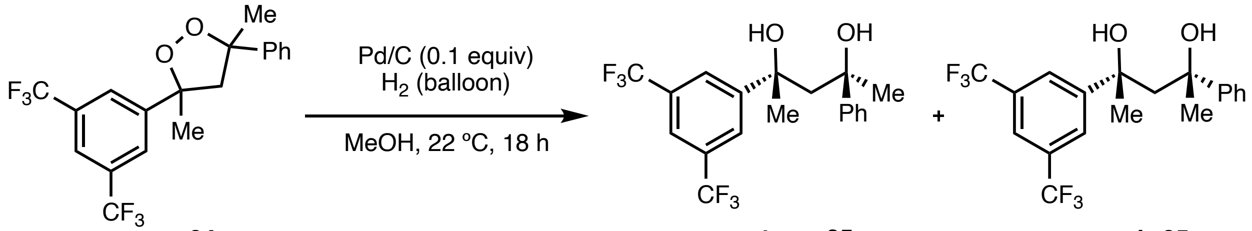

24 ***trans*-25** ***cis*-25**

2-(3,5-Bis(trifluoromethyl)phenyl)-4-phenylpentane-2,4-diol (25). To a solution of endoperoxide **24** (0.0151 g, 0.0387 mmol, 1.00 equiv, 95:5 *trans*:*cis*) dissolved in dry MeOH (3 mL) was added Pd/C (10% w/w, 0.0041 g, 0.0039 mmol, 0.1 equiv). The reaction mixture was sparged with H_2(g) for 1 min. The reaction mixture was then stirred under H_2(g) at 22 ºC for 18 h. Then, the reaction mixture was purged with N_2(g) for 5 min and filtered through Celite with EtOAc (10 mL). The crude mixture was concentrated in vacuo to yield the desired diol **25** as a clear oil (0.0152 g, quant. yield, 95:5 *trans*:*cis*):

The spectral data match those collected for the product of the hydrogenolysis of the silyl-protected hydroperoxide **22**.

B. Assignment of Relative Stereochemical Configuration for Endoperoxide 24

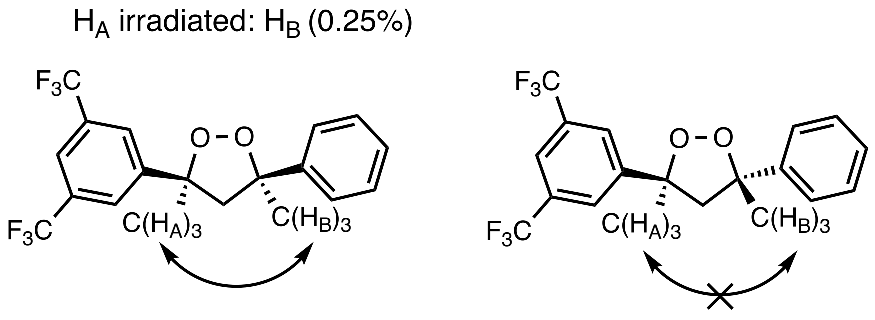

***cis*-24** ***trans*-24**

The 1,3-*cis* and 1,3-*trans* stereochemistry of the two diastereomers of **24** were determined by NOESY studies. For ***cis*-24**, the 1,3 *cis* stereochemistry assignment was supported by the NOE signal between H_A and H_B. Similarly, for ***trans*-24**, the 1,3-*trans* stereochemistry assignment was supported by the absence of the NOE signal between H_A and H_B. The NOESY data can be found on pages S85 to S89.

C. Assignment of Relative Stereochemical Configuration for Endoperoxide 40

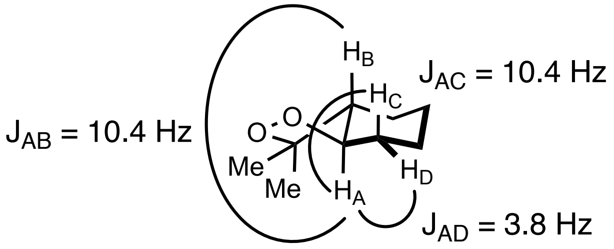

The peaks and relative stereochemistry of the protons in endoperoxide **40** were assigned in the ^{1}H NMR spectrum based on chemical shifts and coupling constants. ^{1}H-^{13}C HSQC experiments were used to identify H_A as the proton attached to the carbon atom adjacent to the endoperoxide oxygen atom. Analysis of neighboring protons relative to H_A indicated that the product is the *trans*-fused isomer.

The observed coupling constant (J_{AB} = 10.4 Hz) confirms that the relative orientation between H_A and H_B is approximately 180°. Similarly, the relative orientation between H_A and H_C can also be determined to be approximately 180° because J_{AC} = 10.4 Hz. In contrast, the smaller coupling constant observed between H_A and H_D (J_{AD} = 3.8 Hz) corresponds to a dihedral angle of approximately 60°, indicating that H_D occupies the equatorial position. These assignments collectively define the relative stereochemistry of the protons on the endoperoxide.

D. Assignment of Relative Stereochemical Configuration for Alkene S5

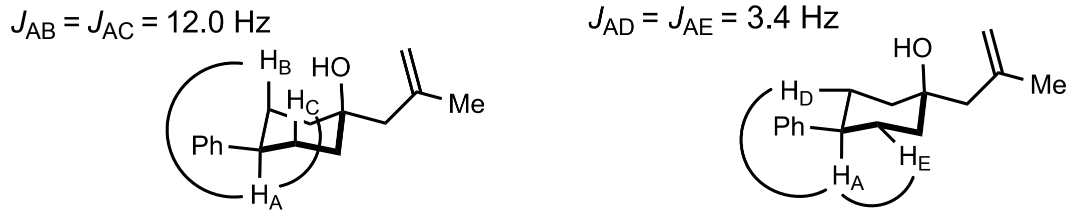

The relative stereochemistry of alkene **S5** was assigned based on coupling constants and chemical shifts in the ^{1}H NMR and ^{13}C{^{1}H} NMR spectrum, respectively. The proton H_A appears as a triplet of triplets with a large coupling constant (J = 12.0 Hz) and a smaller one (J = 3.4 Hz). The large vicinal coupling to protons H_B and H_C is consistent with H_A adopting an axial orientation.

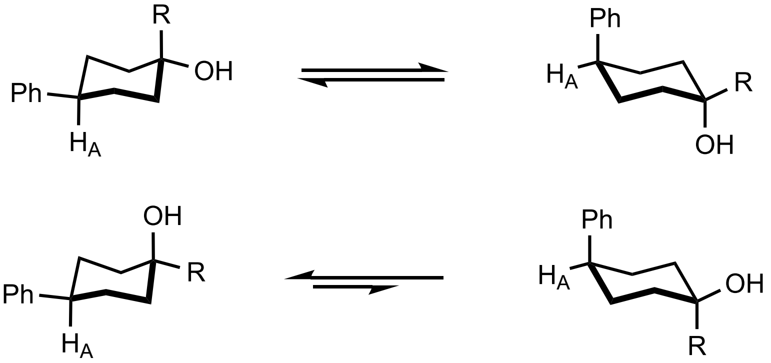

The coupling constants indicate the relative configuration at the two carbon atoms. The *cis* isomer will likely exist as two conformers, meaning that the environment experienced by H_A would be an average of the axial and equatorial positions. As a result, the *J* values will be averaged. In contrast, for the *trans* isomer, the conformer with both alkyl groups in axial orientations would be disfavored. Consequently, the diequatorial conformation would be favored, leading to the coupling constants observed. This conformational analysis is further supported by the reported stereochemistry of known compounds. Grignard additions to carbonyl compounds in similar 4-substituted cyclohexanones produce the *trans* isomer as the major product.[16] The resulting compounds also exhibit the same splitting pattern (triplet of triplets, J = 12.2, 3.4 Hz), further confirming the reported stereochemistry.

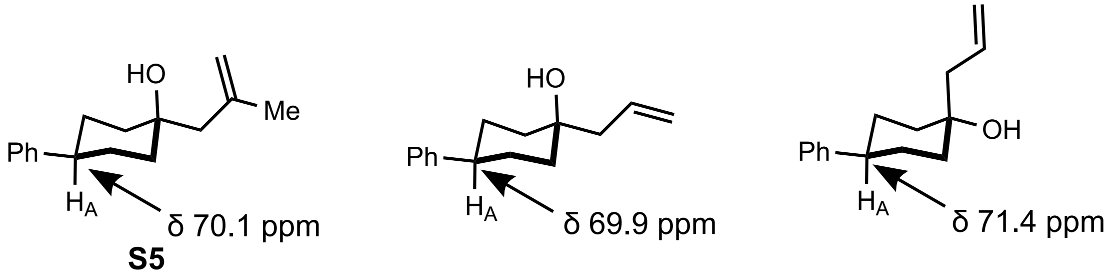

Analysis of $^{13}C\{^1H\}$ NMR spectra support this analysis. By comparison with the reported *trans* and *cis* allyl derivatives, the ^{13}C shift of the carbon atom bearing proton H_A appears at δ 69.94 or δ 71.35 ppm, respectively.[17] These similarities support the conclusion that the isolated product **S5** is the *trans* isomer, which exhibits a ^{13}C shift of δ 70.1 ppm.

IV. Structural Proofs

A. Synthesis for Structural Proof for Benzoylated Peroxide 21

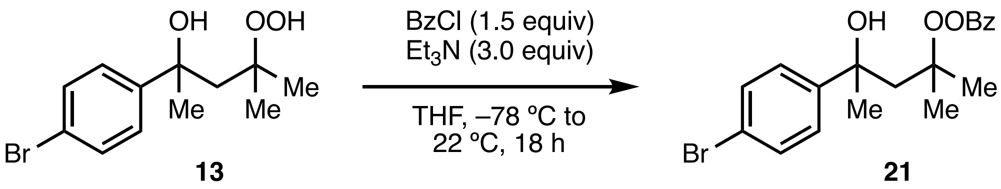

4-(4-Bromophenyl)-4-hydroxy-2-methylpentan-2-yl benzoperoxoate (21). To a cooled (–78 ºC) solution of peroxy-alcohol **13** (0.0358 g, 0.124 mmol, 1.00 equiv) in dry THF (1 mL) were added benzoyl chloride (21.6 µL, 0.186 mmol, 1.50 equiv) then Et_3N (51.8 µL, 0.371 mmol, 3.00 equiv). The reaction mixture was stirred and warmed to 22 ºC for 18 h. The reaction mixture was filtered with EtOAc (5 mL). The filtrate was concentrated in vacuo. The crude product was purified by automated column chromatography (0-50% EtOAc in hexanes) to yield the desired benzoylated peroxide **21** as a clear oil (0.0358 g, 74% yield):

^{1}H NMR (400 MHz, $CDCl_3$) δ 7.97–7.91 (m, 2H), 7.64–7.58 (m, 1H), δ 7.50–7.38 (m, 6H), 4.26 (s, 1H), 2.39 (d, *J* = 15.6 Hz, 1H), 2.30 (d, *J* = 15.6 Hz, 1H), 1.51 (s, 3H), 1.41 (s, 3H), 1.05 (s, 3H);

^{13}C{^{1}H} NMR (100 MHz, $CDCl_3$) δ 165.3 (C), 148.4 (C), 134.0 (CH), 131.3 (CH), 129.4 (CH), 128.9 (CH), 127.0 (C), 126.9 (CH), 120.2 (C), 88.0 (C), 73.7 (C), 50.1 (CH_2), 34.4 (CH_3), 27.9 (CH_3), 24.7 (CH_3);

^{1}H NMR (400 MHz, C_6D_6) δ 8.85–7.79 (m, 2H), 7.30 (d, *J* = 8.7 Hz, 2H), 7.21 (d, *J* = 8.7 Hz, 2H), 7.06–7.00 (m, 1H), 6.96–6.88 (m, 2H), 4.38 (s, 1H), 2.14 (d, *J* = 15.6 Hz, 1H), 1.93 (d, *J* = 15.6 Hz, 1H), 1.40 (s, 3H), 1.17 (s, 3H), 0.95 (s, 3H);

^{13}C{^{1}H} NMR (100 MHz, C_6D_6) δ 165.4 (C), 149.2 (C), 133.6 (CH), 131.4 (C), 129.4 (CH), 128.8 (CH), 127.4 (CH), 127.3 (CH), 120.4 (C), 87.8 (C), 73.5 (C), 50.2 (CH_2), 34.6 (CH_3), 27.7 (CH_3), 24.5 (CH_3);

IR (ATR) 3497, 2978, 1745, 1233, 1051, 1023, 703 cm^{-1};

HRMS (ESI) *m/z:* $[(M + Na)]^+$ Calcd for $C_{19}H_{21}BrNaO_4$ 415.0521; Found 415.0522.

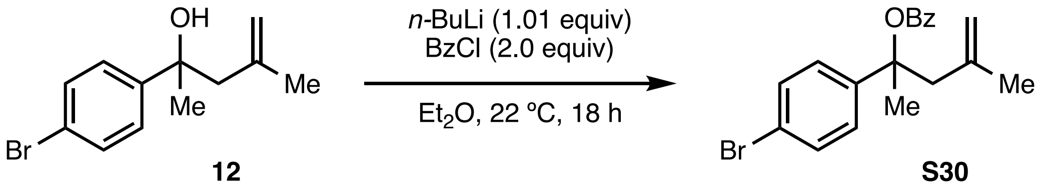

2-(4-Bromophenyl)-4-methylpent-4-en-2-yl benzoate (S30). To a solution of alcohol **12** (0.128 g, 0.502 mmol, 1.00 equiv) dissolved in dry Et_2O (5 mL) were added *n*-BuLi (1.18 M, 430 µL, 0.508 mmol, 1.01 equiv) and benzoyl chloride (117 µL, 1.01 mmol, 2.00 equiv). The reaction mixture was stirred at 22 ºC for 18 h. A saturated solution of ammonium chloride (5 mL) was added, and the organic layer was separated. The aqueous layer was extracted with EtOAc (3 × 5 mL). The combined organic layers were then washed with brine (1 × 5 mL), dried with sodium sulfate, filtered, then concentrated in vacuo. The crude product was purified by automated

column chromatography (0-15% EtOAc in hexanes) to yield the desired protected alcohol **S30** as a clear oil (0.0838 g, 46% yield):

^{1}H NMR (400 MHz, $CDCl_3$) δ 8.11–8.02 (m, 2H), 7.63–7.54 (m, 1H), 7.52–7.42 (m, 4H), 7.32–7.25 (m, 2H), 4.91 (s, 1H), 4.74 (s, 1H), 2.89 (d, J = 13.8 Hz, 1H), 2.78 (d, J = 13.8 Hz 1H), 2.00 (s, 3H), 1.65 (s, 3H);

^{13}C{^{1}H} NMR (100 MHz, $CDCl_3$) δ 165.2 (C), 144.3 (C), 140.8 (C), 133.0 (CH), 131.5 (CH), 131.4 (C), 129.7 (CH), 128.6 (CH), 126.7 (CH), 121.2 (C), 116.7 (CH_2), 83.9 (C), 51.0 (CH_2), 24.6 (CH_3), 24.4 (CH_3);

IR (ATR) 3073, 2981, 2943, 1715, 1271, 1075, 1068, 708 cm^{-1};

HRMS (ESI) *m/z:* [(M + Na)]$^{+}$ Calcd for $C_{19}H_{19}BrNaO_2$ 381.0466; Found 381.0469.

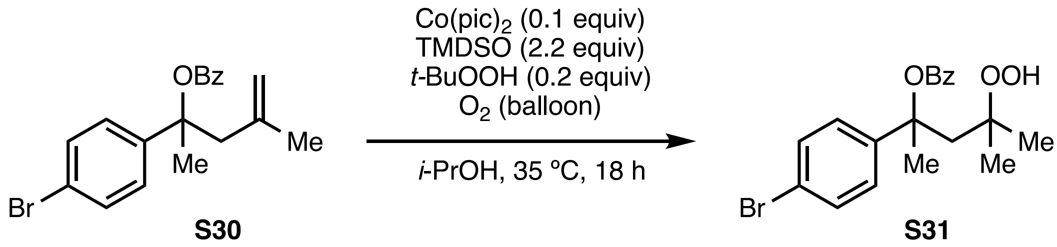

2-(4-Bromophenyl)-4-hydroperoxy-4-methylpentan-2-yl benzoate (S31). To a solution of protected alcohol **S30** (0.0251 g, 0.070 mmol, 1.00 equiv) dissolved in *i*-PrOH (3 mL) was added Co(pic)$_2$ (0.0021 g, 0.0070 mmol, 0.10 equiv). The reaction mixture was sonicated for 1 min. Next, TMDSO (27.2 μL, 0.154 mmol, 2.20 equiv) and *t*-BuOOH (1.0 M, 14.0 μL, 0.014 mmol, 0.20 equiv) was added. The reaction mixture was sparged with O_2(g) for 1 min, then stirred under O_2(g) at 35 ºC for 18 h. The reaction mixture was cooled to 22 ºC, then concentrated in vacuo. The crude product was purified by automated column chromatography (0-50% EtOAc in hexanes) to yield the desired peroxide **S31** as a clear oil (0.0086 g, 31% yield):

^{1}H NMR (400 MHz, C_6D_6) δ 8.15–8.09 (m, 2H), 7.48 (s, 1H), 7.23 (d, J = 8.8 Hz, 2H), 7.14–7.00 (m, 3H), 6.98 (d, J = 8.8 Hz, 2H), 2.67 (d, J = 15.7 Hz, 1H), 2.22 (d, J = 15.7 Hz, 1H), 1.85 (s, 3H), 1.09 (s, 3H), 1.05 (s, 3H);

^{13}C{^{1}H} NMR (100 MHz, C_6D_6) δ 166.5 (C), 146.2 (C), 133.0 (CH), 132.2 (C), 131.7 (CH), 129.9 (CH), 128.7 (CH), 126.6 (CH), 121.0 (C), 84.1 (C), 82.2 (C), 45.2 (CH_2), 28.4 (CH_3), 26.9 (CH_3), 25.5 (CH_3);

IR (ATR) 3404, 2981, 1717, 1700, 1283, 1026, 711 cm^{-1};

HRMS (ESI) *m/z:* [(M + Na)]$^{+}$ Calcd for $C_{19}H_{21}BrNaO_4$ 415.0521; Found 415.0525.

B. Structural Proof for Benzoylated Peroxide 21

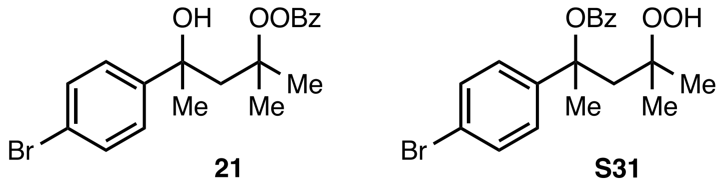

21 **S31**

To confirm the structure of benzoylated peroxide **21**, benzoylated alcohol **S31** was synthesized. The NMR spectra of **21** were compared to **S31**. In deuterated benzene, the chemical shifts of the peaks for both the ^{1}H and ^{13}C{^{1}H} NMR spectra of **21** are different than those for **S31**. Additionally, a peroxide peak is absent in the ^{1}H NMR spectrum of **21** but is present in the ^{1}H NMR spectrum of **S31** as indicated by the downfield singlet at δ 7.48 ppm. Conversely, an alcohol peak is present in the ^{1}H NMR spectrum of **21** as indicated by the upfield singlet at δ 4.38 ppm which is absent in the ^{1}H NMR spectrum of **S31**. Lastly, when comparing the infrared spectra of **21** and **S31**, there is a carbonyl band at 1745 cm^{-1} for **21** while there are two carbonyl bands at 1717 cm^{-1} and 1700 cm^{-1} for **S31**. The wavenumber of the carbonyl band of **21** is closer to reported values for peroxyesters (1760–1780 cm^{-1}). In contrast, the wavenumbers of the carbonyl bands of **S31** are closer to reported values for esters (1720–1740 cm^{-1}).[18] These data collectively support that, for compound **21**, the peroxide group was benzoylated regioselectively.

V. Synthesis for ^{17}O-labeled Substrates

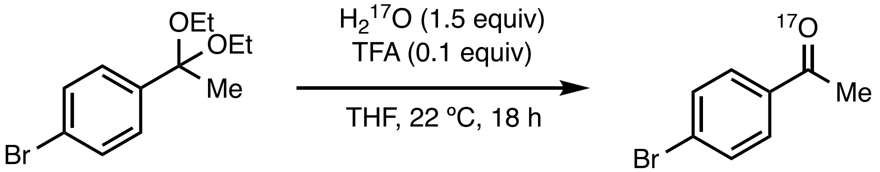

S32 **S33**

1-(4-Bromophenyl)ethan-1-one-^{17}O (S33). 1-Bromo-4-(1,1-diethoxyethyl)benzene **(S32)** was prepared according to a reported procedure.[19] To a solution of 1-bromo-4-(1,1-diethoxyethyl)benzene (0.562 g, 2.06 mmol, 1.00 equiv) dissolved in dry THF (4 mL) were added $H_2{}^{17}O$ (2% enriched, 100 μL, 5.55 mmol, 2.70 equiv) then TFA (15.8 μL, 0.206 mmol, 0.10 equiv). The reaction mixture was stirred at 22 °C for 18 h. Et_3N was then added (28.7 μL, 0.206 mmol, 0.1 equiv). The reaction mixture was concentrated in vacuo then purified by automated column chromatography (0-15% EtOAc in hexanes) to yield the desired ketone **S33** as a white solid (0.367 g, 89% yield). The spectroscopic data are consistent with the data reported for the non-isotope labeled ketone **S33**:[20]

mp = 49 °C–51 °C;

^{1}H NMR (400 MHz, $CDCl_3$) δ 7.80 (d, J = 8.6 Hz, 2H), 7.59 (d, J = 8.6 Hz, 2H), 2.57 (s, 3H);

$^{13}C\{^1H\}$ NMR (100 MHz, $CDCl_3$) δ 196.9 (C), 135.9 (C), 131.9 (CH), 129.8 (CH), 128.3 (C), 26.5 (CH_3);

^{17}O NMR (54 MHz, $CDCl_3$) δ 544;

IR (ATR) 3010, 2970, 1739, 1672, 1588, 1365, 824 cm^{-1};

HRMS (ESI) *m/z:* $[(M + H)]^+$ Calcd for $C_8H_8Br^{16}O$ 198.9753; Found 198.9763.

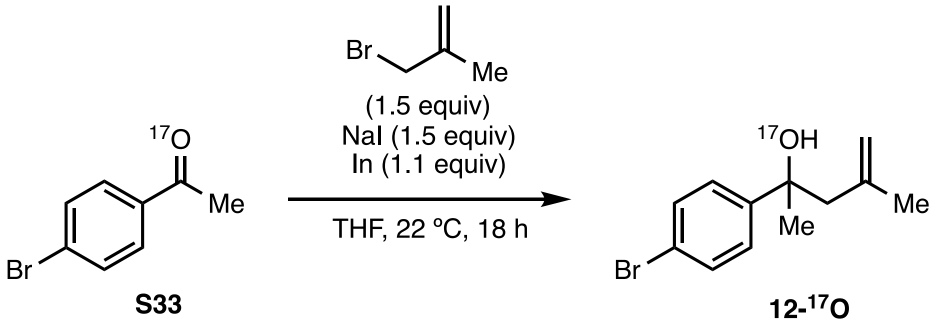

2-(4-Bromophenyl)-4-methylpent-4-en-2-ol-^{17}O (12-^{17}O). The isotope labeled product was prepared according to the procedure used for the non-isotope labeled product using ketone **S33** (0.312 g, 1.57 mmol, 1.00 equiv), sodium iodide (352 mg, 2.35 mmol, 1.50 equiv), 3-bromo-2-methylprop-1-ene (237 μL, 2.35 mmol, 1.50 equiv), indium powder (198 mg, 1.72 mmol, 1.10 equiv), and THF (8 mL). The crude product was purified by automated column chromatography (0-50% EtOAc in hexanes) to yield the desired alcohol **12-^{17}O** as a clear oil (0.356 g, 89% yield). The ^1H, $^{13}C\{^1H\}$, and IR spectra of the isotope labeled material matched those of the non-isotope labeled material. The HRMS also matched, which is consistent with the fact that the product is ≤ 2% labeled:

^{17}O NMR (54 MHz, $CDCl_3$) δ 45.3.

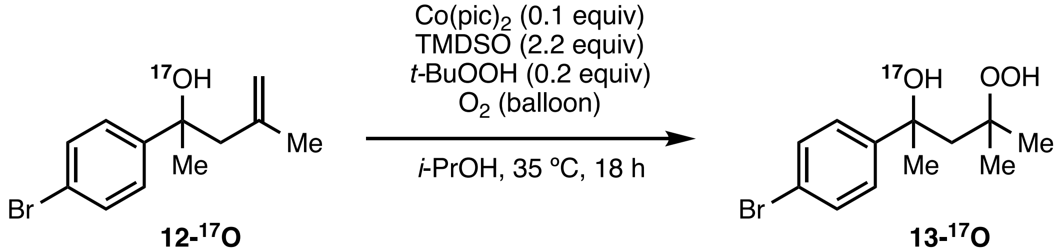

2-(4-Bromophenyl)-4-hydroperoxy-4-methylpentan-2-ol-^{17}O (13-^{17}O). The isotope labeled product was prepared according to the procedure used for the non-isotope labeled product using alcohol **12-^{17}O** (0.206 g, 0.807 mmol, 1.00 equiv), $Co(pic)_2$ (0.0245 g, 0.0807 mmol, 0.10 equiv), TMDSO (314 μL, 1.77 mmol, 2.20 equiv), *t*-BuOOH (1.0 M, 161 μL, 0.16 mmol, 0.20

equiv), and *i*-PrOH (8 mL). The crude product was purified by automated column chromatography (0-50% EtOAc in hexanes) to yield the desired peroxide **13-^{17}O** as a clear oil (0.226 g, 97% yield). The ^{1}H, ^{13}C{^{1}H}, and IR spectra of the isotope labeled material matched those of the non-isotope labeled material. The HRMS also matched, which is consistent with the fact that the product is ≤ 2% labeled:

^{17}O NMR (54 MHz, $CDCl_3$) δ 40.1.

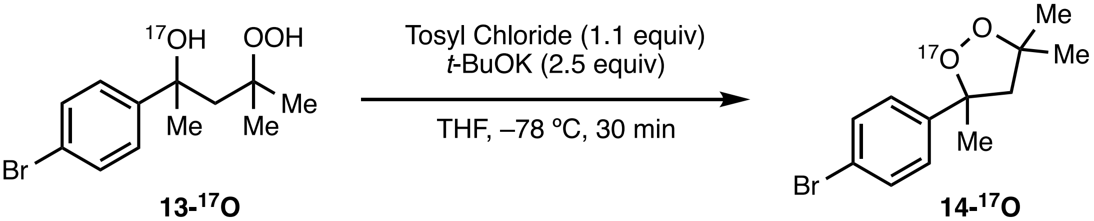

13-^{17}O **14-^{17}O**

3-(4-Bromophenyl)-3,5,5-trimethyl-1,2-dioxolane-2-^{17}O (14-^{17}O). The isotope labeled compound was prepared according to the procedure used for the non-isotope labeled material using peroxy-alcohol **13-^{17}O** (0.180 g, 0.621 mmol, 1.00 equiv), tosyl chloride (0.133 g, 0.683 mmol, 1.10 equiv), *t*-BuOK (0.209 g, 1.86 mmol, 3.00 equiv), and THF (6 mL). The crude product was purified by automated column chromatography (0-15% EtOAc in hexanes) to yield the desired endoperoxide **14-^{17}O** as a clear oil (0.141 g, 84% yield). The ^{1}H, ^{13}C{^{1}H}, and IR spectra of the isotope labeled material matched those of the non-isotope labeled material. The HRMS also matched, which is consistent with the fact that the product is ≤ 2% labeled:

^{17}O NMR (54 MHz, $CDCl_3$) δ 296.

VI. Radical Quenching Experiments

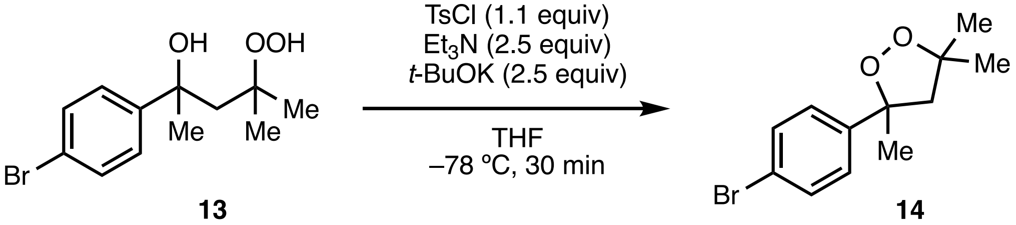

13 **14**

To a cooled (–78 ºC) solution of peroxide **13** (0.0467 g, 0.161 mmol, 1.00 equiv) in dry THF (1.6 mL) were added tosyl chloride (0.0346 g, 0.178 mmol, 1.10 equiv), Et_3N (56.3 µL, 0.404 mmol, 2.50 equiv) then *t*-BuOK (0.0453 g, 0.404 mmol, 2.50 equiv). The reaction mixture was stirred at –78 ºC for 30 min. A saturated solution of ammonium chloride (2 mL) was added, then the mixture was warmed to 22 ºC. The organic layer was separated. The aqueous layer was extracted with EtOAc (3 × 2 mL). The combined organic layers were then washed with brine (1 × 2 mL), dried with sodium sulfate, filtered, then concentrated in vacuo. The crude NMR spectrum indicated that the desired product **14** was formed (^{1}H NMR yield: 90%).

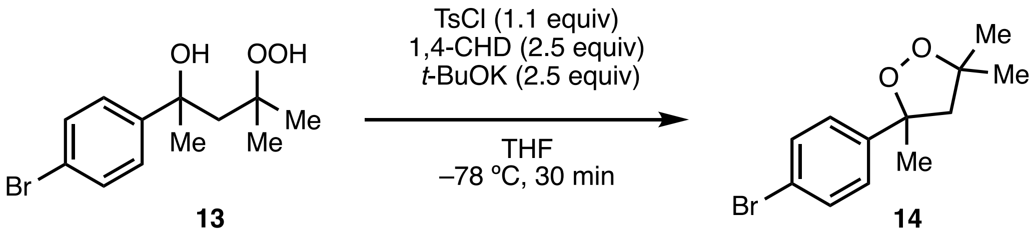

To a cooled (–78 ºC) solution of peroxide **13** (0.0496 g, 0.172 mmol, 1.00 equiv) in dry THF (1.7 mL) were added tosyl chloride (0.0367 g, 0.189 mmol, 1.10 equiv), 1,4-cyclohexadiene (40.6 µL, 0.429 mmol, 2.50 equiv) then *t*-BuOK (0.0481 g, 0.429 mmol, 2.50 equiv). The reaction mixture was stirred at –78 ºC for 30 min. A saturated solution of ammonium chloride (2 mL) was added, then the mixture was warmed to 22 ºC. The organic layer was separated. The aqueous layer was extracted with EtOAc (3 × 2 mL). The combined organic layers were then washed with brine (1 × 2 mL), dried with sodium sulfate, filtered, then concentrated in vacuo. The crude NMR spectrum indicated that the desired product **14** was formed (^{1}H NMR yield: 93%).

VII. Miscellaneous Mechanistic Experiments

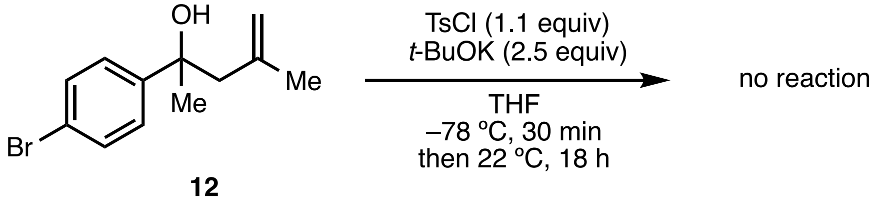

To a cooled (–78 ºC) solution of alcohol **12** (0.0300 g, 0.118 mmol, 1.00 equiv) in dry THF (1.2 mL) were added tosyl chloride (0.0252 g, 0.129 mmol, 1.10 equiv) then *t*-BuOK (0.0330 g, 0.294 mmol, 2.50 equiv). The reaction mixture was stirred at –78 ºC for 30 min. Then, the reaction mixture was warmed to 22 ºC and stirred for 18 h. A saturated solution of ammonium chloride (1 mL) was added. The organic layer was separated. The aqueous layer was extracted with EtOAc (3 × 1 mL). The combined organic layers were then washed with brine (1 × 1 mL), dried with sodium sulfate, filtered, then concentrated in vacuo. Analysis by NMR spectroscopy indicated that no reaction occurred.

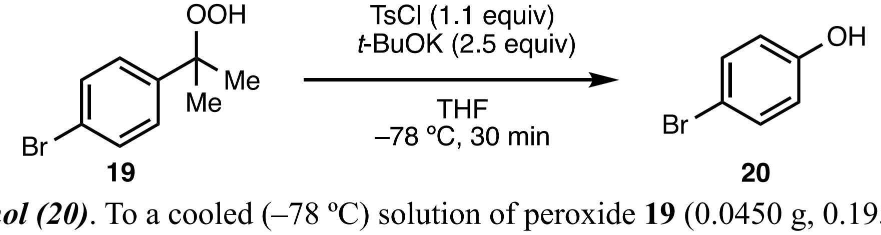

4-Bromophenol (20). To a cooled (–78 ºC) solution of peroxide **19** (0.0450 g, 0.195 mmol, 1.00 equiv) in dry THF (2 mL) were added tosyl chloride (0.0417 g, 0.214 mmol, 1.10 equiv) then *t*-BuOK (0.0240 g, 0.214 mmol, 1.10 equiv). The reaction mixture was stirred at –78 ºC for 30

min. A saturated solution of ammonium chloride (2 mL) was added, then the mixture was warmed to 22 ºC. The organic layer was separated. The aqueous layer was extracted with EtOAc (3 × 2 mL). The combined organic layers were then washed with brine (1 × 2 mL), dried with sodium sulfate, filtered, then concentrated in vacuo. The crude product was purified by automated column chromatography (0-50% EtOAc in hexanes) to yield the desired alcohol **20** as a clear oil (0.0145 g, 43% yield). The spectroscopic data are consistent with the data reported:[21]

^{1}H NMR (400 MHz, $CDCl_3$) δ 7.33 (d, *J* = 9.0 Hz, 2H), 6.72 (d, *J* = 9.0 Hz, 2H), 4.81 (s, 1H);

^{13}C{^{1}H} NMR (100 MHz, $CDCl_3$) δ 154.8 (C), 132.6 (CH), 117.3 (CH), 113.0 (C).

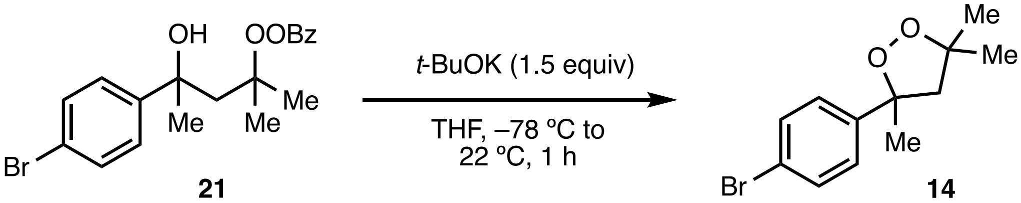

3-(4-Bromophenyl)-3,5,5-trimethyl-1,2-dioxolane (14). To a cooled (–78 ºC) solution of benzoylated peroxide **21** (0.0088 g, 0.022 mmol, 1.0 equiv) in dry THF (1 mL) was added *t*-BuOK (0.0038 g, 0.034 mmol, 1.5 equiv). The reaction mixture was stirred and warmed to 22 ºC for 1 h. A saturated solution of ammonium chloride (1 mL) was added. The organic layer was separated. The aqueous layer was extracted with EtOAc (3 × 1 mL). The combined organic layers were then washed with brine (1 × 1 mL), dried with sodium sulfate, filtered, then concentrated in vacuo. The crude NMR spectrum indicated that the desired product **14** was formed (^{1}H NMR yield: 55%).

VIII. Crystallographic Data of Endoperoxide 36

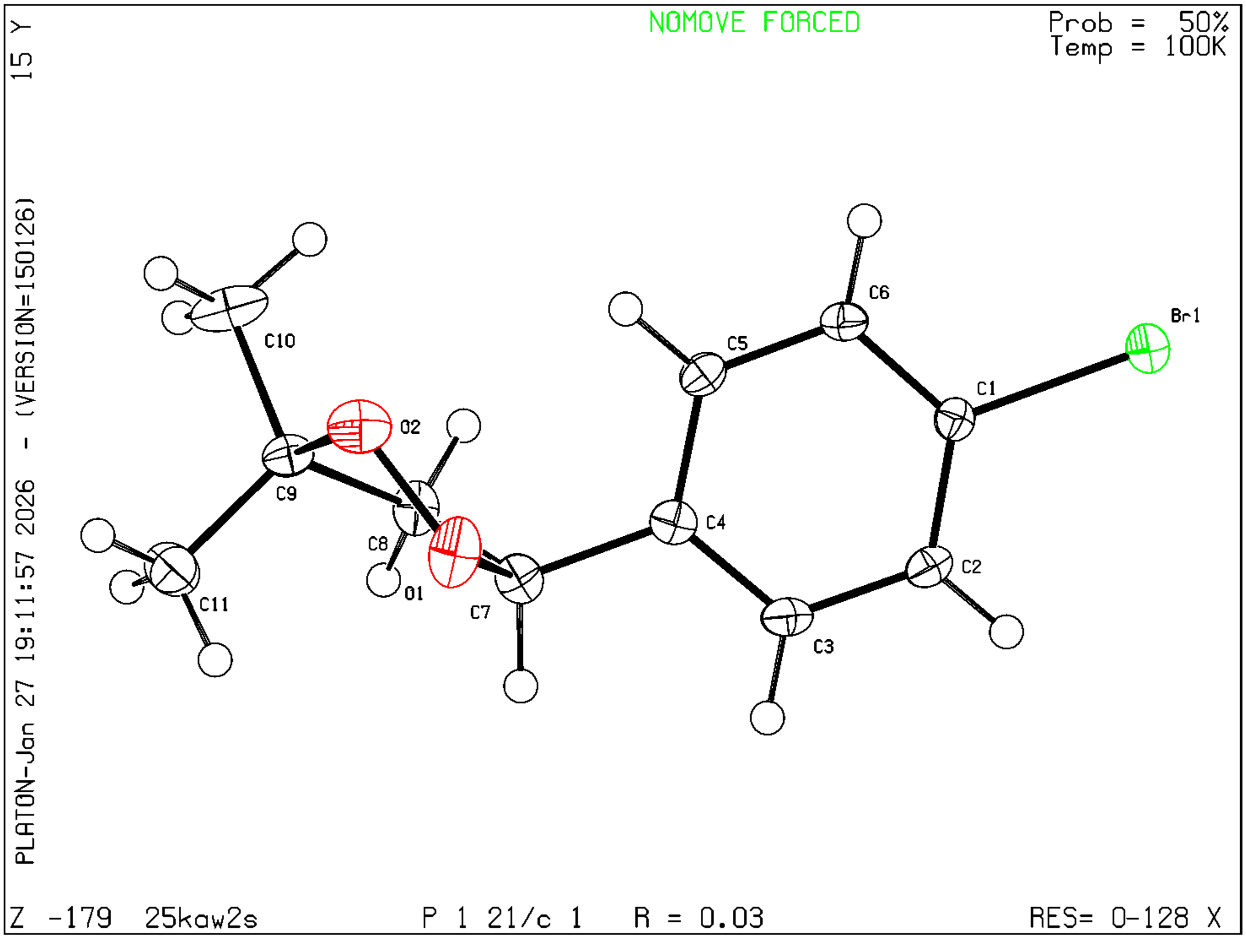

Table S1. Sample and crystal data for endoperoxide **36**.

Identification code	25kaw2s	
Accession code	CCDC 2526365	
Chemical formula	$C_{11}H_{13}BrO_2$	
Formula weight	257.13 g/mol	
Temperature	100 K	
Wavelength	0.71073 Å	
Space group	P 1 21/c 1	
Unit cell dimensions	a = 11.5728(5) Å	α = 90°
	b = 8.7766(4) Å	β = 111.316(2)°
	c = 11.2262(5) Å	γ = 90°
Volume	1062.24(8) $Å^3$	
Z	4	

Density (calculated)	1.608 g/cm^3
Absorption coefficient	3.841 mm^{-1}
F(000)	520.0

IX. Computational Investigations of the Cyclization Reaction

Computational studies of the cyclization reaction were performed to gain insight into the thermodynamics and the most likely mechanism of the reaction (Scheme 2A and eq S1). Based upon the observation that two equivalents of base were needed for efficient cyclization, the cyclization step likely involved an alkoxide attacking a peroxysulfonate to displace the tosylate ion (eq S1). The structures of these three compounds were therefore examined computationally, and studies were conducted to identify a transition state for this process. Calculations were performed using Spartan'24 (Wavefunction, Inc.) by density functional methods (B3LYP/6-31G*) using the integrated equation formalism of the polarizable continuum model (IEFPCM) with THF as the solvent for the final structures (C-PCM dielectric = 7.43).

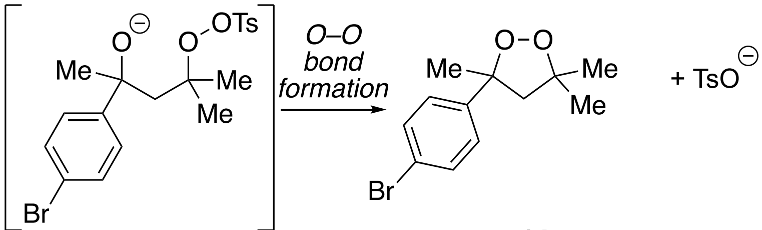

15 14 (S1)

Determining the structure of alkoxide **15** was performed in two stages. First, a conformational search was performed for the neutral alcohol corresponding to **15** using the Merck molecular force field (MMFF; the force field could not accommodate the anion). A total of 130 conformers were identified within 10 kcal/mol of the minimum energy structure. These conformers were optimized using semi-empirical methods (PM3). The conformers of the alcohol within 4.0 kcal/mol of the lowest energy one were retained (a total of 55 conformers). These 55 structures were converted to the corresponding alkoxides and their geometries were optimized (B3LYP/6-31G*) in THF solvent. Several of these conformers collapsed to form cyclic peroxide **14**. This problem may indicate that cyclization of the anion has a low activation barrier (as noted below) or it may reflect the difficulty of modeling an anion without a counterion and explicit solvent molecules. The lowest energy structures (within 2 kcal/mol of the minimum energy structure) were subjected to vibration calculations to determine thermodynamic parameters and to establish that they were energy minima. In some of these calculations, tight restrictions on tolerances of geometry and gradient ("GEOMTOL = TIGHT") were required to minimize the number of low-frequency imaginary frequencies. One conformer for alkoxide **15** is reported below, but four other distinct conformers were found to have G° values within 1 kcal/mol of the lowest one.

Similar procedures were performed for endoperoxide **14** and the tosylate ion. Only three conformers of **14** were found, and only one was found for the tosylate ion. All structures were determined to be energy minima by vibrational calculations, which showed no imaginary

frequencies. These computations permitted an estimate of ΔG° = –67.0 kcal/mol at 298 K for the overall reaction shown in eq S1.

Identifying a transition state for these reactions required several steps. An energy profile search of alkoxide **15** was performed where the distance was decreased between the two oxygen atoms that would form the bond. The maximum energy structure (less than 2 kcal/mol higher than the lowest energy structure reported below) was found with a O···O distance of about 2.6 Å, which is about 70% longer than the O–O bond in the cyclic peroxide **14**. Any shorter distance led to collapse to the cyclic peroxide **14**. This distance was refined more finely resulted in a similar profile, with the cutoff point at 2.51 Å. Several starting points were used to identify a transition state, but all these structures either collapsed to the cyclic peroxide **14** or the groups rotated away from each other to return to the alkoxide **15**. With the O···O distance of about 2.6 Å, profiles of the O–OTs bond length were performed to identify a different starting structure for finding a transition state, and a similar profile was observed. One of these structures, however, was used to identify a transition state that was only 1.4 kcal/mol higher than the lowest energy conformer of the starting alkoxide **15**.

Taken together, these calculations suggest that the overall conversion from the anion **15** to the endoperoxide **14** and the tosylate ion is highly exothermic, which would be expected considering where the negative charge was distributed. The identification of a low-energy transition state ($\Delta G^\ddagger$ = 1.4 kcal/mol) may not be quantitatively accurate, but it does suggest that cyclization is rapid, which is consistent with the fact that these reactions occur in short periods of time at –78 °C.

The structures of the relevant compounds are described below.

<u>Alkoxide **15**</u>

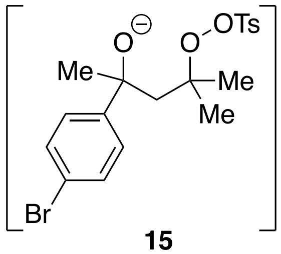

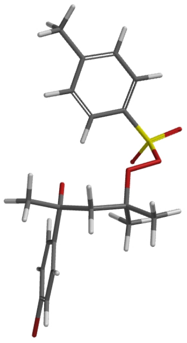

15

Energy: –4085.09837 au
Number of imaginary vibrations: 1 (*i*59 cm–1, corresponding to rotations and bending near the CO–OS bond; this imaginary frequency is retained in the transition state, so is likely not contributing much to their relative energy.[22]
Bond length: O–OTs, 1.476 Å

Table S1: Cartesian Coordinates (Å) for **15**

Atom	X	Y	Z
1 C C2	0.5707132	0.2726176	-1.6236051
2 C C3	-0.8982075	0.8264759	-1.4111565
3 H H3a	-0.8140690	1.7738151	-0.8605406
4 H H3b	-1.3675731	1.0856422	-2.3690685
5 C C4	1.4151063	1.5184592	-2.0802936
6 H H4c	1.0195854	2.0368533	-2.9654416
7 H H4b	2.4391841	1.1928419	-2.3000697
8 H H4a	1.4593384	2.2347406	-1.2495941
9 C C5	0.5902596	-0.6981413	-2.8574752
10 C C6	0.8006606	-2.5063418	-4.9930881
11 C C7	1.2918072	-1.9023174	-2.7255093
12 C C8	0.0103650	-0.4200195	-4.1056807
13 C C9	0.1023067	-1.3147060	-5.1762161
14 C C10	1.4053427	-2.8145161	-3.7767005
15 H H7	1.7367603	-2.0983844	-1.7545814
16 H H8	-0.5288631	0.5107718	-4.2664388
17 H H9	-0.3626887	-1.0846081	-6.1298109
18 H H10	1.9487296	-3.7461312	-3.6483344
19 O O3	1.1136003	-0.2956628	-0.5264490
20 Br Br1	0.9148863	-3.7654734	-6.4558370
21 C C11	-1.9281040	-0.0323562	-0.6312894
22 C C12	-3.3485131	0.5036679	-0.8526110
23 H H12c	-3.6636013	0.3157896	-1.8848422
24 H H12b	-3.3915455	1.5827863	-0.6675617
25 H H12a	-4.0596767	0.0052527	-0.1849122
26 C C13	-1.8489453	-1.5297867	-0.9093080
27 H H13c	-0.8382729	-1.8825226	-0.7092609
28 H H13b	-2.0978266	-1.7179233	-1.9588490
29 H H13a	-2.5647583	-2.0768581	-0.2883777
30 O O1	-1.5316311	0.2903478	0.7440796
31 O O2	-2.3760279	-0.4149679	1.7280799
32 S S1	-1.3626385	-1.2806673	2.7378484
33 C C1	-0.1820162	-0.0873784	3.3313541
34 C C14	1.6906442	1.7083542	4.3506751
35 C C15	0.8378336	0.3704030	2.4866963
36 C C16	-0.2917440	0.3322530	4.6597369
37 C C17	0.6505387	1.2285967	5.1597127
38 C C18	1.7588745	1.2726537	3.0179021
39 H H15	0.8966096	0.0796714	1.4288883
40 H H16	-1.0937695	-0.0386811	5.2884735
41 H H17	0.5752466	1.5594014	6.1923831
42 H H18	2.5500924	1.6498755	2.3736452
43 C C19	2.7258716	2.6549113	4.9083277
44 H H19c	3.5976206	2.0999641	5.2799027

45	H	H19b	3.0860221	3.3489862	4.1418524
46	H	H19a	2.3258488	3.2359499	5.7453312
47	O	O4	-2.2950955	-1.6600187	3.8040318
48	O	O5	-0.6682806	-2.3236200	1.9839819

Endoperoxide **14**

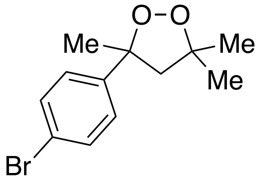

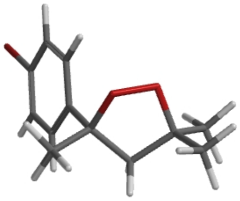

14

G°: –3190.37484 au (298 K)
Number of imaginary vibrations: 0
Bond length: O–O, 1.460 Å

Table S2: Cartesian Coordinates (Å) for **14**

Atom	X	Y	Z
1 O O1	1.7201463	-0.6051316	-0.0474190
2 O O2	0.8647418	-1.7483493	-0.3520820
3 C C3	0.0752750	-1.9274234	0.8594410
4 C C4	-0.1510178	-0.4750827	1.3585296
5 C C5	0.8007826	0.3775344	0.4757495
6 H H4b	-1.1918470	-0.1646292	1.2440154
7 H H4a	0.1146137	-0.3846948	2.4157063
8 C C6	0.0728787	1.1020369	-0.6621250
9 C C7	-1.2147107	2.4939308	-2.7131684
10 C C8	0.4618070	0.9306121	-1.9947495
11 C C9	-0.9710907	1.9938683	-0.3784765
12 C C10	-1.6202043	2.6956739	-1.3951185
13 C C11	-0.1792401	1.6181142	-3.0276252
14 H H8	1.2689275	0.2462858	-2.2303126
15 H H9	-1.2909992	2.1520694	0.6484922
16 H H10	-2.4266583	3.3823024	-1.1602337
17 H H11	0.1270863	1.4683819	-4.0572996
18 Br Br1	-2.1105905	3.4377546	-4.1329222
19 C C1	1.6738769	1.3550758	1.2722051
20 H H1c	2.1956977	0.8281799	2.0771971
21 H H1b	2.4120389	1.8295675	0.6175595
22 H H1a	1.0513968	2.1406765	1.7102351
23 C C2	-1.2010914	-2.6301994	0.4001490
24 H H2c	-1.8550742	-2.8142368	1.2590372
25 H H2b	-1.7418466	-2.0144996	-0.3254703
26 H H2a	-0.9661937	-3.5957273	-0.0610145
27 C C12	0.8712475	-2.7666029	1.8617870
28 H H12c	1.0859143	-3.7571849	1.4462126
29 H H12b	0.3053948	-2.8952234	2.7914538
30 H H12a	1.8187389	-2.2730793	2.1002464

Tosylate Ion

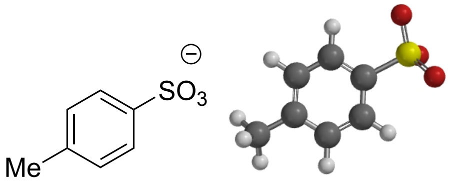

G°: –894.830355 au (298K)
Number of imaginary vibrations: 0

Table S3: Cartesian Coordinates (Å) for Tosylate Ion

Atom	X	Y	Z
1 H H1	-0.1769621	-0.0333076	-2.4447948
2 C C1	-0.1033202	0.0072056	-1.3626093
3 C C4	0.1097979	0.1039222	1.4130257
4 C C2	-0.2463892	1.2204063	-0.6880953
5 C C6	0.1480926	-1.1642893	-0.6448878
6 C C5	0.2546791	-1.1132748	0.7476753
7 C C3	-0.1415280	1.2905454	0.7083330
8 H H4	0.1952636	0.1344028	2.4972828
9 H H2	-0.4407369	2.1282260	-1.2555412
10 H H5	0.4599446	-2.0224963	1.3043096
11 C C7	-0.2660351	2.6118263	1.4311610
12 H H7c	-0.6983494	2.4822344	2.4293085
13 H H7b	-0.8935217	3.3140054	0.8721961
14 H H7a	0.7164990	3.0852521	1.5622615
15 S S1	0.2546175	-2.7510111	-1.5025648
16 O O1	0.7441419	-2.4248883	-2.8736824
17 O O2	1.2163024	-3.5644680	-0.7026401
18 O O3	-1.1324960	-3.3042911	-1.4907380

Transition State for Ring Closure

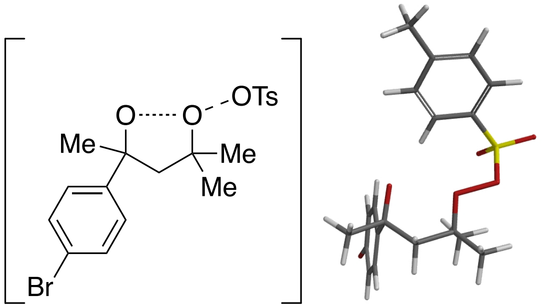

G°: –4085.096196 au (298K), which is 1.4 kcal/mol higher in energy than the calculated structure of **15**
Number of imaginary vibrations: 2 (*i*278, corresponding to shortening of the O···O bond and lengthening of the O–OS bond; *i*67, corresponding to rotations and bending near the CO–OS bond)
Bond lengths: O···O, 2.532 Å; O–OTs, 1.603 Å

Table S4: Cartesian Coordinates (Å) for Transition State for Ring Closure

Atom	X	Y	Z
1 C C2	0.3580369	0.7440158	-1.8533191
2 C C3	-1.1713714	1.0398271	-1.7325141
3 H H3b	-1.2515941	2.0550197	-1.3244663
4 H H3a	-1.6959776	1.0488870	-2.6998795
5 C C4	0.9773053	1.9576787	-2.6361475
6 H H4c	0.5464105	2.1142418	-3.6343515
7 H H4b	2.0561382	1.7964880	-2.7480400
8 H H4a	0.8306854	2.8692187	-2.0435764
9 C C5	0.6231280	-0.5046436	-2.7521962
10 C C6	1.2348951	-2.7635634	-4.2938921
11 C C7	1.5085384	-1.4824748	-2.2856000
12 C C8	0.0685640	-0.6820319	-4.0296436
13 C C9	0.3603264	-1.8064534	-4.8057559
14 C C10	1.8222996	-2.6159063	-3.0402241
15 H H7	1.9345357	-1.3298383	-1.2986286
16 H H8	-0.6113745	0.0613906	-4.4392219
17 H H9	-0.0875351	-1.9325821	-5.7864967
18 H H10	2.5052343	-3.3674049	-2.6557248
19 O O3	0.9164378	0.6469210	-0.6233075
20 Br Br1	1.6237434	-4.3383340	-5.3466018
21 C C11	-2.0072039	0.1229189	-0.7873241
22 C C12	-3.4181951	0.7289088	-0.6930032
23 H H12c	-3.8755312	0.7525267	-1.6880439
24 H H12b	-3.3741443	1.7528771	-0.3070782
25 H H12a	-4.0586620	0.1333378	-0.0374235
26 C C13	-2.0769013	-1.3434416	-1.2213851
27 H H13c	-1.0883739	-1.8039478	-1.2037698
28 H H13b	-2.4760275	-1.4194775	-2.2387553
29 H H13a	-2.7355231	-1.9045354	-0.5508825
30 O O1	-1.3245214	0.2443365	0.4844300
31 O O2	-2.3217965	-0.3188814	1.6055928
32 S S1	-1.4233190	-1.2887047	2.5540765
33 C C1	-0.1601968	-0.2387677	3.2493688
34 C C14	1.8331670	1.3425631	4.4006966
35 C C15	0.8786993	0.2368766	2.4405507
36 C C16	-0.2255199	0.0614625	4.6120648
37 C C17	0.7747880	0.8503148	5.1776655
38 C C18	1.8611061	1.0292155	3.0329740
39 H H15	0.8992958	0.0457371	1.3678977
40 H H16	-1.0432084	-0.3161207	5.2159589
41 H H17	0.7298523	1.0880165	6.2375810
42 H H18	2.6636860	1.4191812	2.4113988
43 C C19	2.9268645	2.1745698	5.0258611
44 H H19c	3.7553965	1.5379667	5.3642817

45 H	H19b	3.3396591	2.8925732	4.3097673
46 H	H19a	2.5604806	2.7244702	5.8987739
47 O	O4	-2.3761149	-1.6701680	3.6068229
48	O5	-0.7861826	-2.3442641	1.7614903

X. References

1. Otte, D. A.; Borchmann, D. E.; Lin, C.; Weck, M.; Woerpel, K. A. *Org. Lett.* **2014**, *16*, 1566–1569.
2. Liu, S.; Thomson, N.; Pettman, A.; Hyder, Z.; Mo, J.; Xiao, J. *J. Mol. Catal. A: Chem*. **2008**, *279*, 210–217.
3. Yang, Y.; Zhong, S.; Ma, D.; Zhao, W.; Wang, G. *J. Org. Chem*. **2025**, *90*, 3653–3658.
4. Bora, S. K.; Medhi, B.; Sarma, M.; Saikia, A. K. *J. Org. Chem*. **2025**, *90*, 6443–6453.
5. Zhang, Y.; Li, N.; Goyal, N.; Li, G.; Lee, H.; Lu, B. Z.; Senanayake, C. H. *J. Org. Chem*. **2013**, *78*, 5775–5781.
6. Tripathi, C. B.; Mukherjee, S. *Angew. Chem. Int. Ed*. **2013**, *52*, 8450–8453.
7. Jin, Y.; Zou, Y.; Hu, Y.; Han, Y.; Zhang, Z.; Zhang, W. *Chem. Eur. J.* **2022**, *28*, e202201517.
8. Paparella, A. N.; Stallone, M.; Pulpito, M.; Perna, F. M.; Capriati, V.; Vitale, P. *Org. Biomol. Chem*. **2024**, *22*, 1885–1891.
9. Stasiak, J. P.; Woerpel, K. A. *Org. Lett*. **2025**, *27*, 2037–2041.
10. Hopkins, B. A.; Garlets, Z. J.; Wolfe, J. P. *Angew. Chem. Int. Ed*. **2015**, *54*, 13390–13392.
11. Peralta-Neel, Z.; Woerpel, K. A. *Org. Lett*. **2021**, *23*, 5002–5006.
12. García-Domínguez, A.; West, T. H.; Primozic, J. J.; Grant, K. M.; Johnston, C. P.; Cumming, G. G.; Leach, A. G.; Lloyd-Jones, G. C. *J. Am. Chem. Soc*. **2020**, *142*, 14649–14663.
13. Sun, G.-J.; Wang, Y.; Kang, Q. *Synthesis* **2015**, *47*, 2931–2936.
14. Chen, Y.-L.; Hoppe, D. *Tetrahedron: Asymm*. **2009**, *20*, 1561–1567.
15. Loh, T.-P.; Yang, J.-Y.; Feng, L.-C.; Zhou, Y. *Tetrahedron Lett*. **2002**, 43, 7193–7196.
16. Li, B.; Driess, M.; Hartwig, J. F. *J. Am. Chem. Soc*. **2014**, *136*, 6586–6589.
17. Schneider, U.; Kobayashi, S. *Angew. Chem. Int. Ed*. **2007**, *46*, 5909–5912.
18. Vacque, V.; Sombret, B.; Huvenne, J. P.; Legrand, P.; Suc, S. *Spectrochim. Acta A Mol. Biomol. Spectrosc.* **1997**, *53*, 55–66.
19. Kobayashi, Y.; Tokoro, Y.; Watatani, K. *Eur. J. Org. Chem.* **2000**, *2000*, 3825–3834.
20. Scheiper, B.; Bonnekessel, M.; Krause, H.; Fürstner, A. *J. Org. Chem.* **2004**, *69*, 3943–3949.
21. Rayment, E. J.; Summerhill, N.; Anderson, E. A. *J. Org. Chem.* **2012**, *77*, 7052–7060.
22. Sousa, S. F.; Fernandes, P. A.; Ramos, M. J. *Phys. Chem. Chem. Phys.* **2012**, *14*, 12431–12441.

XI. NMR Spectra

2-(4-Bromophenyl)-4-methylpent-4-en-2-ol (12).

Solvent: $CDCl_3$
400 MHz
^1H NMR

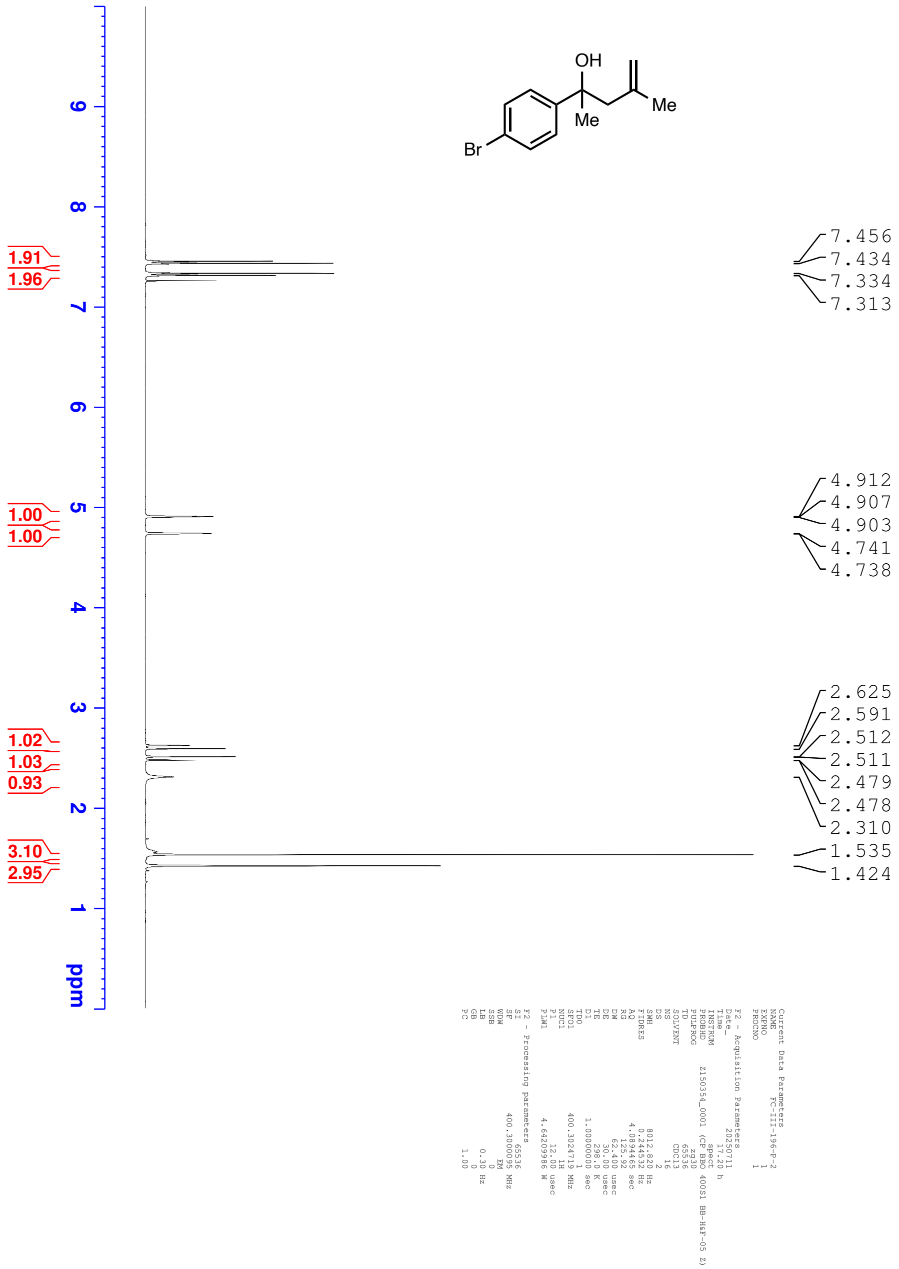

2-(4-Bromophenyl)-4-methylpent-4-en-2-ol (12).

Solvent: $CDCl_3$
100 MHz
$^{13}C\{^1H\}$ NMR

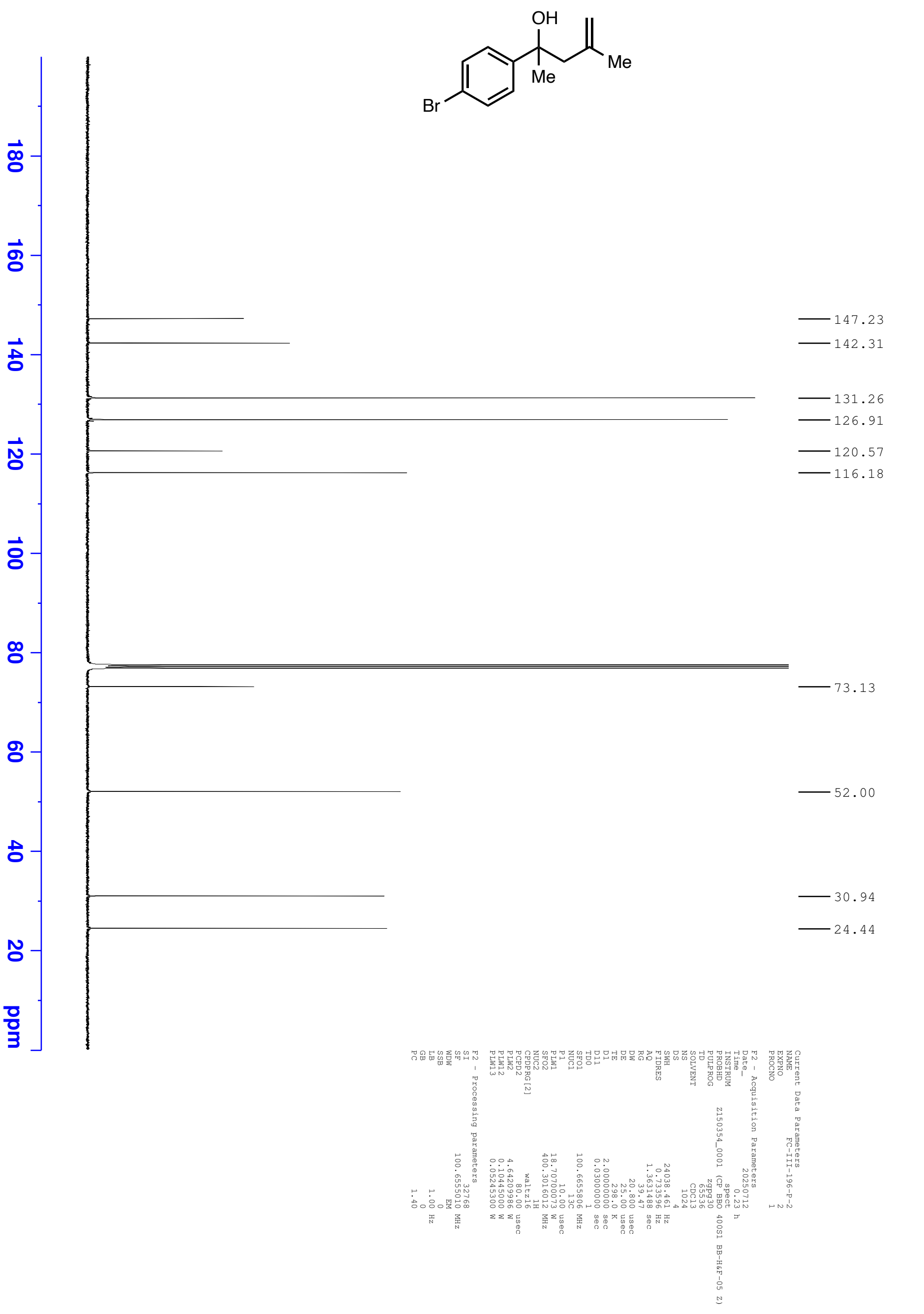

2-(4-Bromophenyl)-4-methylpent-4-en-2-ol-^{17}O (12-^{17}O).

Solvent: $CDCl_3$
54 MHz
^{17}O NMR

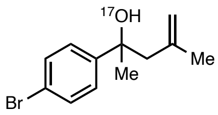

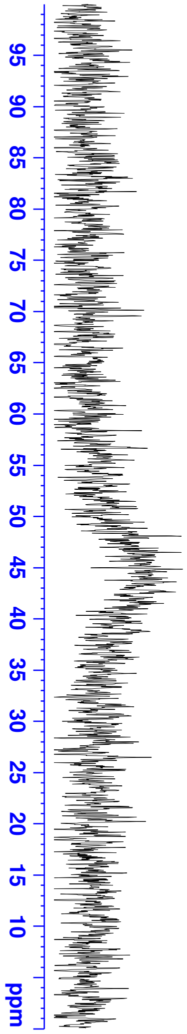


```
Current Data Parameters
NAME              FC-III-196-P-d=0
EXPNO                            1
PROCNO                           1

F2 - Acquisition Parameters
Date_                     20250715
Time                         16.13 h
INSTRUM                      spect
PROBHD     Z133023_0002 (PA BBO 400S1 BBF-H-D-05 XYZ)
PULPROG                         zg
TD                           32768
SOLVENT                      CDCl3
NS                             128
DS                               4
SWH                      27173.912 Hz
FIDRES                    1.658564 Hz
AQ                       0.6029312 sec
RG                          200.67
DW                          18.400 usec
DE                            6.50 usec
TE                           298.0 K
D1                               0 sec
TD0                              1
SFO1                    54.2637906 MHz
NUC1                           17O
P1                           11.00 usec
PLW1                   48.00000000 W

F2 - Processing parameters
SI                           16384
SF                      54.2558351 MHz
WDW                             EM
SSB                              0
LB                            2.00 Hz
GB                               0
PC                            1.40
```

2-(4-Bromophenyl)-4-hydroperoxy-4-methylpentan-2-ol (13).

Solvent: $CDCl_3$
400 MHz
^{1}H NMR

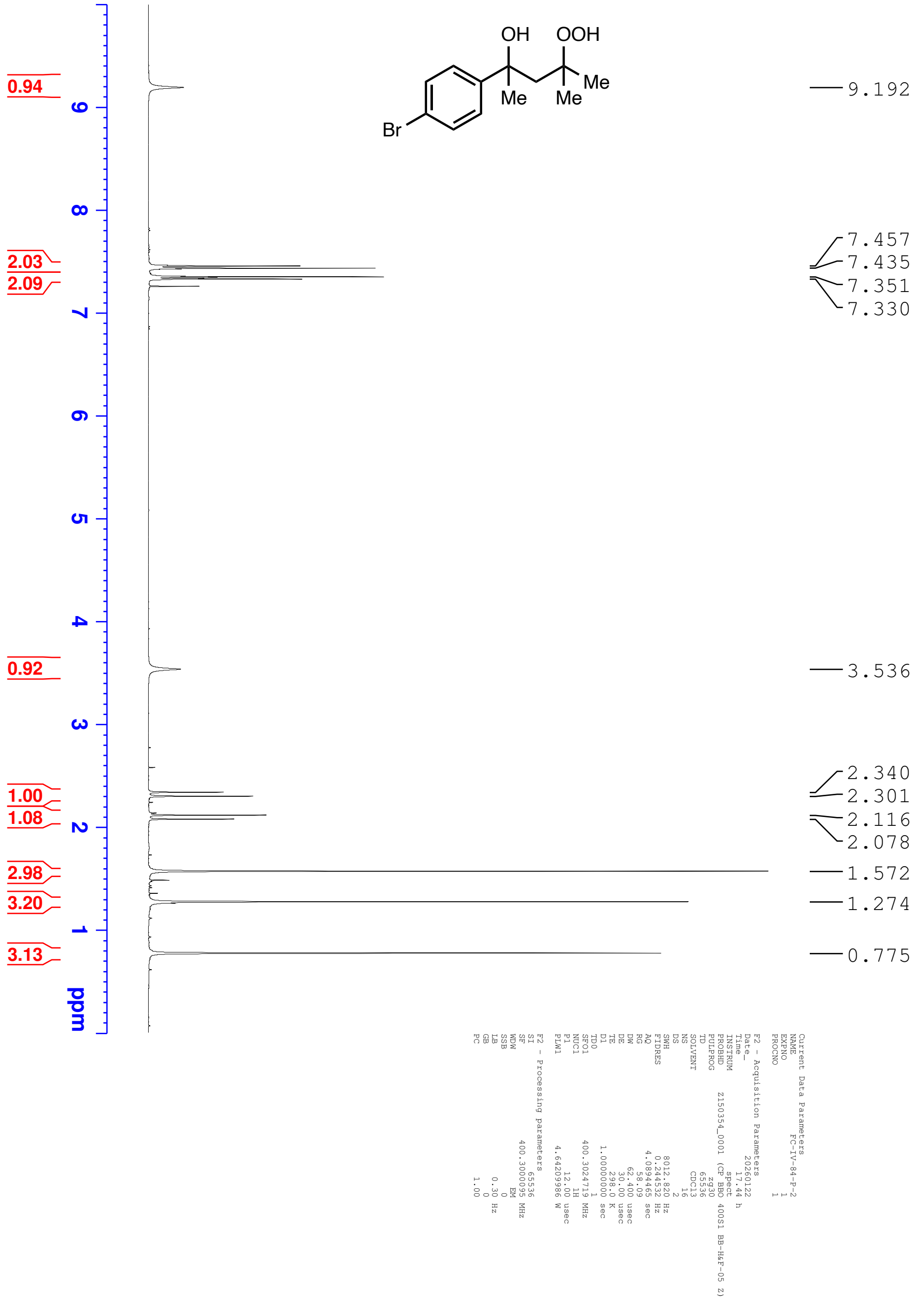

2-(4-Bromophenyl)-4-hydroperoxy-4-methylpentan-2-ol (13).

Solvent: $CDCl_3$
100 MHz
$^{13}C\{^1H\}$ NMR

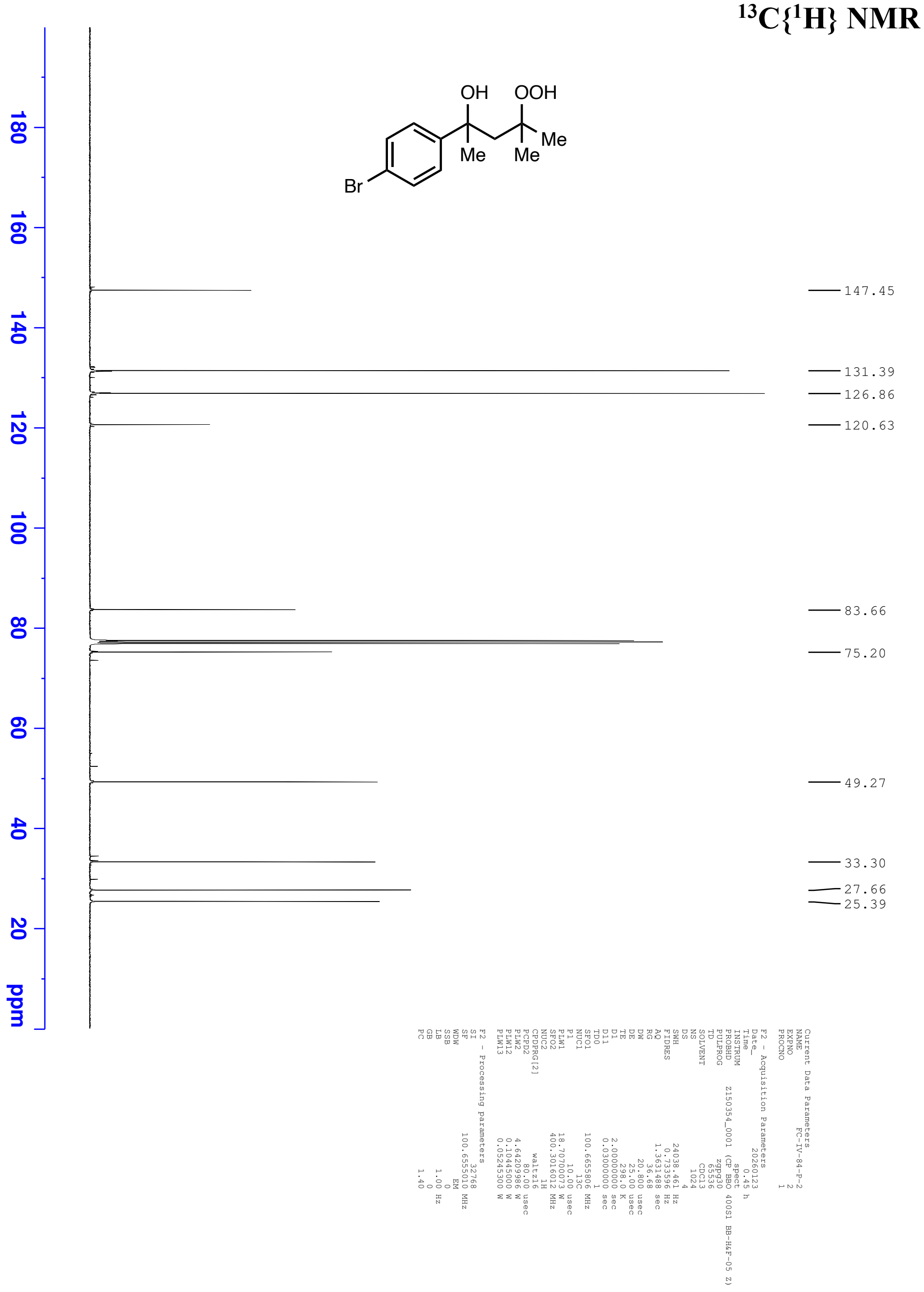

2-(4-Bromophenyl)-4-hydroperoxy-4-methylpentan-2-ol-^{17}O (13-^{17}O).

Solvent: $CDCl_3$
54 MHz
^{17}O NMR

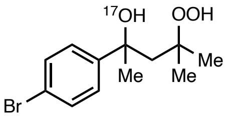

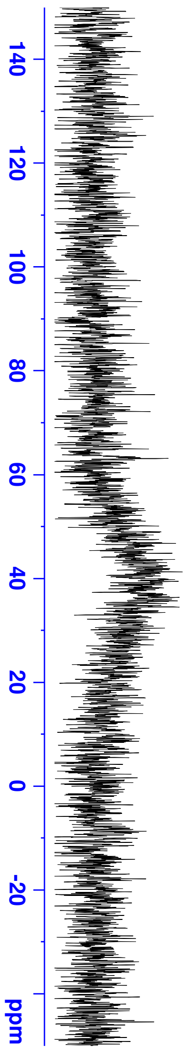


```
Current Data Parameters
NAME          FC-III-198-P-2
EXPNO                      1
PROCNO                     1

F2 - Acquisition Parameters
Date_               20250715
Time                 17.08 h
INSTRUM                spect
PROBHD   Z133023_0002 (PA BBO 400S1 BBF-H-D-05 XYZ)
PULPROG                   zg
TD                     32768
SOLVENT               CH2Cl2
NS                      1024
DS                         4
SWH              27173.912 Hz
FIDRES            1.658564 Hz
AQ               0.6029312 sec
RG                    200.67
DW                    18.400 usec
DE                      6.50 usec
TE                     298.0 K
D1                         0 sec
TD0                        1
SFO1             54.2637906 MHz
NUC1                     17O
P1                     11.00 usec
PLW1             48.00000000 W

F2 - Processing parameters
SI                     16384
SF               54.2558351 MHz
WDW                       EM
SSB                        0
LB                      2.00 Hz
GB                         0
PC                      1.40
```

3-(4-Bromophenyl)-3,5,5-trimethyl-1,2-dioxolane (14).

Solvent: $CDCl_3$
400 MHz
^{1}H NMR

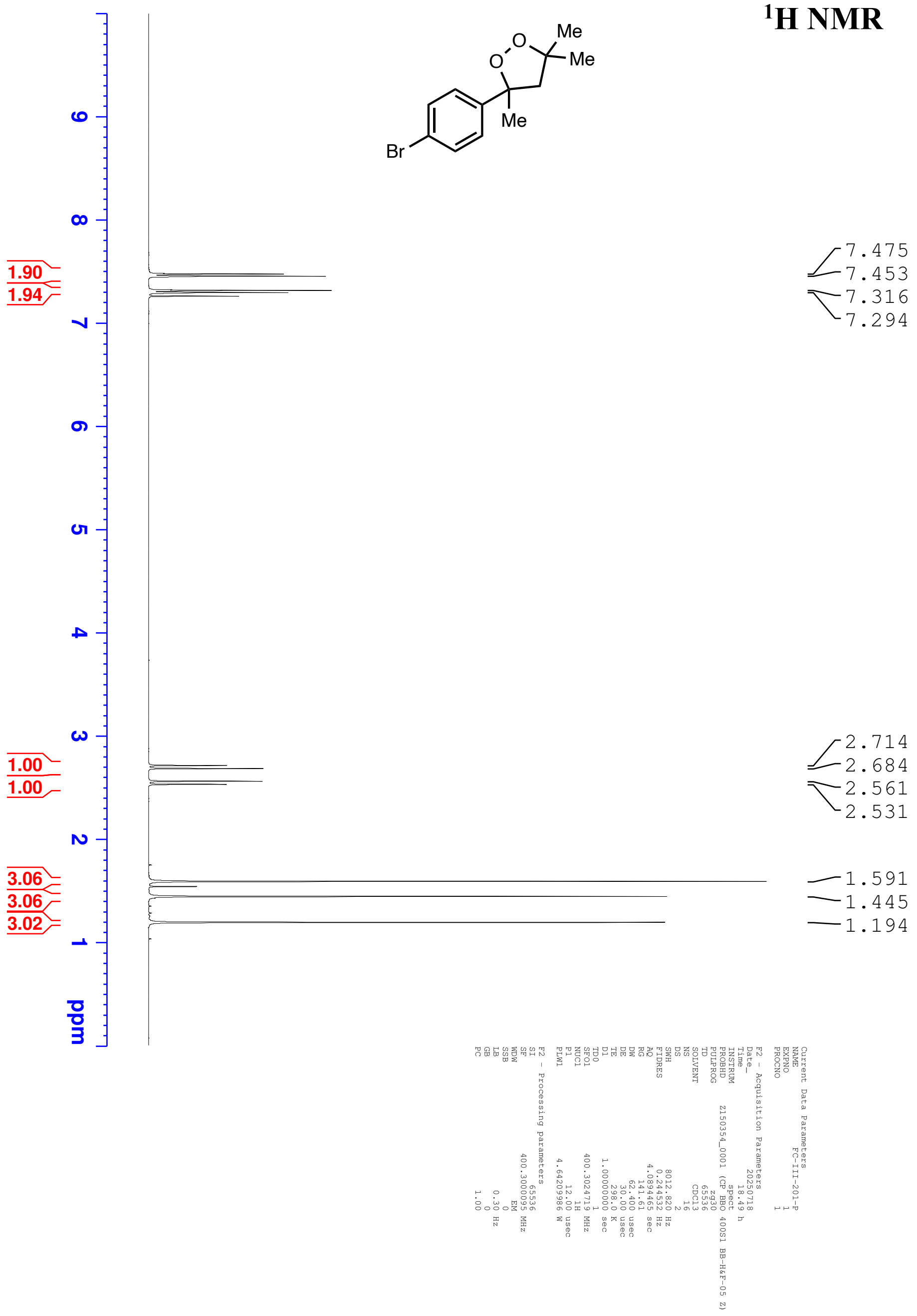

3-(4-Bromophenyl)-3,5,5-trimethyl-1,2-dioxolane (14).

Solvent: $CDCl_3$
100 MHz
$^{13}C\{^1H\}$ NMR

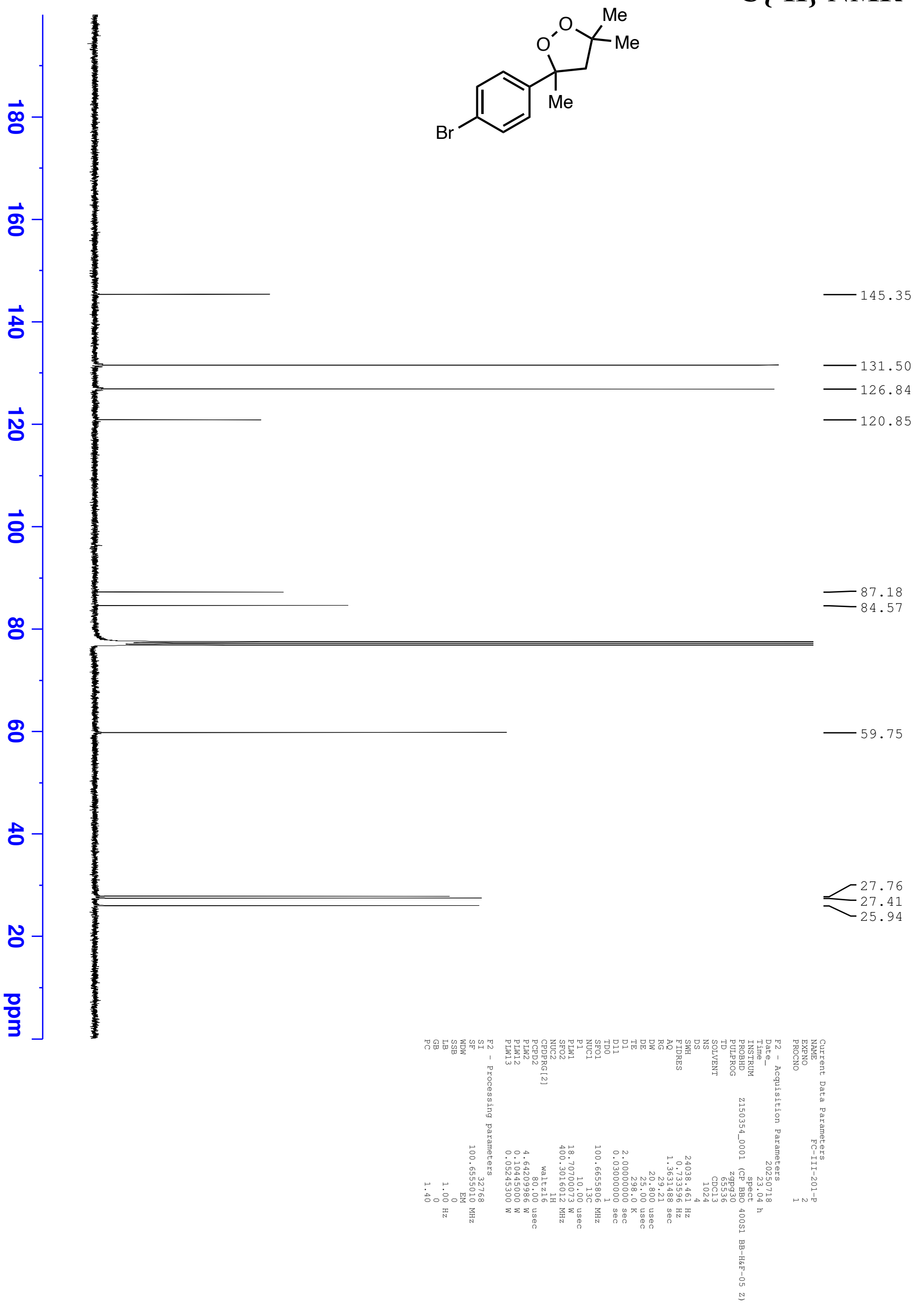

3-(4-Bromophenyl)-3,5,5-trimethyl-1,2-dioxolane-2-^{17}O (14-^{17}O).

Solvent: $CDCl_3$
54 MHz
^{17}O NMR

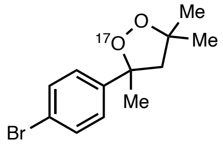

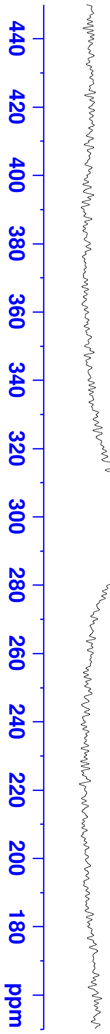


```
Current Data Parameters
NAME          FC-III-201-C-017
EXPNO                        3
PROCNO                       1

F2 - Acquisition Parameters
Date_                 20250717
Time                     14.40 h
INSTRUM                  spect
PROBHD     Z133023_0002 (PA BBO 400S1 BBF-H-D-05 XYZ)
PULPROG                     zg
TD                        1000
SOLVENT                  CDCl3
NS                       20000
DS                           4
SWH                  27173.912 Hz
FIDRES               54.347828 Hz
AQ                   0.0184000 sec
RG                      200.67
DW                      18.400 usec
DE                        6.50 usec
TE                       298.0 K
D1                           0 sec
TD0                          1
SFO1                54.2692161 MHz
NUC1                       17O
P1                       11.00 usec
PLW1               48.00000000 W

F2 - Processing parameters
SI                       16384
SF                  54.2558351 MHz
WDW                         EM
SSB                          0
LB                        2.00 Hz
GB                           0
PC                        1.40
```

1-Bromo-4-(2-hydroperoxypropan-2-yl)benzene (19).

Solvent: $CDCl_3$
400 MHz
^{1}H NMR

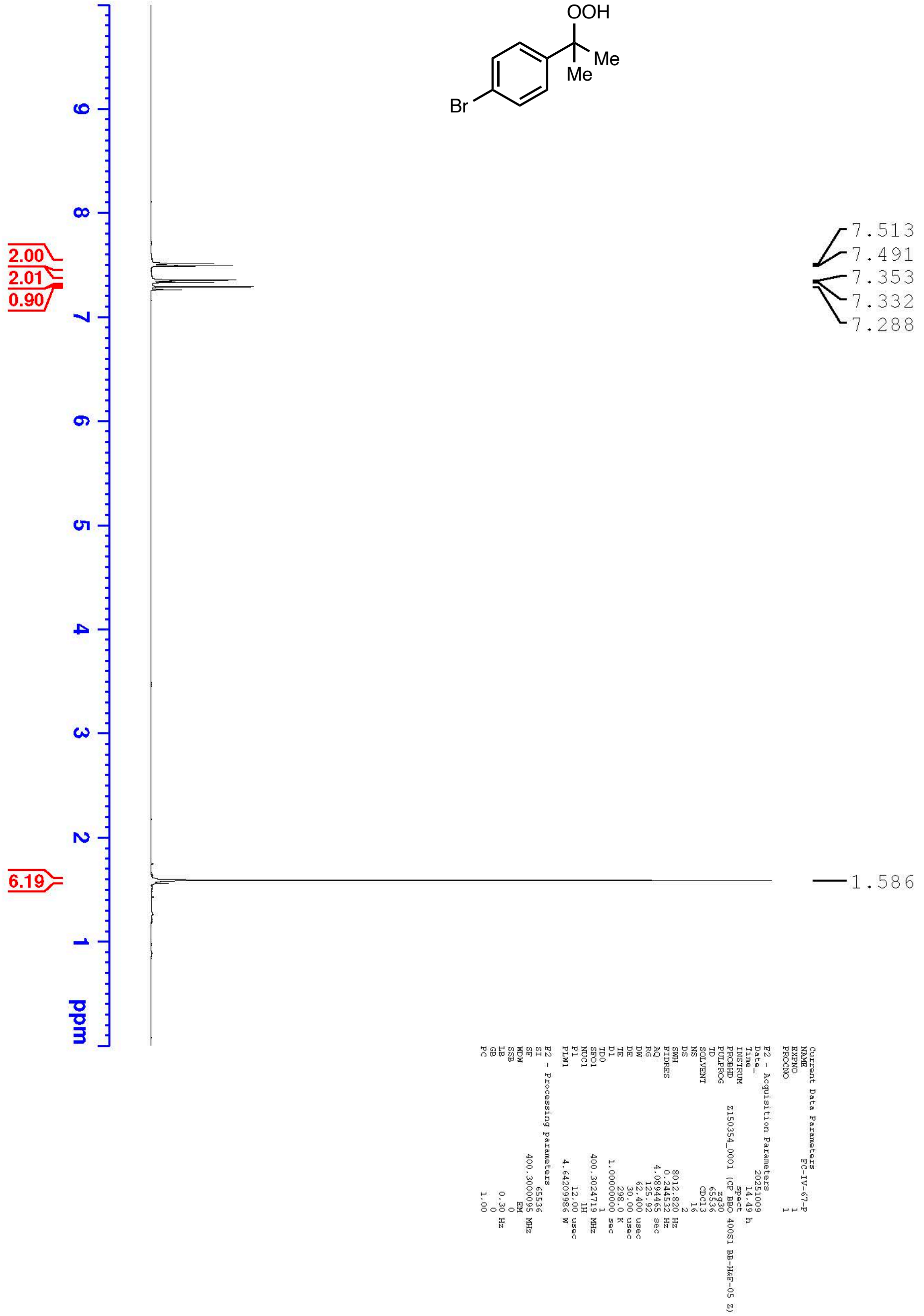

1-Bromo-4-(2-hydroperoxypropan-2-yl)benzene (19).

Solvent: $CDCl_3$
100 MHz
$^{13}C\{^1H\}$ NMR

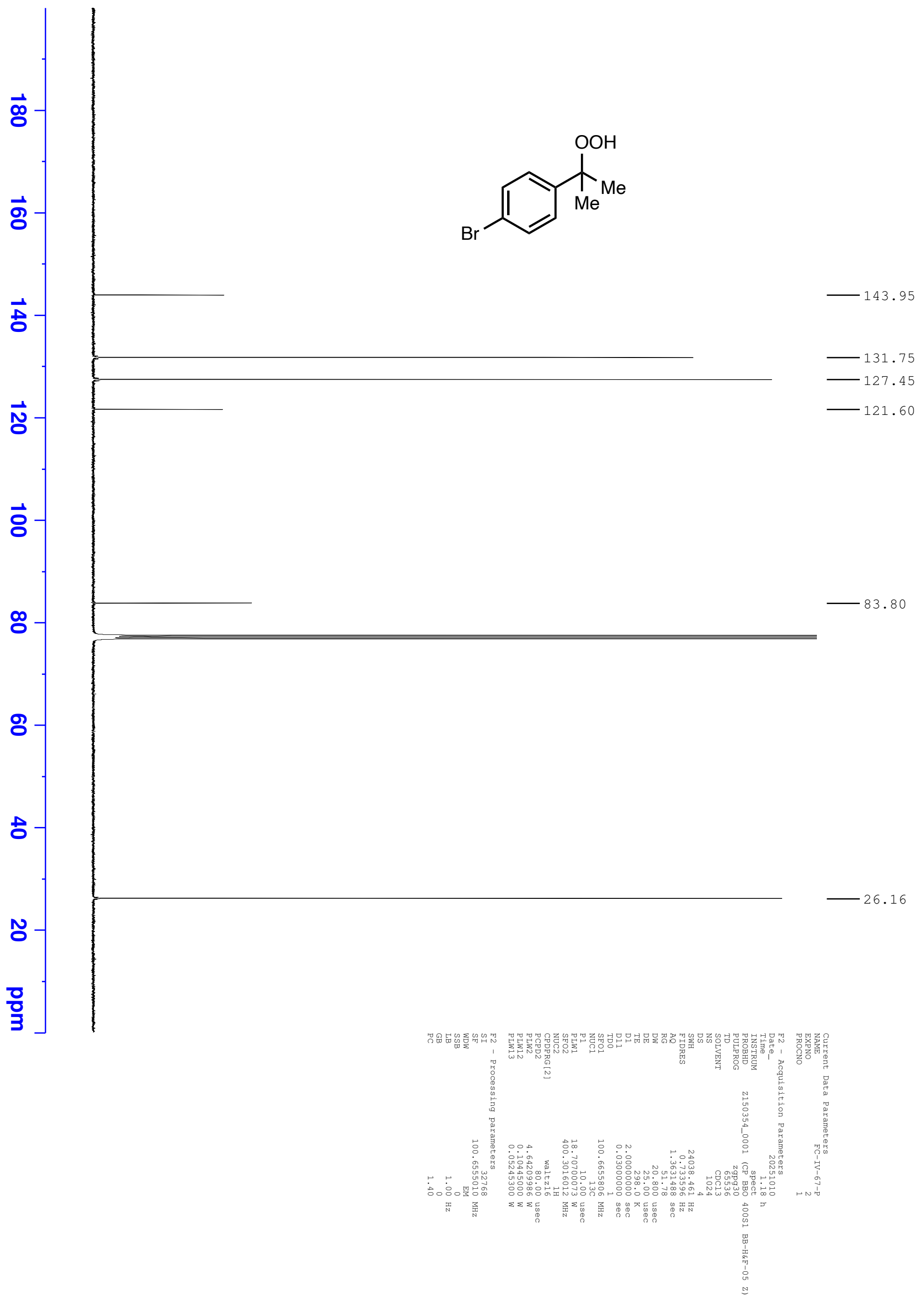

4-(4-Bromophenyl)-4-hydroxy-2-methylpentan-2-yl benzoperoxoate (21).

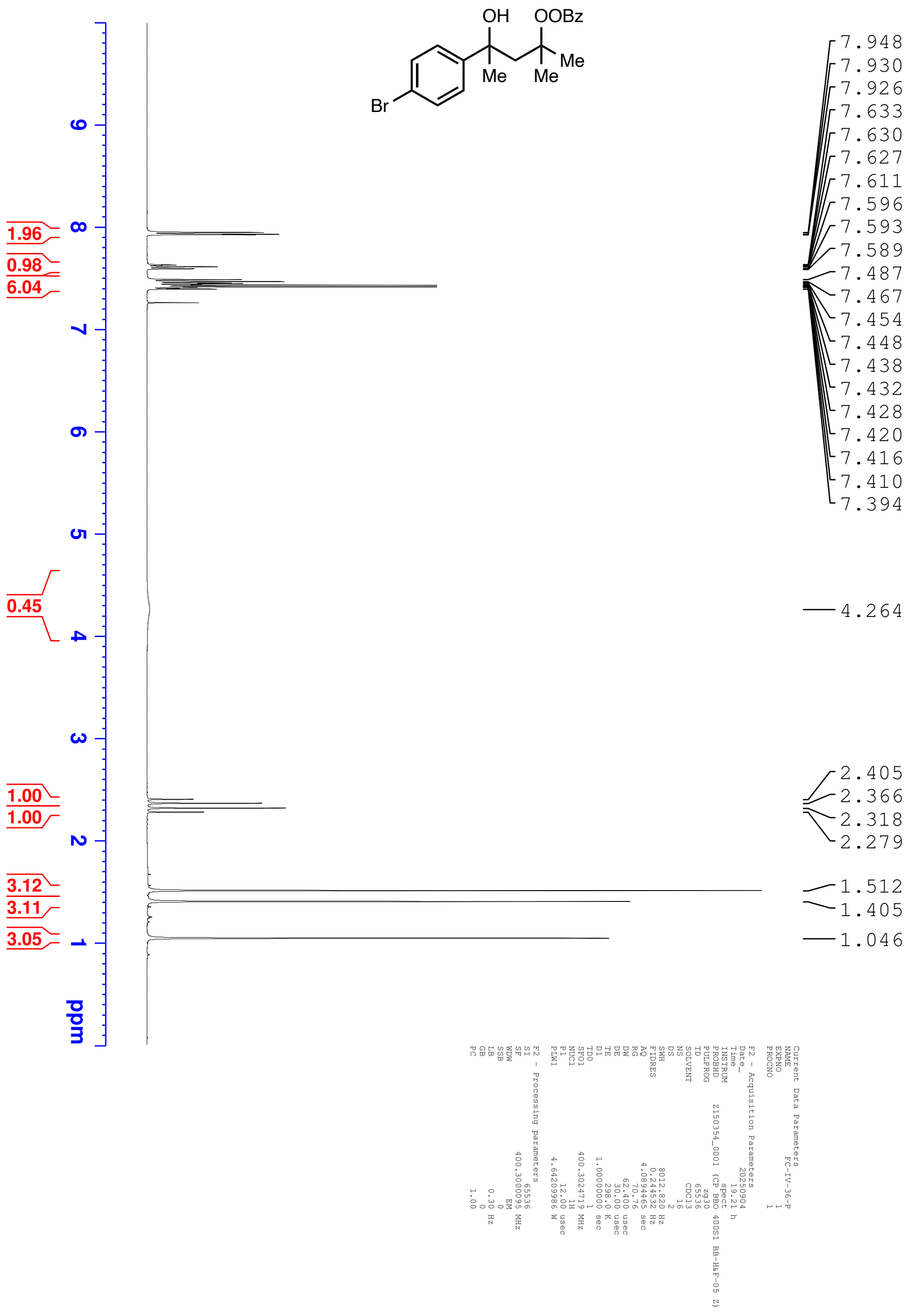

Solvent: $CDCl_3$
400 MHz
^{1}H NMR

4-(4-Bromophenyl)-4-hydroxy-2-methylpentan-2-yl benzoperoxoate (21).

Solvent: $CDCl_3$
100 MHz
$^{13}C\{^1H\}$ NMR

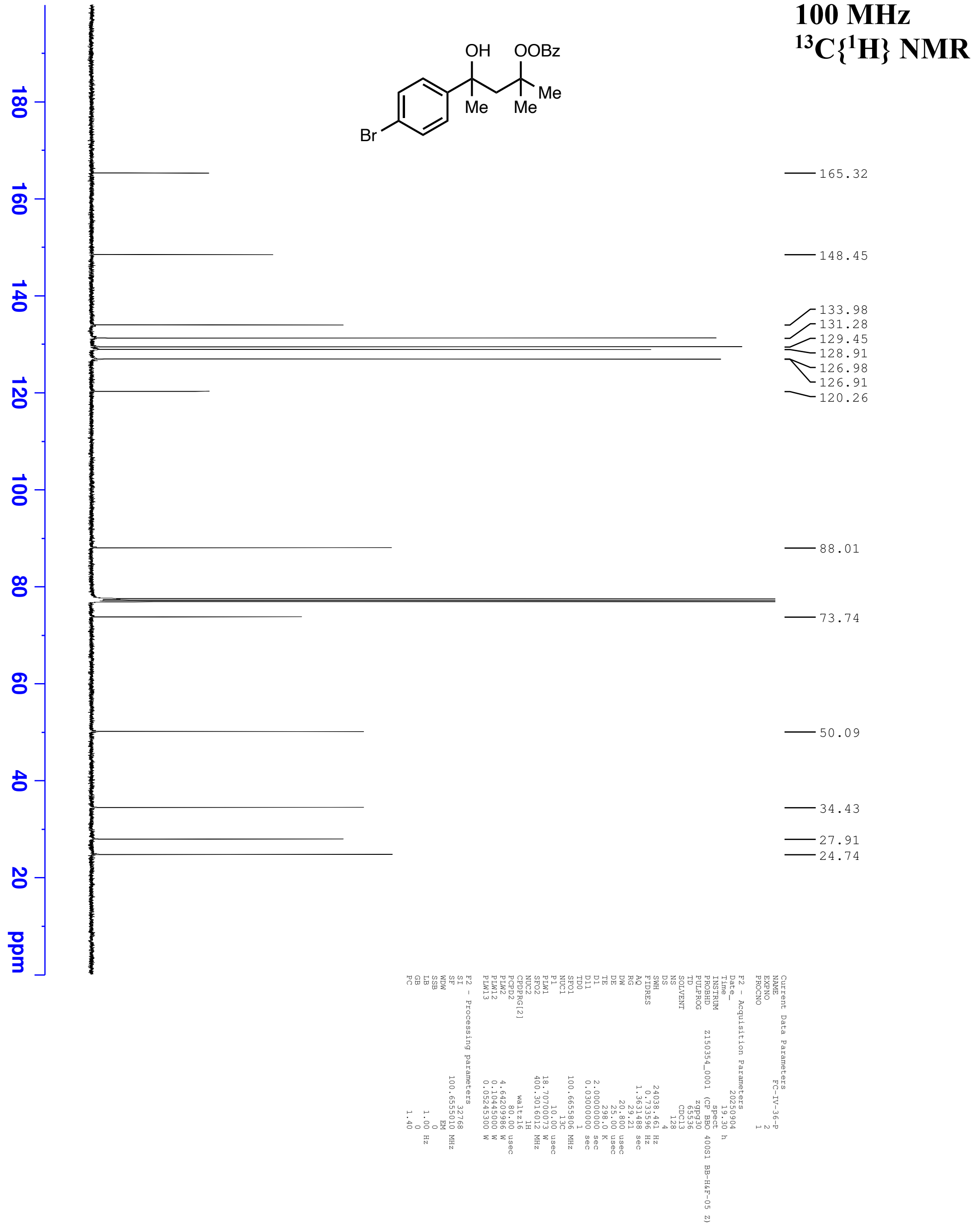

4-(4-Bromophenyl)-4-hydroxy-2-methylpentan-2-yl benzoperoxoate (21).

Solvent: C_6D_6
400 MHz
^{1}H NMR

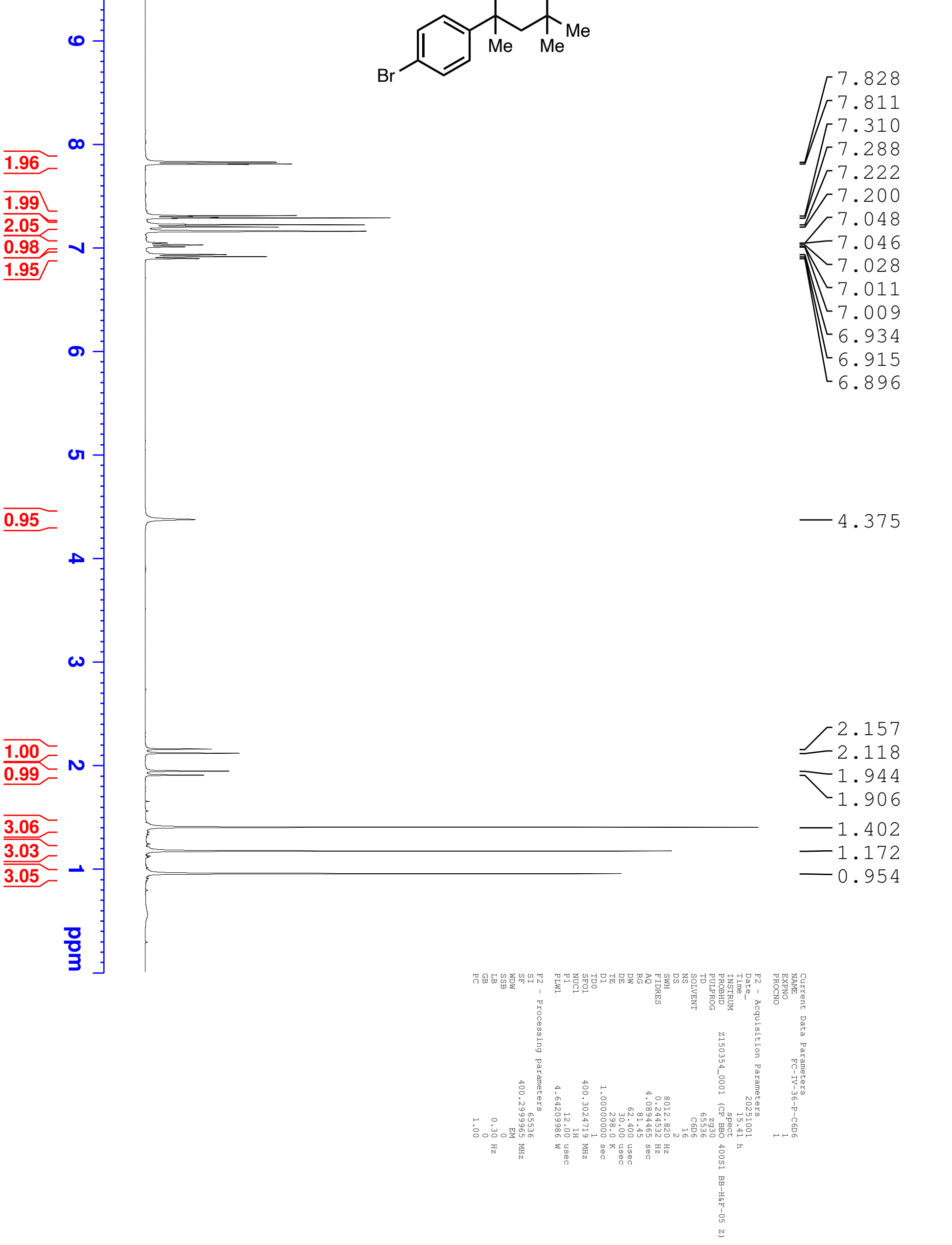

4-(4-Bromophenyl)-4-hydroxy-2-methylpentan-2-yl benzoperoxoate (21).

Solvent: C_6D_6
100 MHz
$^{13}C\{^1H\}$ NMR

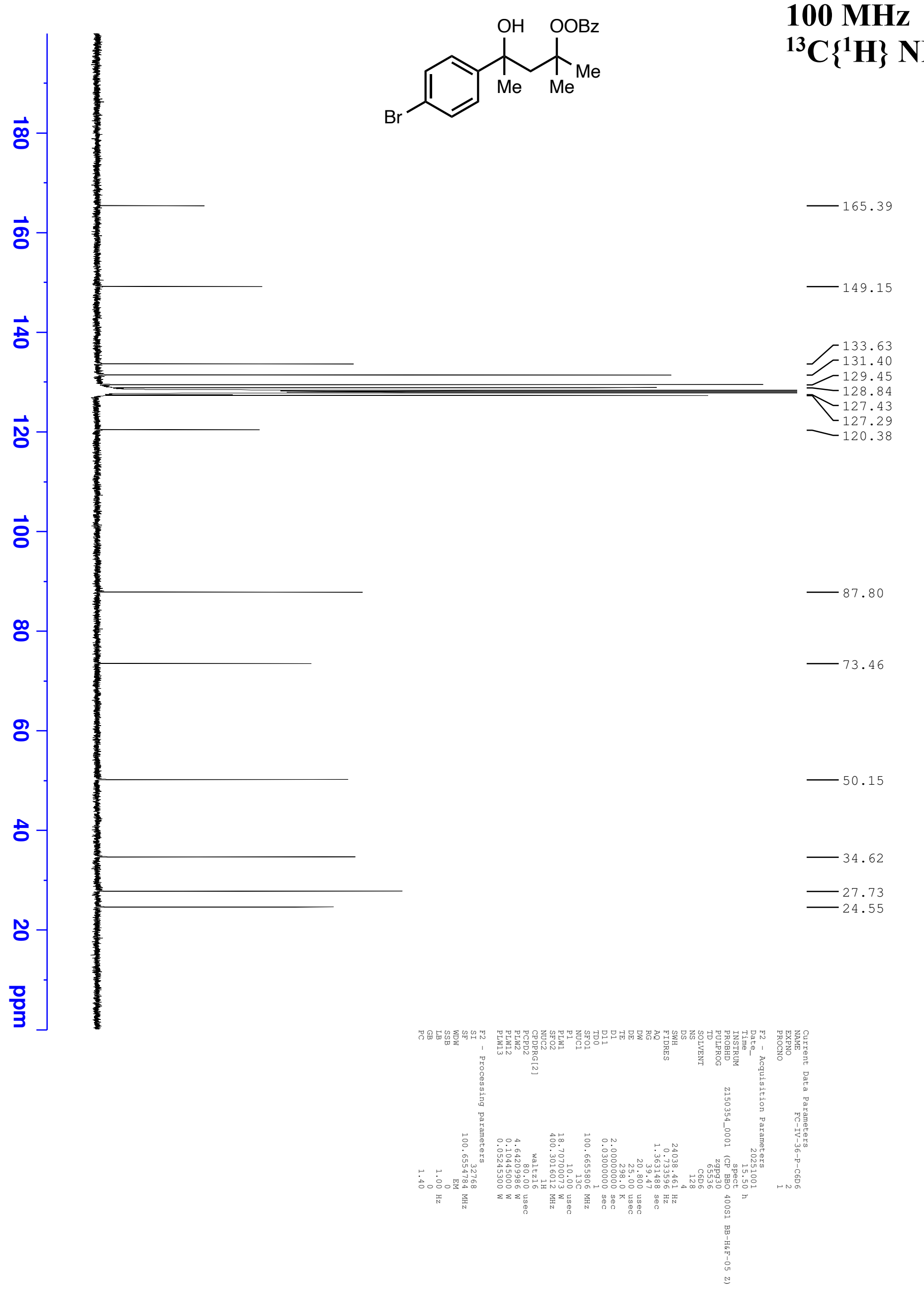

4-(3,5-Bis(trifluoromethyl)phenyl)-2-phenyl-4-((triethylsilyl)peroxy)pentan-2-ol (22).

Solvent: $CDCl_3$
400 MHz
^{1}H NMR

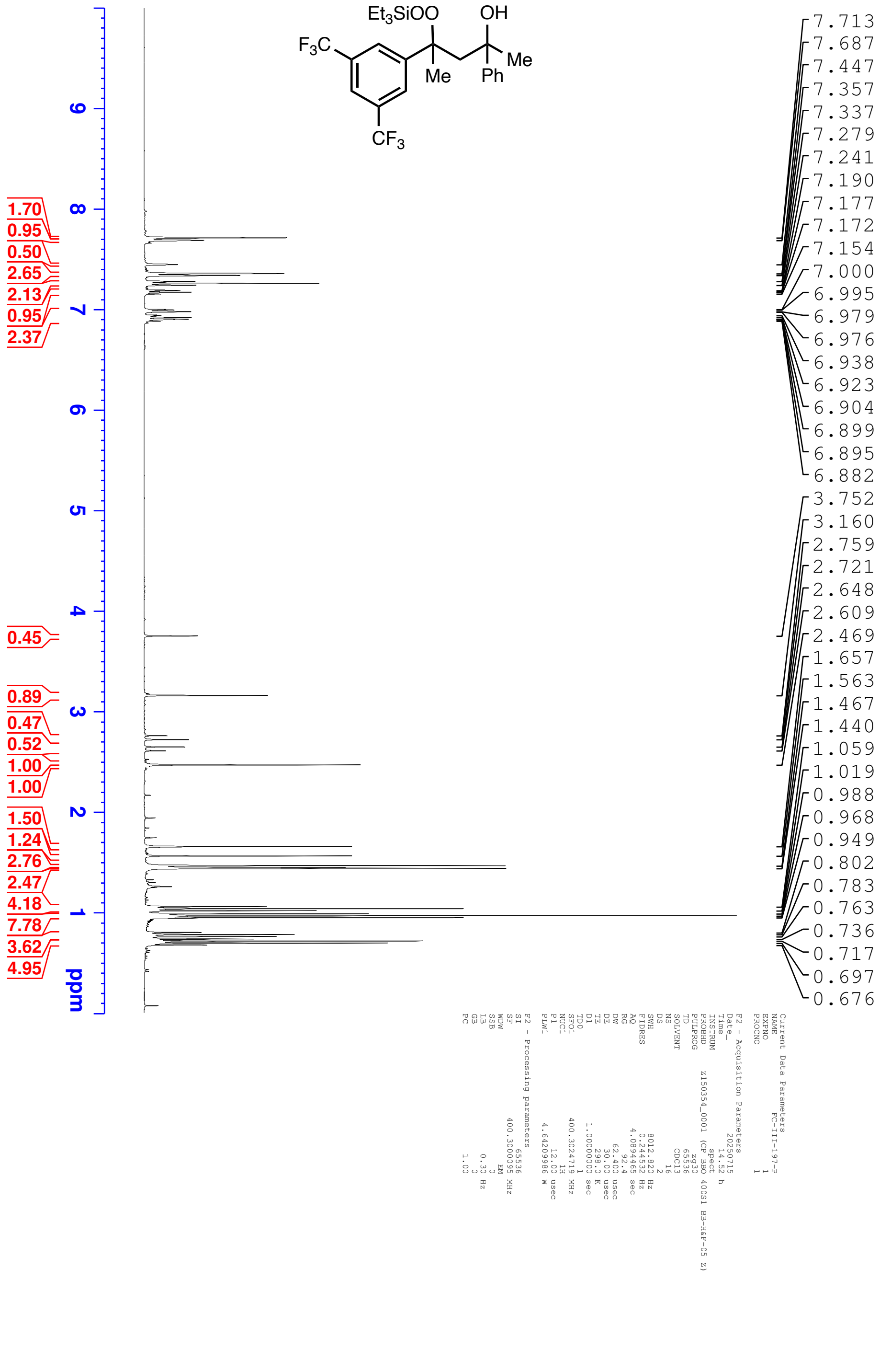

4-(3,5-Bis(trifluoromethyl)phenyl)-2-phenyl-4-((triethylsilyl)peroxy)pentan-2-ol (22).

Solvent: $CDCl_3$
100 MHz
$^{13}C\{^1H\}$ NMR

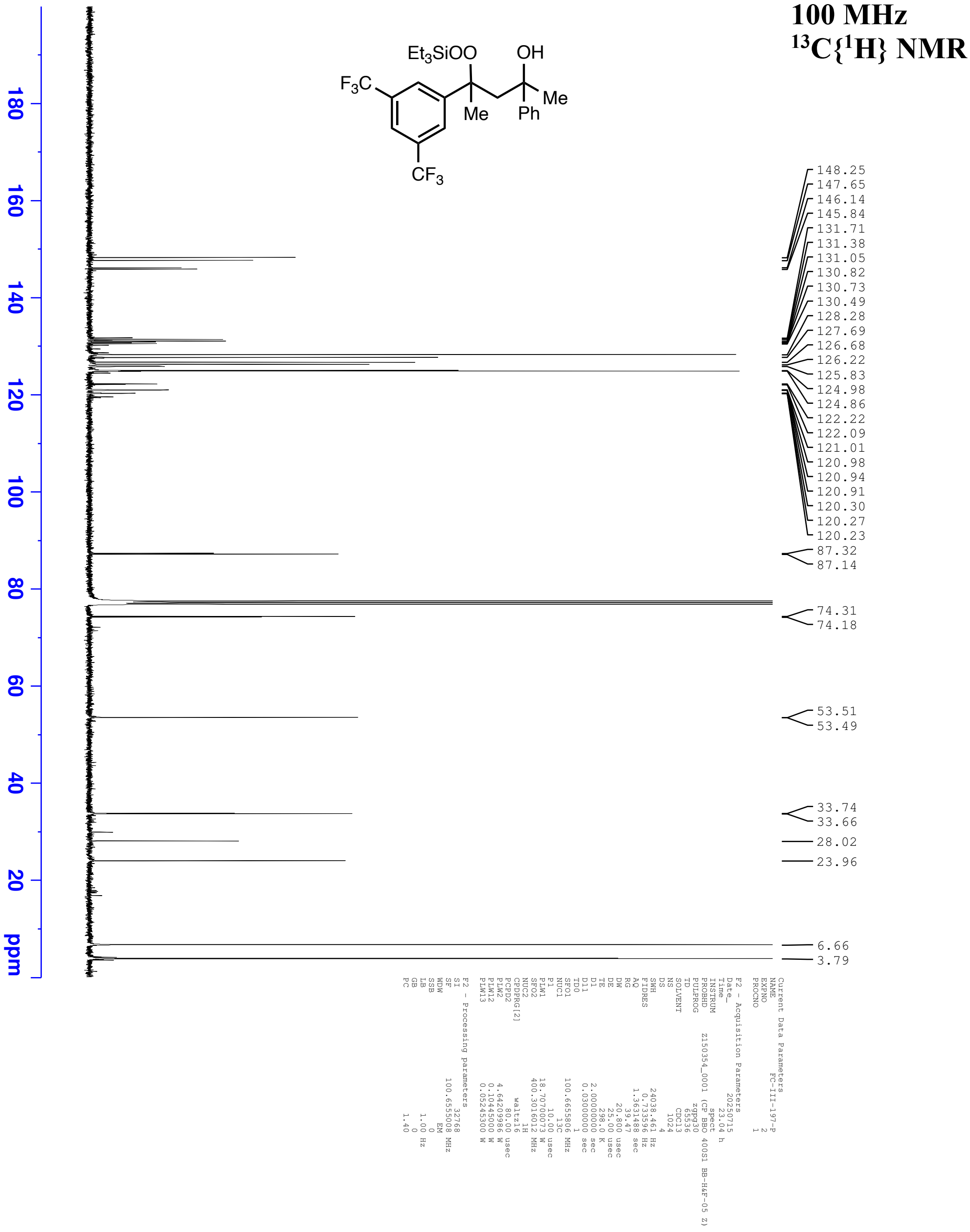

4-(3,5-Bis(trifluoromethyl)phenyl)-2-phenyl-4-((triethylsilyl)peroxy)pentan-2-ol (22).

Solvent: $CDCl_3$
377 MHz
$^{19}F\{^{1}H\}$ NMR

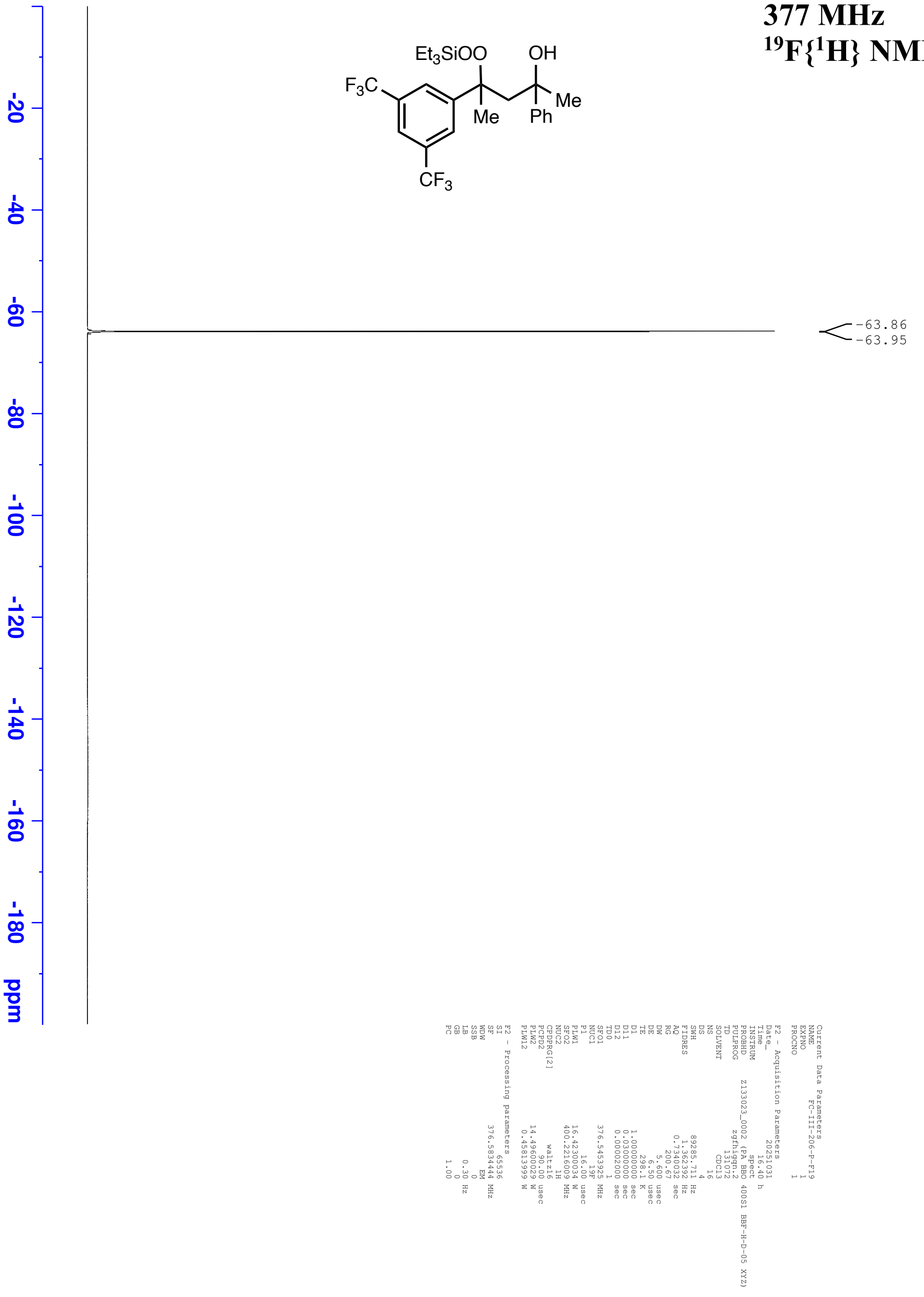

4-(3,5-Bis(trifluoromethyl)phenyl)-4-hydroperoxy-2-phenylpentan-2-ol (23).

The following proton and carbon NMR spectra indicate a ratio of 95:5 of *trans:cis* for **23**, which was not used for characterization. The succeeding proton and carbon NMR of **23** which indicate a ratio of 50:50 of *trans:cis* was used for characterization.

Solvent: $CDCl_3$
400 MHz
^{1}H NMR

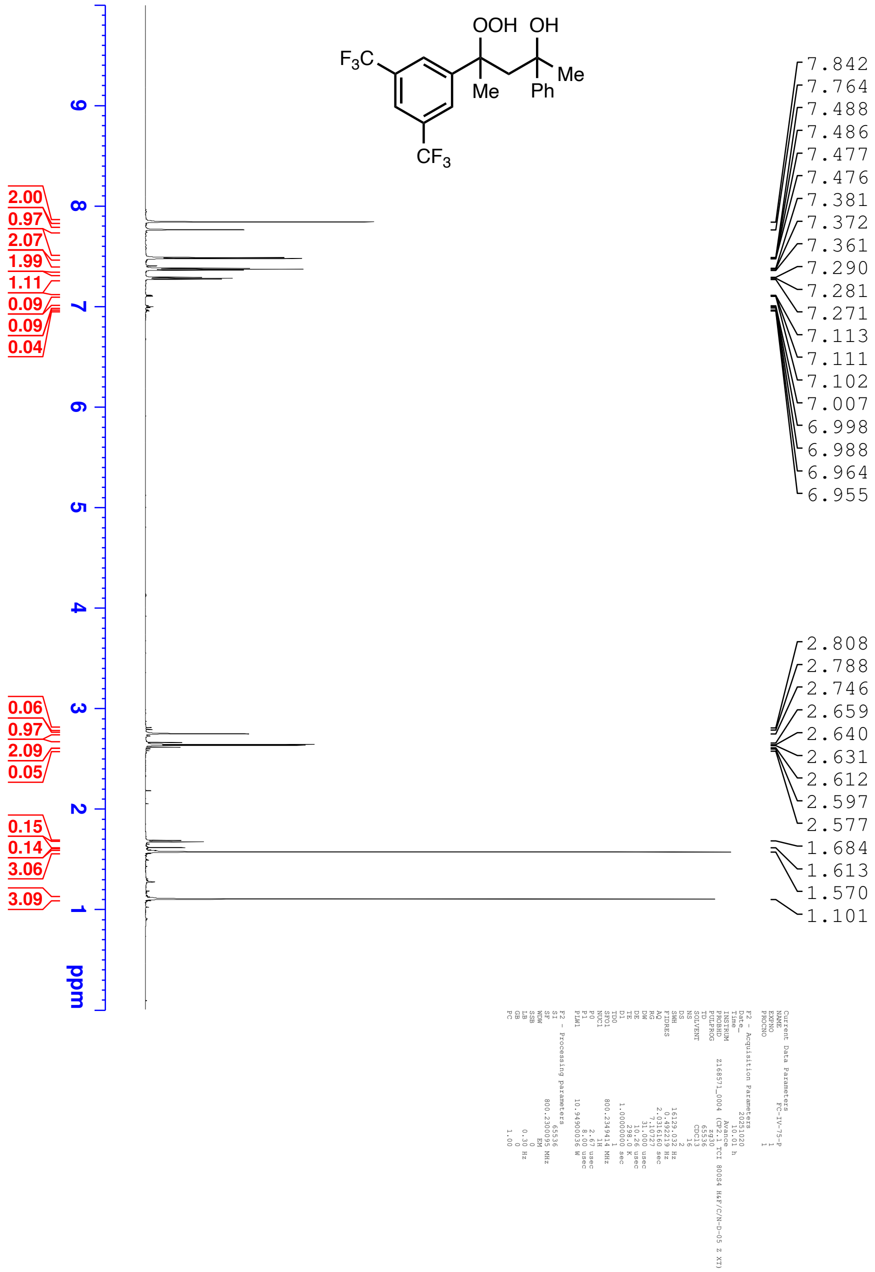

4-(3,5-Bis(trifluoromethyl)phenyl)-4-hydroperoxy-2-phenylpentan-2-ol (23).

Solvent: $CDCl_3$
100 MHz
$^{13}C\{^{1}H\}$ NMR

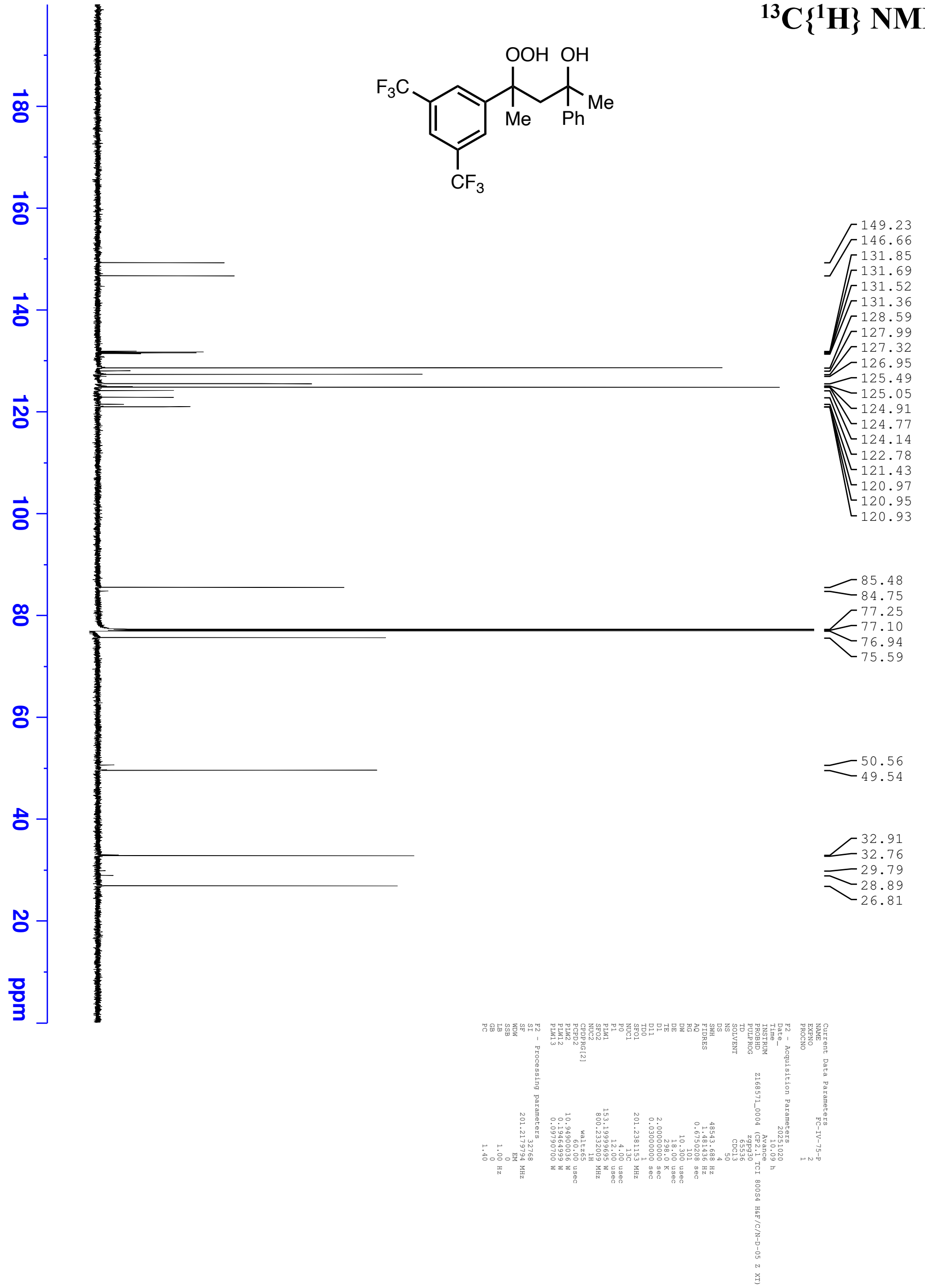

4-(3,5-Bis(trifluoromethyl)phenyl)-4-hydroperoxy-2-phenylpentan-2-ol (23).

Solvent: $CDCl_3$
400 MHz
^{1}H NMR

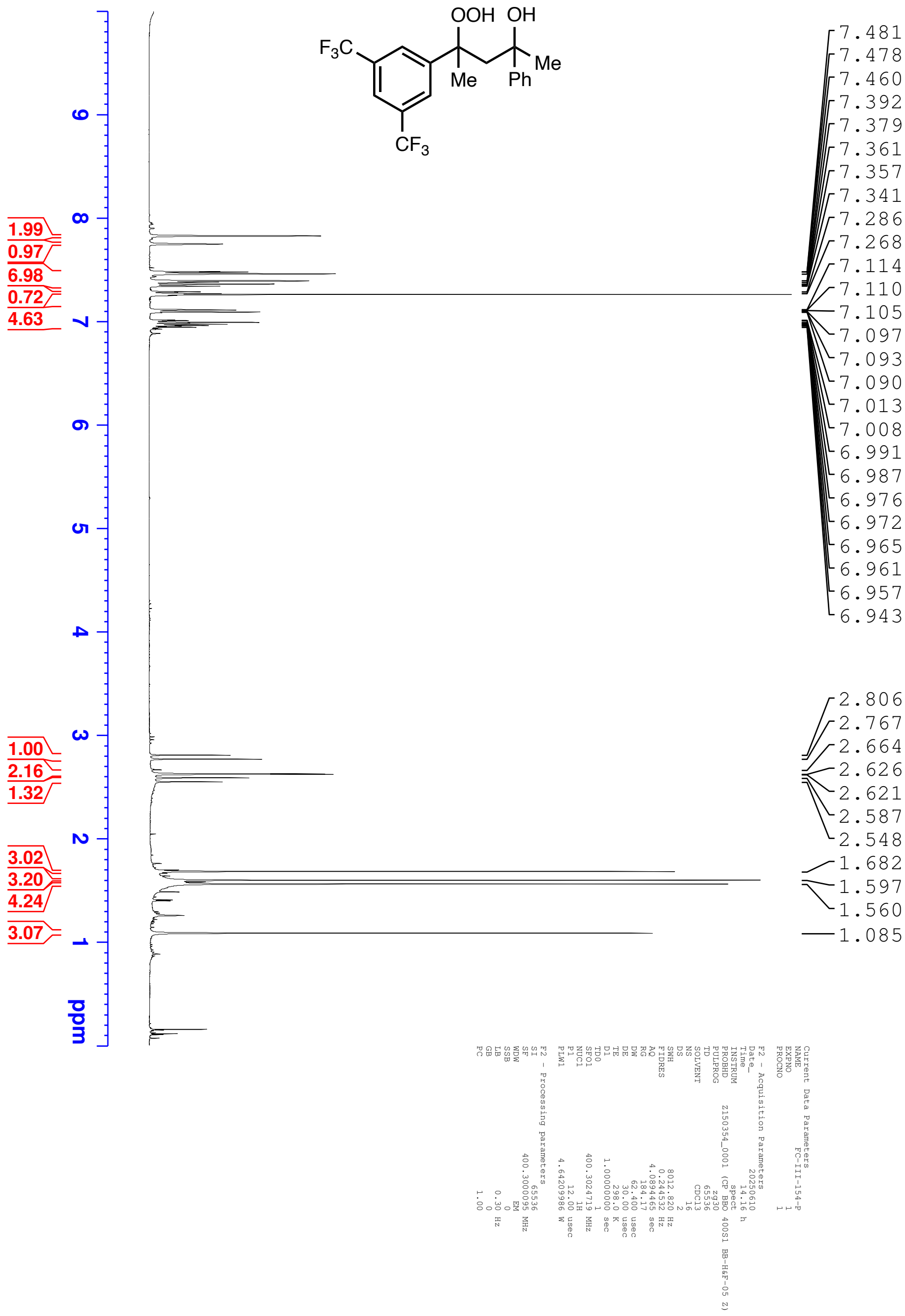

4-(3,5-Bis(trifluoromethyl)phenyl)-4-hydroperoxy-2-phenylpentan-2-ol (23).

Solvent: $CDCl_3$
100 MHz
$^{13}C\{^1H\}$ NMR

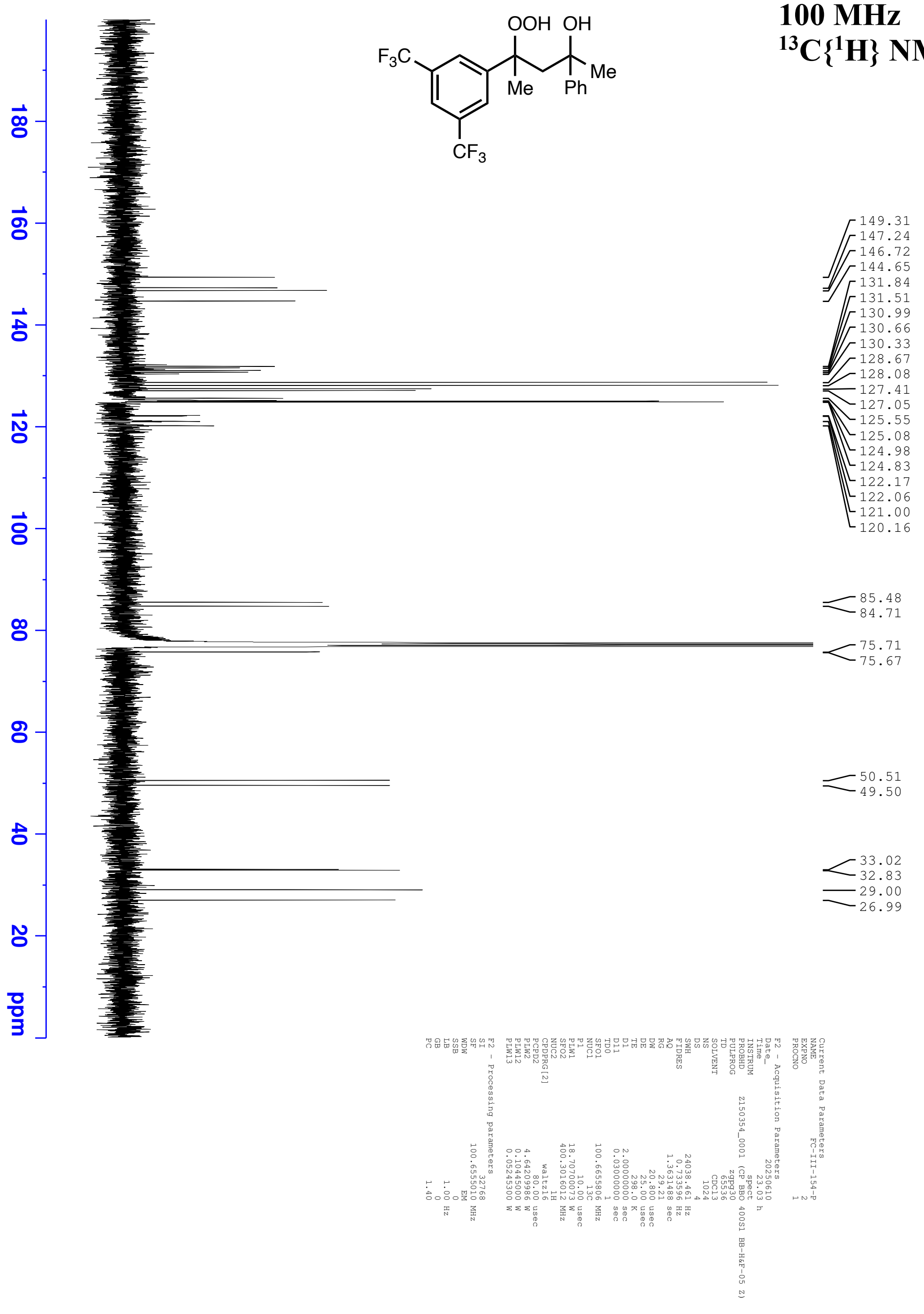

4-(3,5-Bis(trifluoromethyl)phenyl)-4-hydroperoxy-2-phenylpentan-2-ol (23).

Solvent: $CDCl_3$
377 MHz
$^{19}F\{^{1}H\}$ NMR

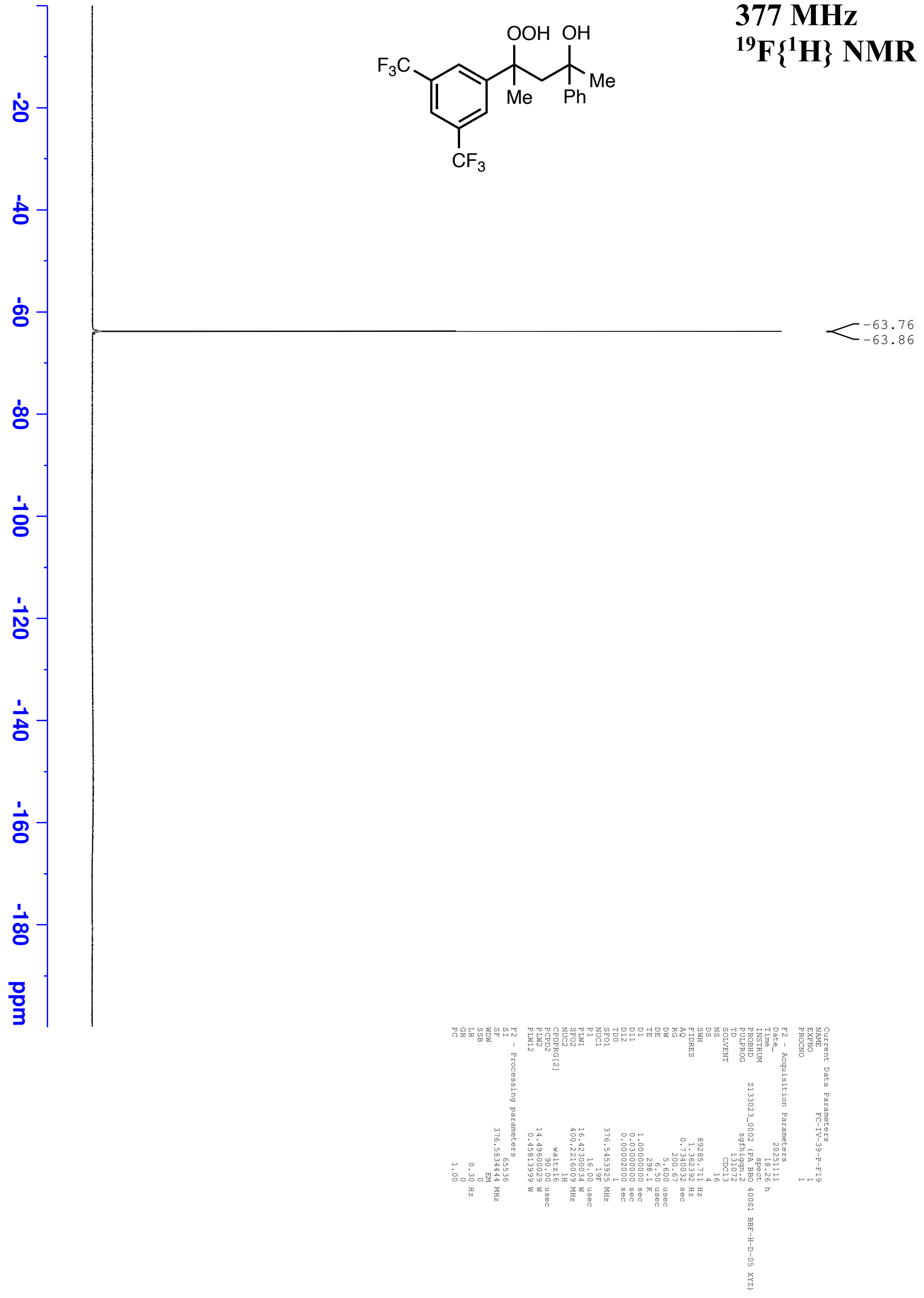

3-(3,5-Bis(trifluoromethyl)phenyl)-3,5-dimethyl-5-phenyl-1,2-dioxolane (24).

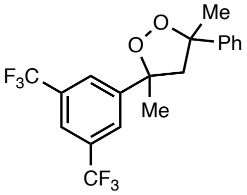

Solvent: $CDCl_3$
400 MHz
^{1}H NMR

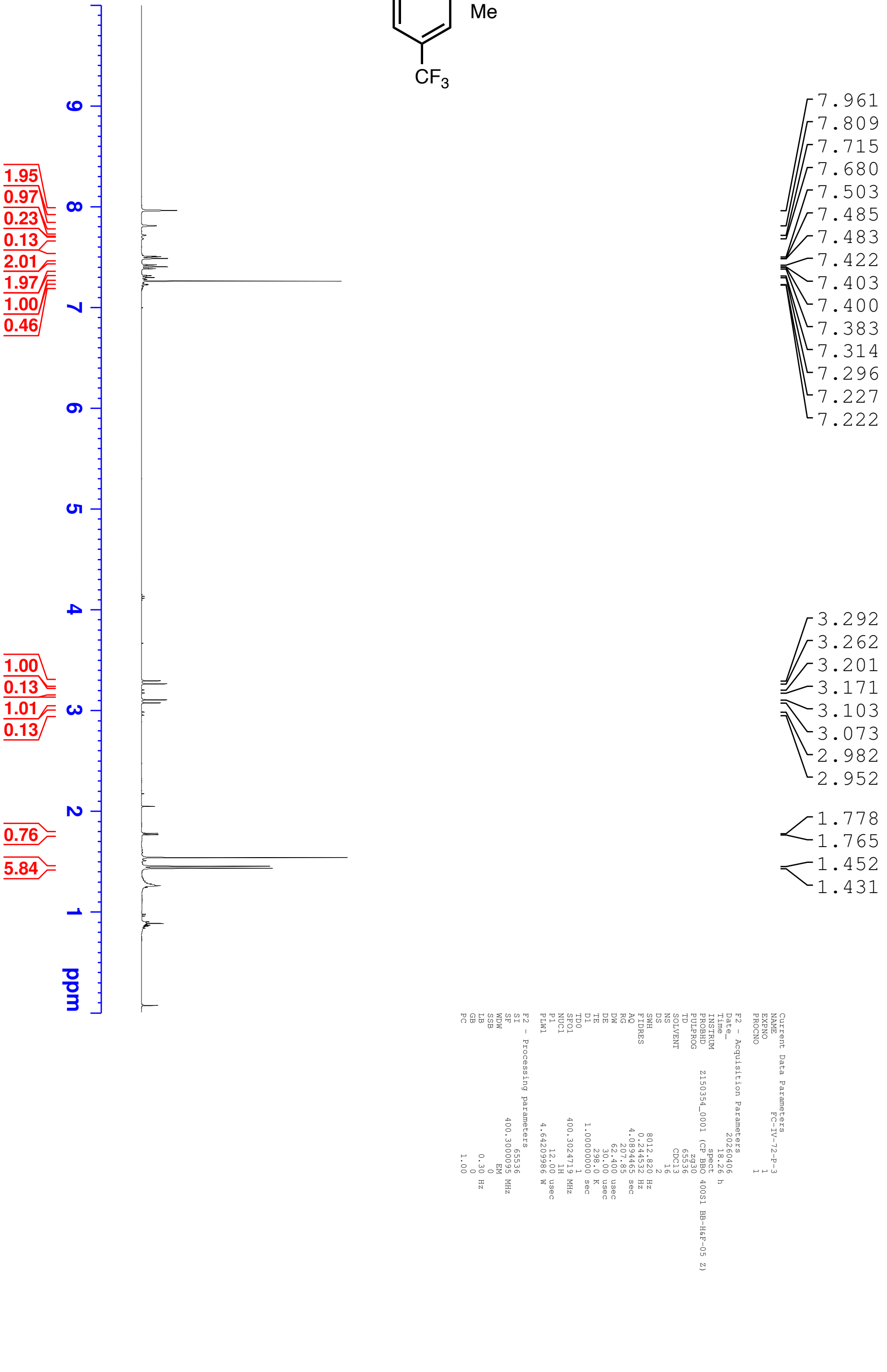

3-(3,5-Bis(trifluoromethyl)phenyl)-3,5-dimethyl-5-phenyl-1,2-dioxolane (24).

Solvent: $CDCl_3$
100 MHz
$^{13}C\{^1H\}$ NMR

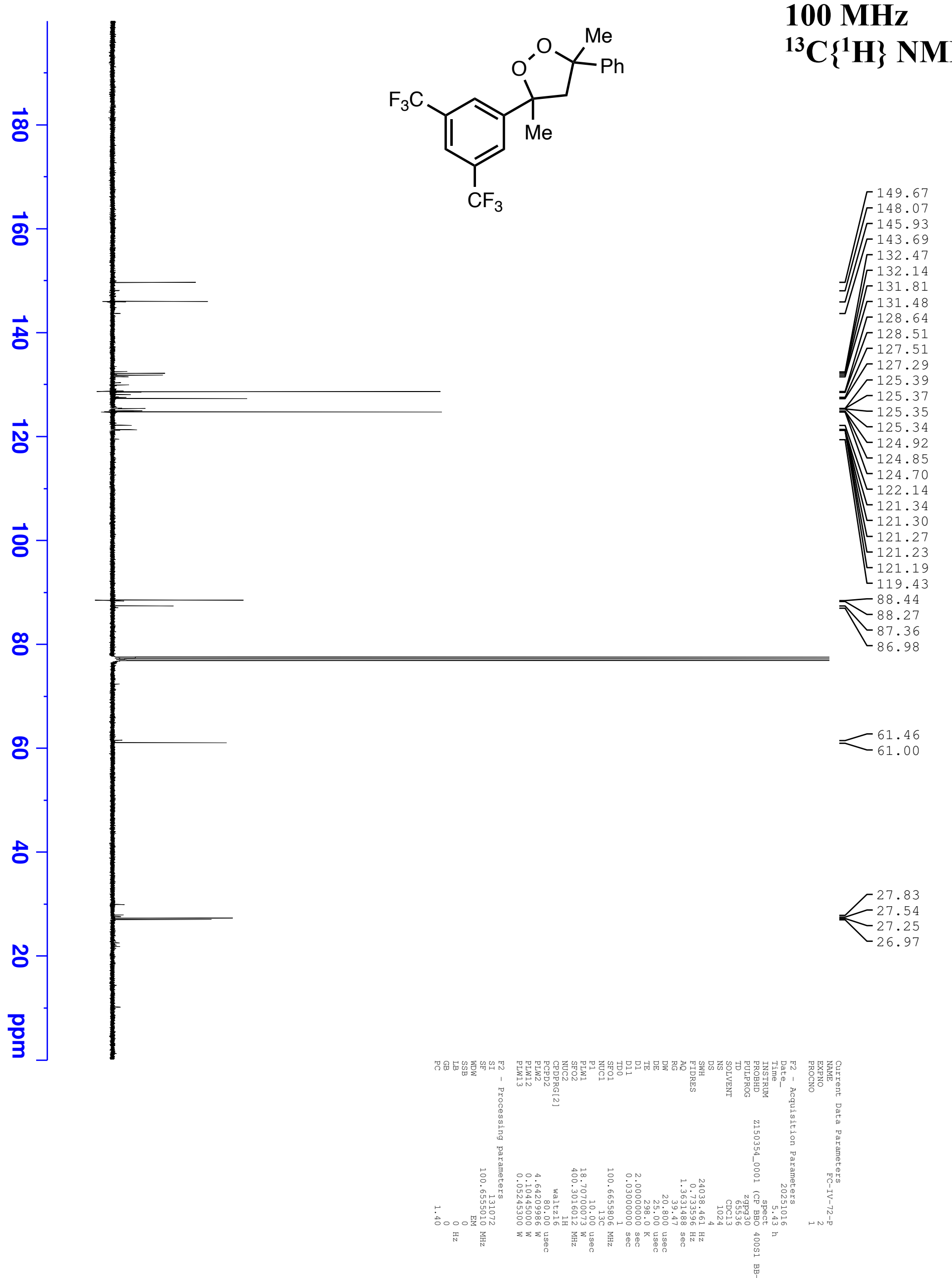

3-(3,5-Bis(trifluoromethyl)phenyl)-3,5-dimethyl-5-phenyl-1,2-dioxolane (24).

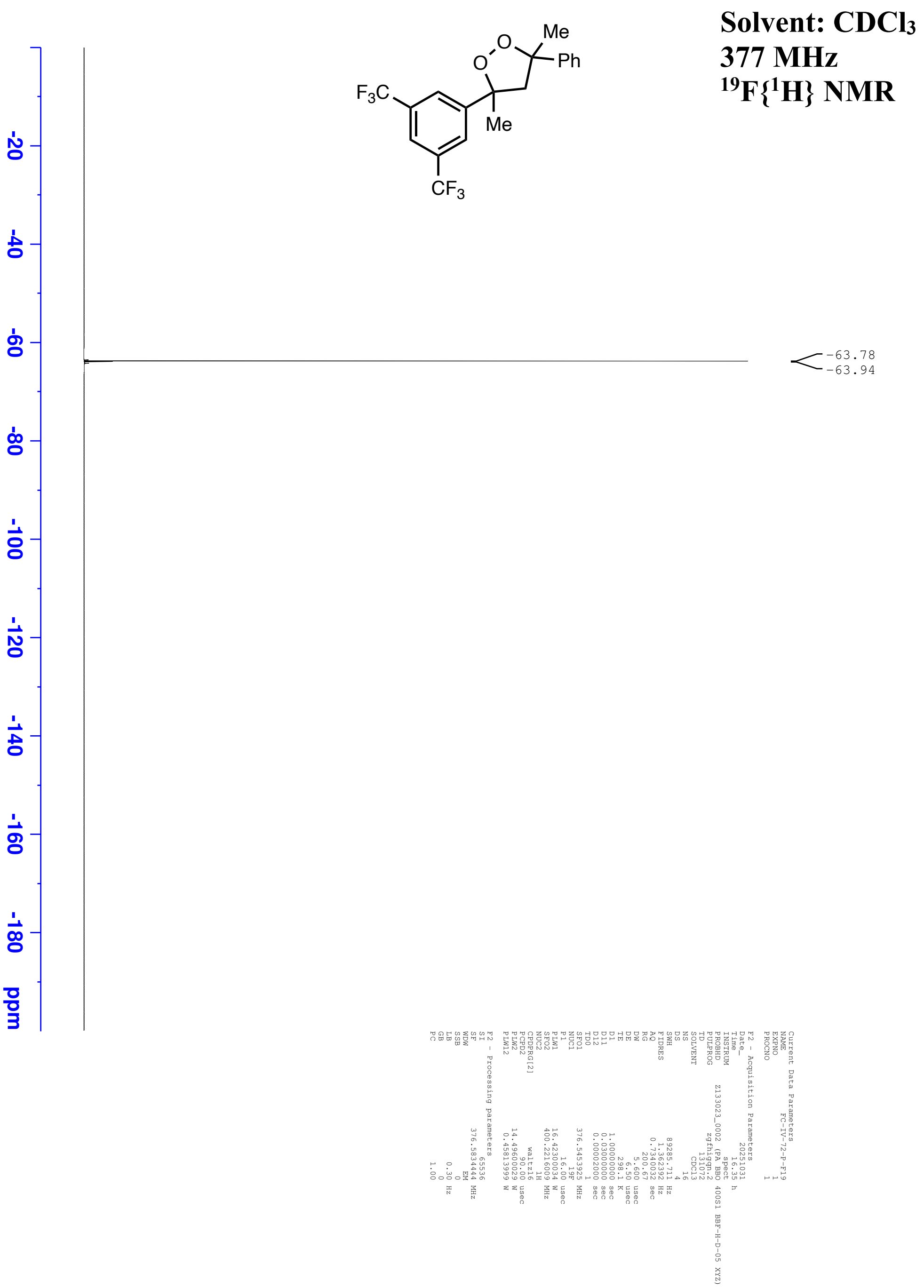

3-(3,5-Bis(trifluoromethyl)phenyl)-3,5-dimethyl-5-phenyl-1,2-dioxolane (24).

Solvent: C_6D_6
400 MHz
^{1}H NMR

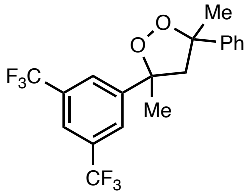

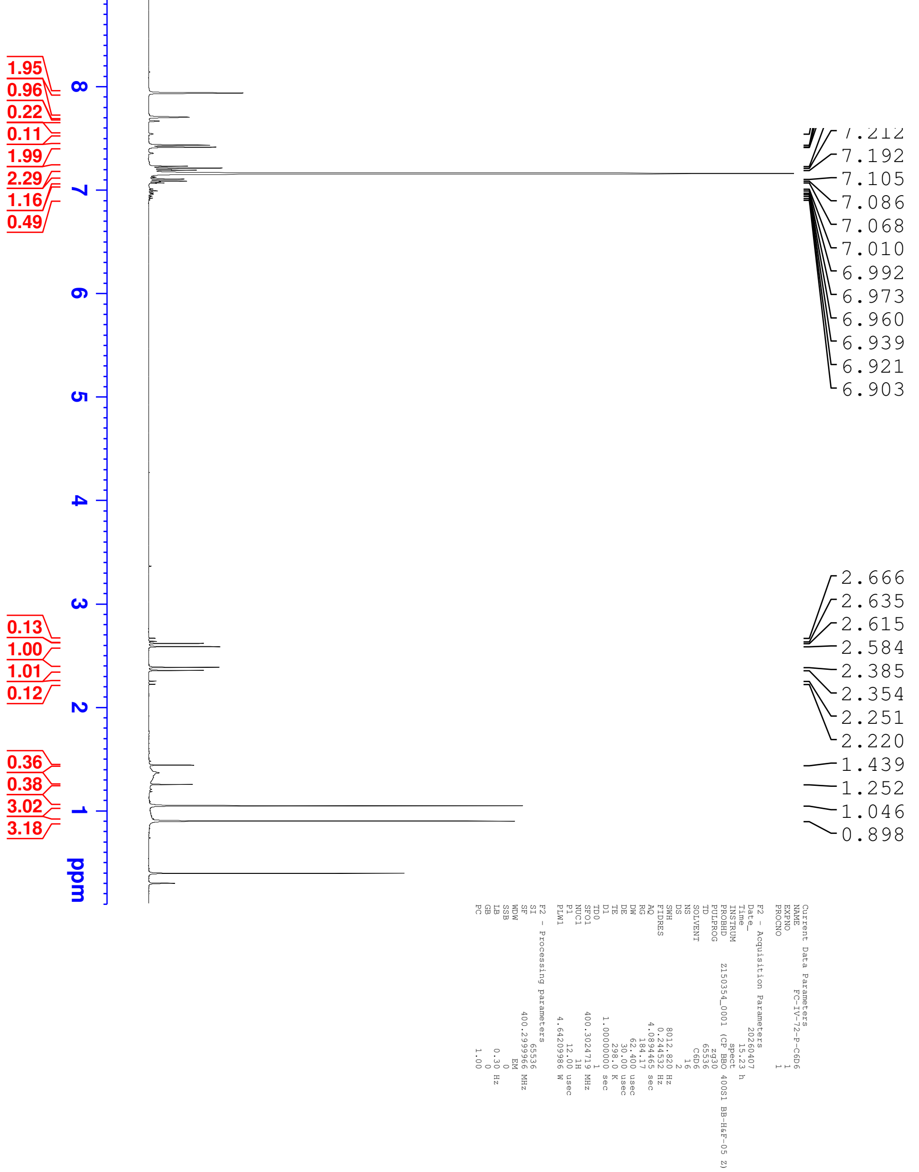

3-(3,5-Bis(trifluoromethyl)phenyl)-3,5-dimethyl-5-phenyl-1,2-dioxolane (24).

Solvent: C_6D_6
100 MHz
$^{13}C\{^1H\}$ NMR

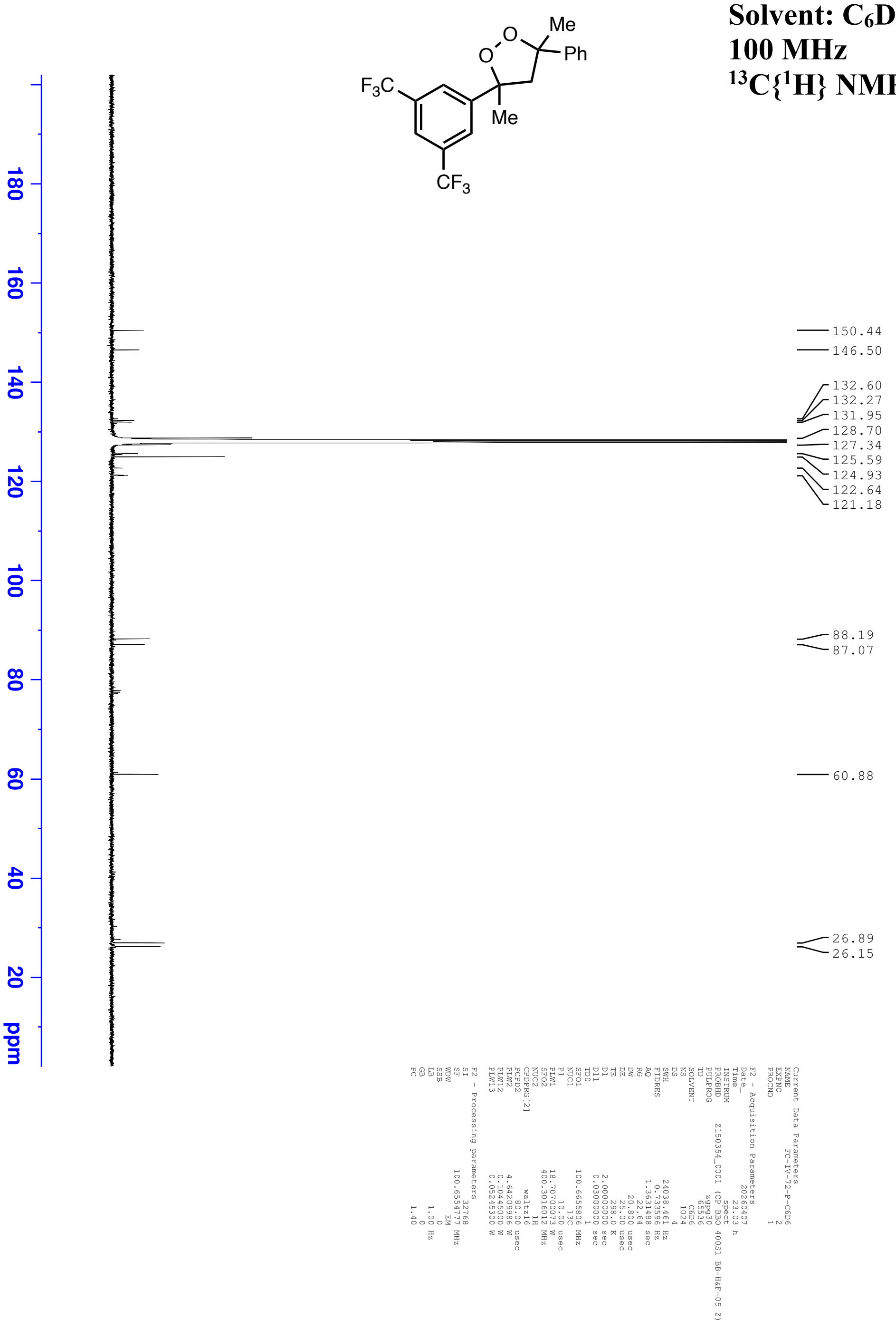

3-(3,5-Bis(trifluoromethyl)phenyl)-3,5-dimethyl-5-phenyl-1,2-dioxolane (24).

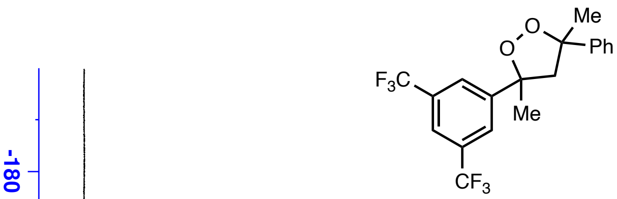

Solvent: C_6D_6
377 MHz
$^{19}F\{^1H\}$ NMR

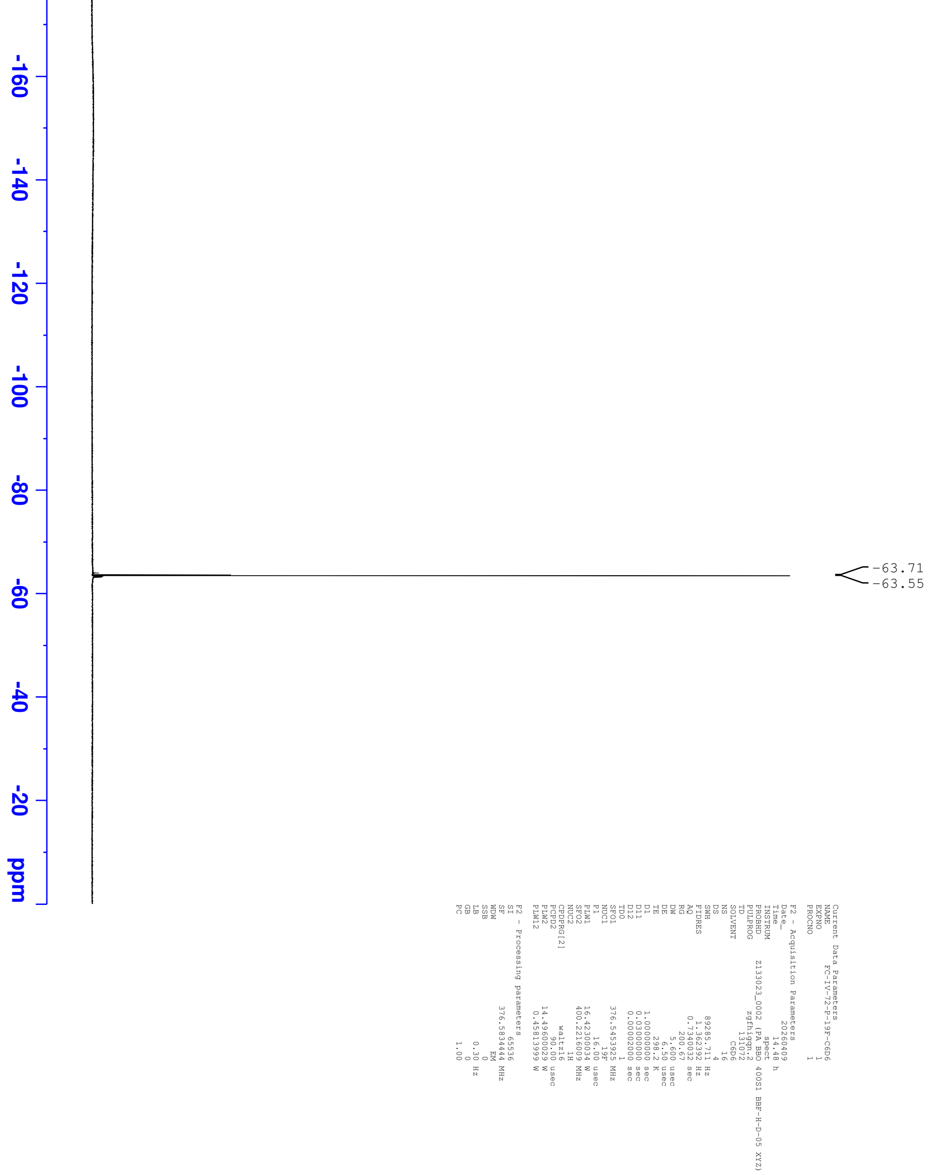

3-(3,5-Bis(trifluoromethyl)phenyl)-3,5-dimethyl-5-phenyl-1,2-dioxolane (24).

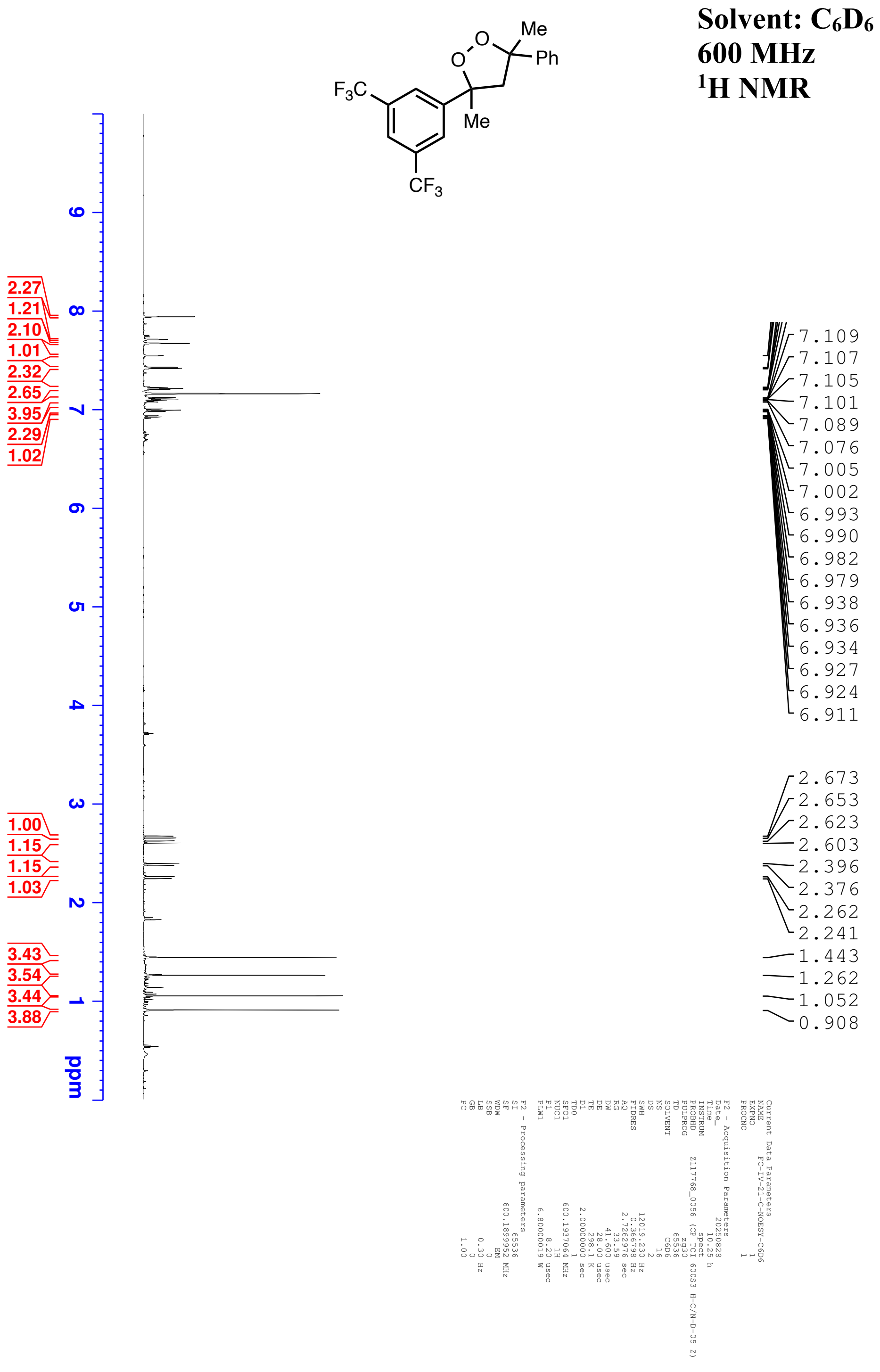

The ^{1}H NMR spectrum above was used for the following NOESY studies.

3-(3,5-Bis(trifluoromethyl)phenyl)-3,5-dimethyl-5-phenyl-1,2-dioxolane (24).

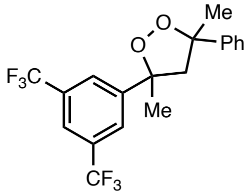

Solvent: C_6D_6
600 MHz
^{1}H NMR

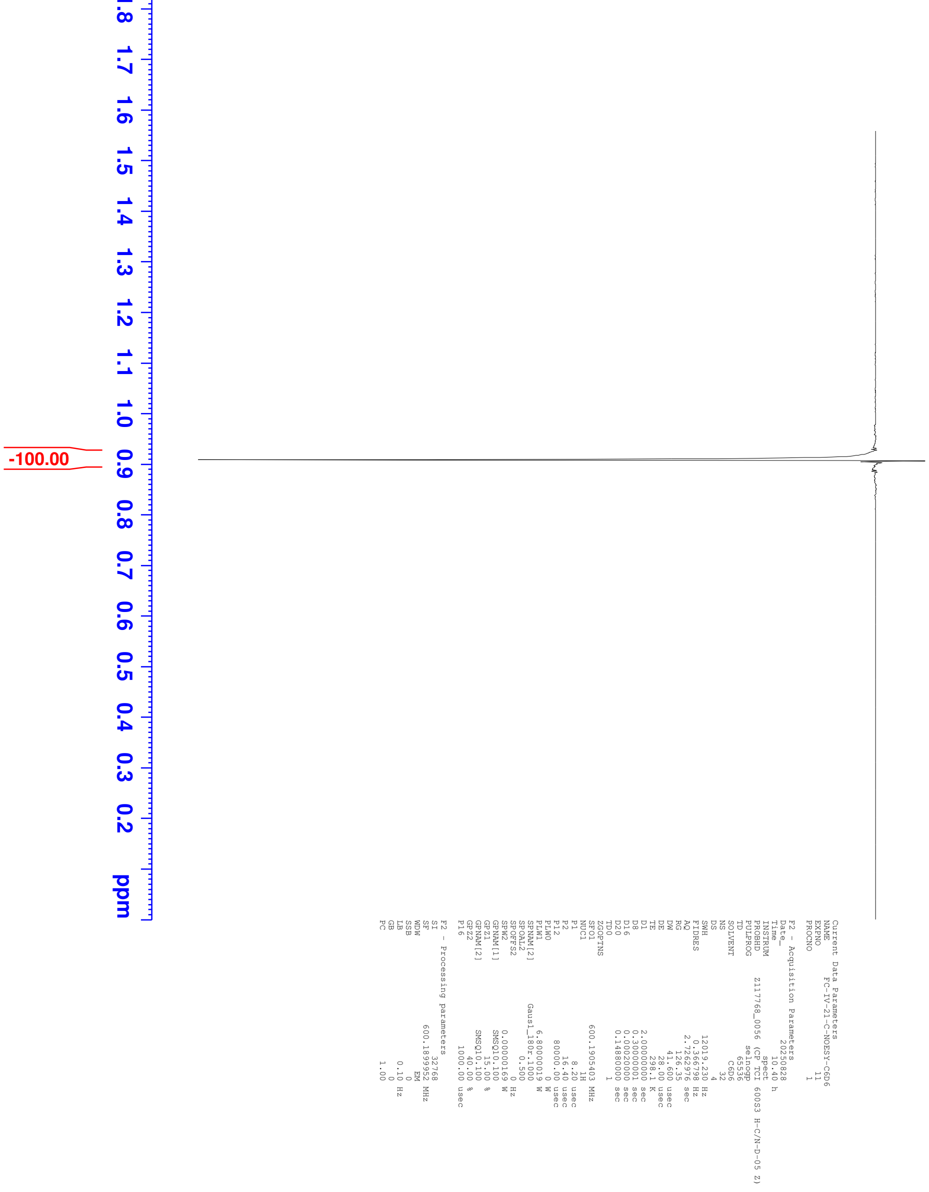

Irradiation of the *trans* methyl peak at δ 0.90 ppm led to no NOE signal at the other *trans* methyl peak at δ 1.05 ppm.

3-(3,5-Bis(trifluoromethyl)phenyl)-3,5-dimethyl-5-phenyl-1,2-dioxolane (24).

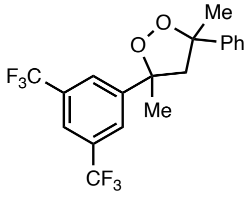

Solvent: C_6D_6
600 MHz
^{1}H NMR

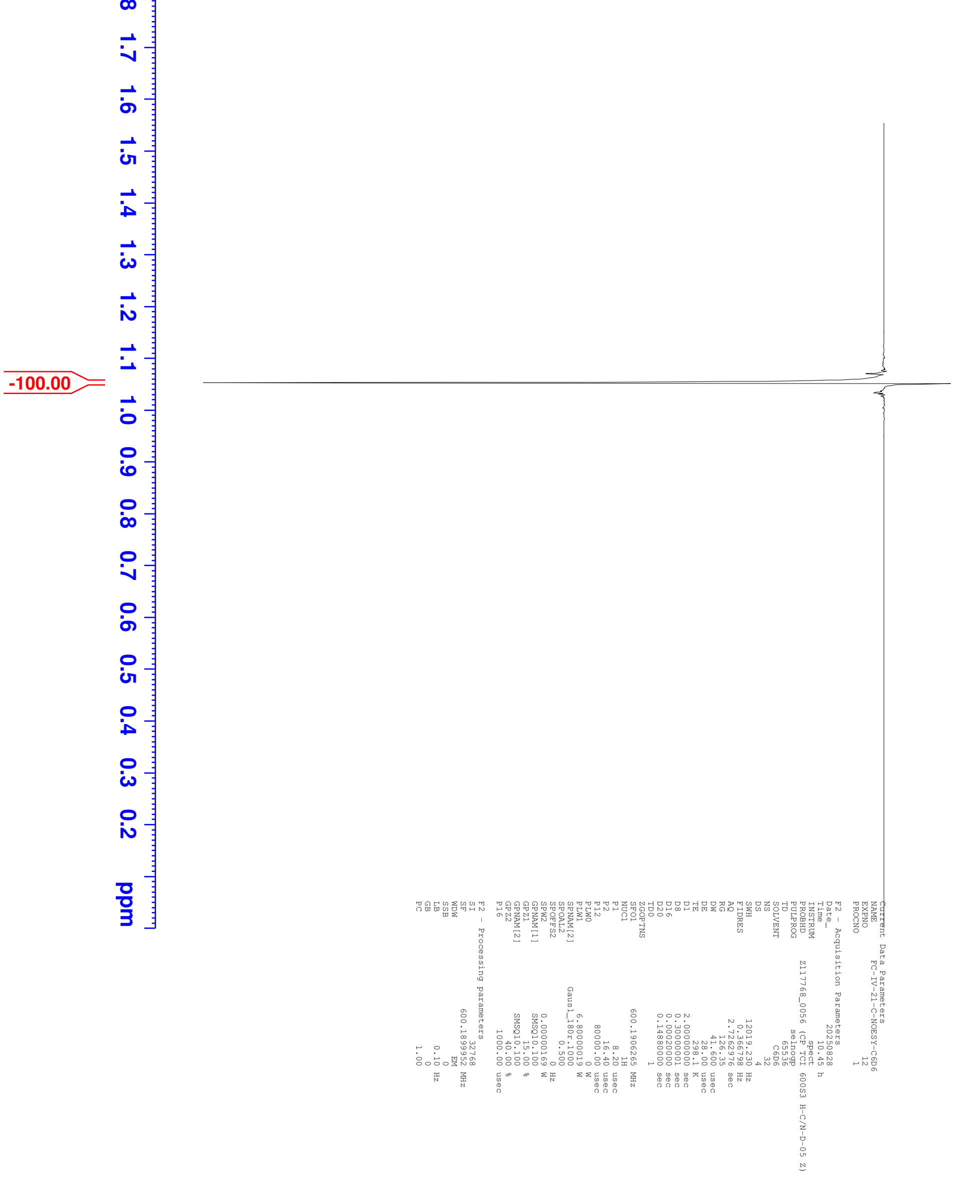

Irradiation of the *trans* methyl peak at δ 1.05 ppm led to no NOE signal at the other *trans* methyl peak at δ 0.90 ppm.

3-(3,5-Bis(trifluoromethyl)phenyl)-3,5-dimethyl-5-phenyl-1,2-dioxolane (24).

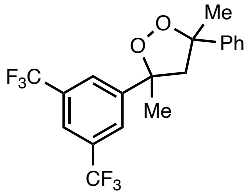

Solvent: C_6D_6
600 MHz
^{1}H NMR

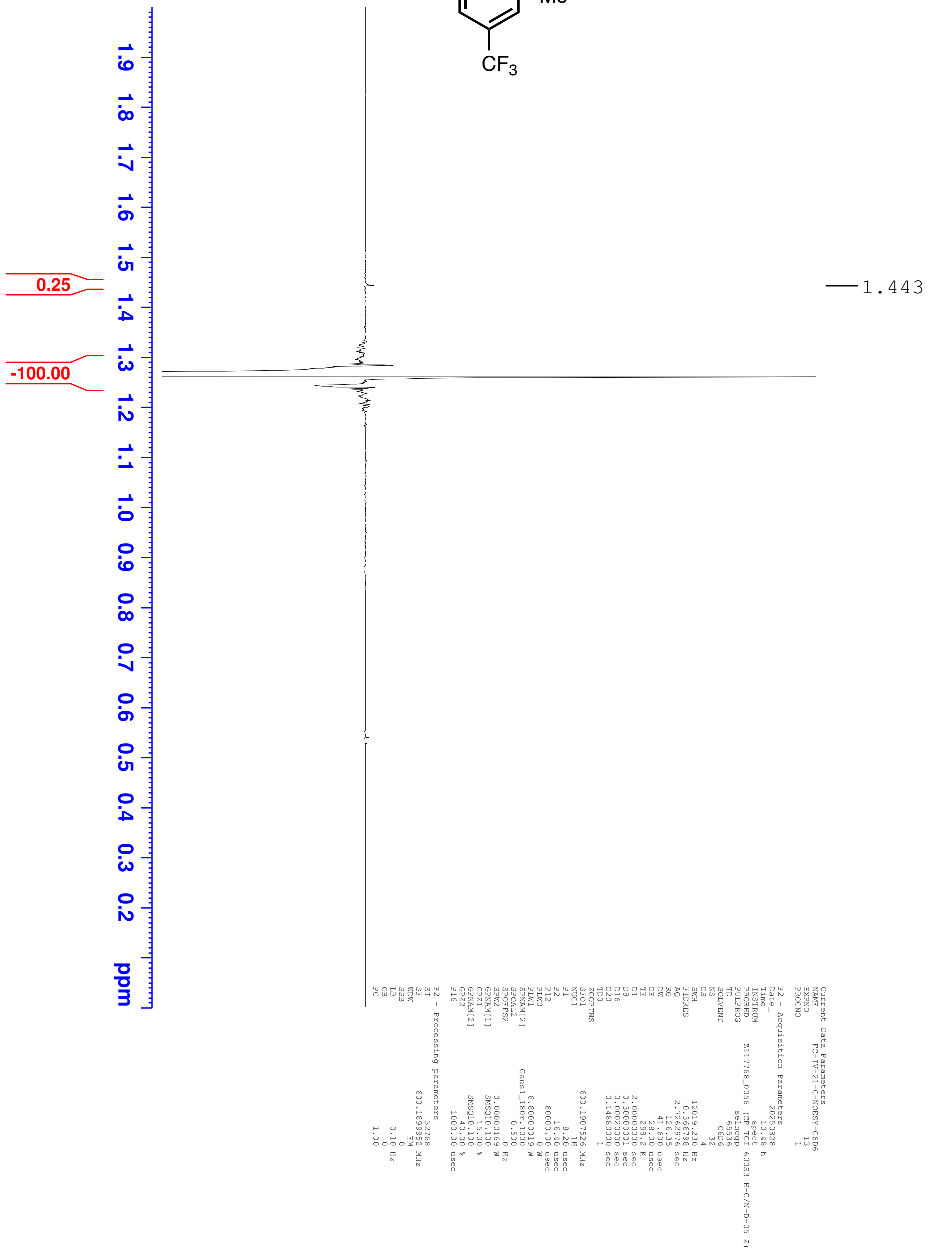

Irradiation of the *cis* methyl peak at δ 1.26 ppm led to a NOE signal at the other *cis* methyl peak at δ 1.44 ppm.

3-(3,5-Bis(trifluoromethyl)phenyl)-3,5-dimethyl-5-phenyl-1,2-dioxolane (24).

Solvent: C_6D_6
600 MHz
^{1}H NMR

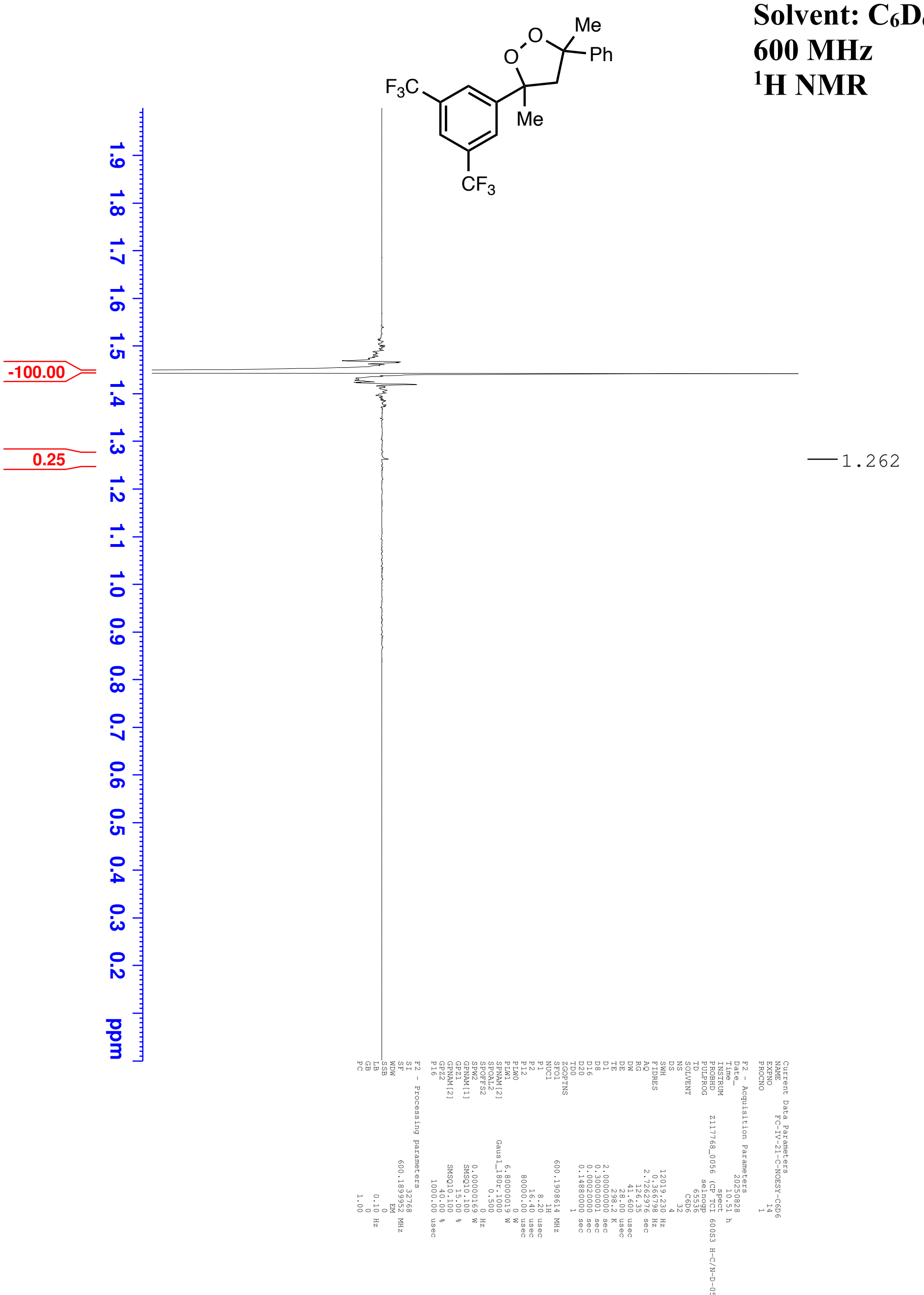

Irradiation of the *cis* methyl peak at δ 1.44 ppm led to a NOE signal at the other *cis* methyl peak at δ 1.26 ppm.

2-(3,5-Bis(trifluoromethyl)phenyl)-4-phenylpentane-2,4-diol (25).

Solvent: $CDCl_3$
400 MHz
^{1}H NMR

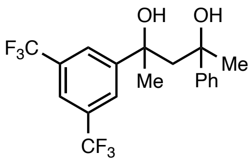

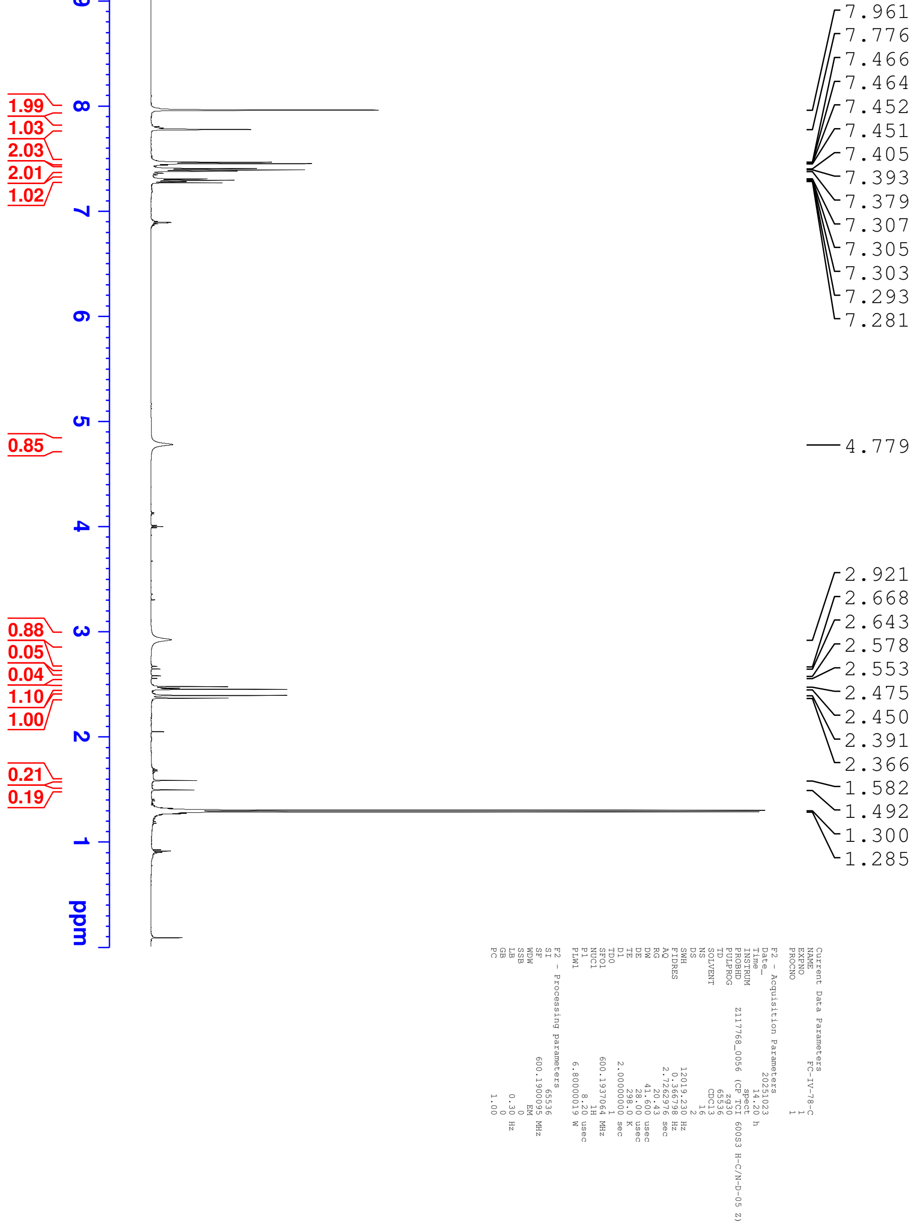

2-(3,5-Bis(trifluoromethyl)phenyl)-4-phenylpentane-2,4-diol (25).

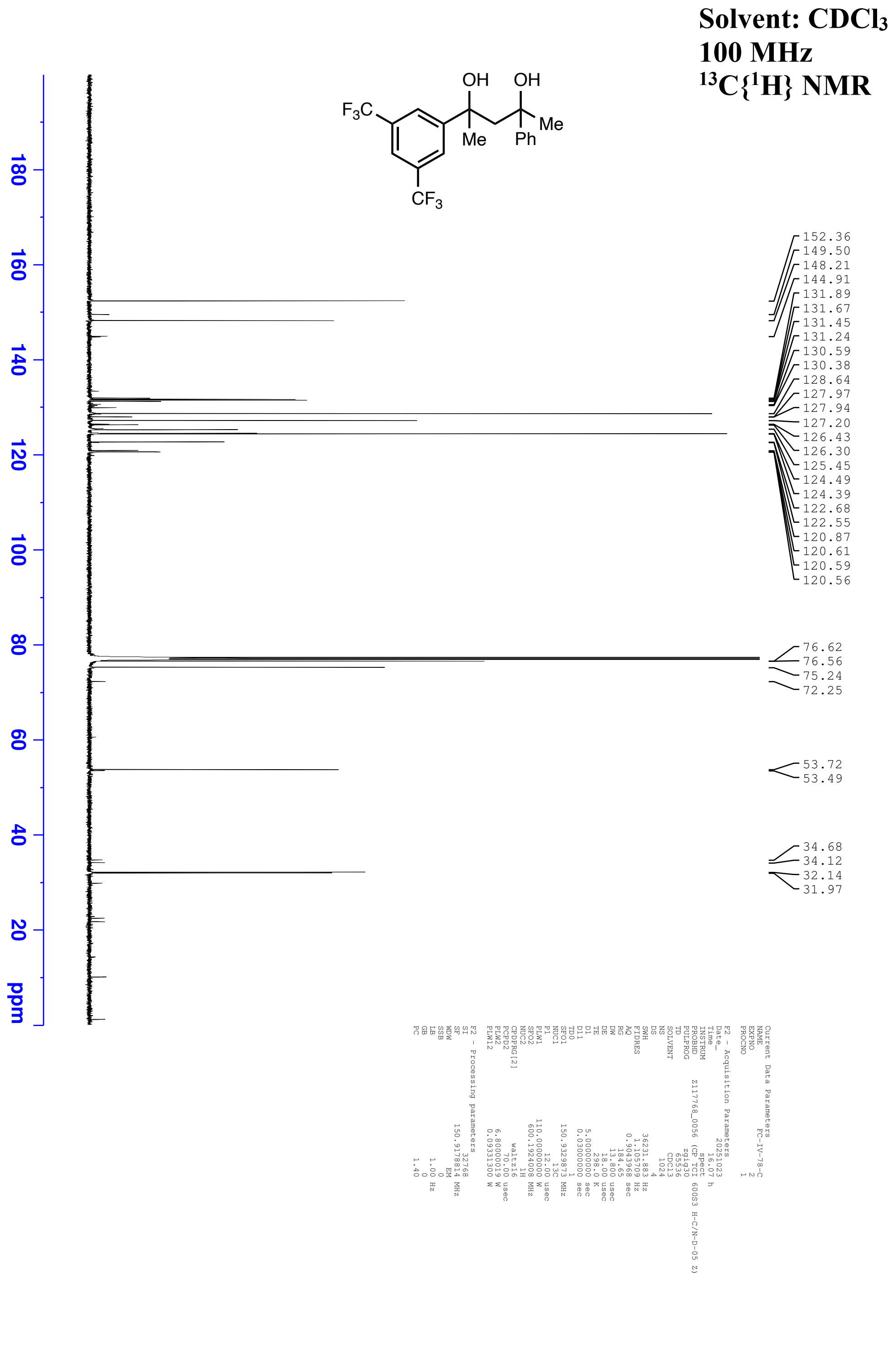

2-(3,5-Bis(trifluoromethyl)phenyl)-4-phenylpentane-2,4-diol (25).

Solvent: $CDCl_3$
377 MHz
$^{19}F\{^{1}H\}$ NMR

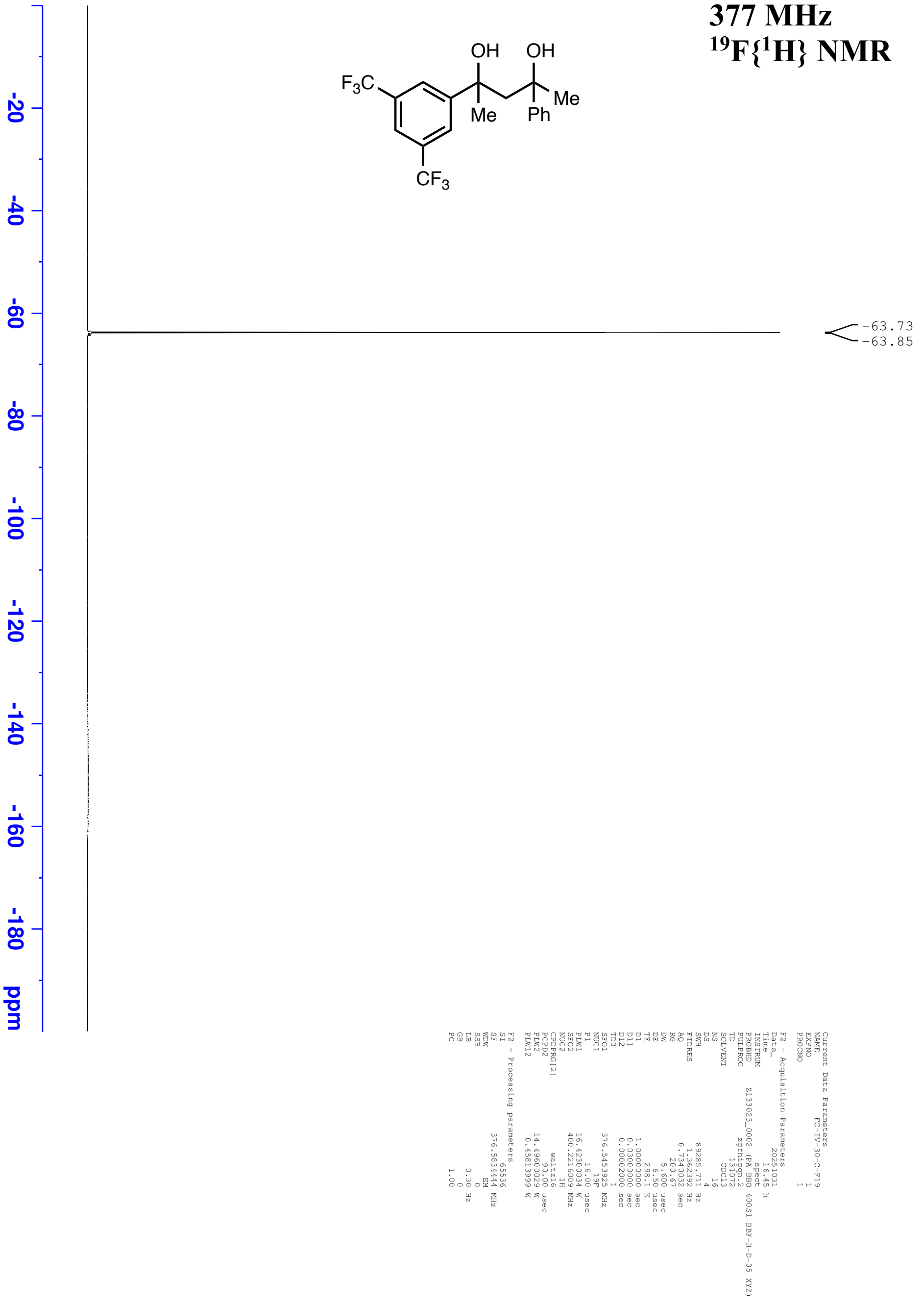

3-(3,5-Bis(trifluoromethyl)phenyl)-3,5,5-trimethyl-1,2-dioxolane (28).

Solvent: $CDCl_3$
400 MHz
^{1}H NMR

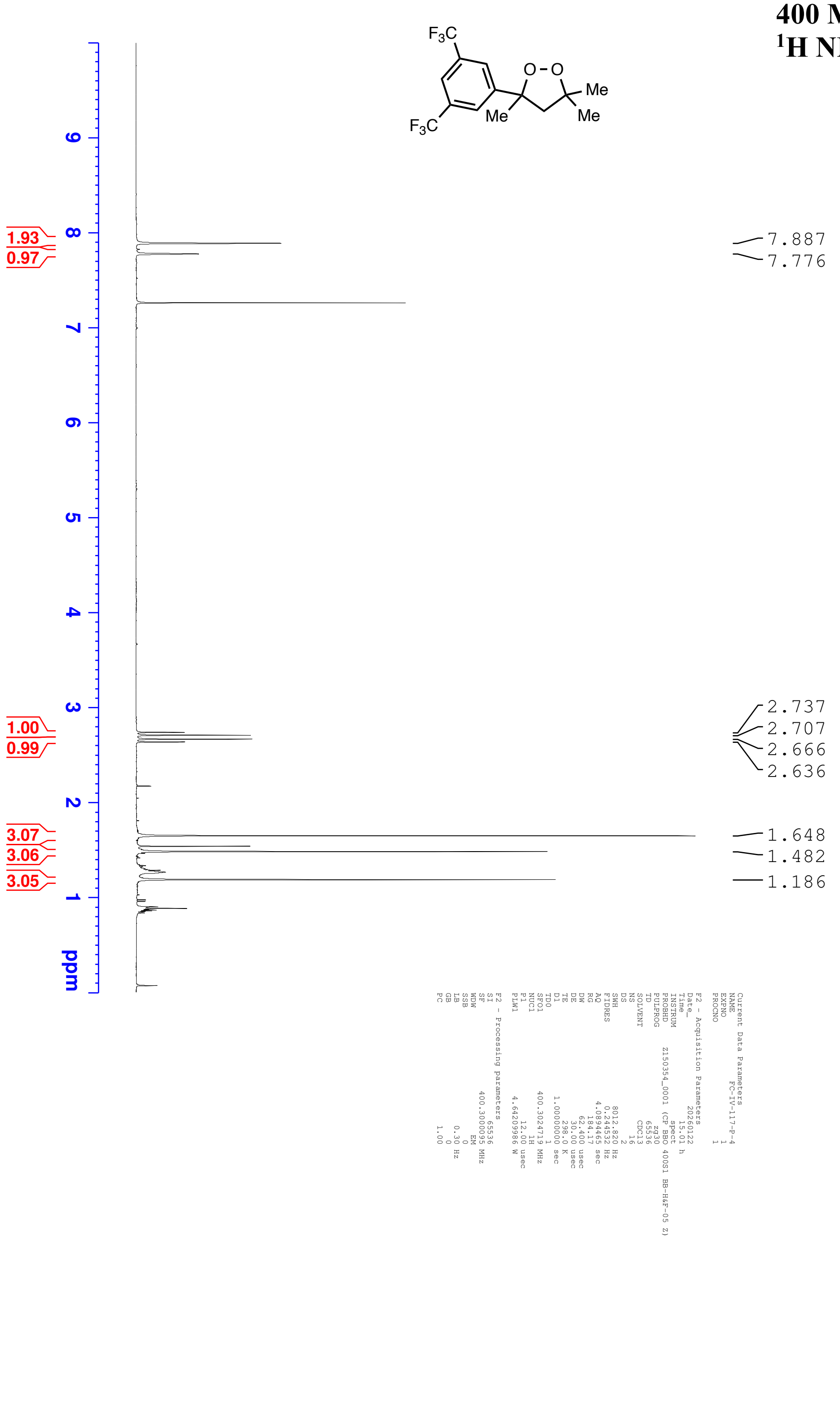

3-(3,5-Bis(trifluoromethyl)phenyl)-3,5,5-trimethyl-1,2-dioxolane (28).

Solvent: $CDCl_3$
100 MHz
$^{13}C\{^1H\}$ NMR

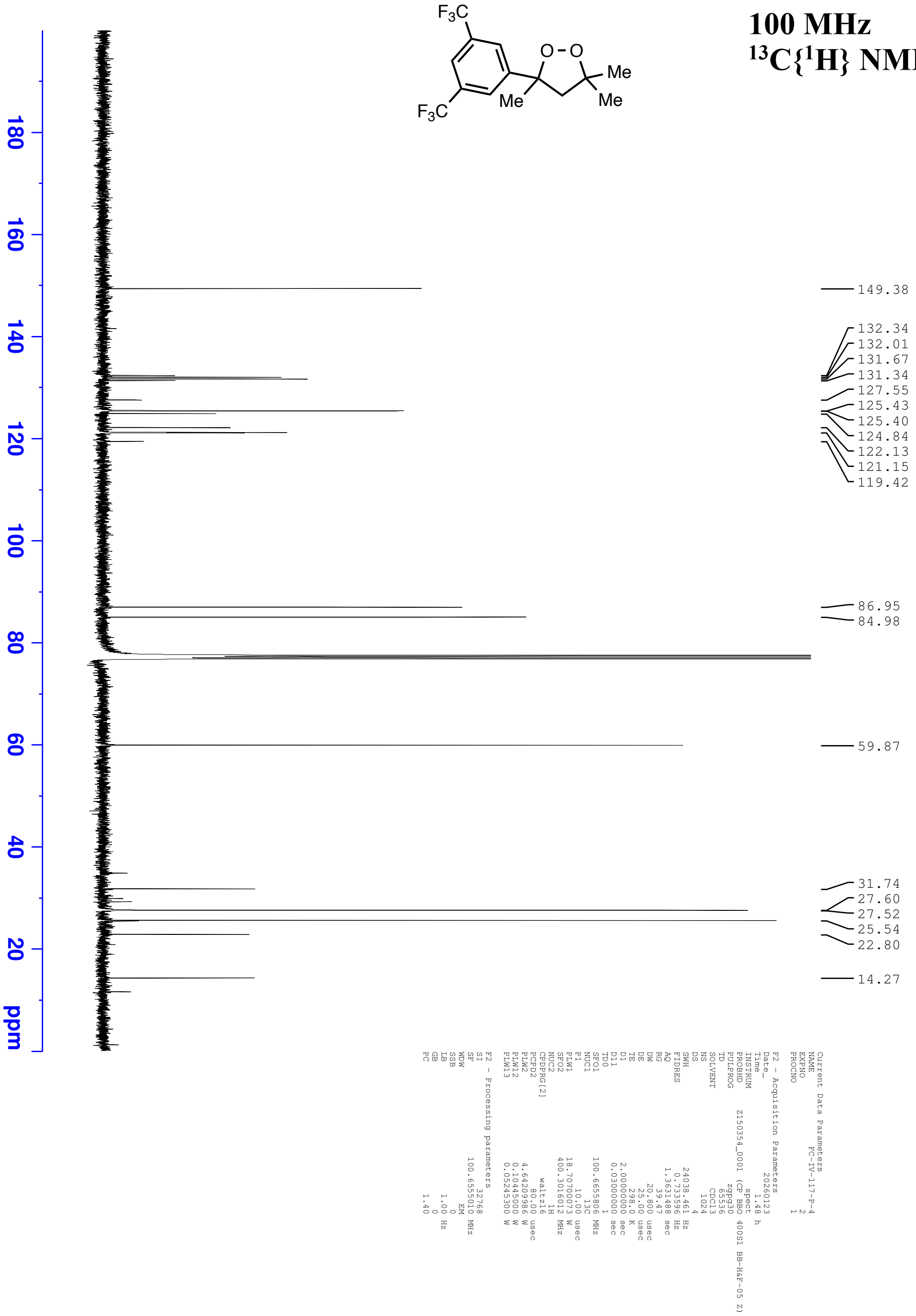

3-(3,5-Bis(trifluoromethyl)phenyl)-3,5,5-trimethyl-1,2-dioxolane (28).

Solvent: $CDCl_3$
377 MHz
$^{19}F\{^1H\}$ NMR

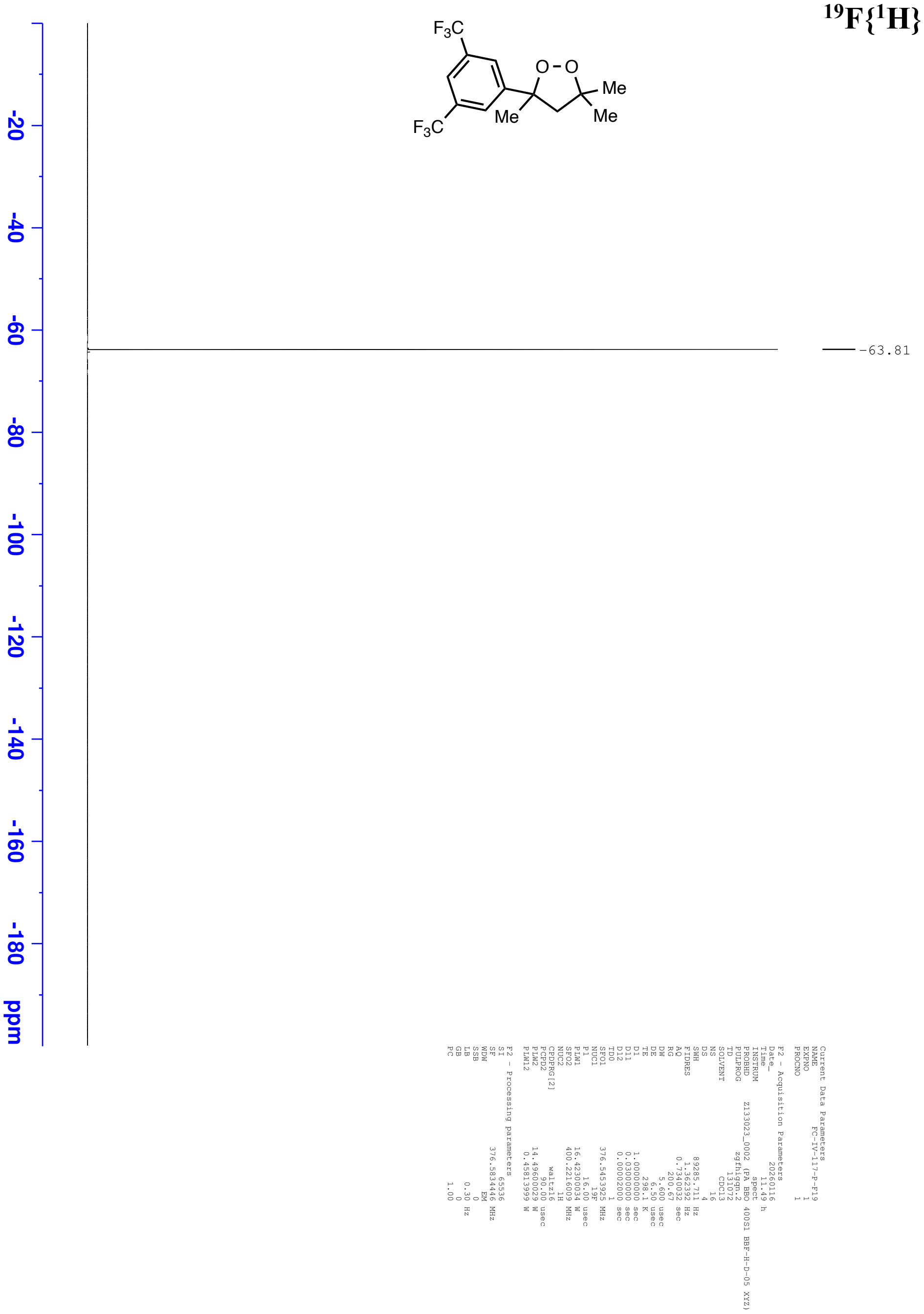

3-(3,5-Bis(trifluoromethyl)phenyl)-3-methyl-5,5-diphenyl-1,2-dioxolane (29).

Solvent: $CDCl_3$
400 MHz
^{1}H NMR

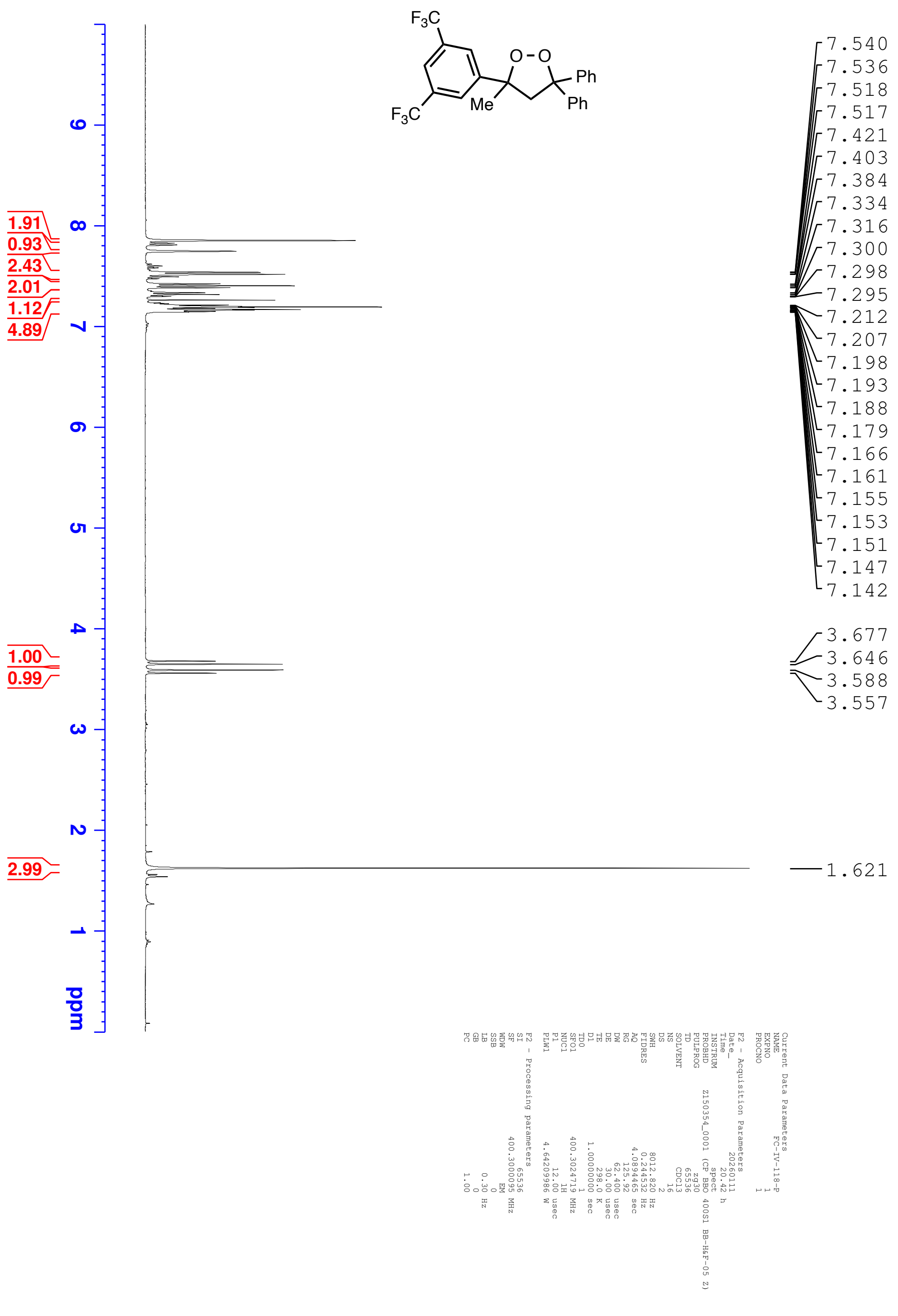

3-(3,5-Bis(trifluoromethyl)phenyl)-3-methyl-5,5-diphenyl-1,2-dioxolane (29).

Solvent: $CDCl_3$
100 MHz
$^{13}C\{^1H\}$ NMR

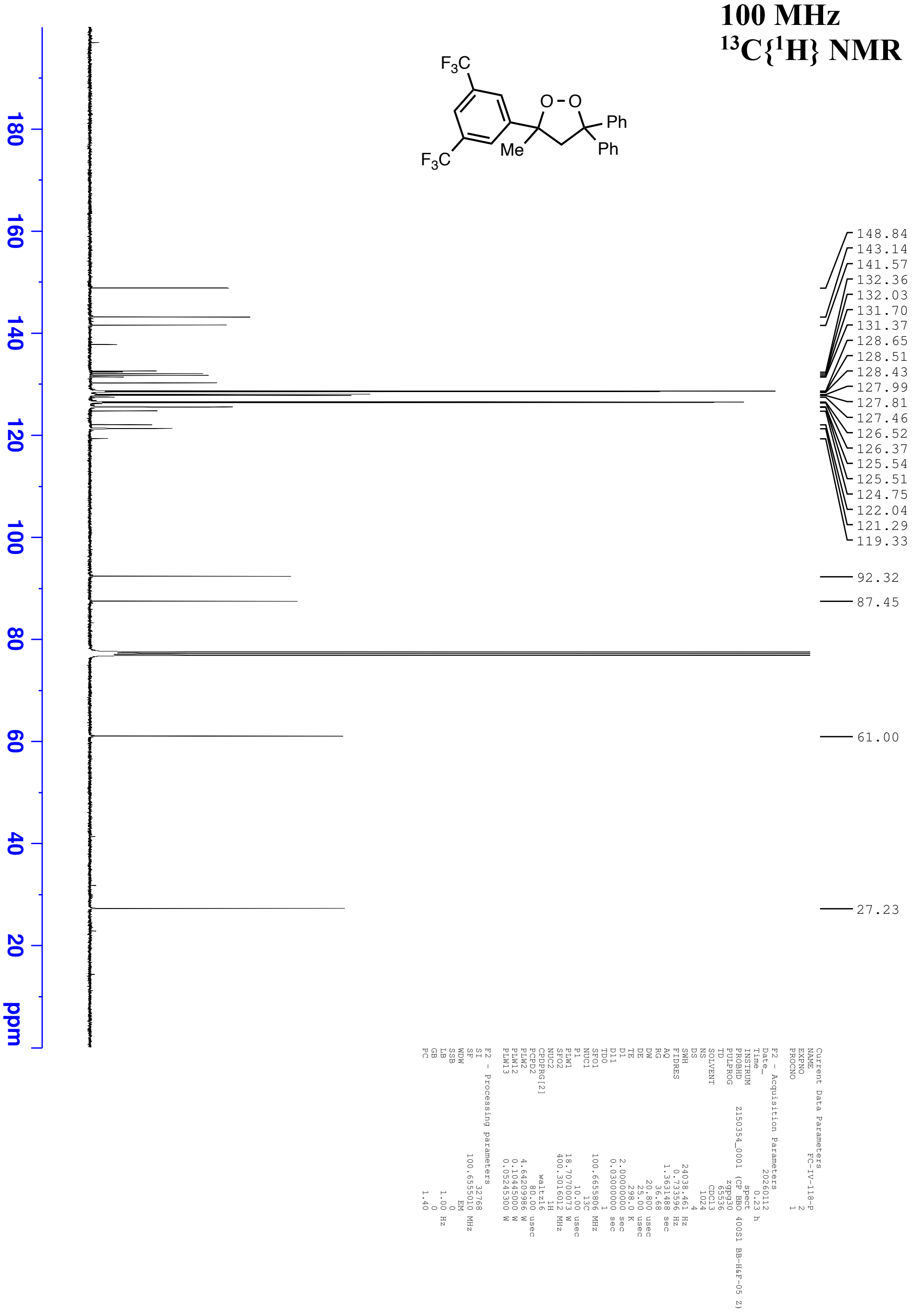

3-(3,5-Bis(trifluoromethyl)phenyl)-3-methyl-5,5-diphenyl-1,2-dioxolane (29).

Solvent: $CDCl_3$
377 MHz
$^{19}F\{^{1}H\}$ NMR

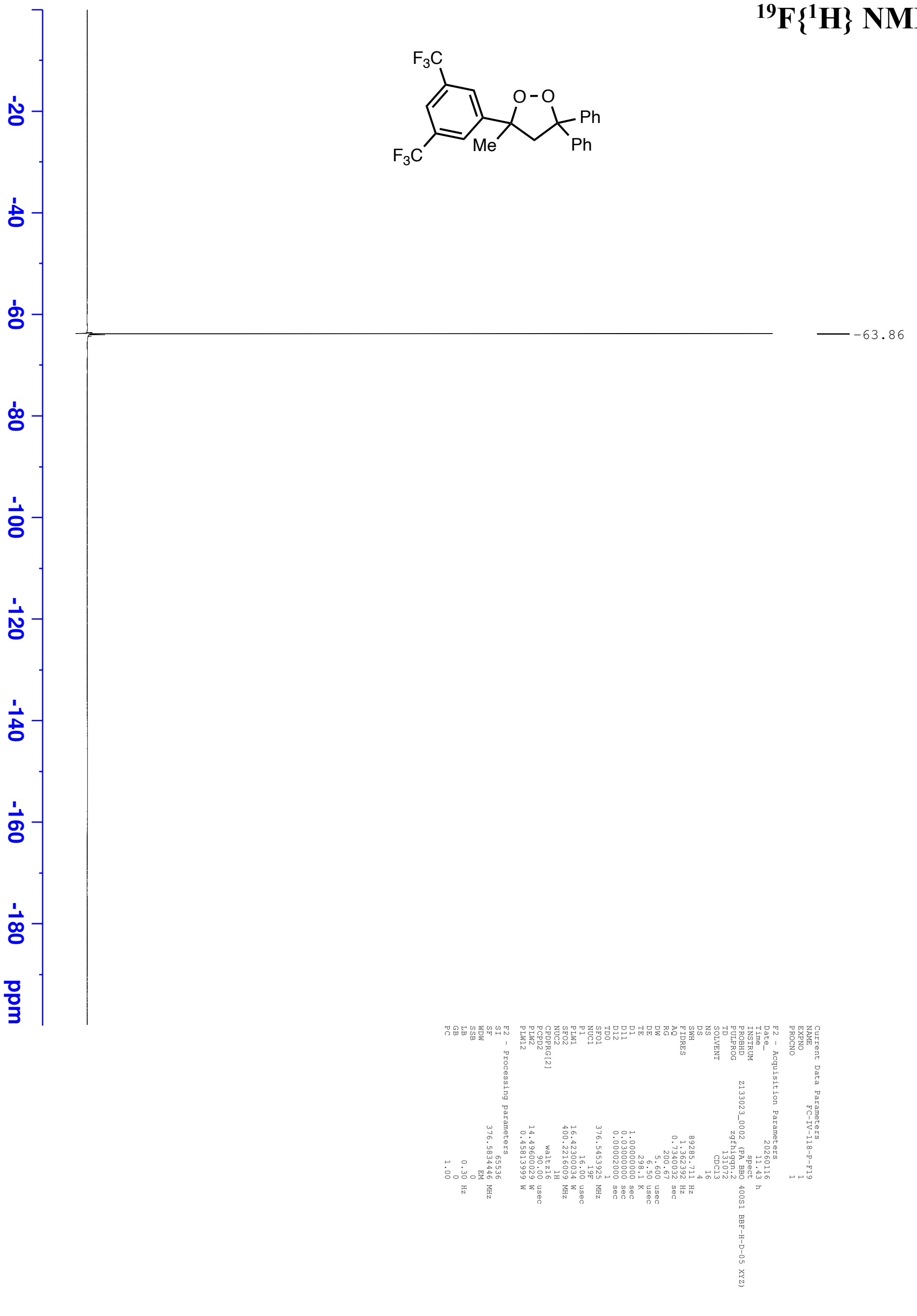

3-([1,1'-Biphenyl]-4-yl)-3,5,5-trimethyl-1,2-dioxolane (30).

Solvent: $CDCl_3$
400 MHz
^{1}H NMR

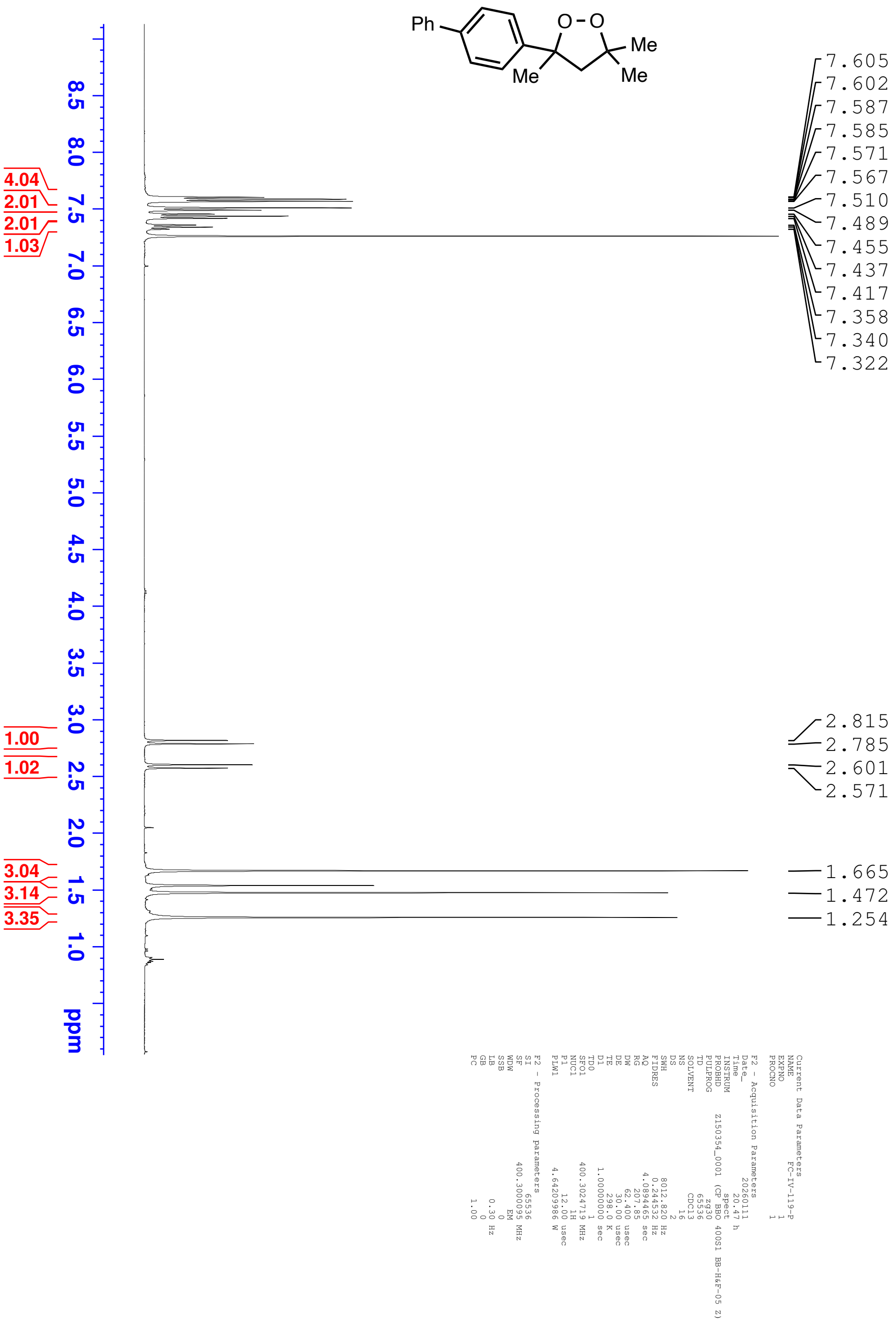

3-([1,1'-Biphenyl]-4-yl)-3,5,5-trimethyl-1,2-dioxolane (30).

Solvent: $CDCl_3$
100 MHz
$^{13}C\{^{1}H\}$ NMR

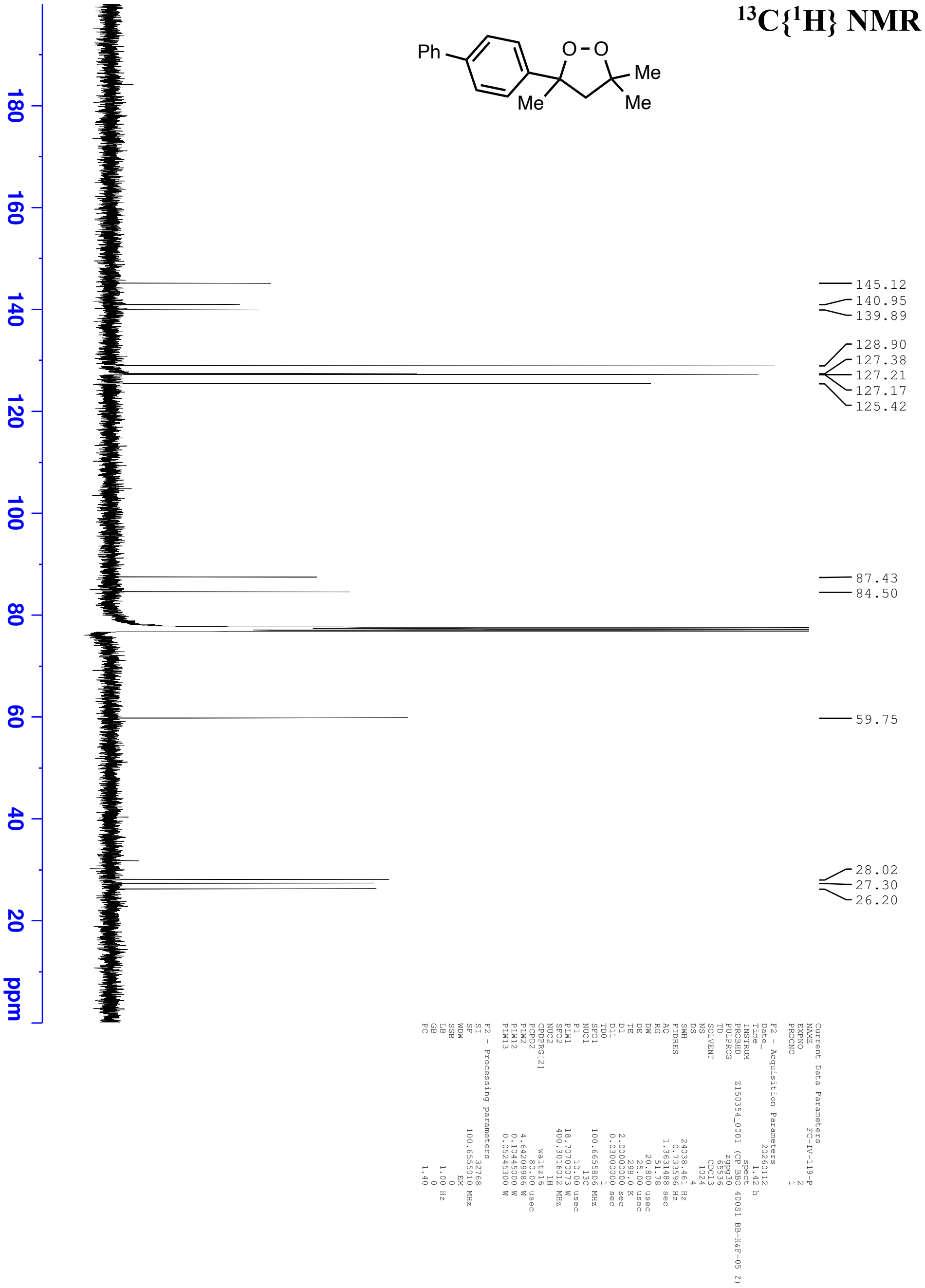

3-Methyl-3,5,5-triphenyl-1,2-dioxolane (31).

Solvent: $CDCl_3$
400 MHz
^{1}H NMR

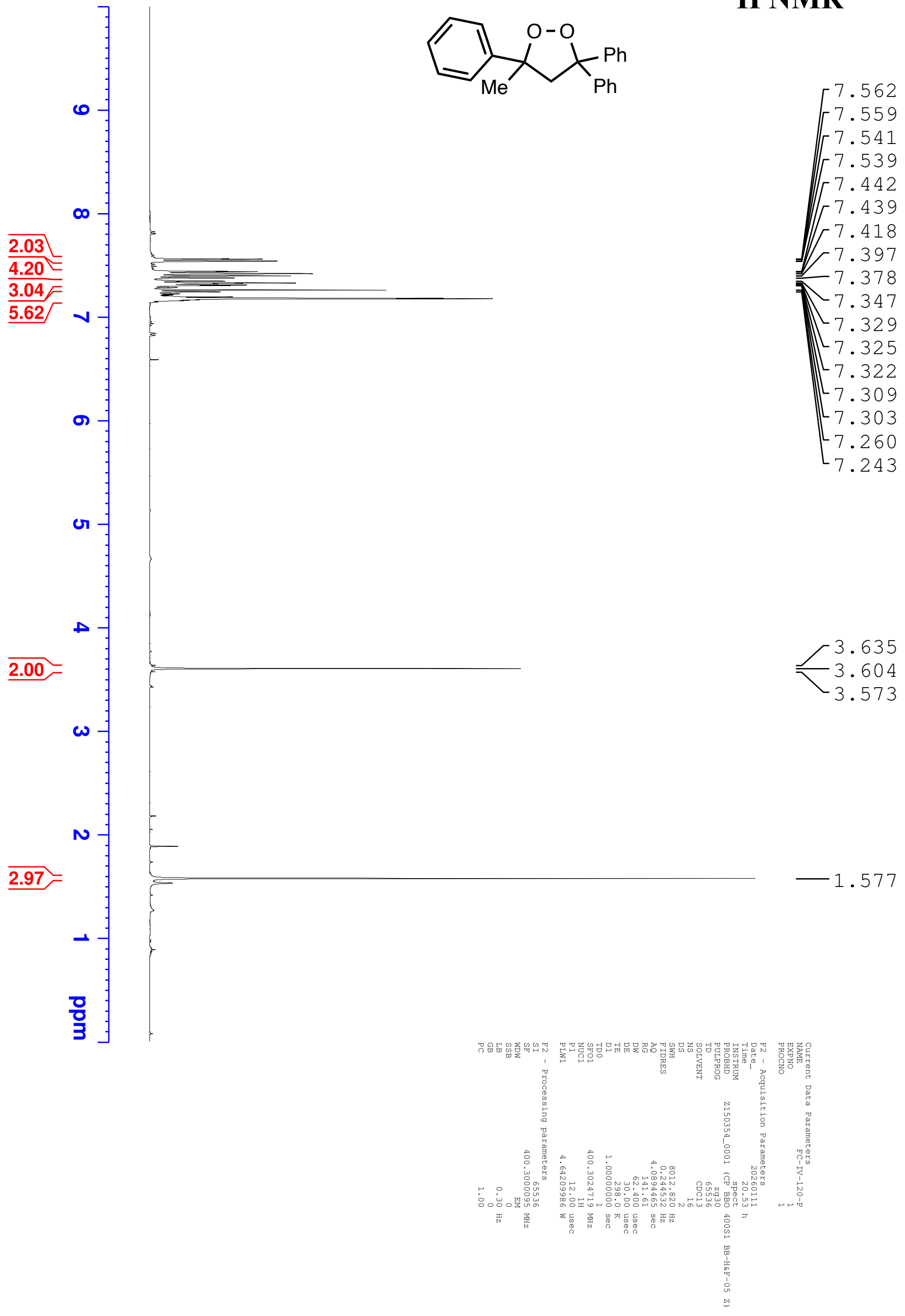

3-Methyl-3,5,5-triphenyl-1,2-dioxolane (31).

Solvent: $CDCl_3$
100 MHz
$^{13}C\{^1H\}$ NMR

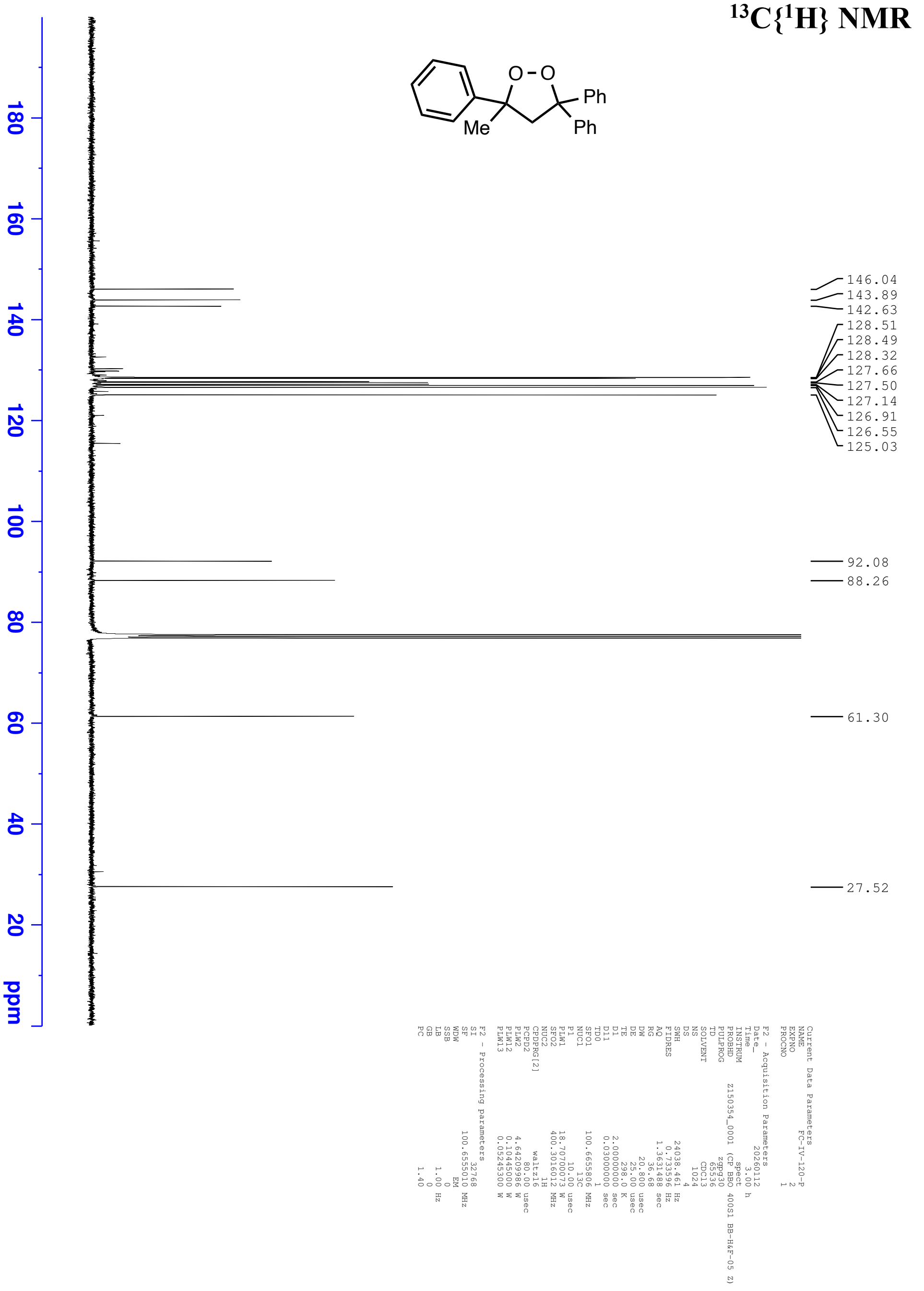

3-Allyl-5,5-dimethyl-3-phenyl-1,2-dioxolane (32).

Solvent: $CDCl_3$
400 MHz
^{1}H NMR

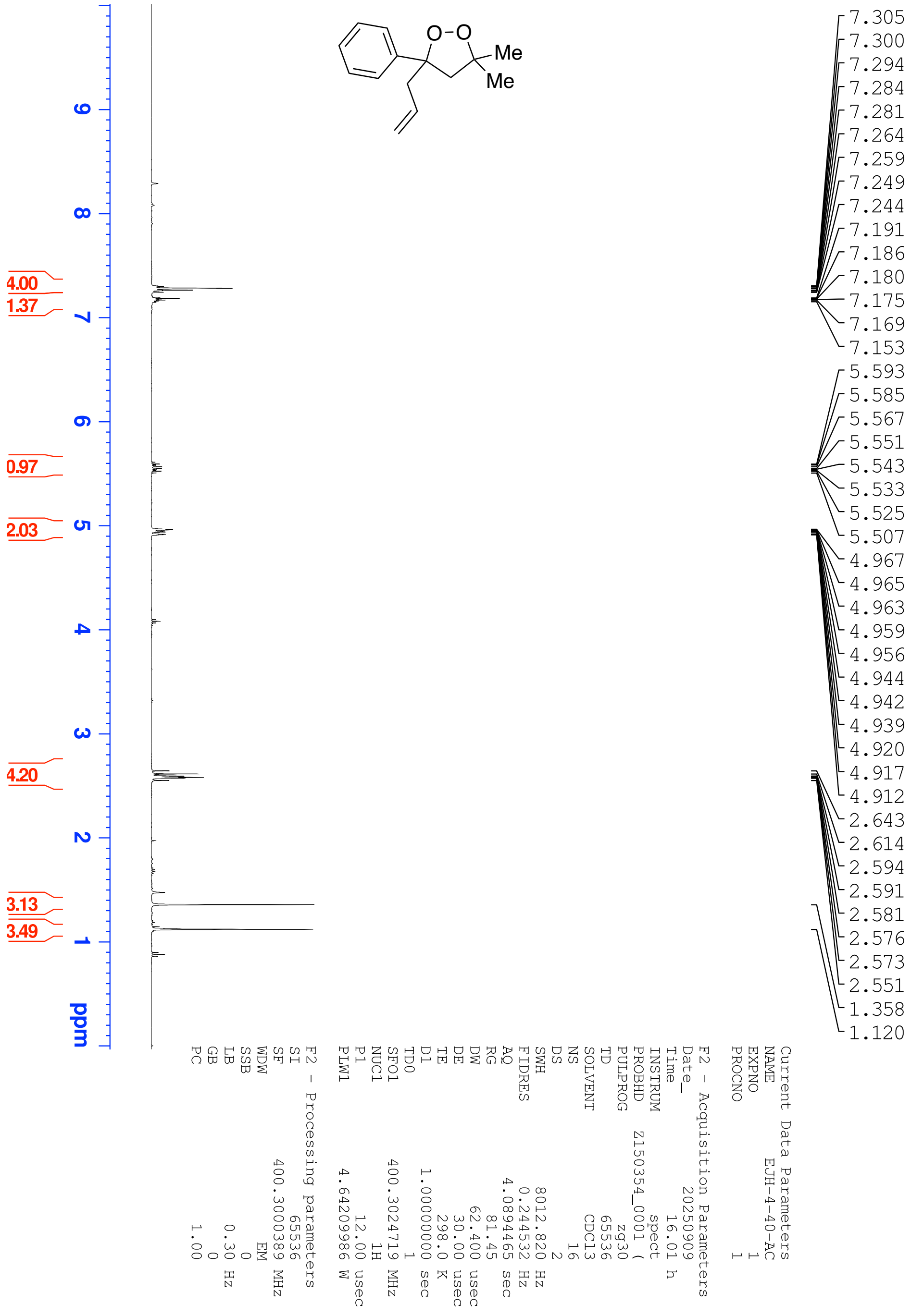

3-Allyl-5,5-dimethyl-3-phenyl-1,2-dioxolane (32).

Solvent: $CDCl_3$
100 MHz
$^{13}C\{^1H\}$ NMR

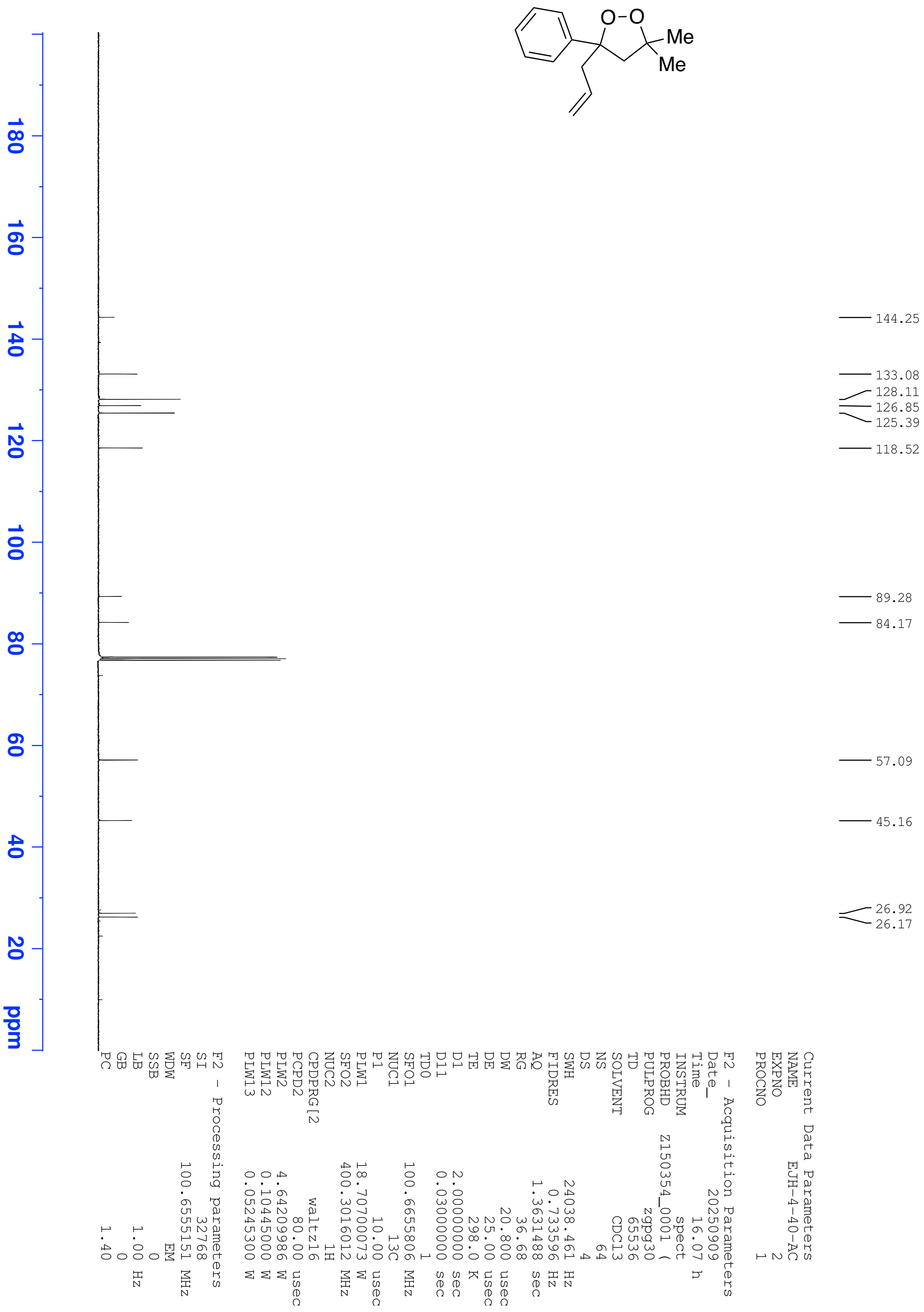

3,3,5-Trimethyl-5-undecyl-1,2-dioxolane (33).

Solvent: $CDCl_3$
400 MHz
^{1}H NMR

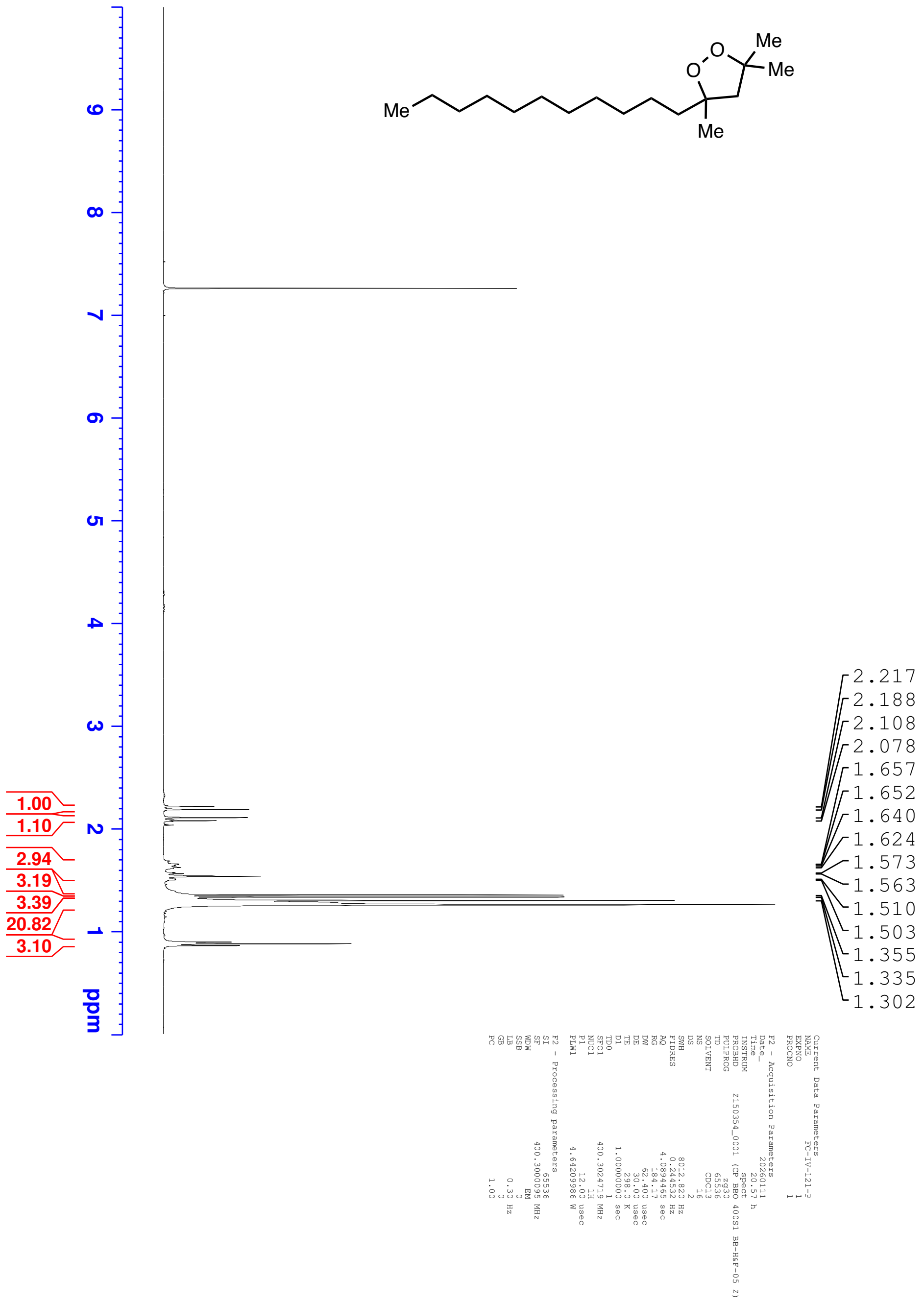

3,3,5-Trimethyl-5-undecyl-1,2-dioxolane (33).

Solvent: $CDCl_3$
100 MHz
$^{13}C\{^1H\}$ NMR

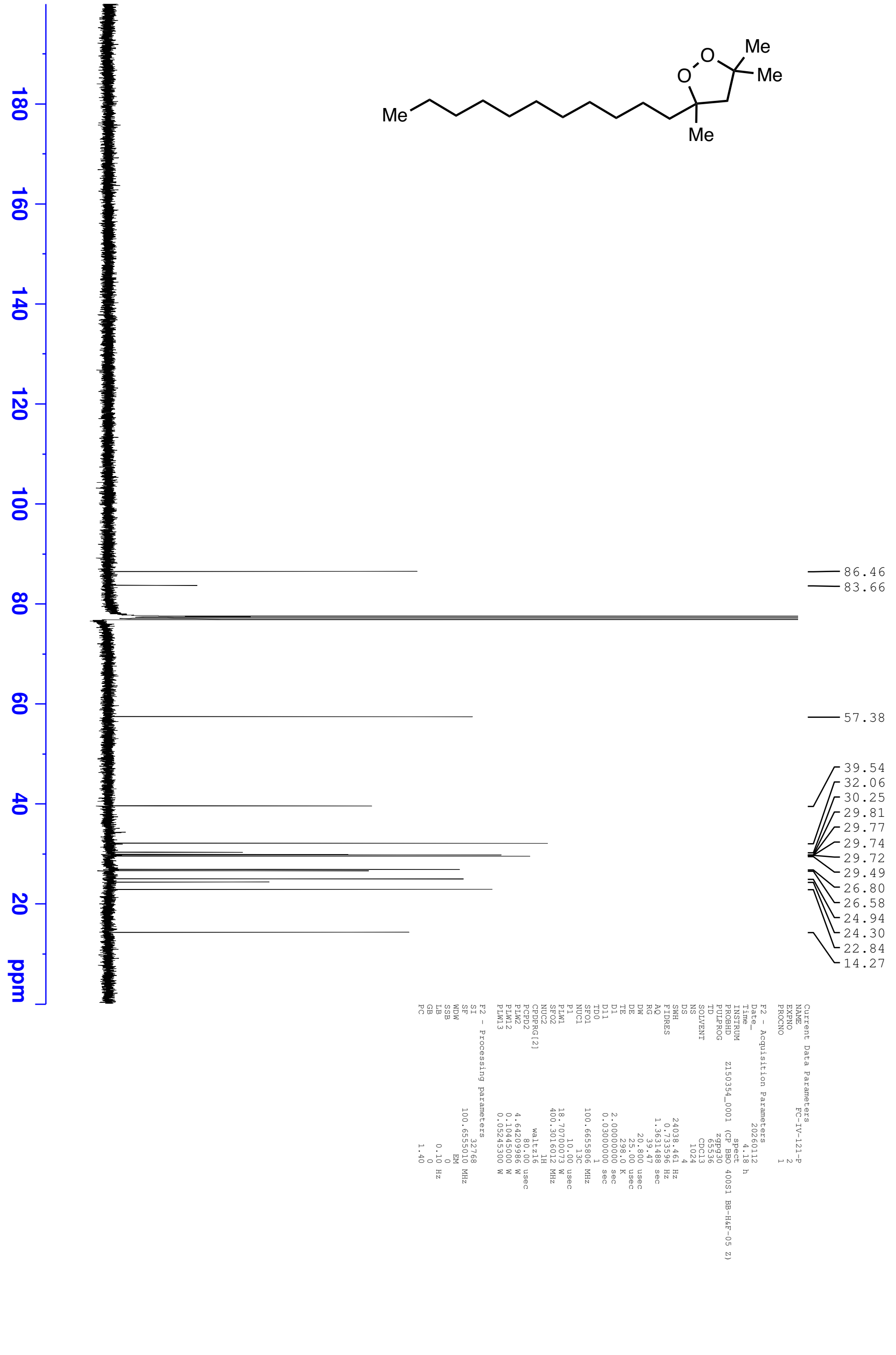

3,3-Dimethyl-5-phenyl-1,2-dioxolane (34).

Solvent: $CDCl_3$
400 MHz
^{1}H NMR

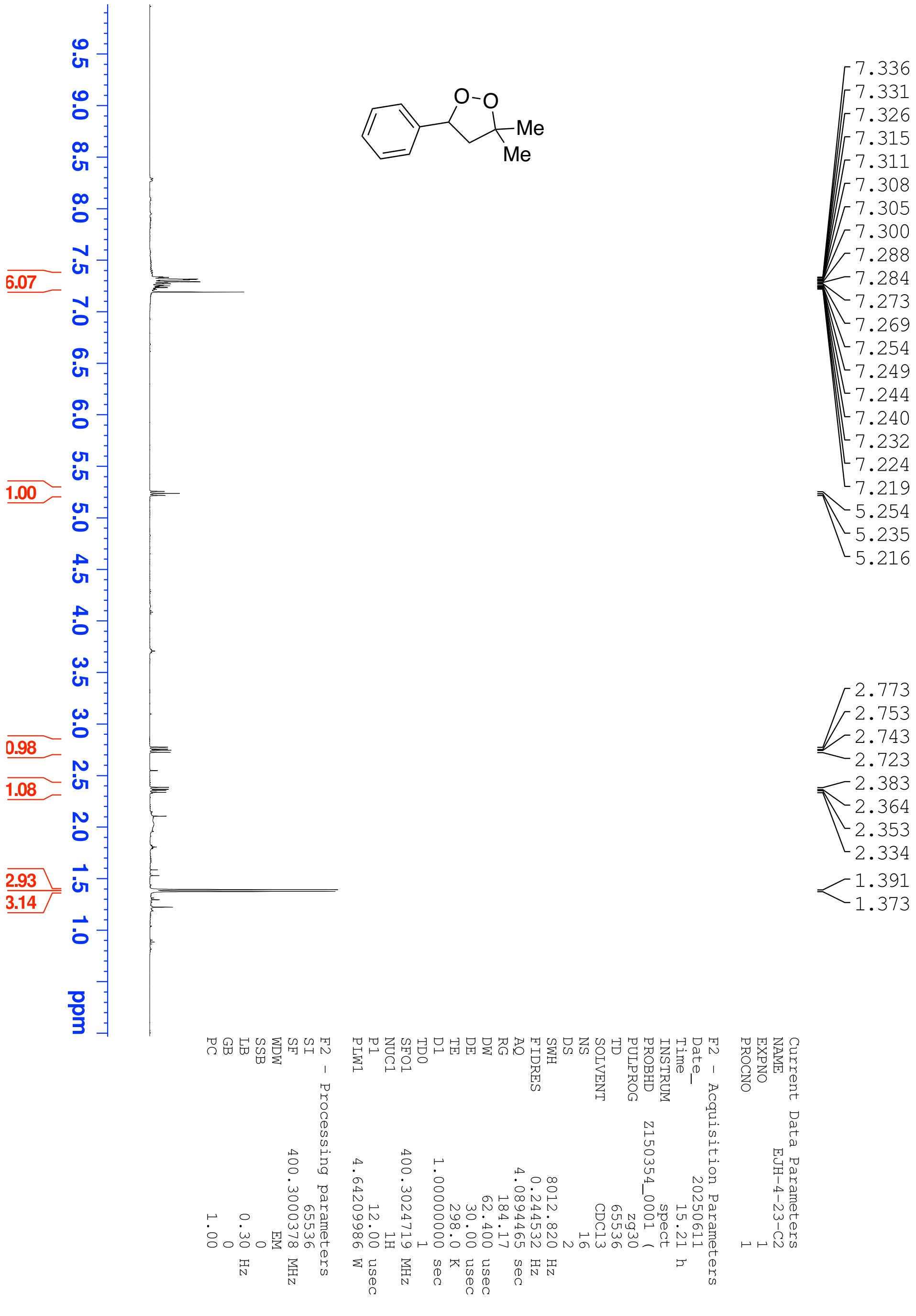

3,3-Dimethyl-5-phenyl-1,2-dioxolane (34).

Solvent: $CDCl_3$
100 MHz
$^{13}C\{^1H\}$ NMR

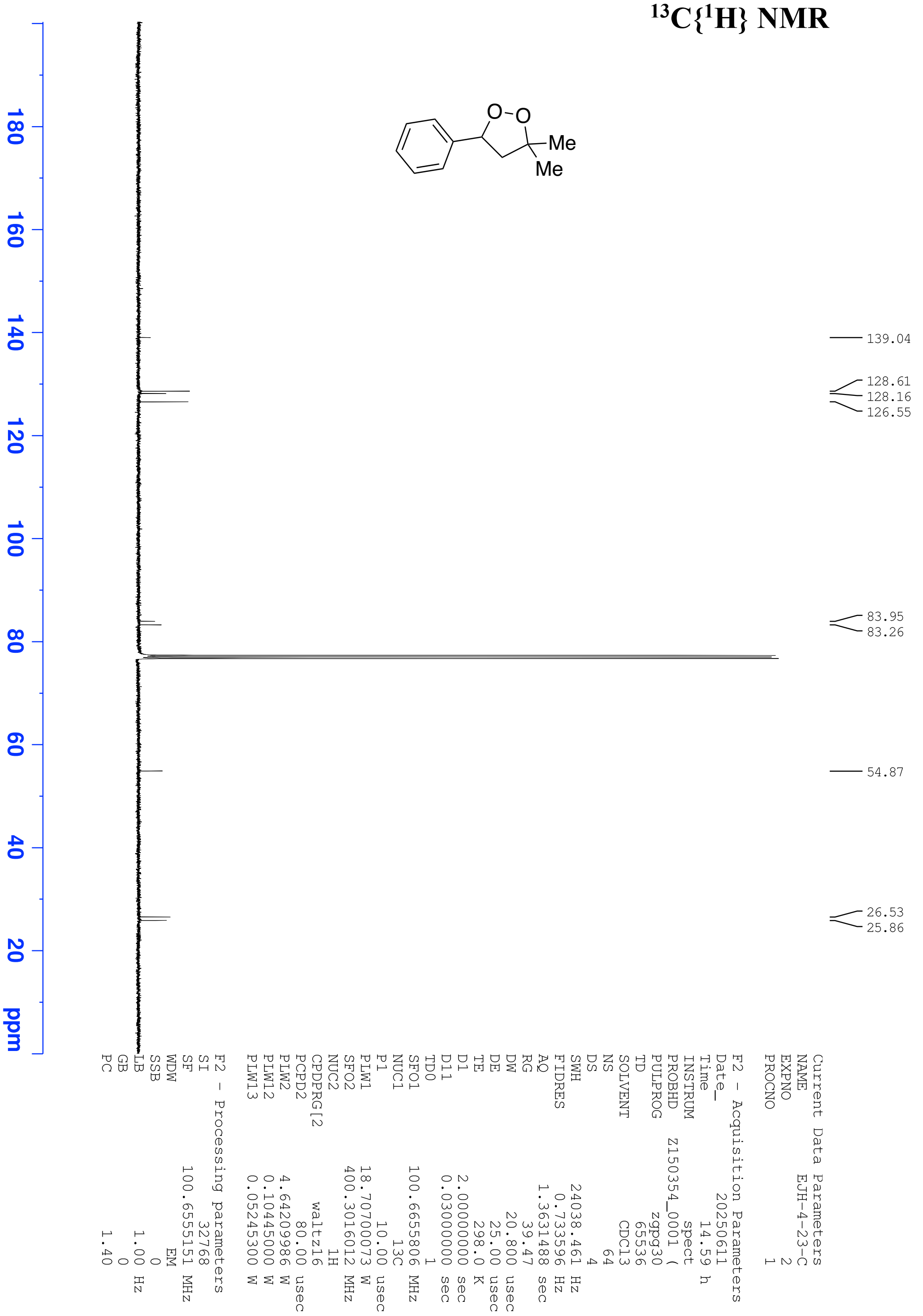

1-(4-(5,5-Dimethyl-1,2-dioxolan-3-yl)phenyl)ethan-1-one (35).

Solvent: $CDCl_3$
400 MHz
^{1}H NMR

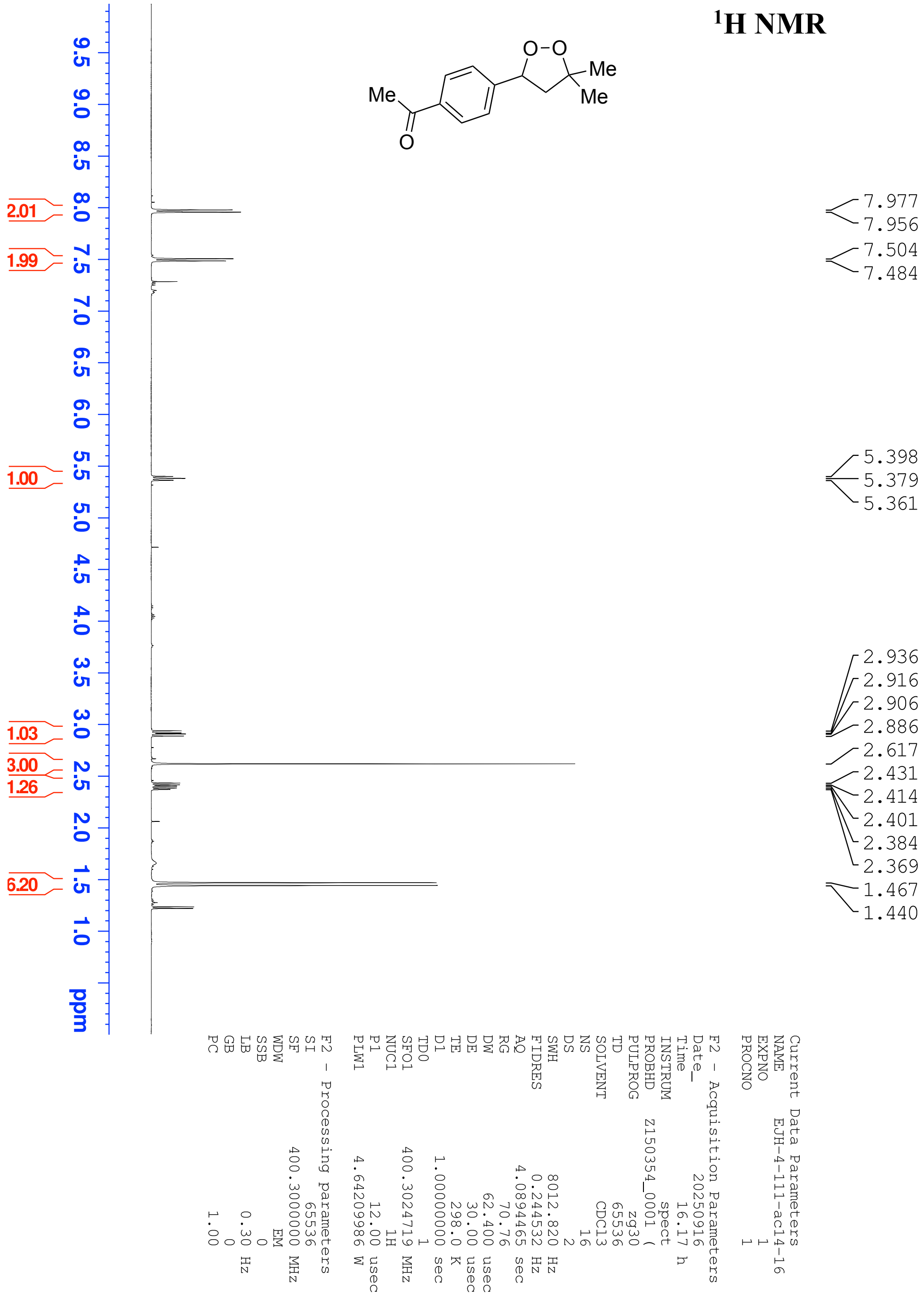

1-(4-(5,5-Dimethyl-1,2-dioxolan-3-yl)phenyl)ethan-1-one (35).

Solvent: $CDCl_3$
100 MHz
$^{13}C\{^{1}H\}$ NMR

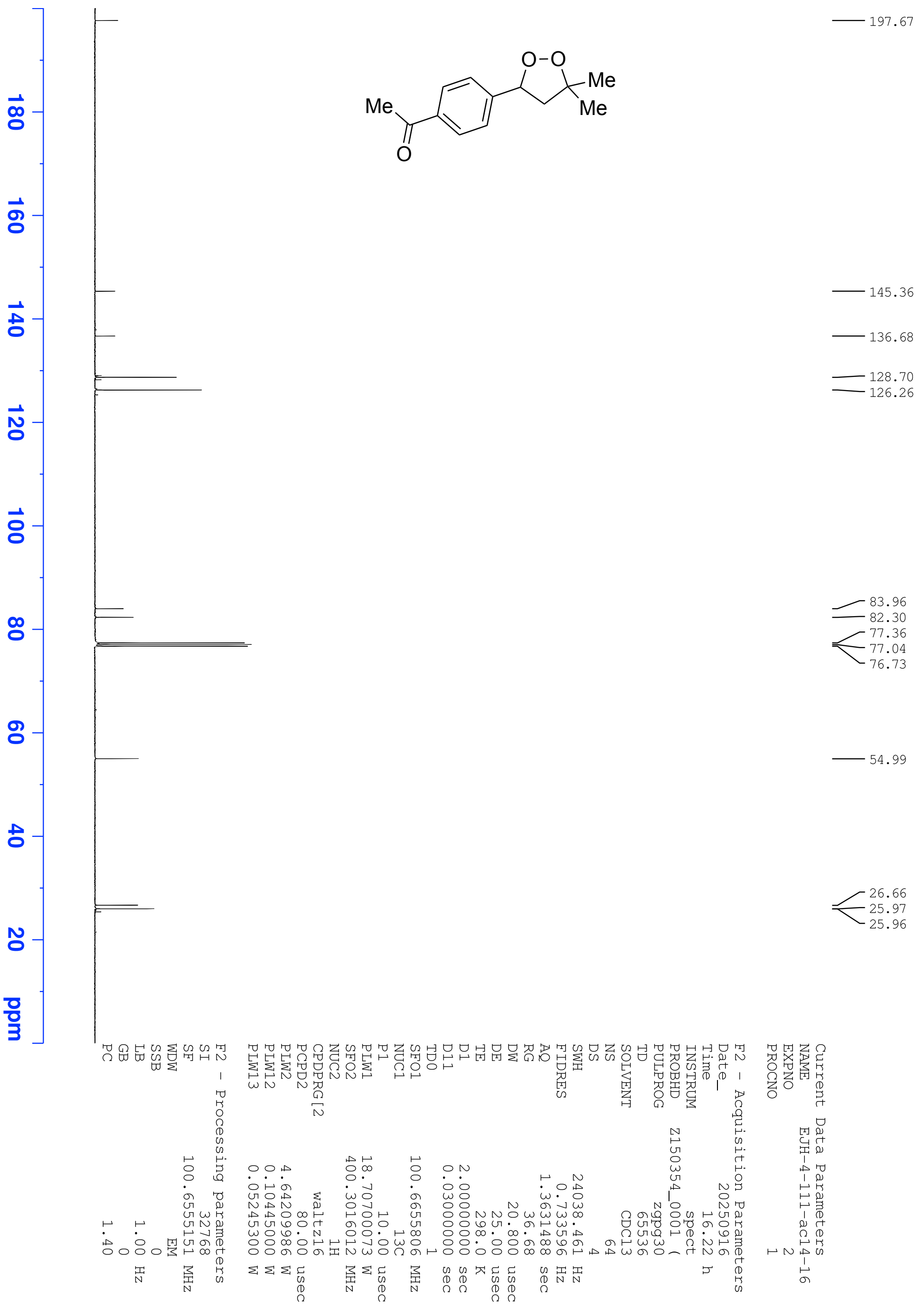

5-(4-Bromophenyl)-3,3-dimethyl-1,2-dioxolane (36).

Solvent: $CDCl_3$
400 MHz
^{1}H NMR

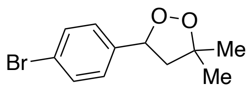

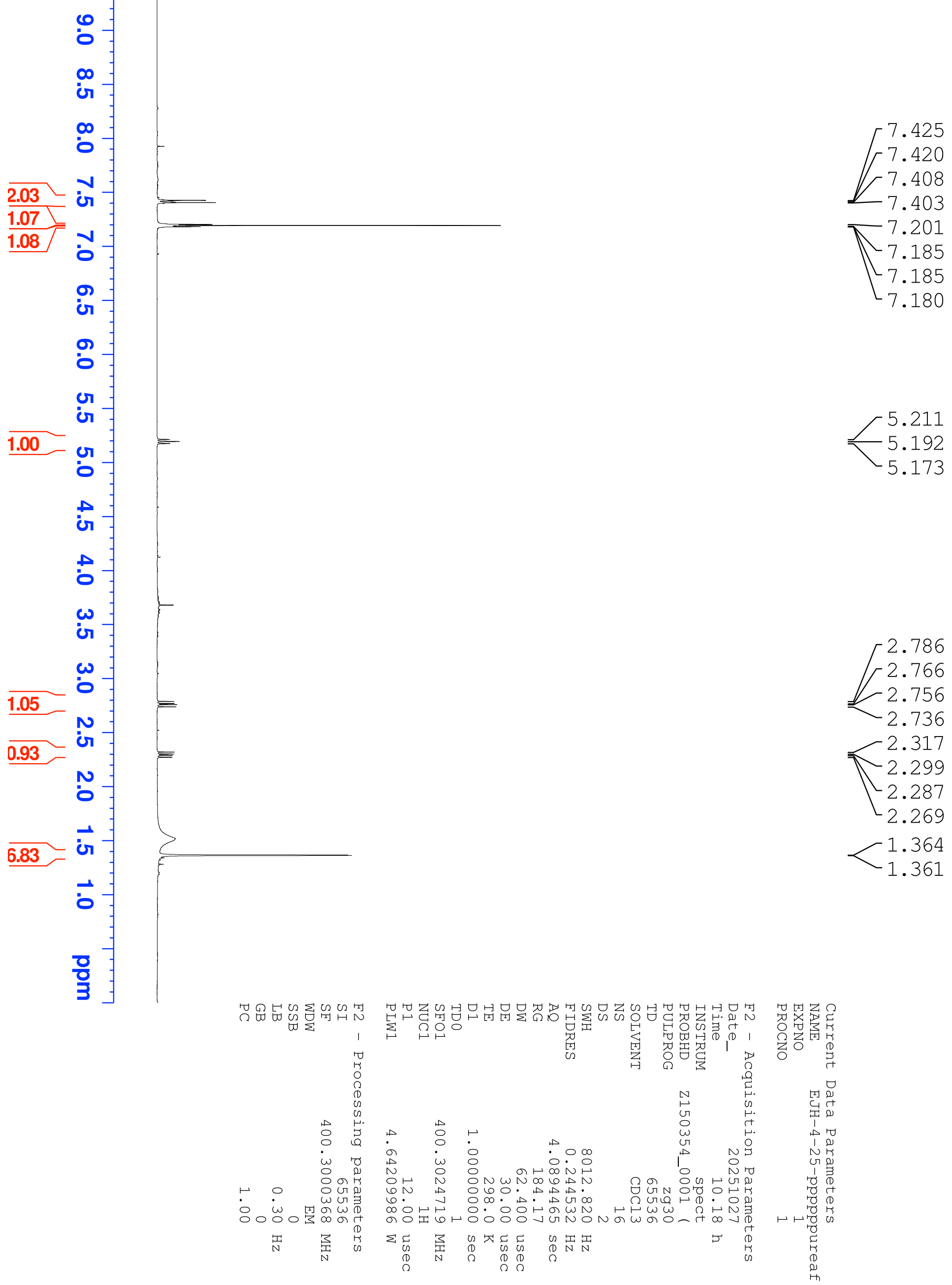

5-(4-Bromophenyl)-3,3-dimethyl-1,2-dioxolane (36).

Solvent: $CDCl_3$
100 MHz
$^{13}C\{^{1}H\}$ NMR

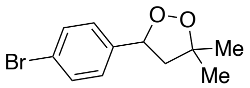

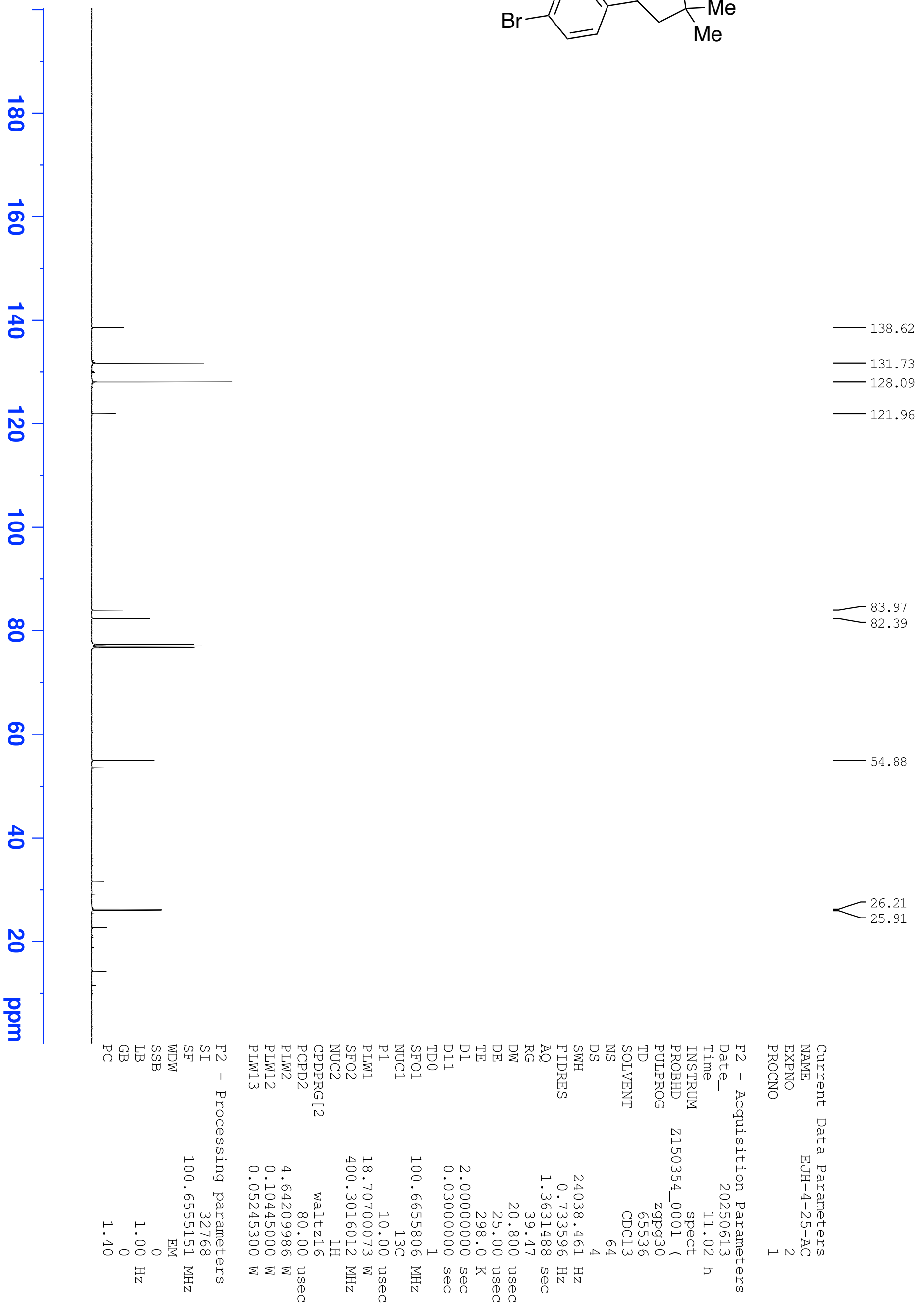

5-Methyl-3,3-diphenyl-1,2-dioxolane (37).

Solvent: $CDCl_3$
400 MHz
^{1}H NMR

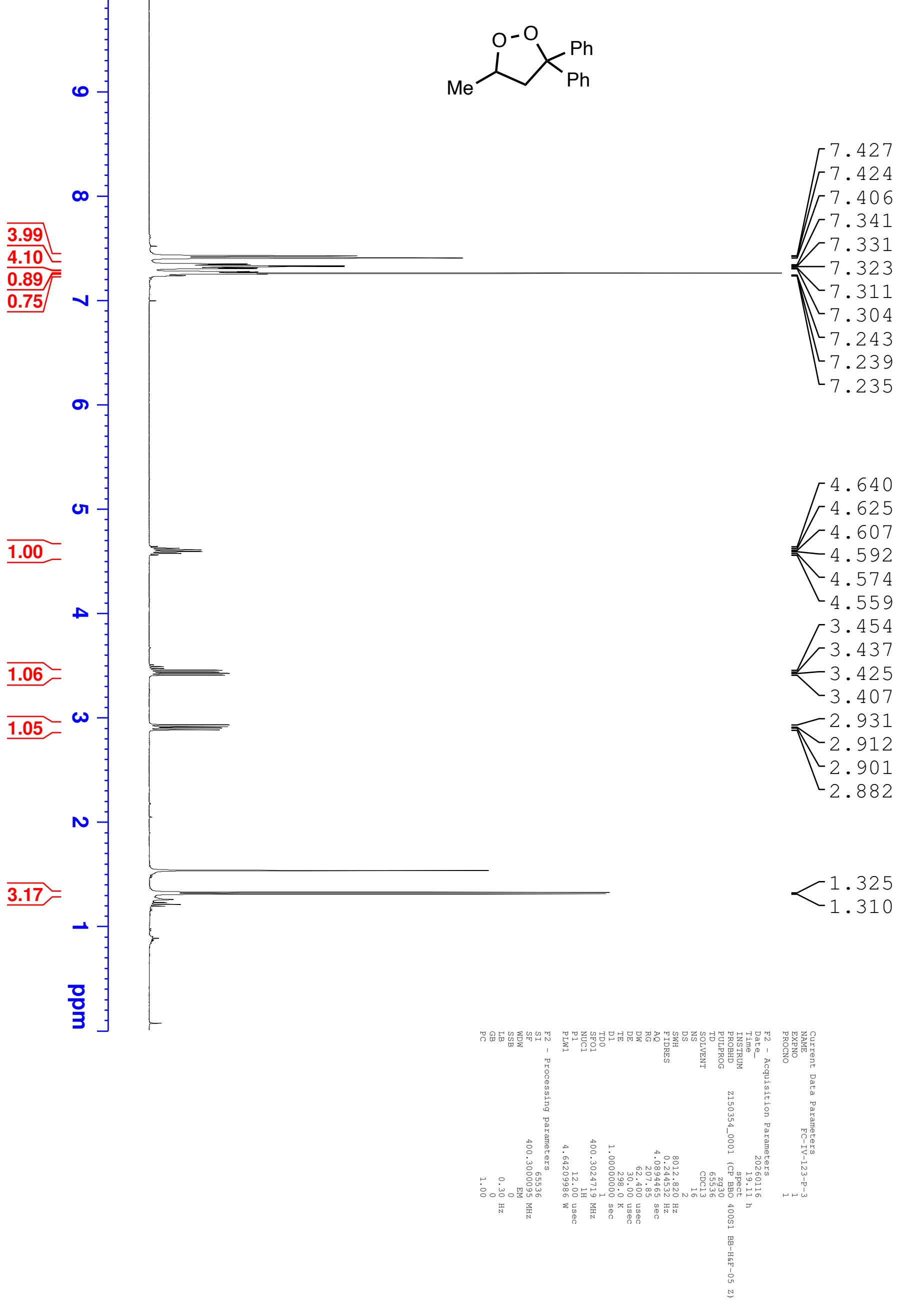

5-Methyl-3,3-diphenyl-1,2-dioxolane (37).

Solvent: $CDCl_3$
100 MHz
$^{13}C\{^1H\}$ NMR

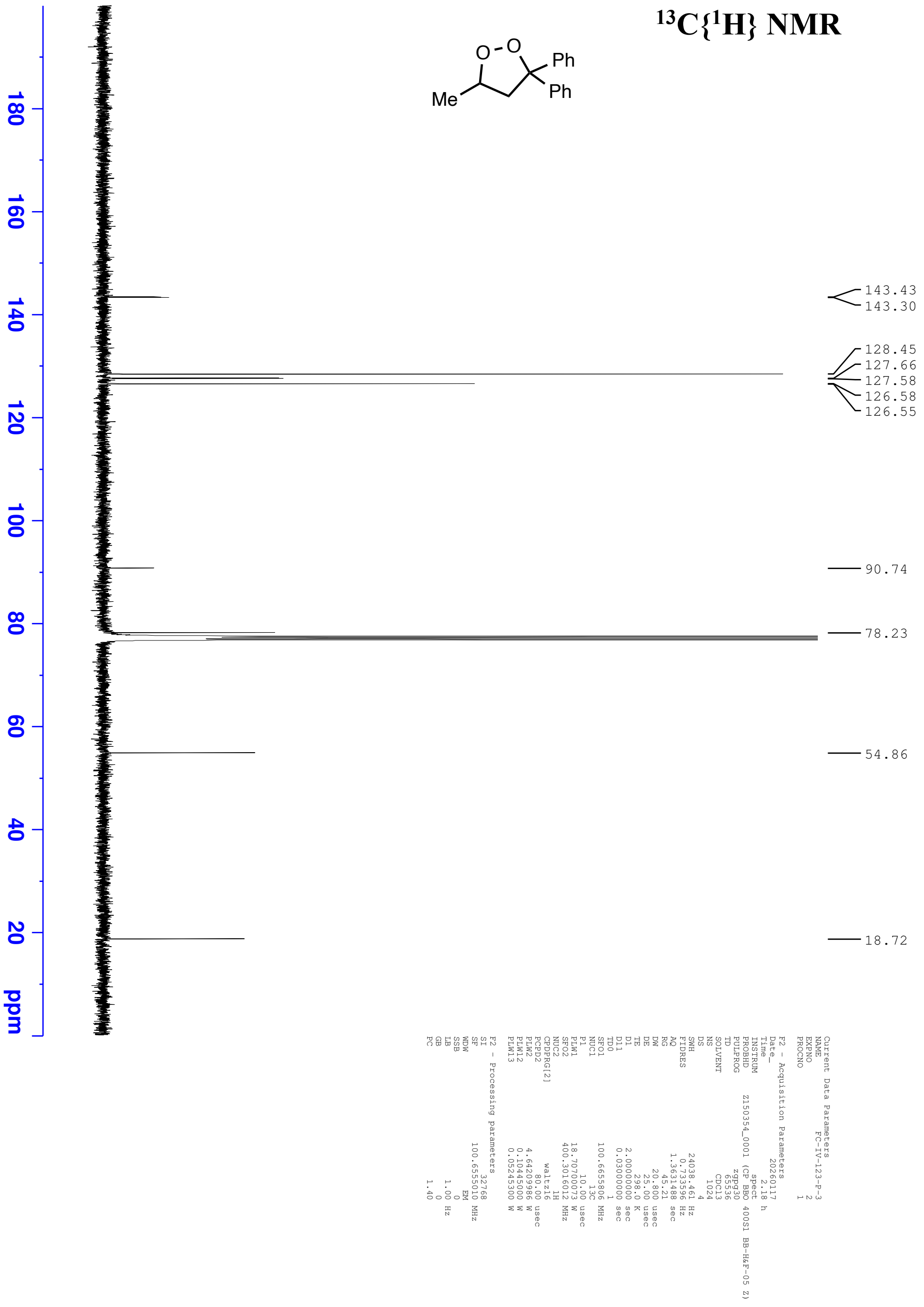

Solvent: $CDCl_3$
400 MHz
^{1}H NMR

(5s,8s)-3,3-Dimethyl-8-phenyl-1,2-dioxaspiro[4.5]decane (38).

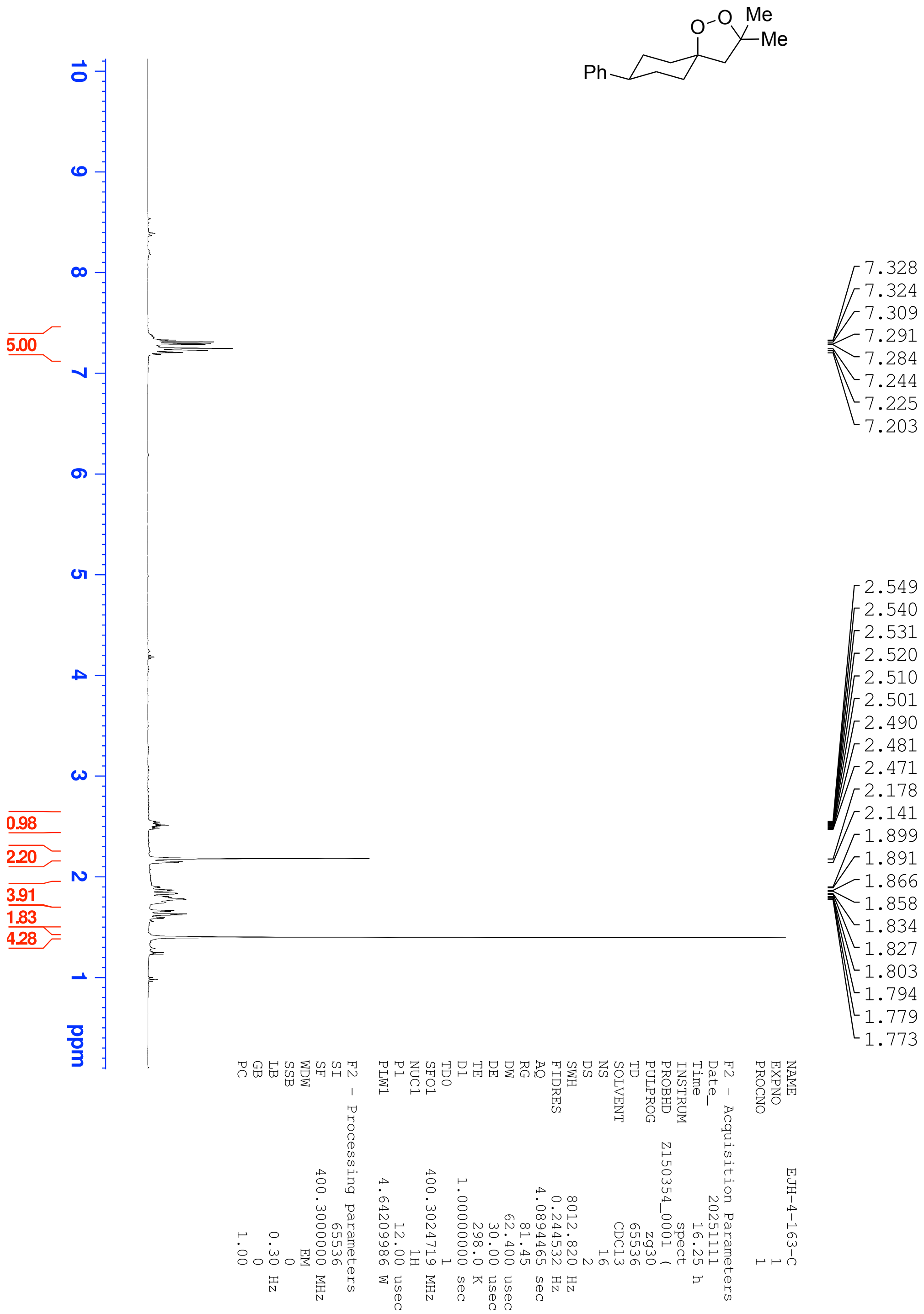

(5s,8s)-3,3-Dimethyl-8-phenyl-1,2-dioxaspiro[4.5]decane (38).

Solvent: $CDCl_3$
100 MHz
$^{13}C\{^1H\}$ NMR

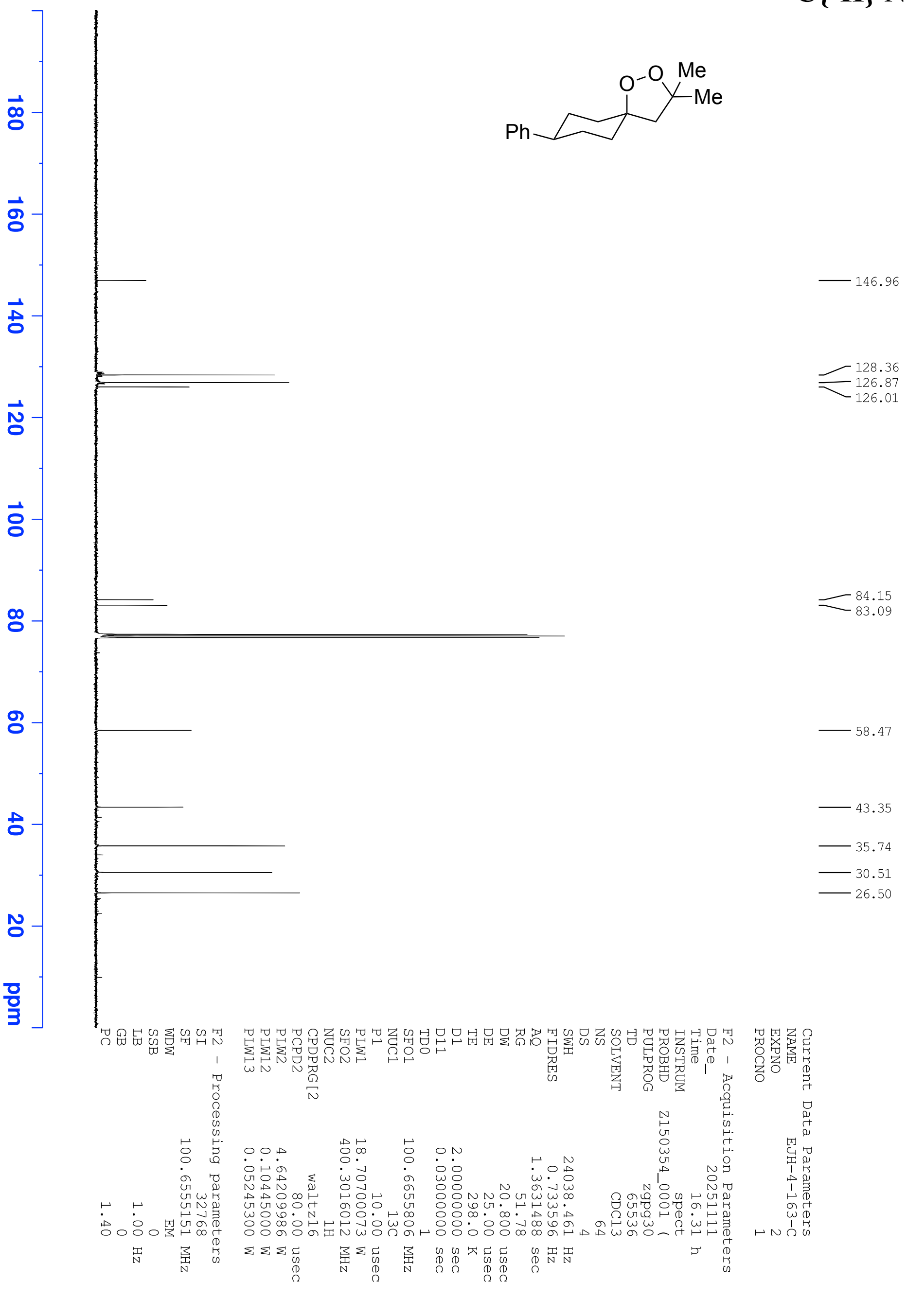

3,3-Dimethyl-4-phenyl-1,2-dioxolane (39).

Solvent: $CDCl_3$
400 MHz
^{1}H NMR

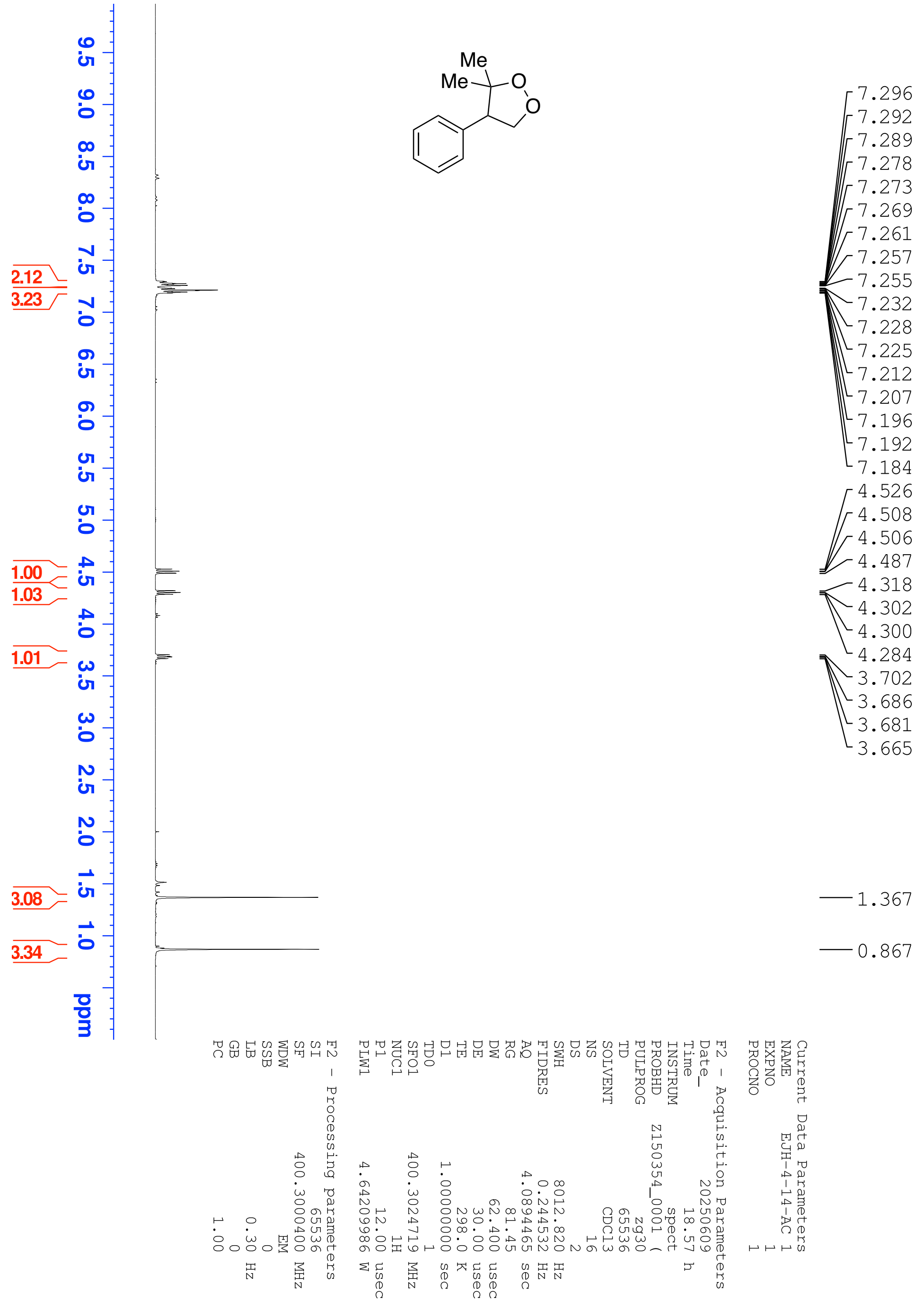

3,3-Dimethyl-4-phenyl-1,2-dioxolane (39).

Solvent: $CDCl_3$
100 MHz
$^{13}C\{^1H\}$ NMR

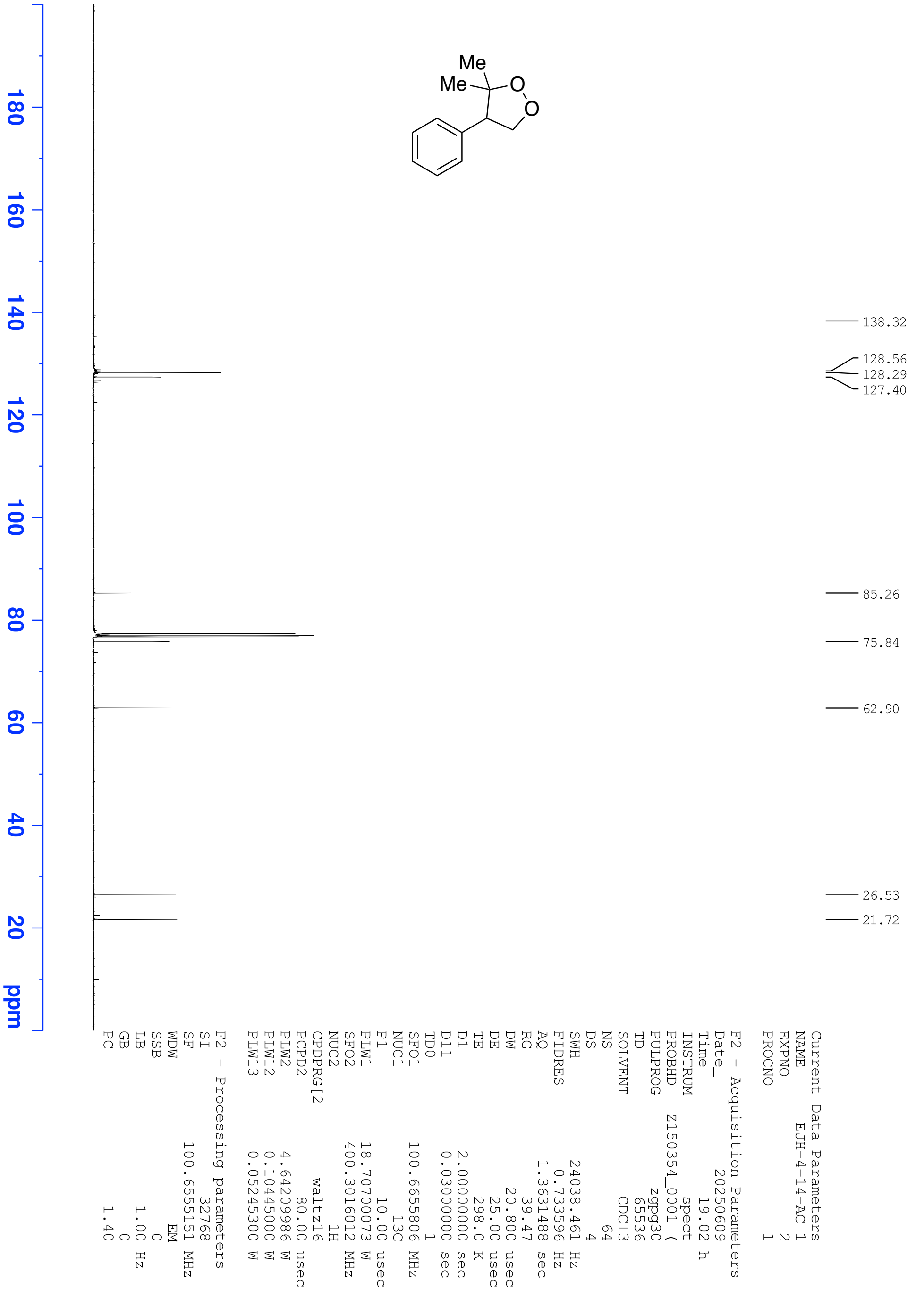

(3aS,7aS)-3,3-Dimethylhexahydro-3H-benzo[c][1,2]dioxole (40).

Solvent: $CDCl_3$
400 MHz
^{1}H NMR

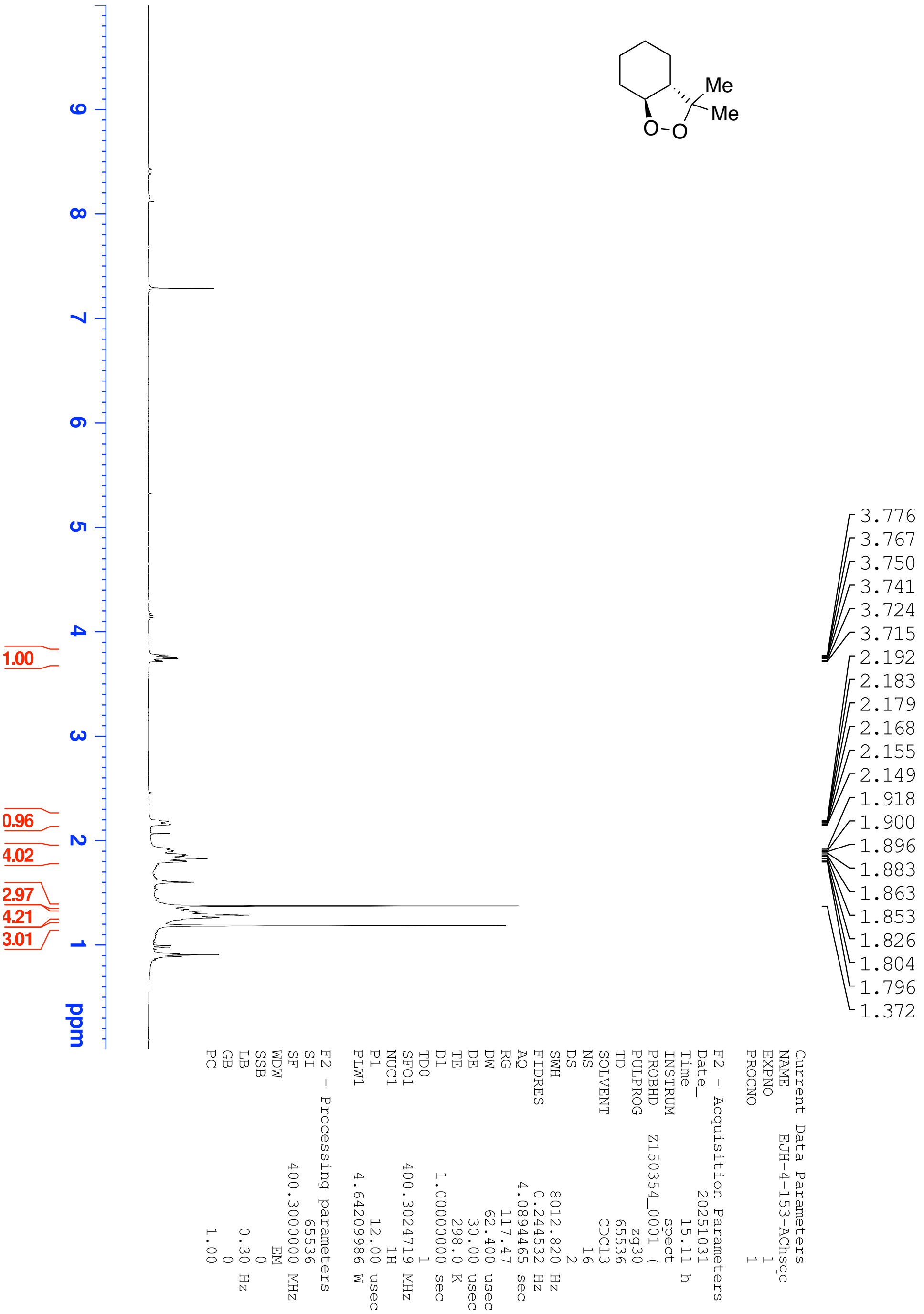

(3aS,7aS)-3,3-Dimethylhexahydro-3H-benzo[c][1,2]dioxole (40).

Solvent: $CDCl_3$
100 MHz
$^{13}C\{^1H\}$ NMR

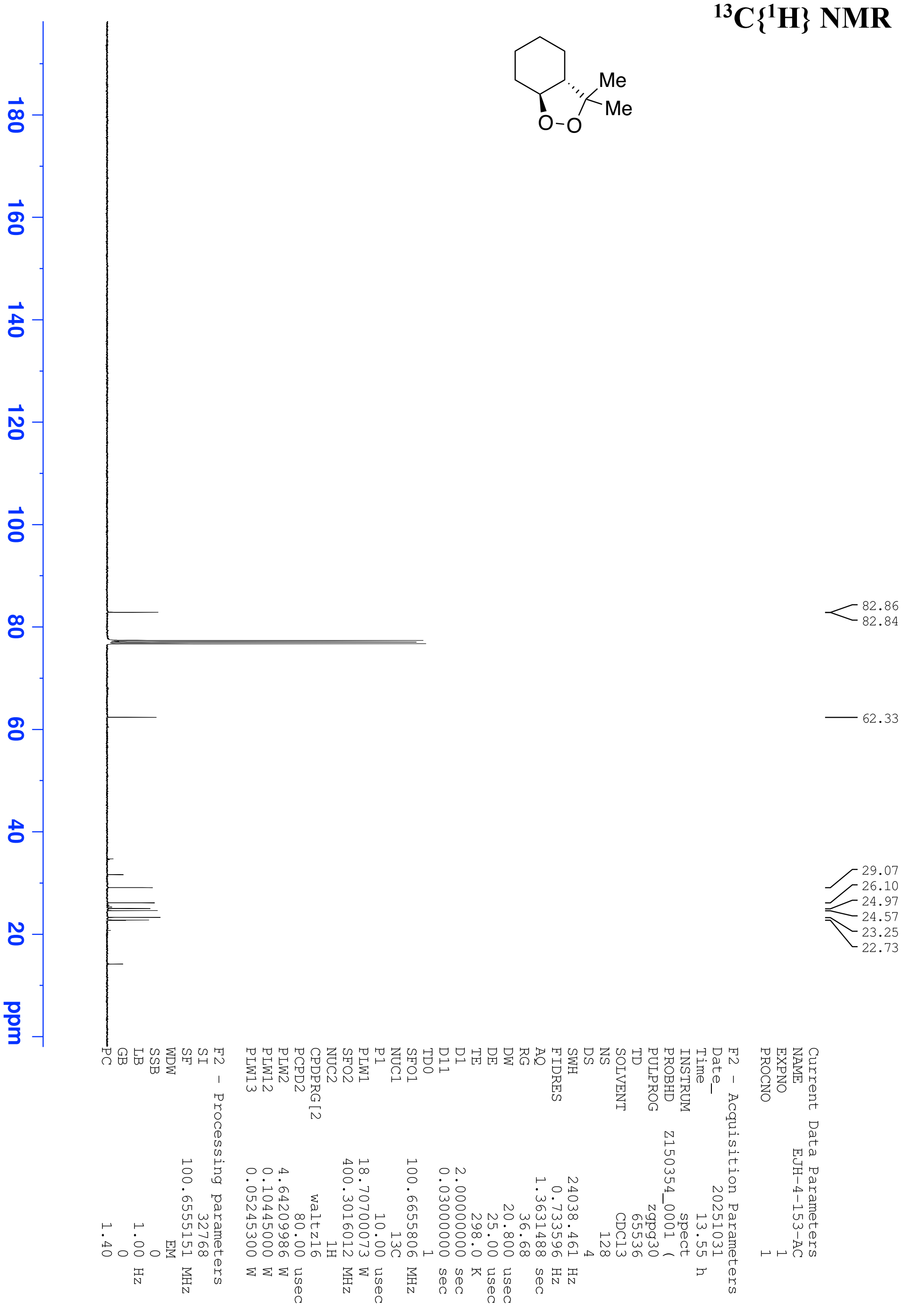

3,3-Dimethyl-6,6-diphenyl-1,2-dioxane (41).

Solvent: $CDCl_3$
400 MHz
^{1}H NMR

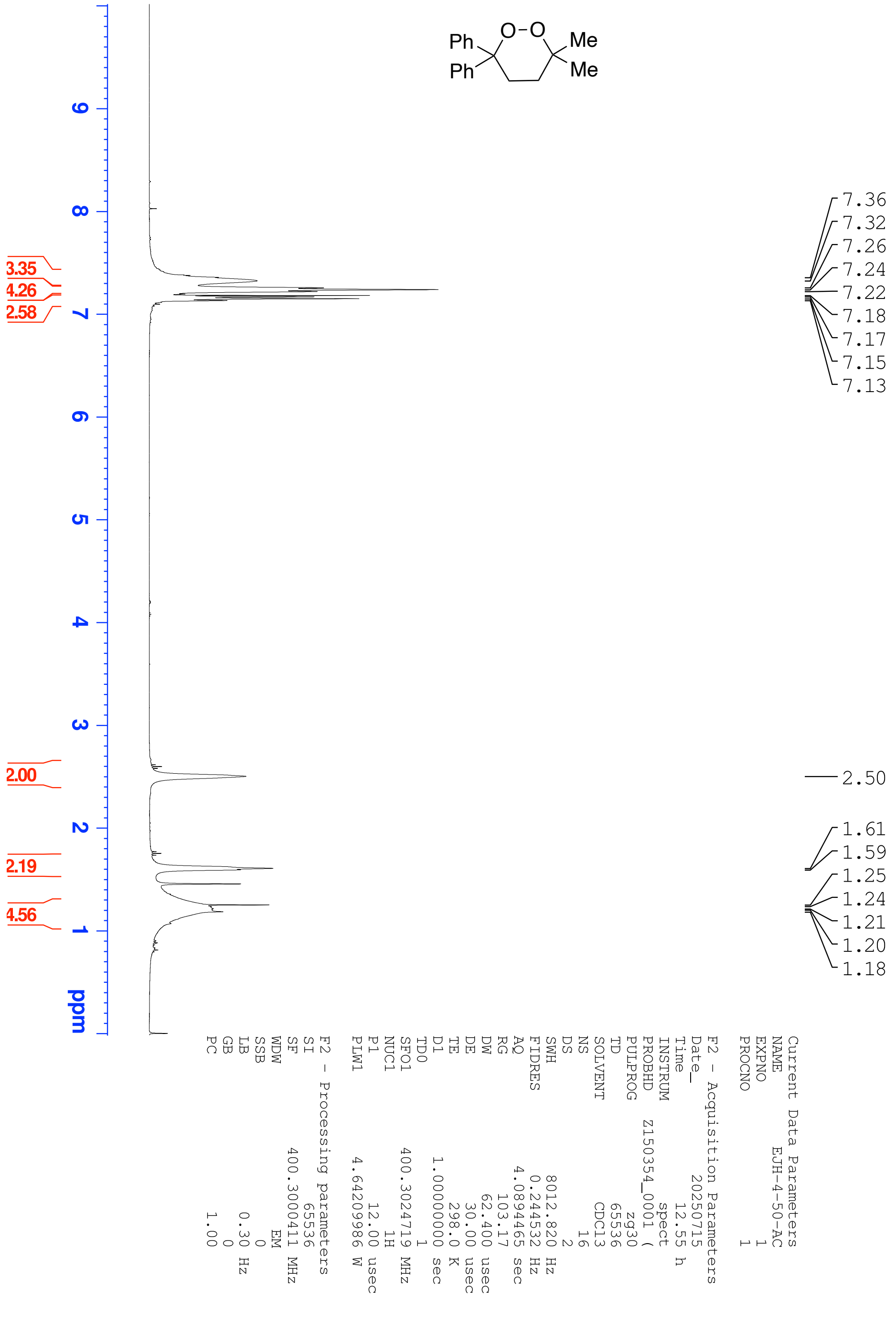

3,3-Dimethyl-6,6-diphenyl-1,2-dioxane (41).

Solvent: $CDCl_3$
100 MHz
$^{13}C\{^1H\}$ NMR

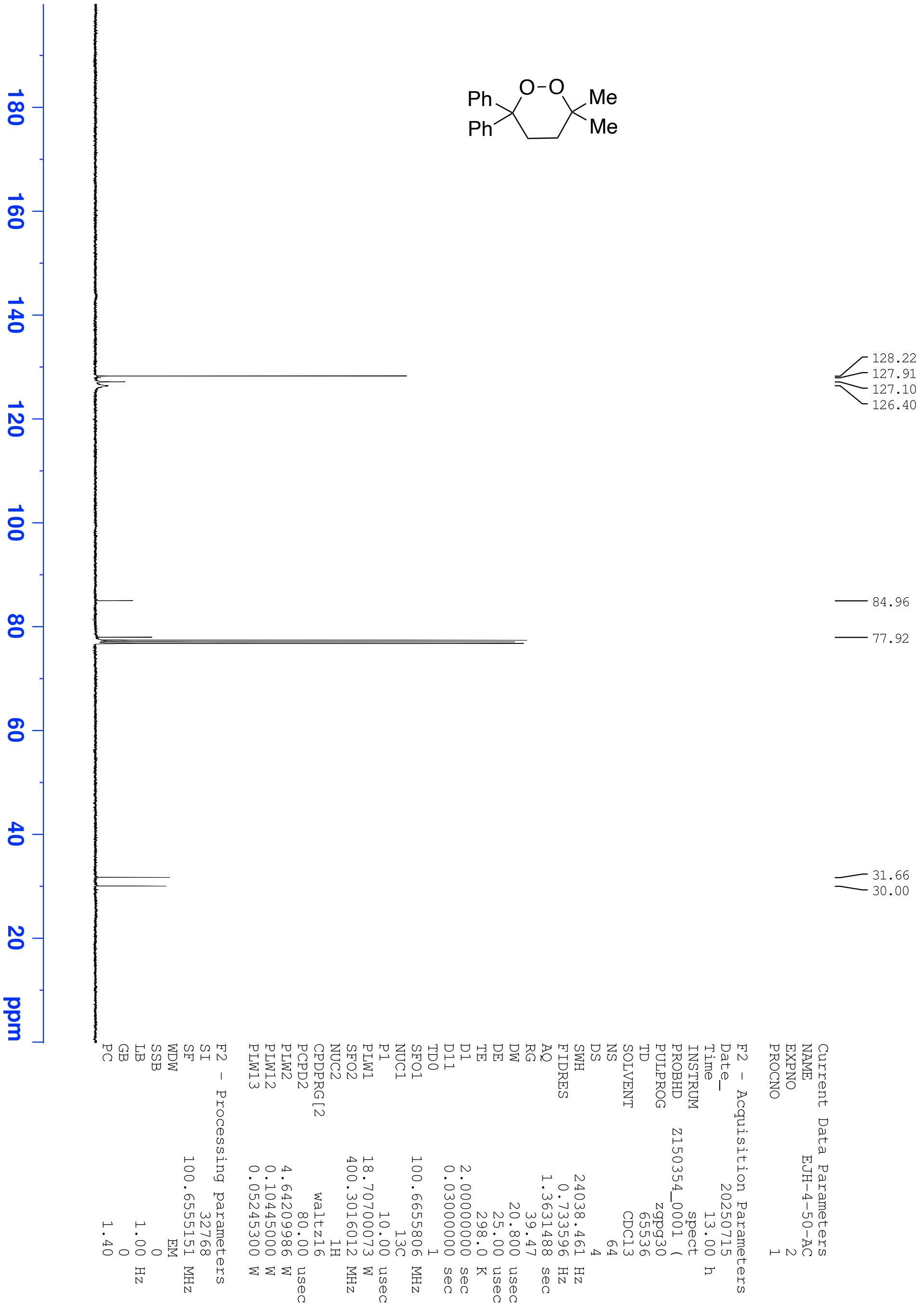

3,3-Dimethyl-6,6-diphenyl-1,2-dioxane (41).

Solvent: DMSO-D_6
Temperature: 70 ºC
400 MHz
^{1}H NMR

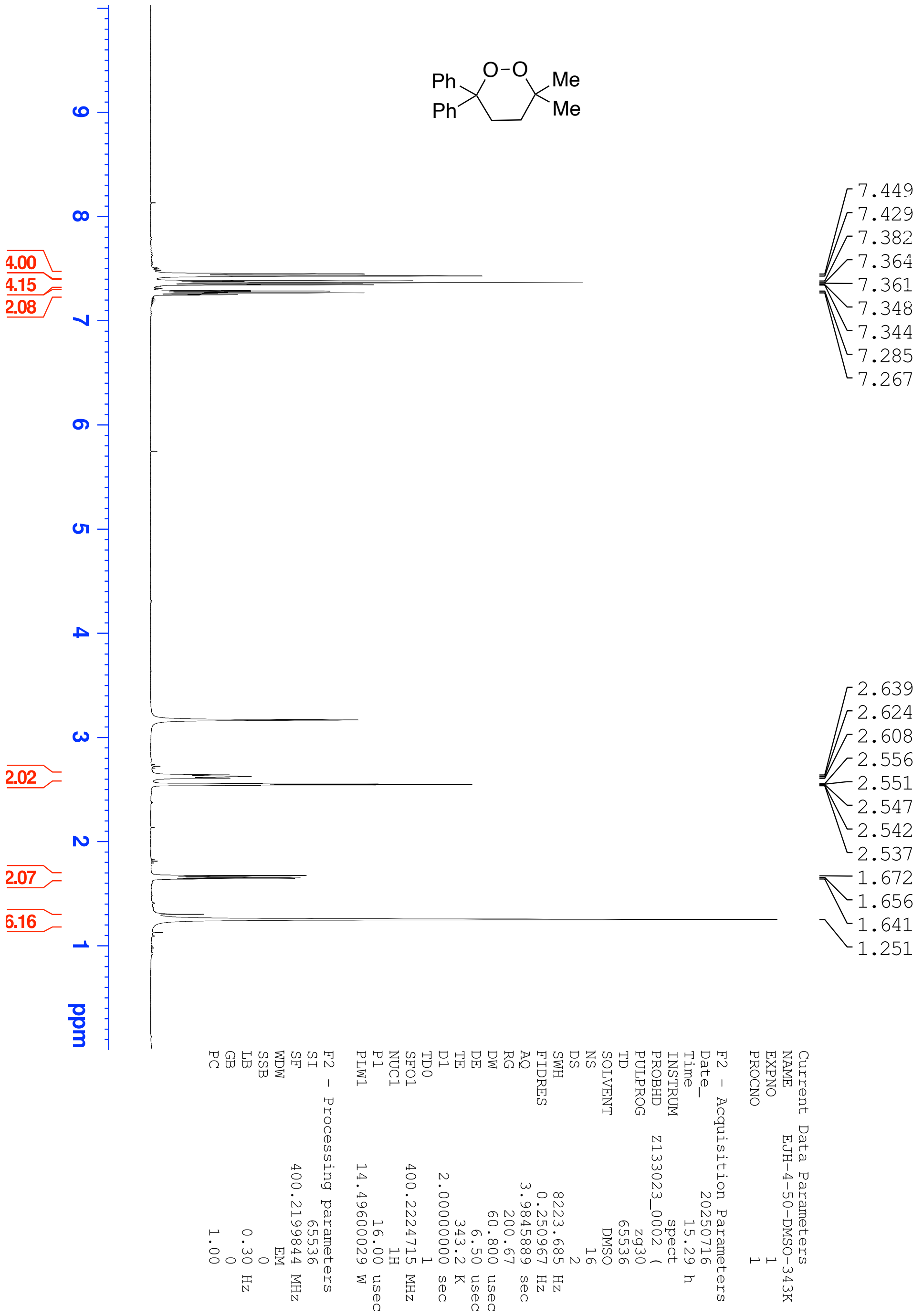

3,3-Dimethyl-6,6-diphenyl-1,2-dioxane (41).

Solvent: DMSO-D$_6$
Temperature: 70 °C
100 MHz
^{13}C NMR

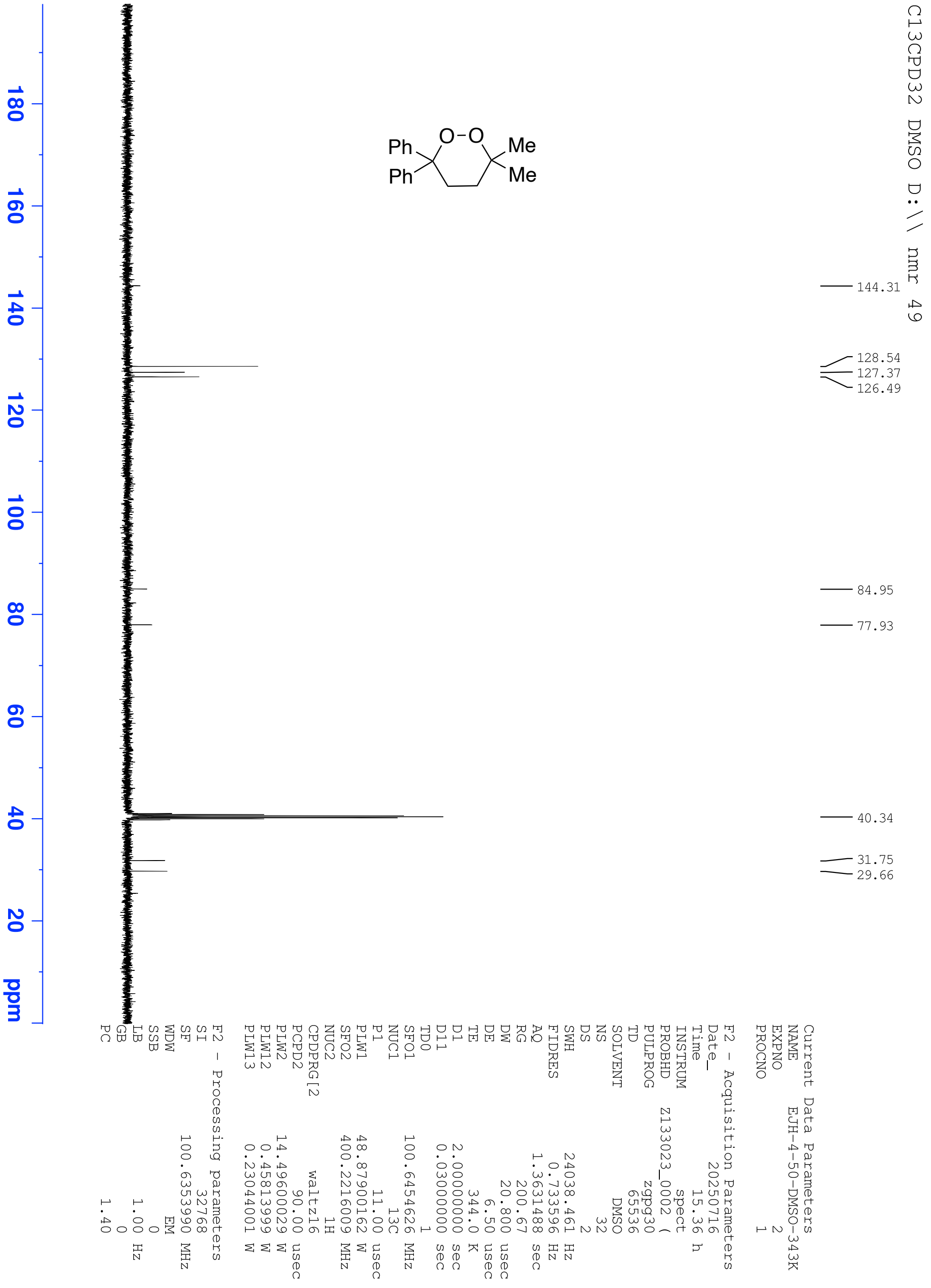

2,4-Dimethylpentadec-1-en-4-ol (S1).

Solvent: $CDCl_3$
400 MHz
^{1}H NMR

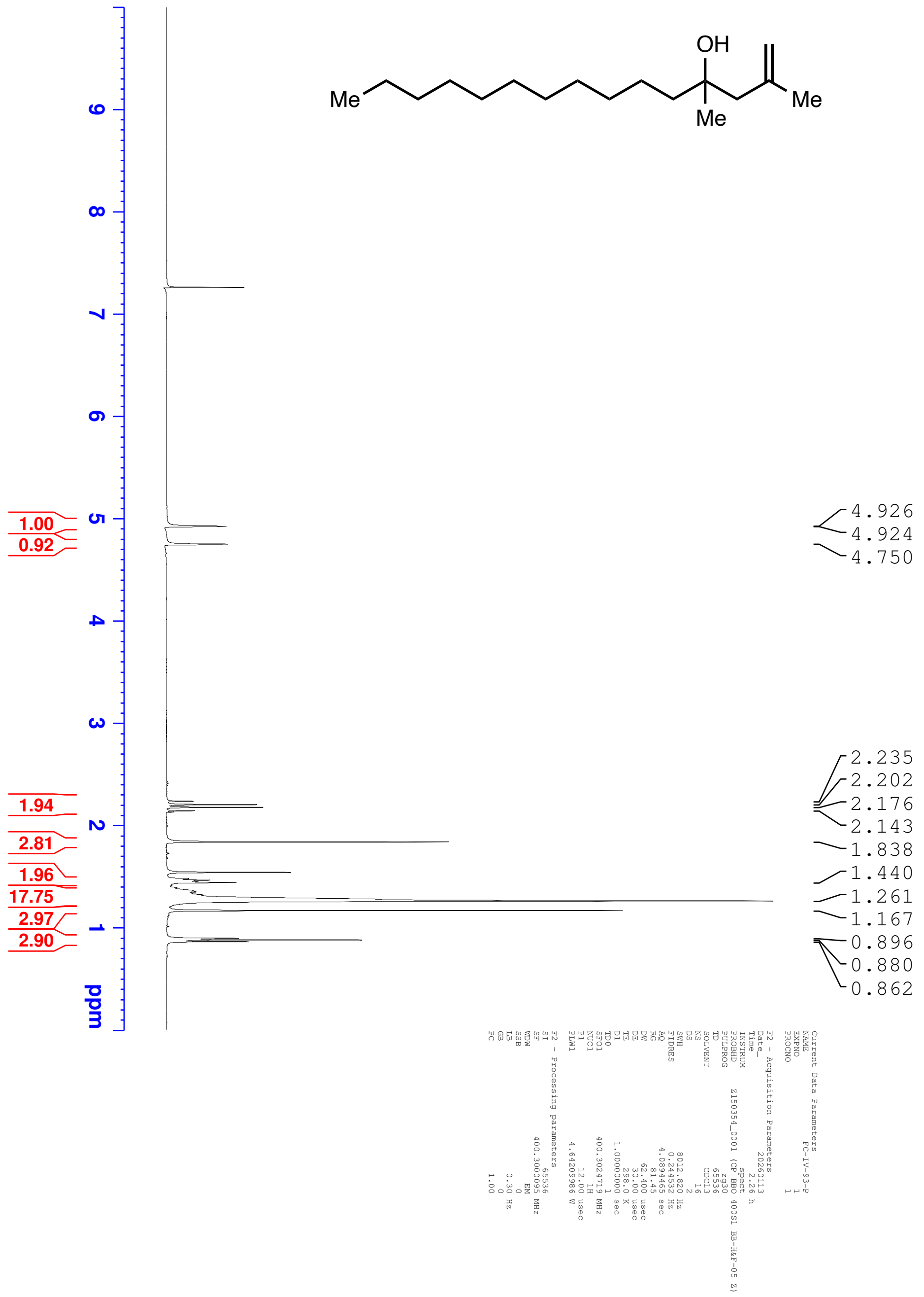

2,4-Dimethylpentadec-1-en-4-ol (S1).

Solvent: $CDCl_3$
100 MHz
$^{13}C\{^1H\}$ NMR

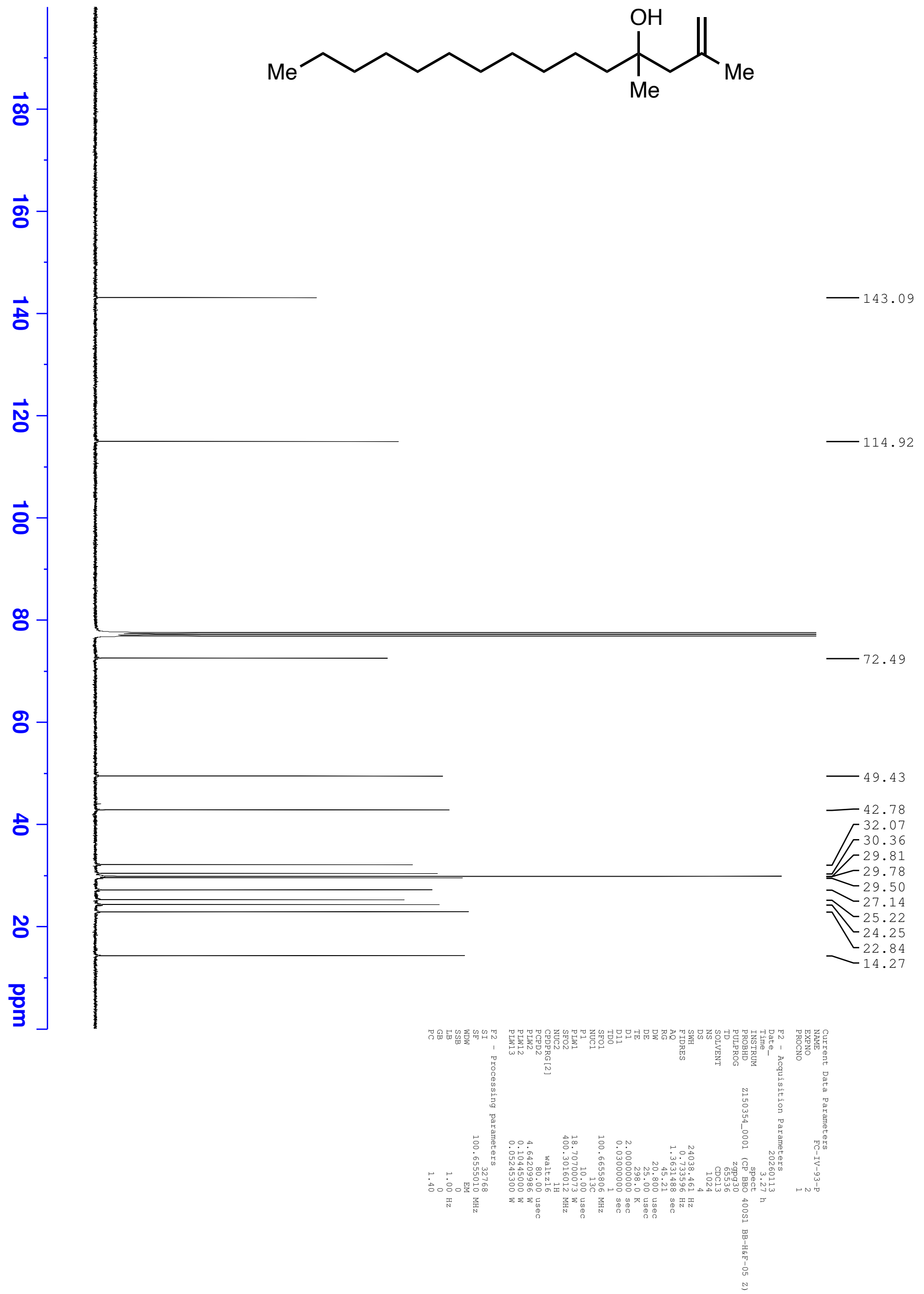

1,1,3-Triphenylbut-3-en-1-ol (S6).

Solvent: $CDCl_3$
400 MHz
^1H NMR

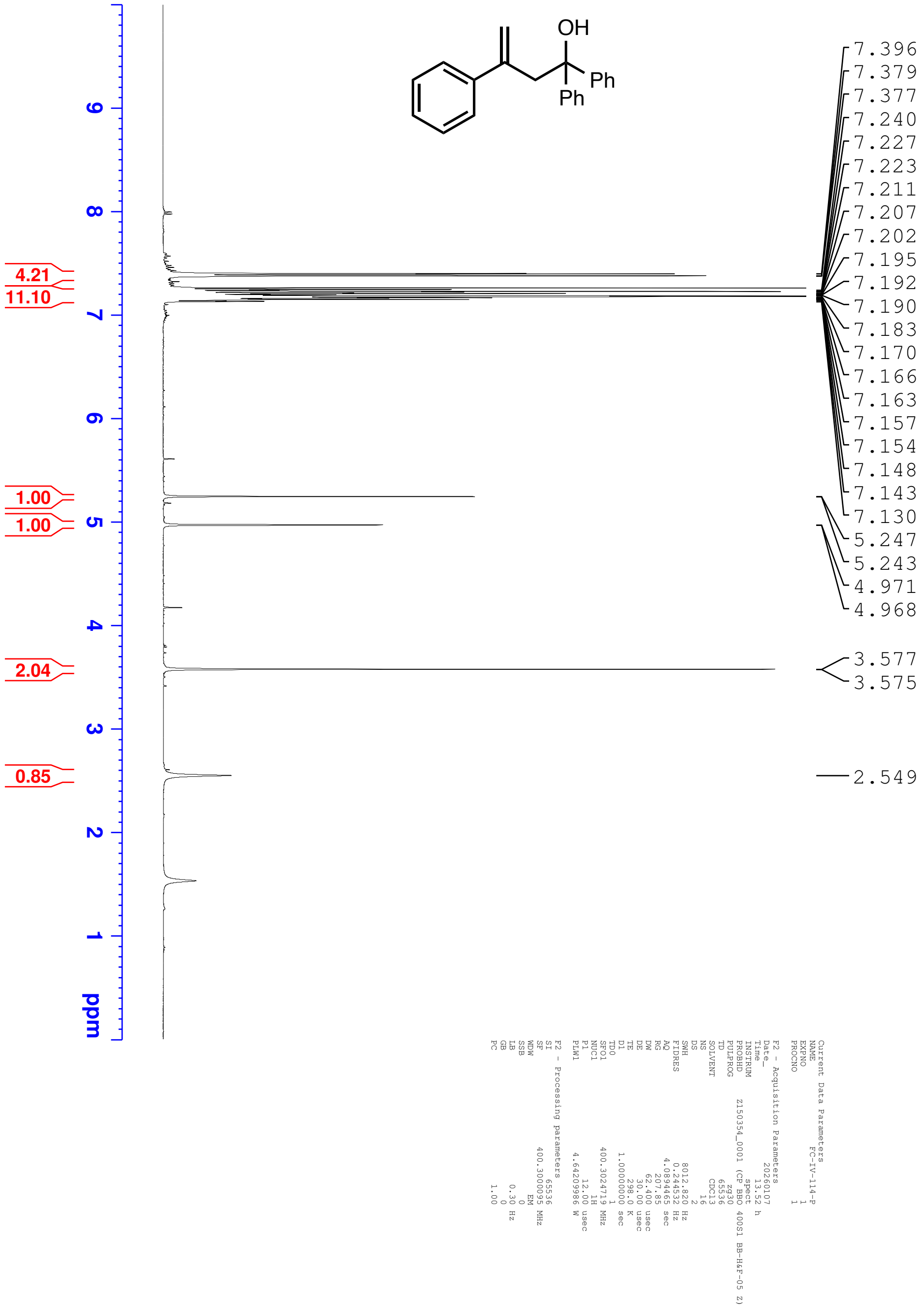

1,1,3-Triphenylbut-3-en-1-ol (S6).

Solvent: $CDCl_3$
100 MHz
$^{13}C\{^{1}H\}$ NMR

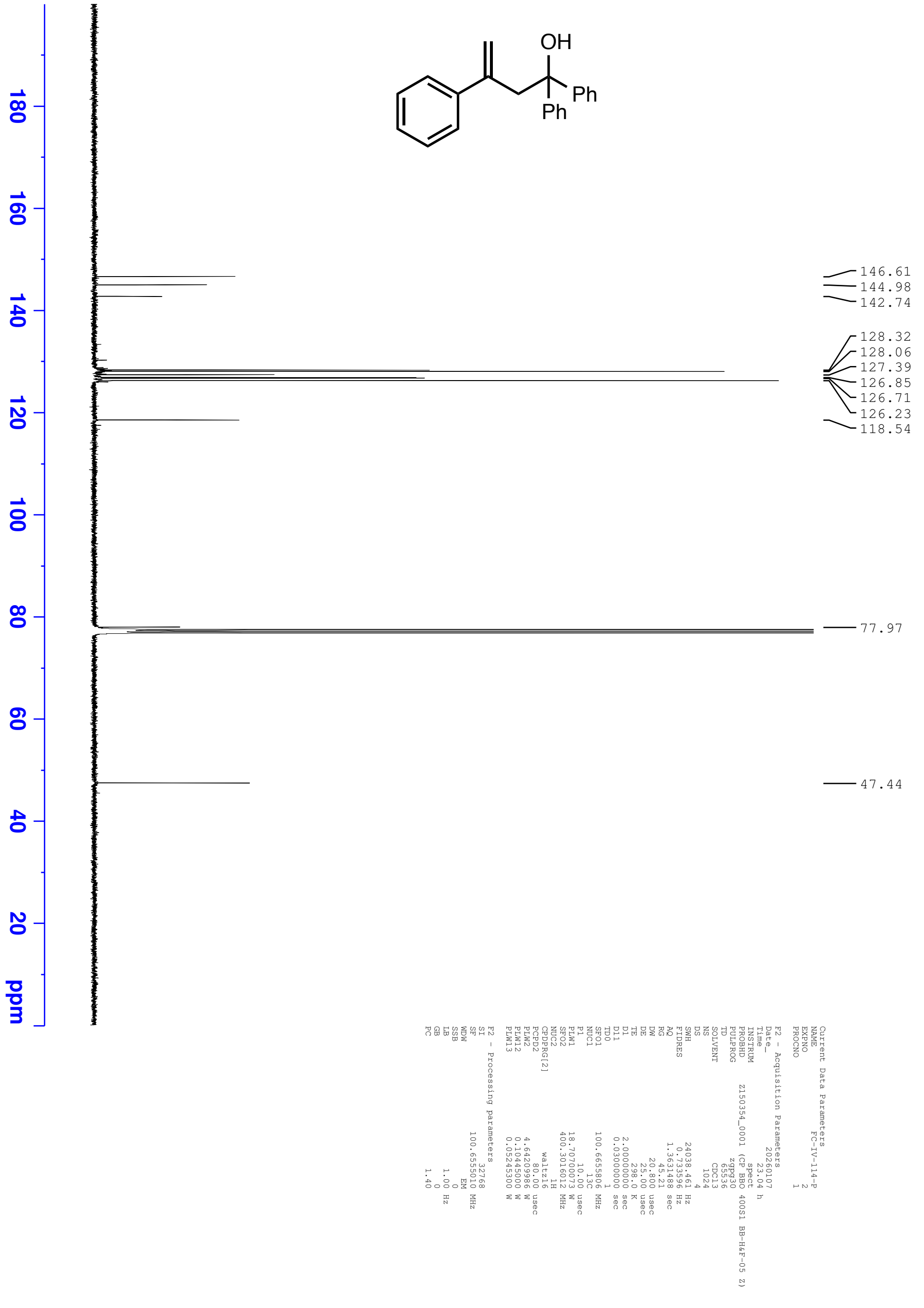

4-([1,1'-Biphenyl]-4-yl)-2-methylpent-4-en-2-ol (S7).

Solvent: $CDCl_3$
400 MHz
^{1}H NMR

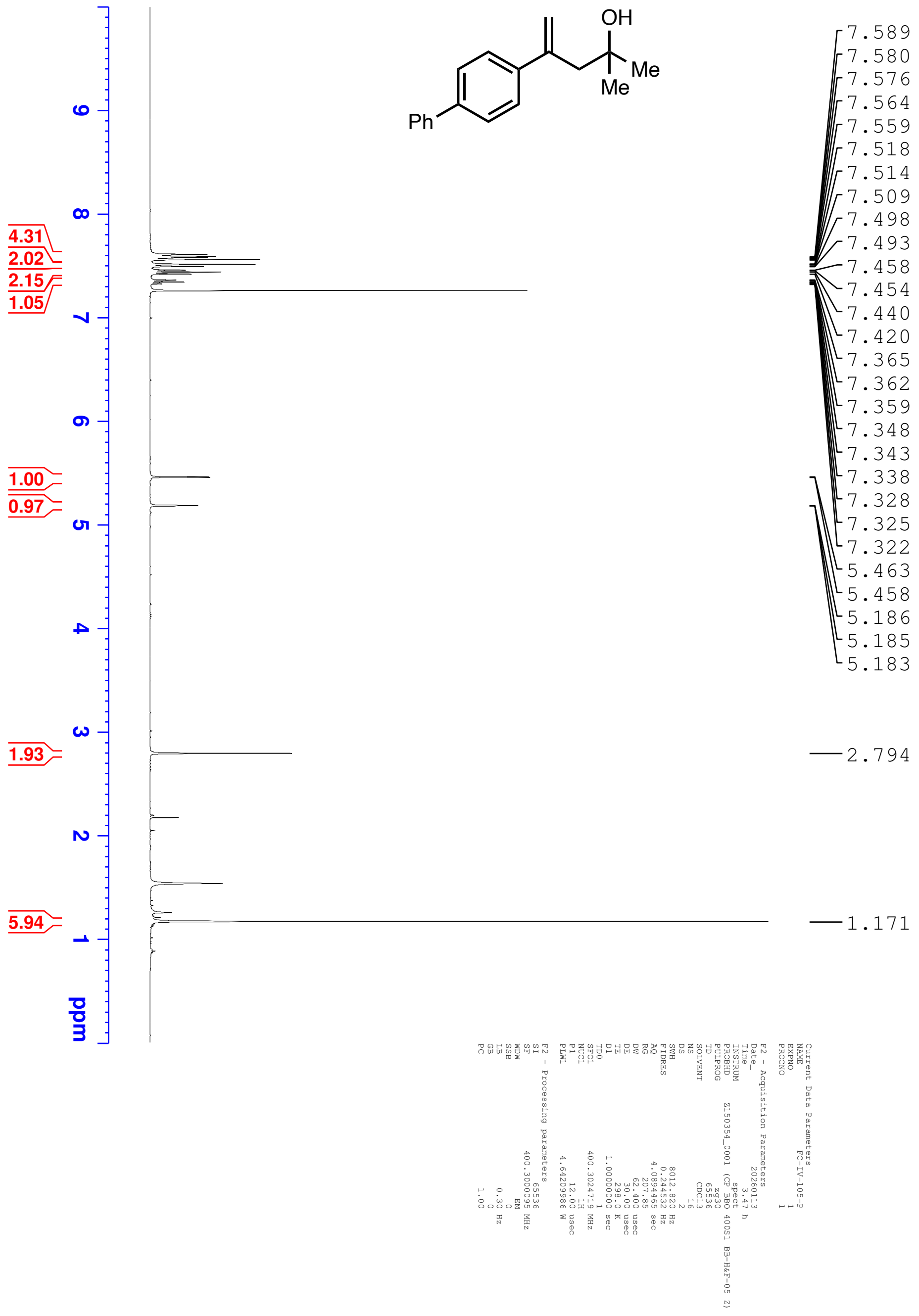

4-([1,1'-Biphenyl]-4-yl)-2-methylpent-4-en-2-ol (S7).

Solvent: $CDCl_3$
100 MHz
$^{13}C\{^{1}H\}$ NMR

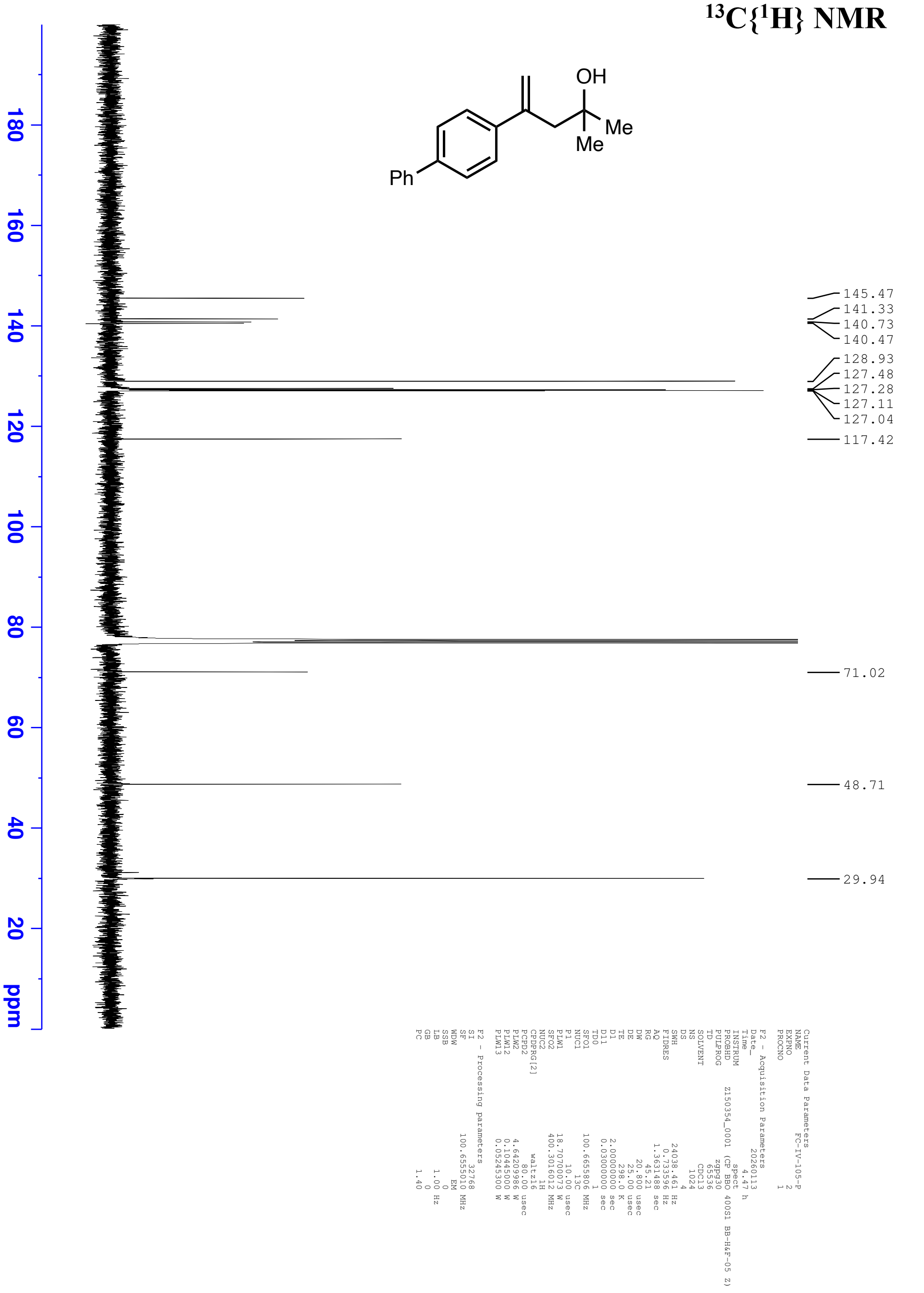

4-(3,5-Bis(trifluoromethyl)phenyl)-2-methylpent-3-en-2-ol (S9).

Solvent: $CDCl_3$
400 MHz
^1H NMR

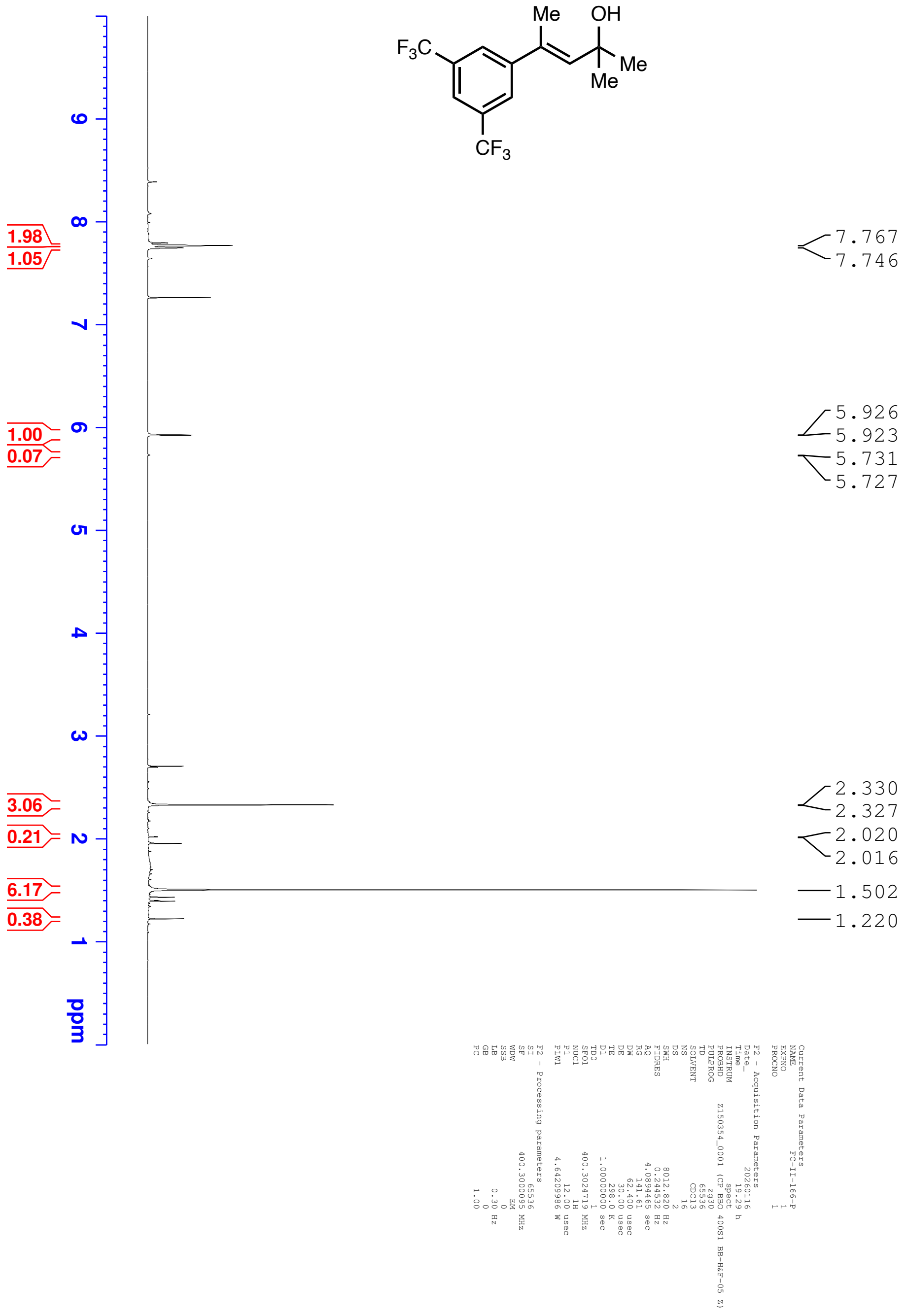

4-(3,5-Bis(trifluoromethyl)phenyl)-2-methylpent-3-en-2-ol (S9).

Solvent: $CDCl_3$
100 MHz
$^{13}C\{^{1}H\}$ NMR

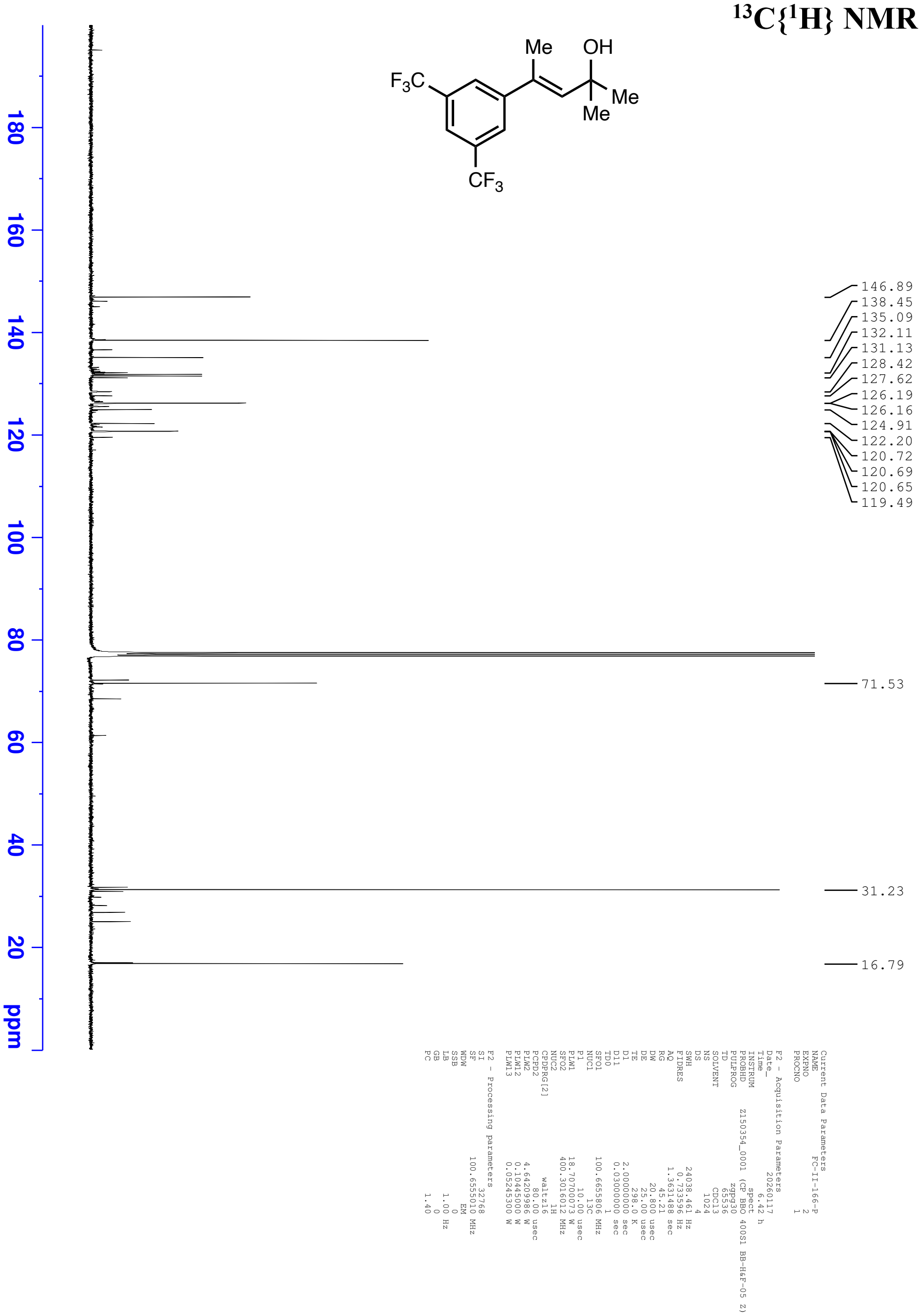

4-(3,5-Bis(trifluoromethyl)phenyl)-2-methylpent-3-en-2-ol (S9).

Solvent: $CDCl_3$
377 MHz
$^{19}F\{^{1}H\}$ NMR

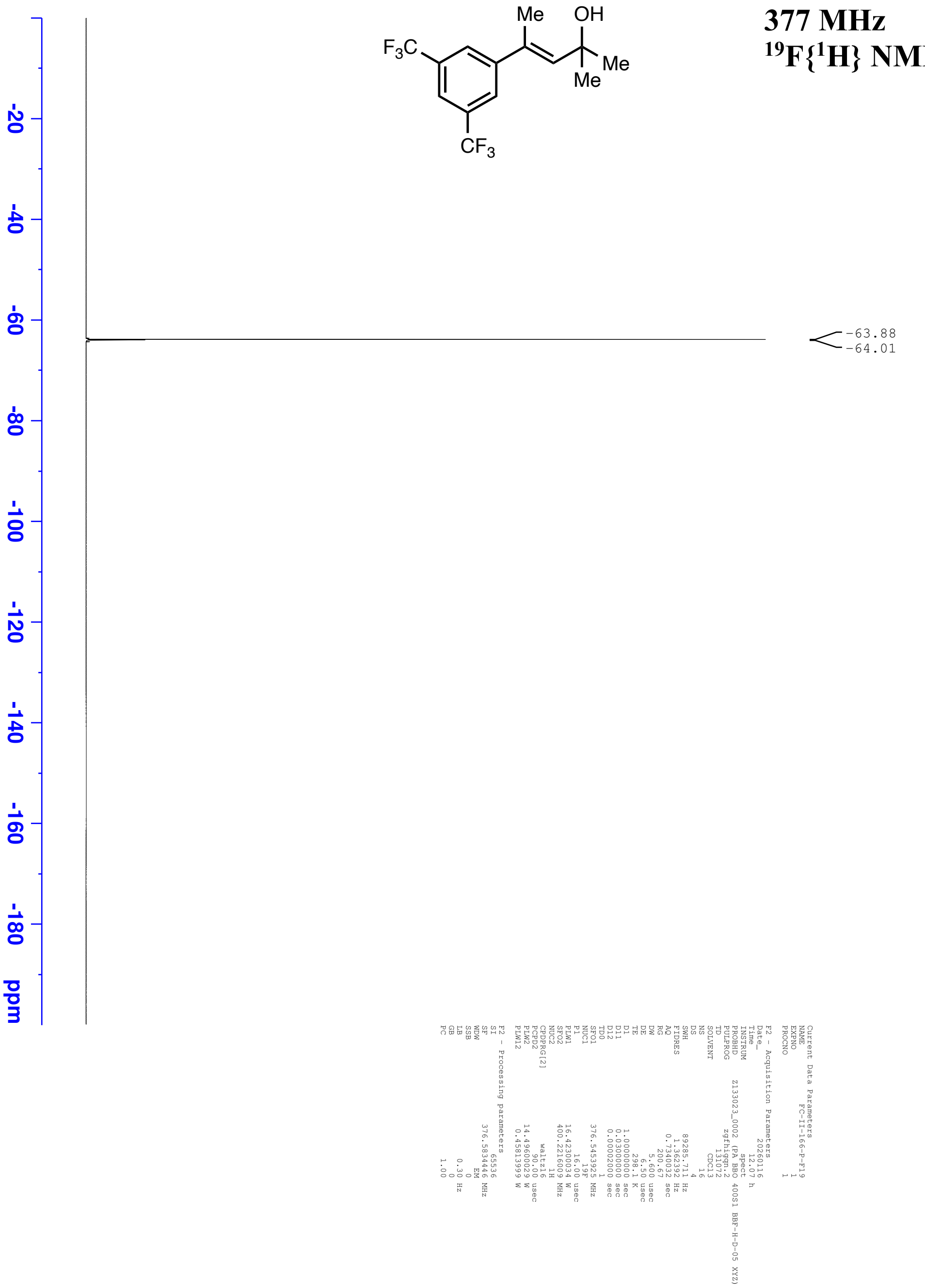

3-(3,5-Bis(trifluoromethyl)phenyl)-1,1-diphenylbut-2-en-1-ol (S10).

Solvent: $CDCl_3$
400 MHz
^{1}H NMR

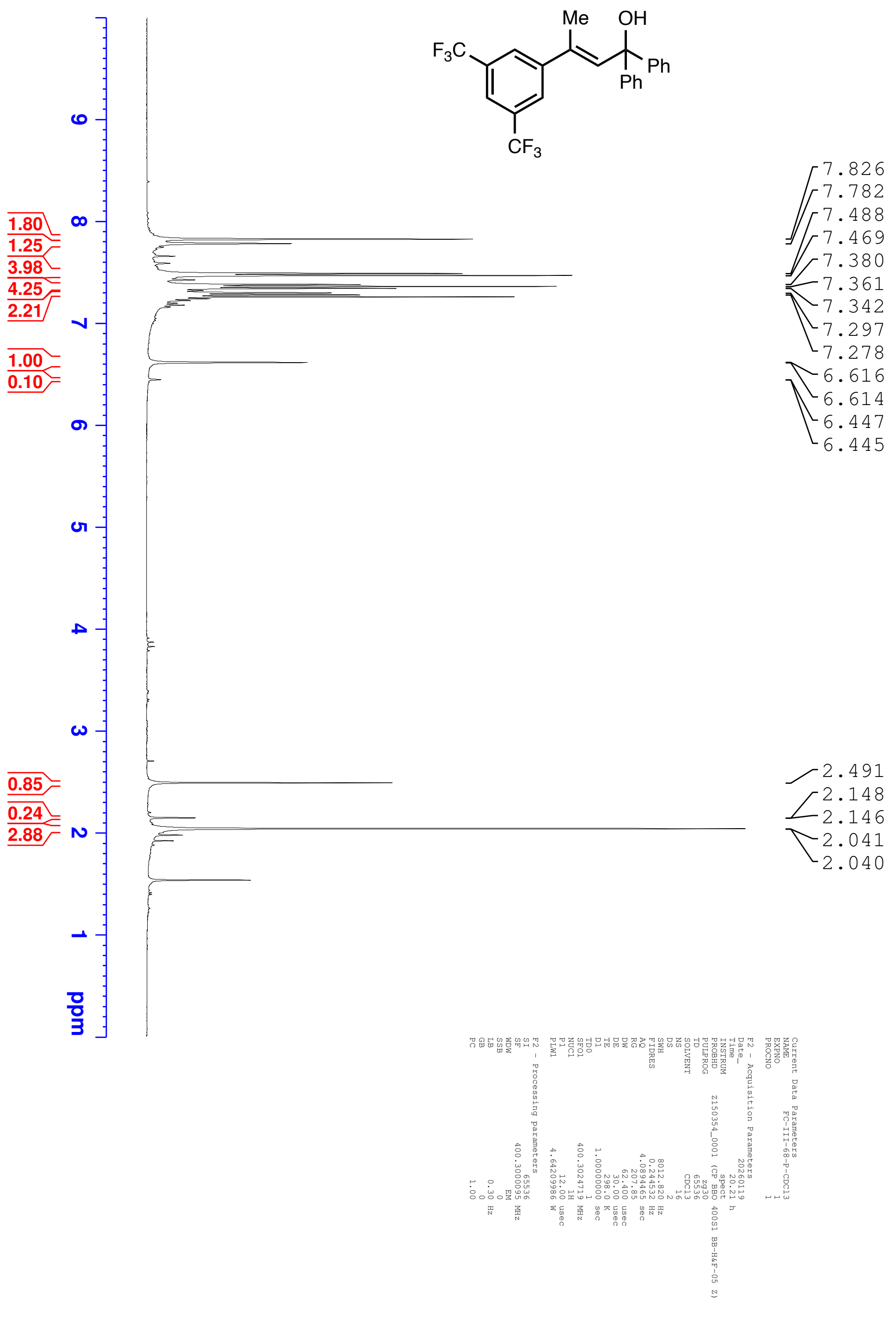

3-(3,5-Bis(trifluoromethyl)phenyl)-1,1-diphenylbut-2-en-1-ol (S10).

Solvent: $CDCl_3$
100 MHz
$^{13}C\{^1H\}$ NMR

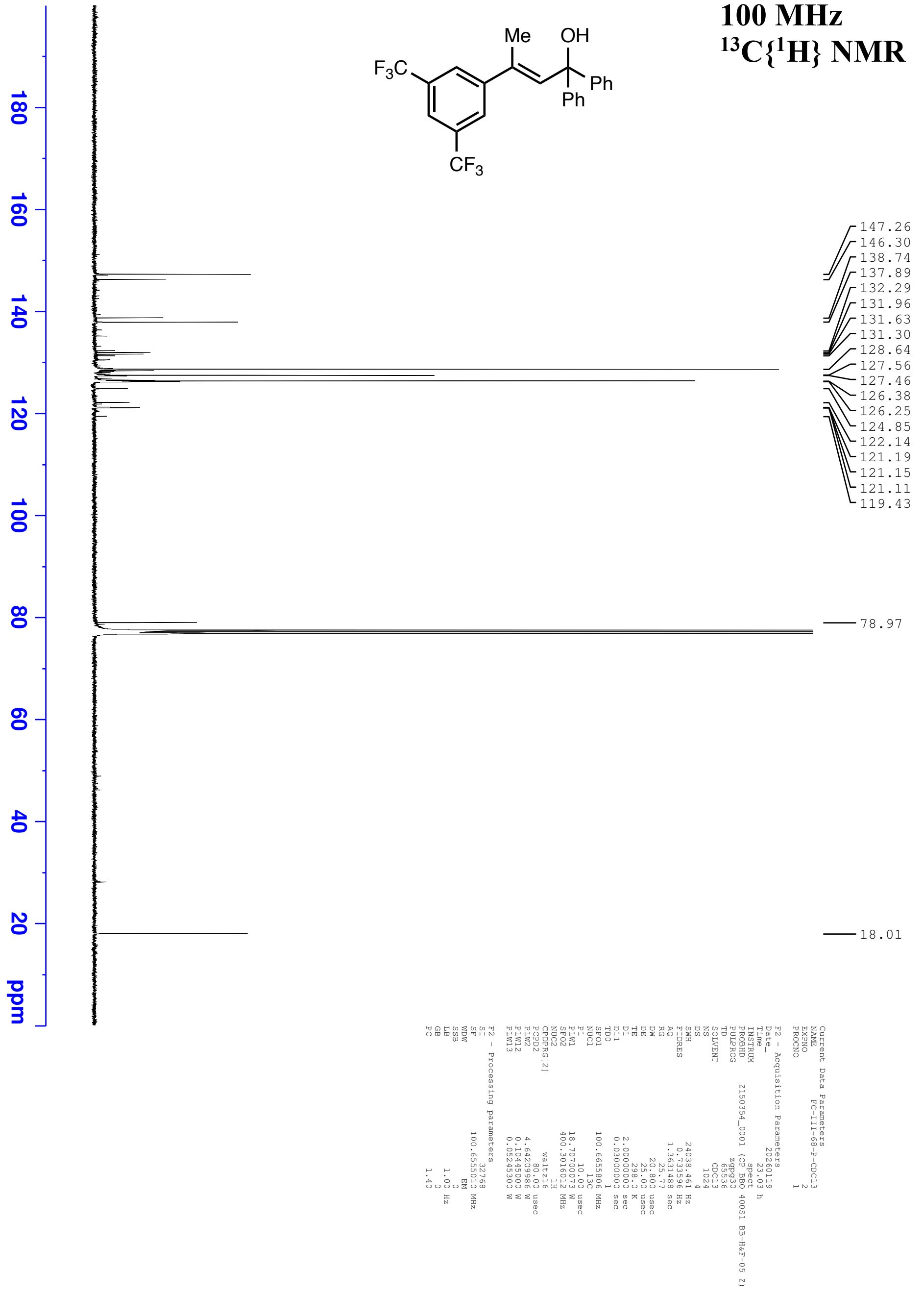

3-(3,5-Bis(trifluoromethyl)phenyl)-1,1-diphenylbut-2-en-1-ol (S10).

Solvent: $CDCl_3$
377 MHz
$^{19}F\{^1H\}$ NMR

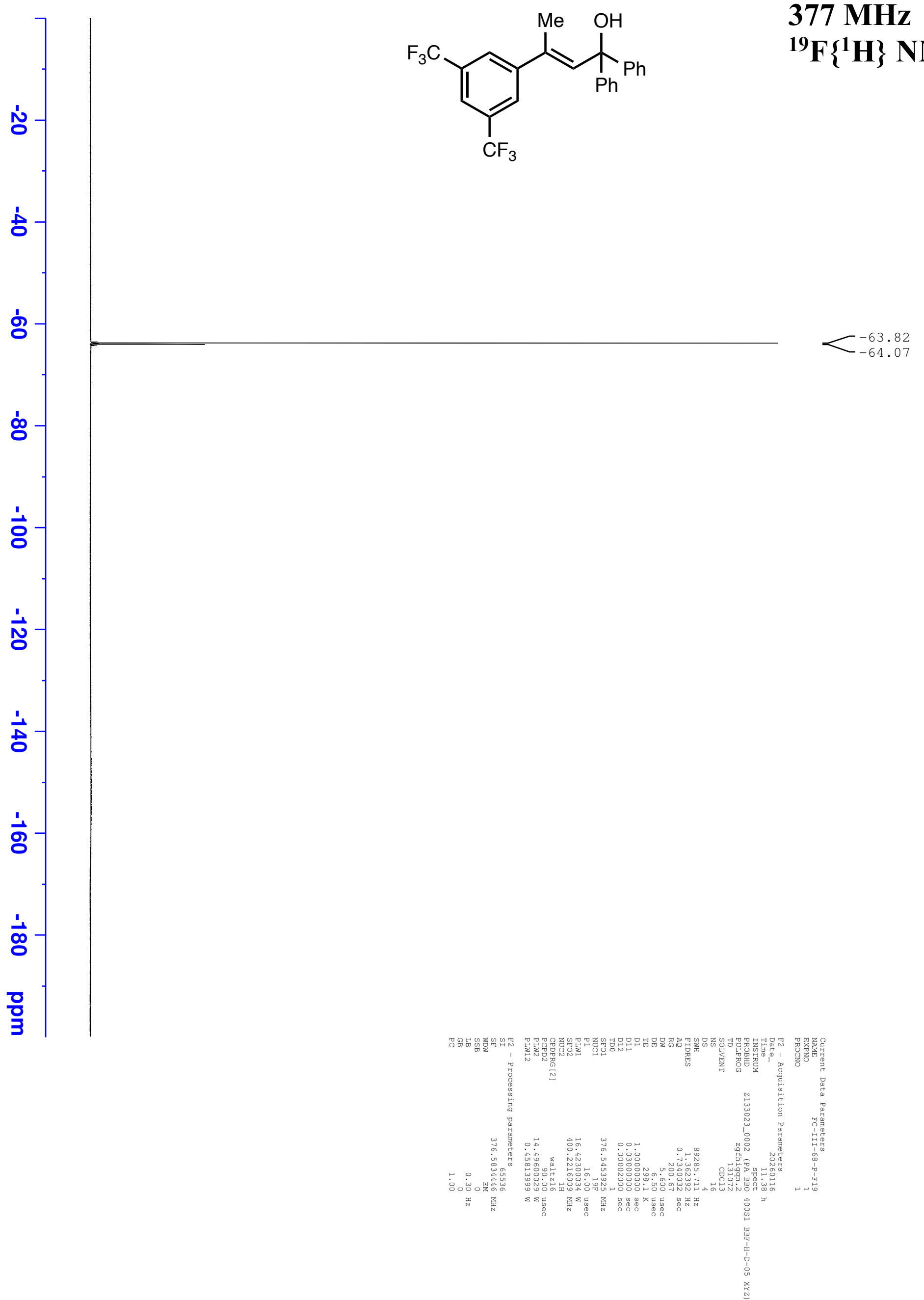

3-Methyl-1-phenylbut-3-en-1-ol (S11).*

Solvent: $CDCl_3$
400 MHz
^{1}H NMR

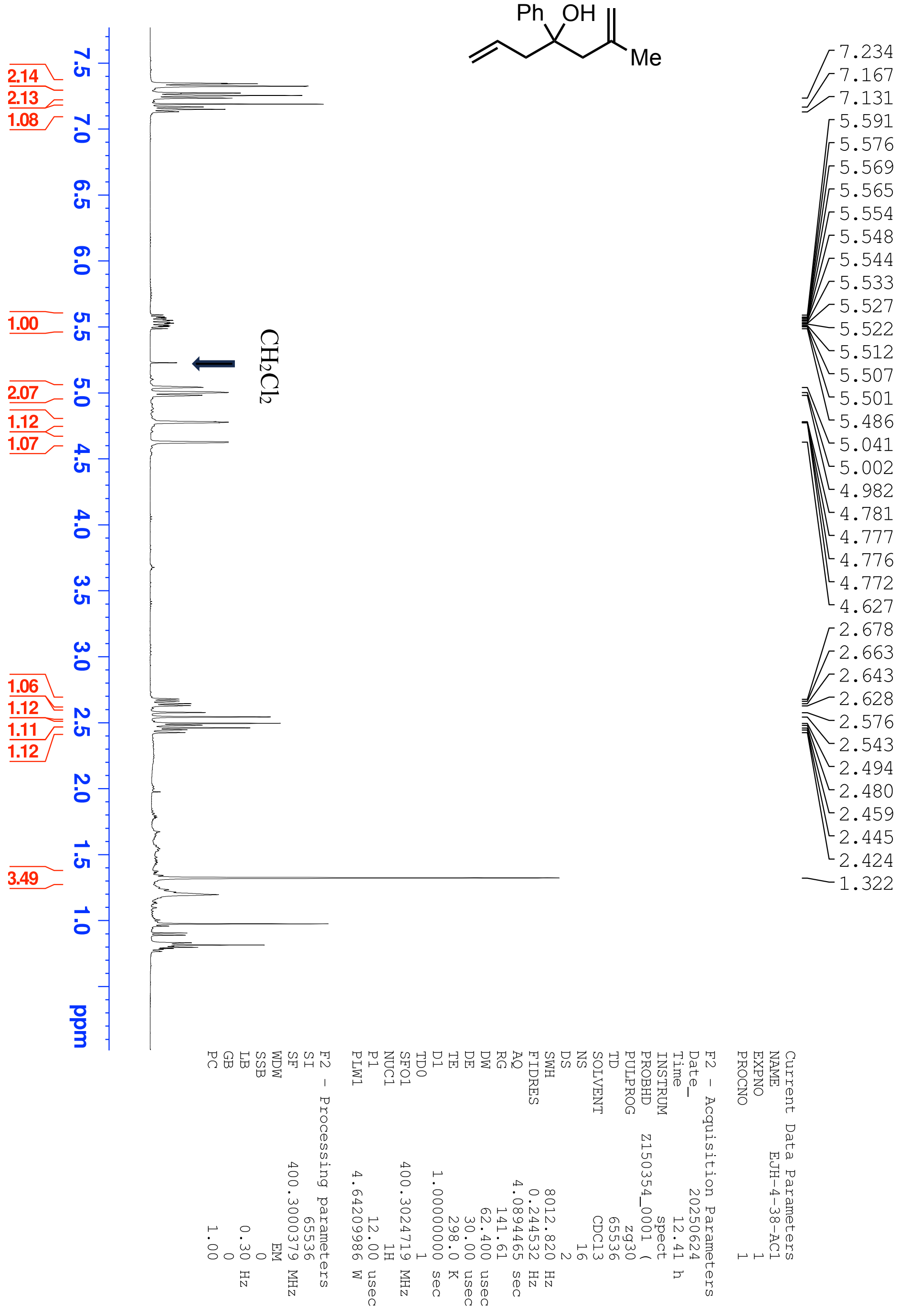

*S11 was brought forward to the next reaction as a crude mixture.

3-Methyl-1-phenylbut-3-en-1-ol (S11). [*]

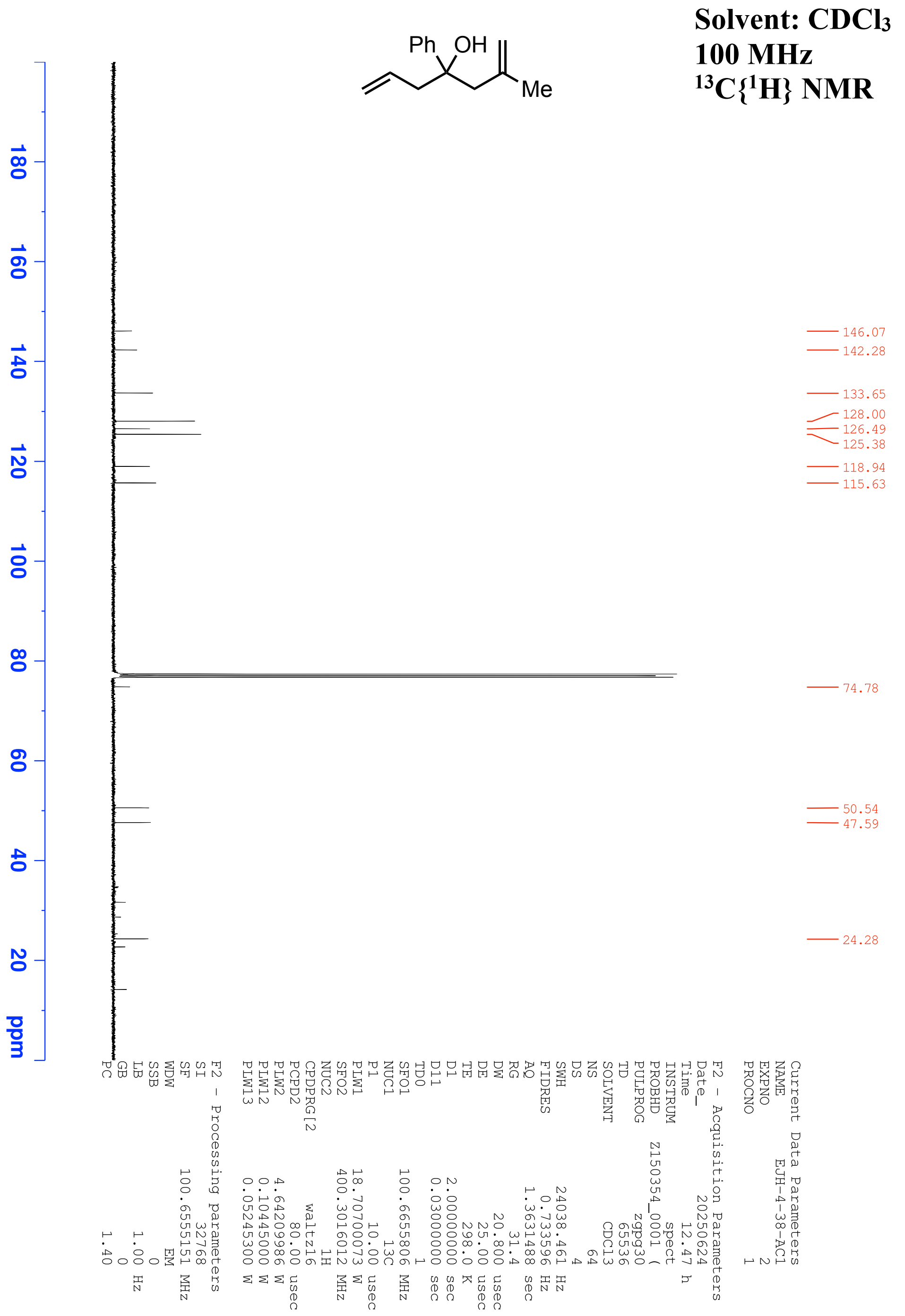

*[*] **S11** was brought forward to the next reaction as a crude mixture.

4-(3,5-Bis(trifluoromethyl)phenyl)-4-hydroperoxy-2-methylpentan-2-ol (S14).

Solvent: $CDCl_3$
400 MHz
^{1}H NMR

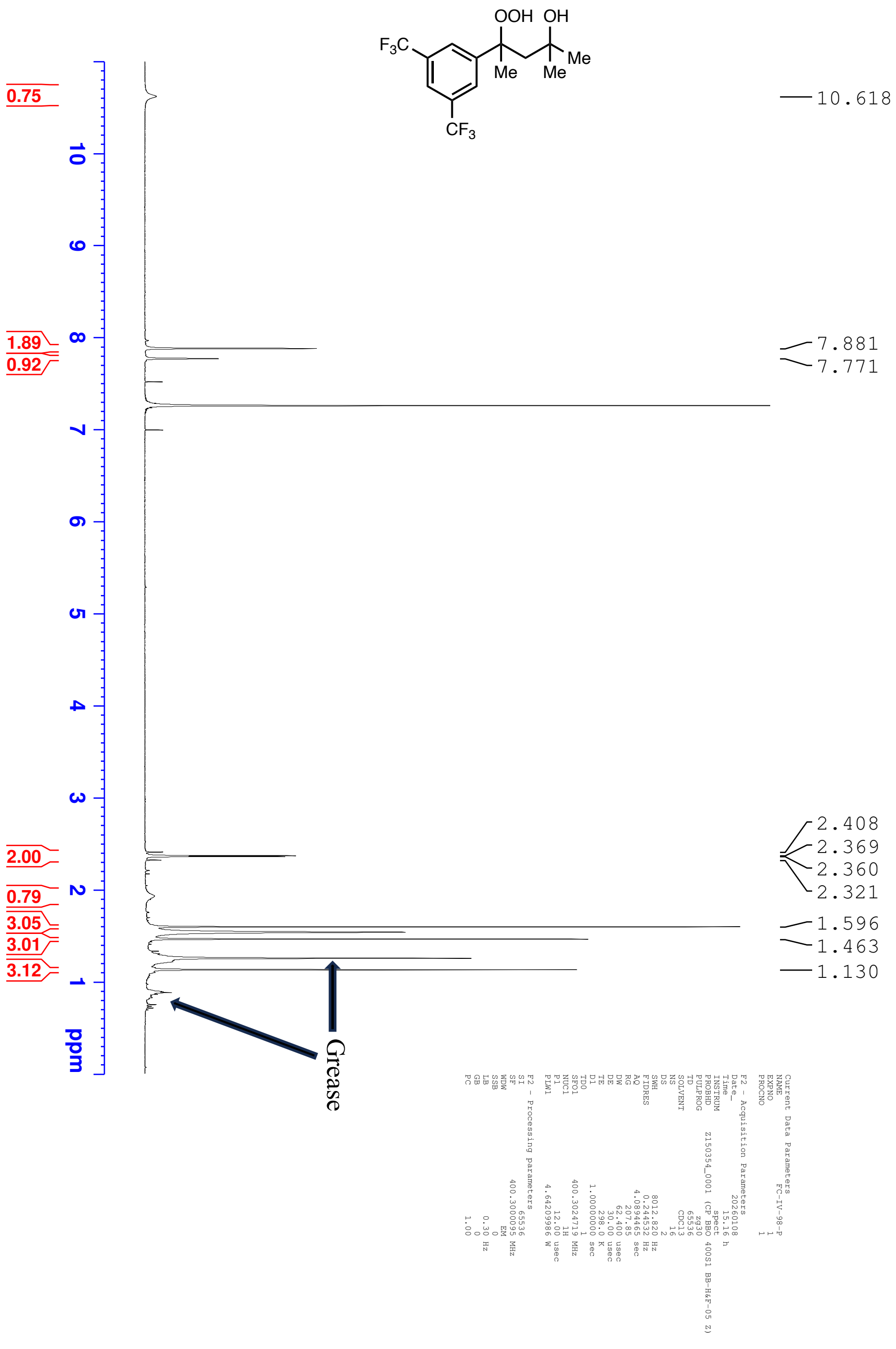

4-(3,5-Bis(trifluoromethyl)phenyl)-4-hydroperoxy-2-methylpentan-2-ol (S14).

Solvent: $CDCl_3$
100 MHz
$^{13}C\{^1H\}$ NMR

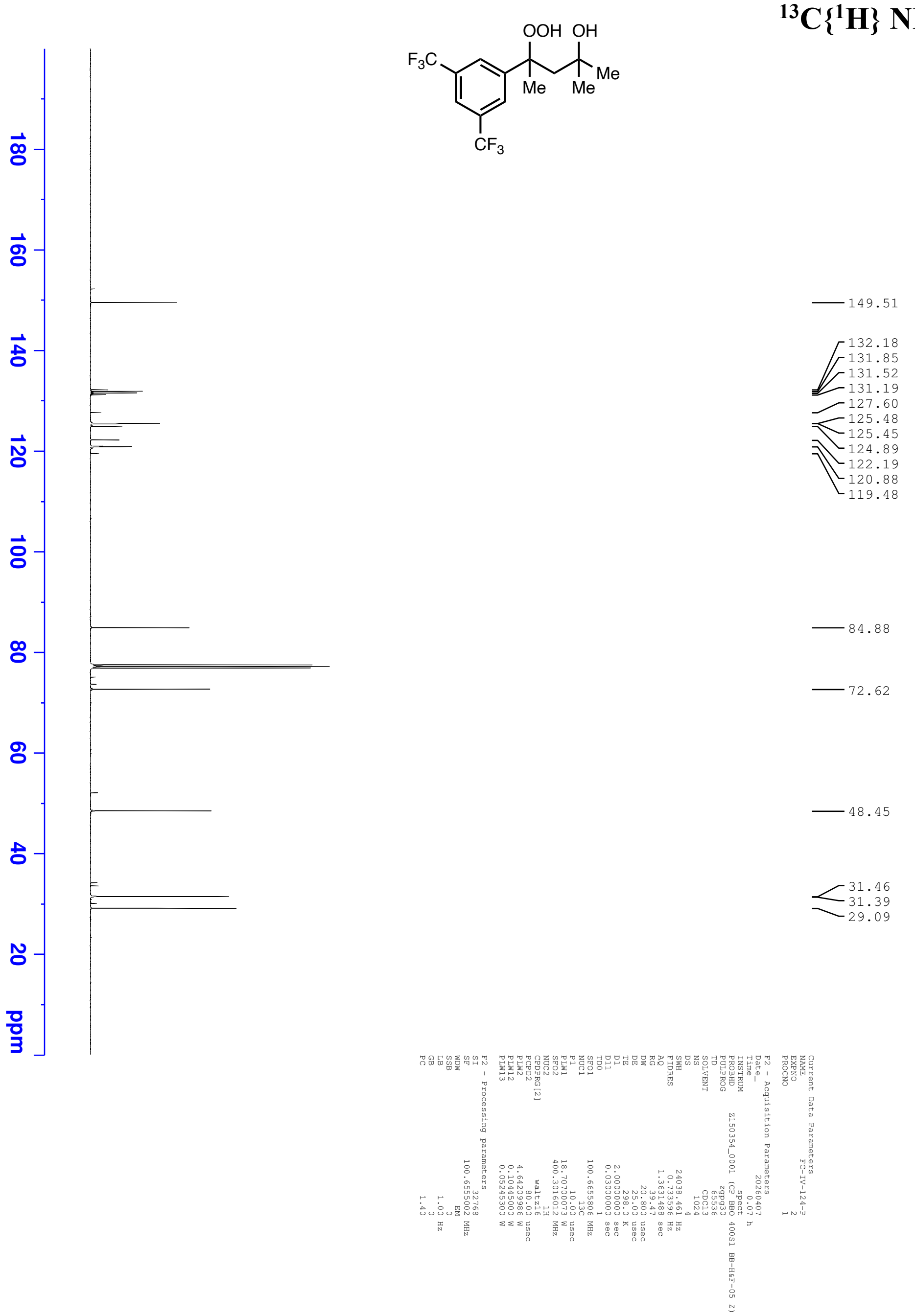

4-(3,5-Bis(trifluoromethyl)phenyl)-4-hydroperoxy-2-methylpentan-2-ol (S14).

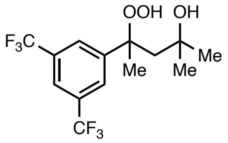

Solvent: $CDCl_3$
377 MHz
$^{19}F\{^1H\}$ NMR

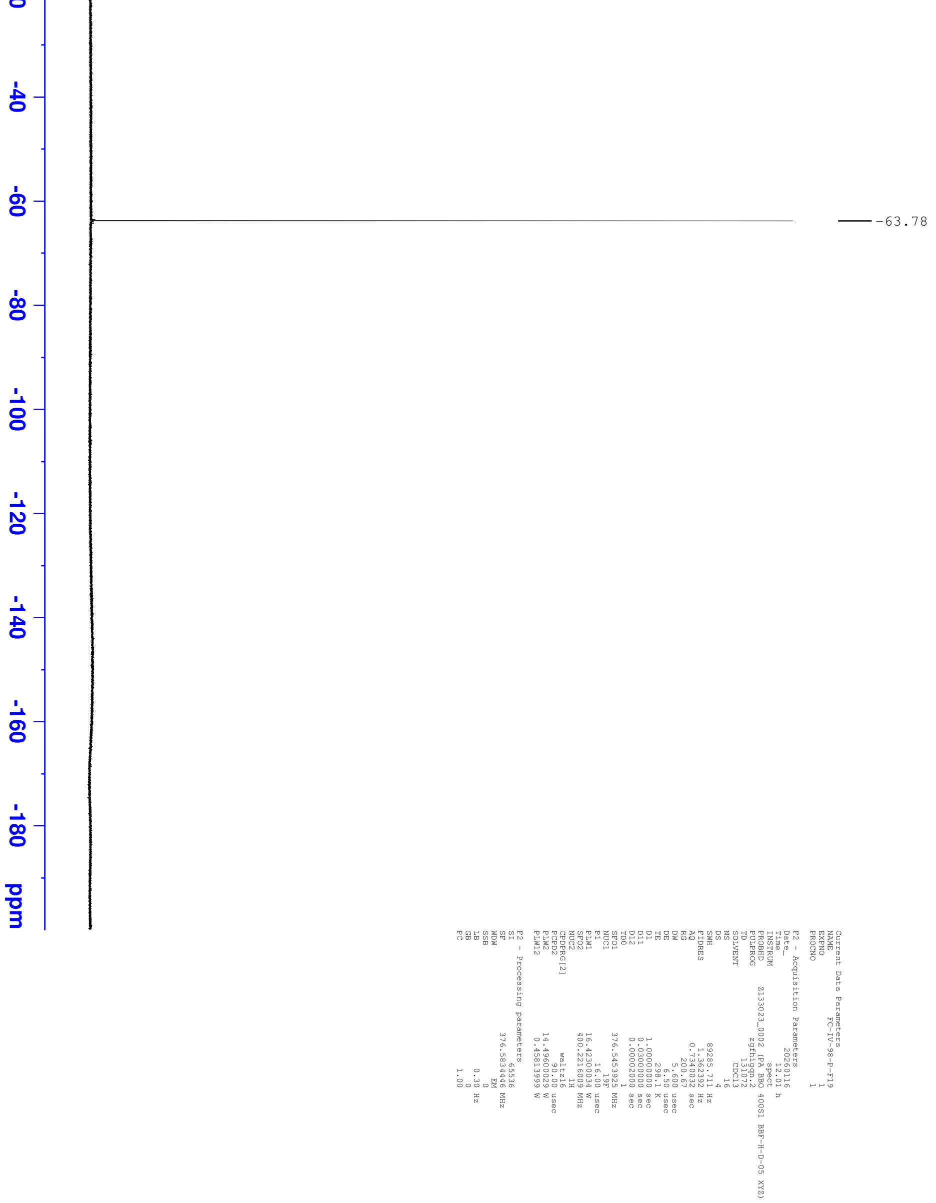

3-(3,5-Bis(trifluoromethyl)phenyl)-3-hydroperoxy-1,1-diphenylbutan-1-ol (S15).

Solvent: $CDCl_3$
400 MHz
^{1}H NMR

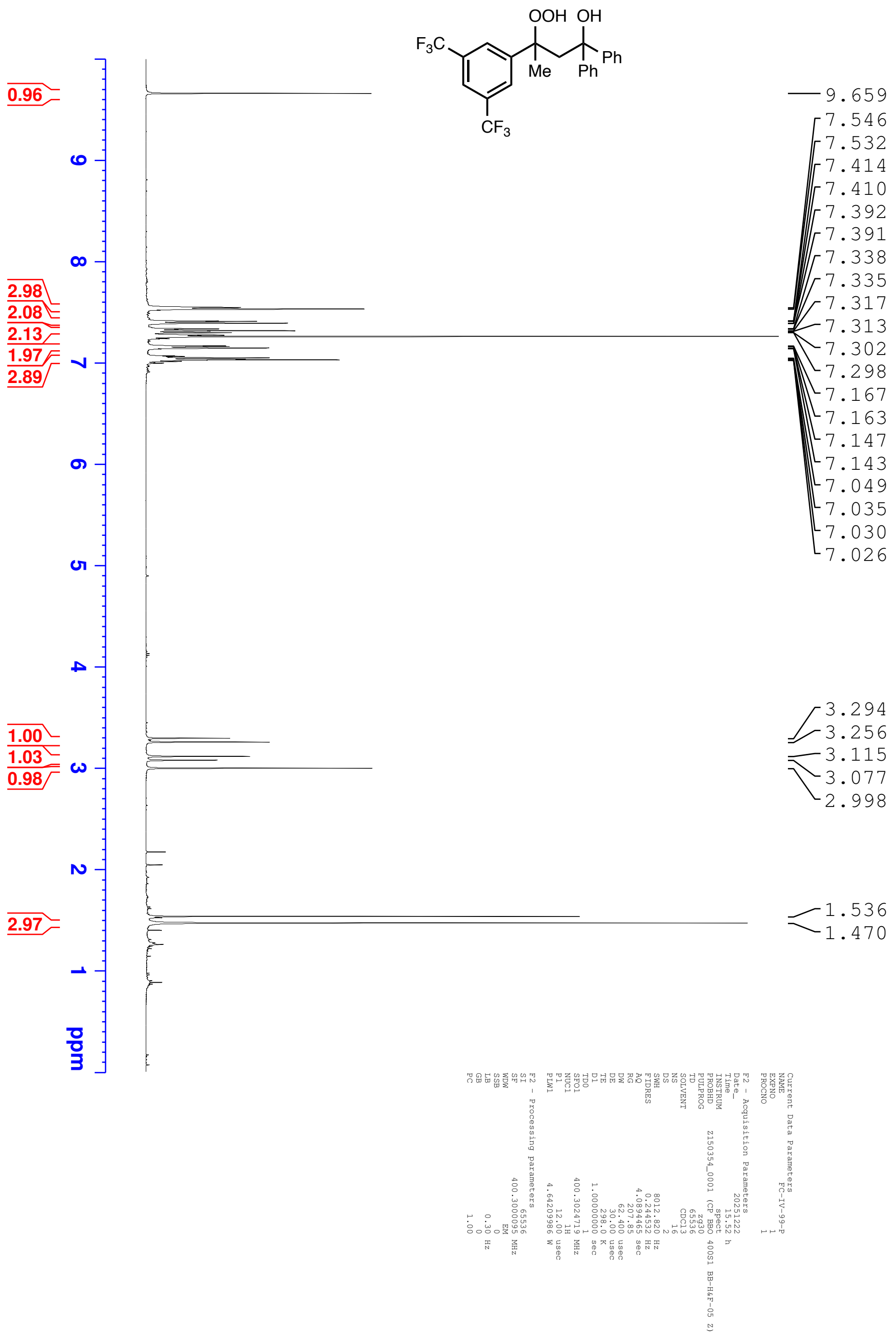

3-(3,5-Bis(trifluoromethyl)phenyl)-3-hydroperoxy-1,1-diphenylbutan-1-ol (S15).

Solvent: $CDCl_3$
100 MHz
$^{13}C\{^1H\}$ NMR

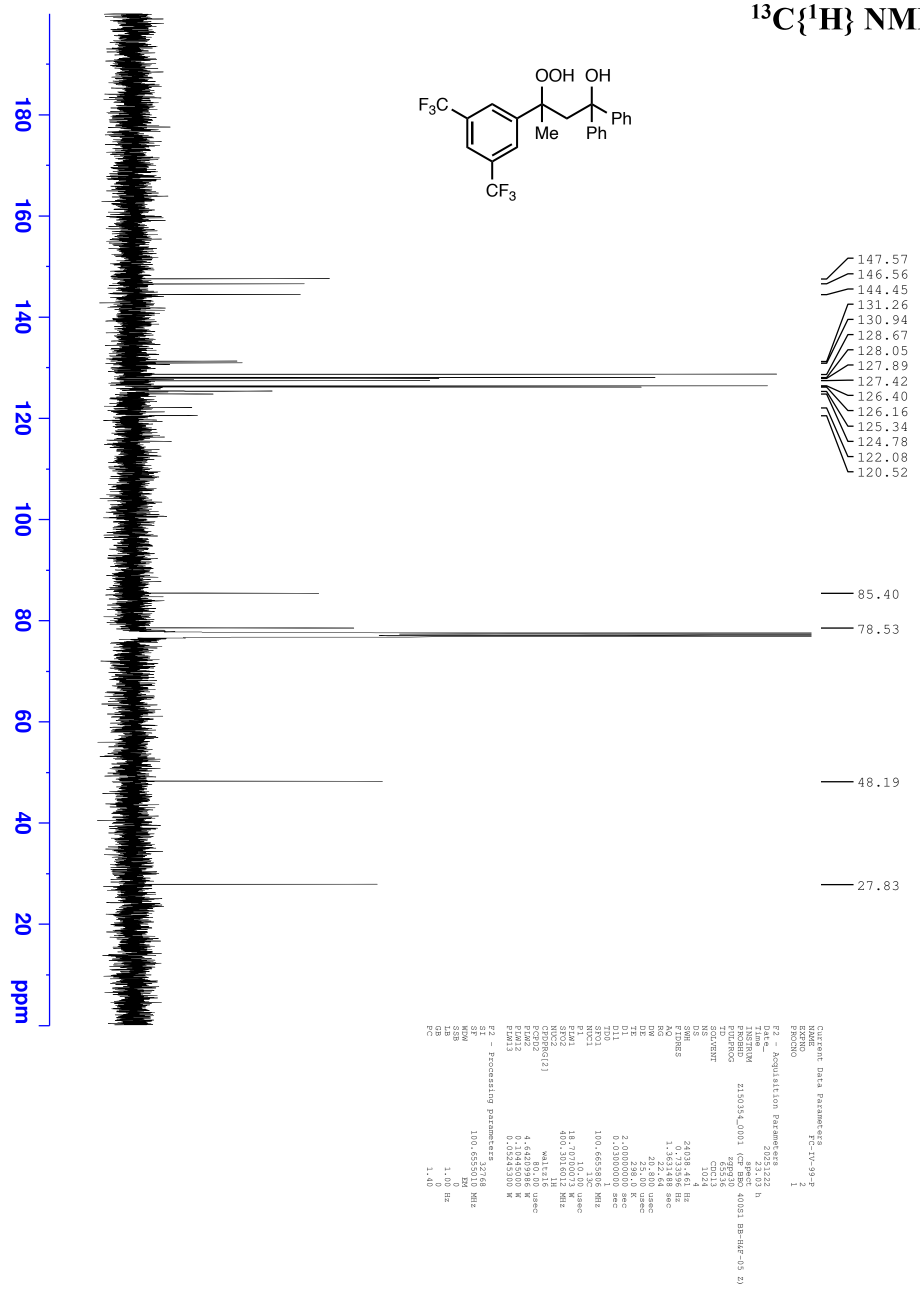

3-(3,5-Bis(trifluoromethyl)phenyl)-3-hydroperoxy-1,1-diphenylbutan-1-ol (S15).

Solvent: $CDCl_3$
377 MHz
$^{19}F\{^1H\}$ NMR

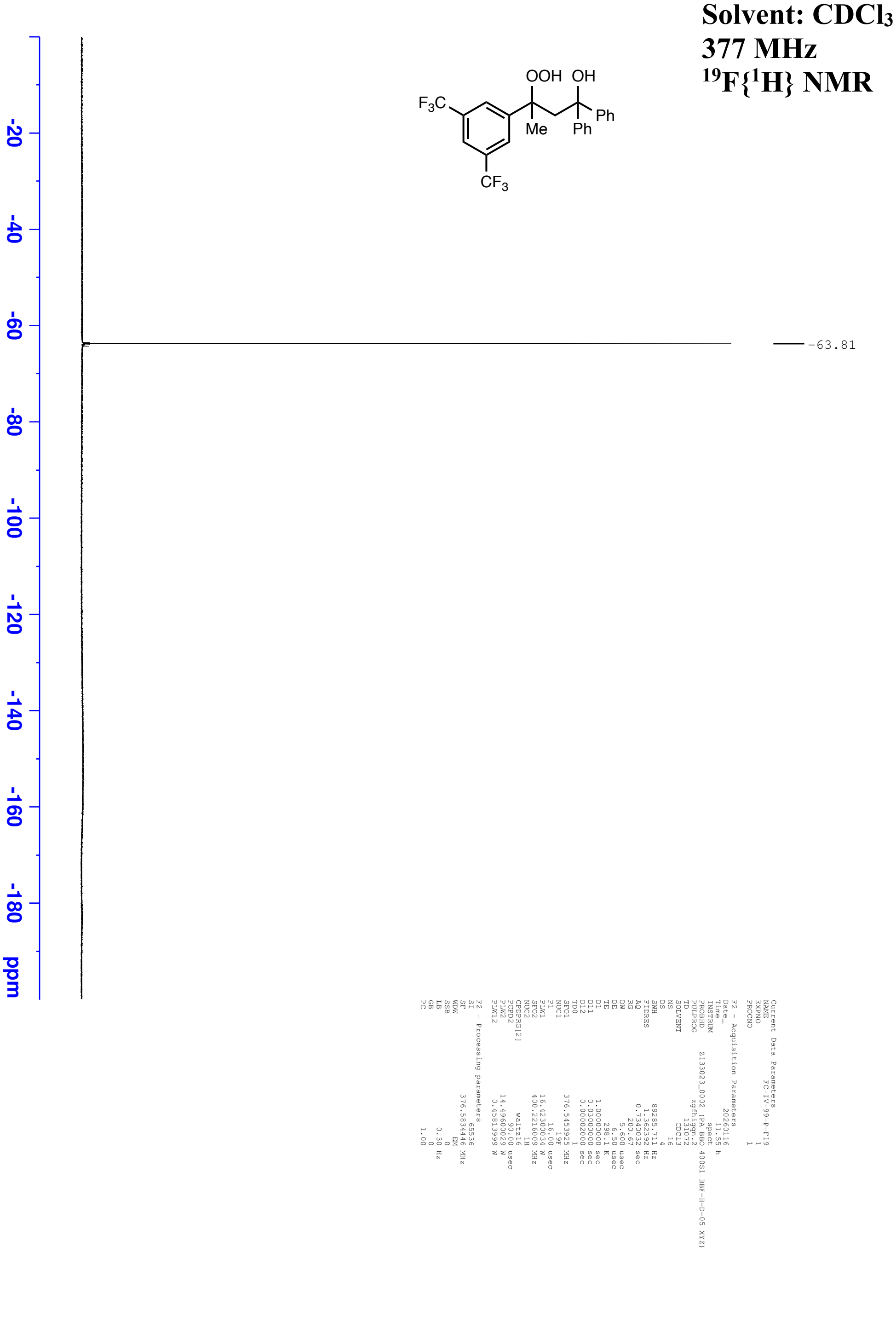

Solvent: $CDCl_3$
400 MHz
^{1}H NMR

4-([1,1'-Biphenyl]-4-yl)-4-hydroperoxy-2-methylpentan-2-ol (S16).

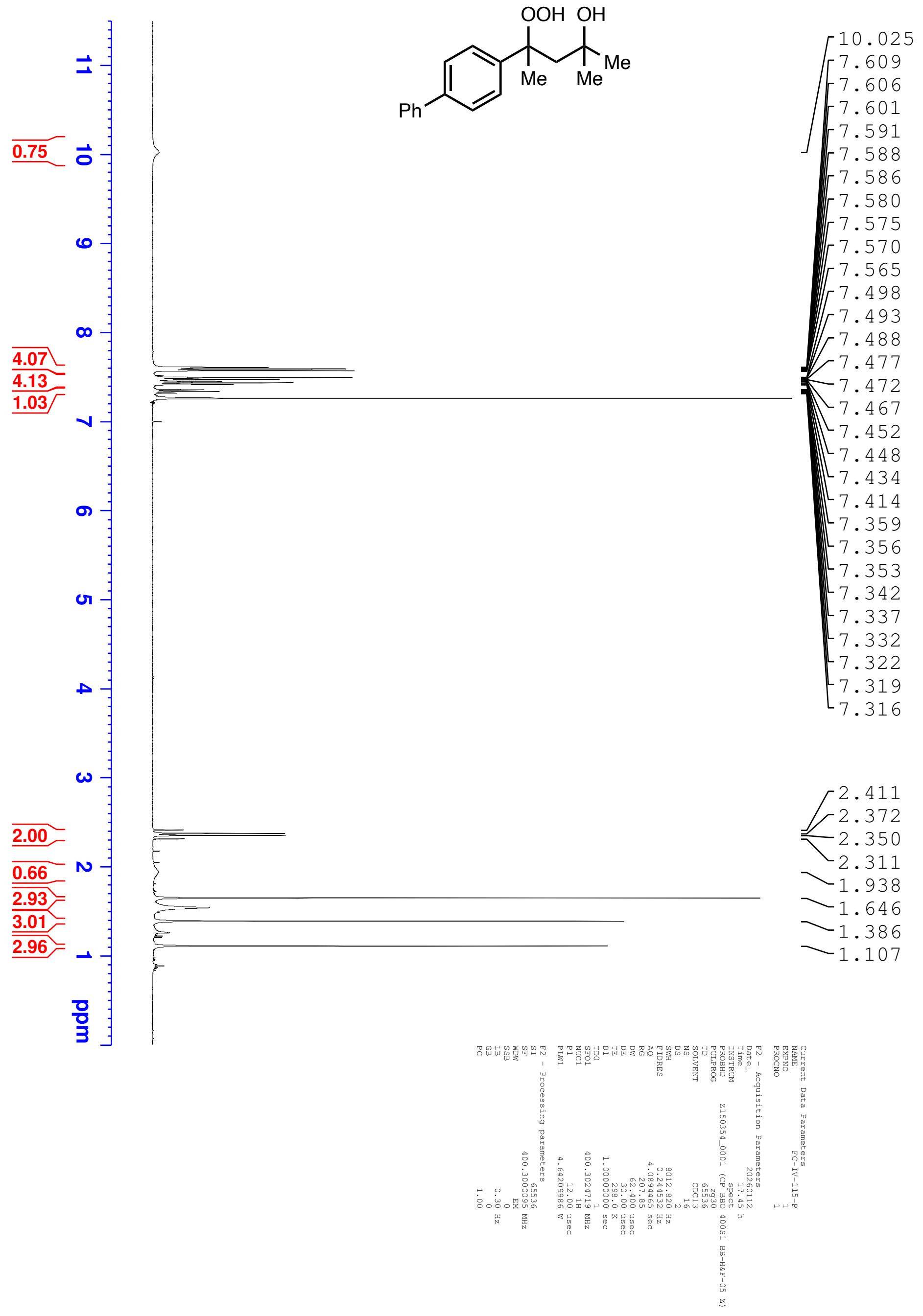

4-([1,1'-Biphenyl]-4-yl)-4-hydroperoxy-2-methylpentan-2-ol (S16).

Solvent: $CDCl_3$
100 MHz
$^{13}C\{^{1}H\}$ NMR

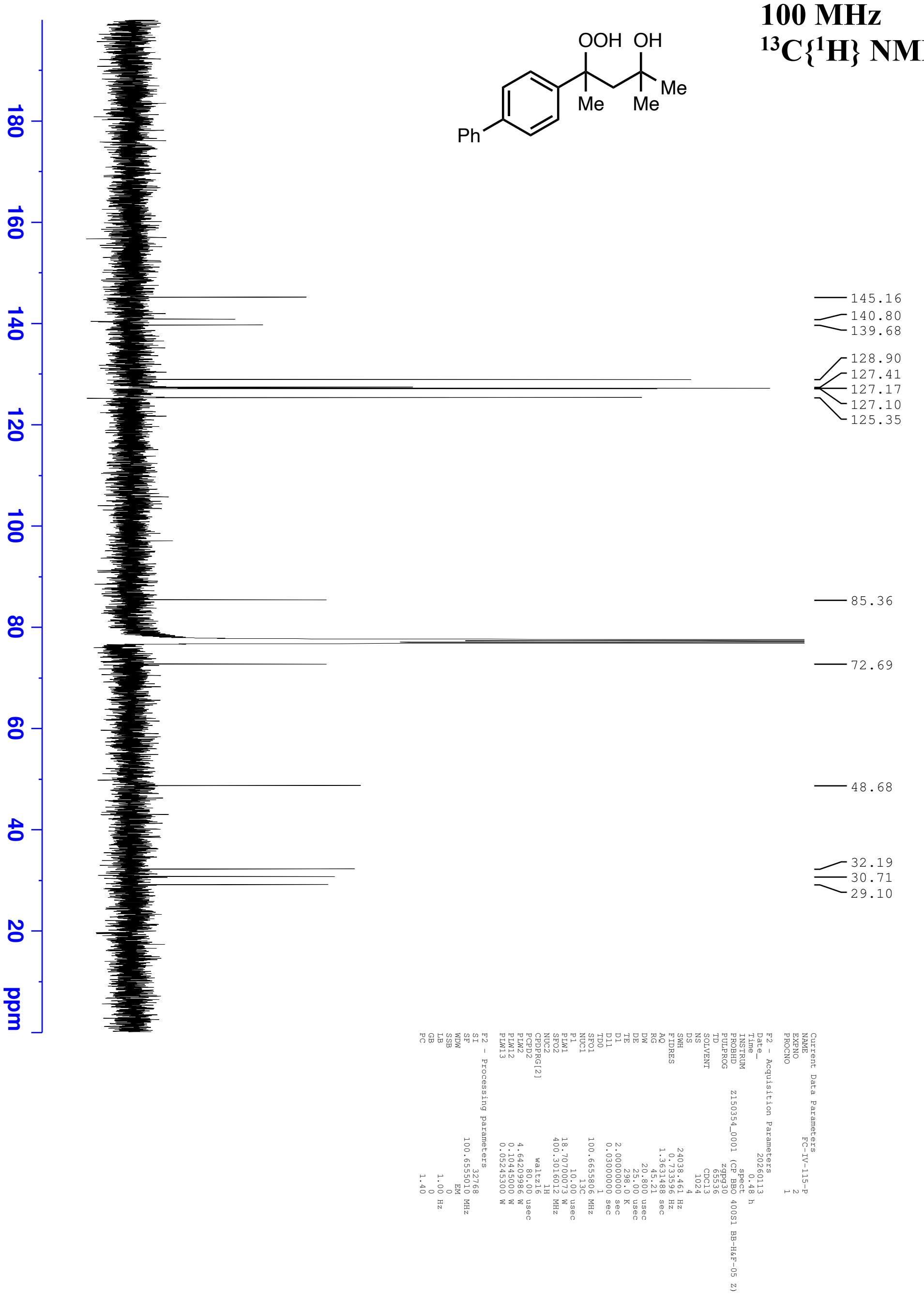

3-Hydroperoxy-1,1,3-triphenylbutan-1-ol (S17).

Solvent: $CDCl_3$
400 MHz
^{1}H NMR

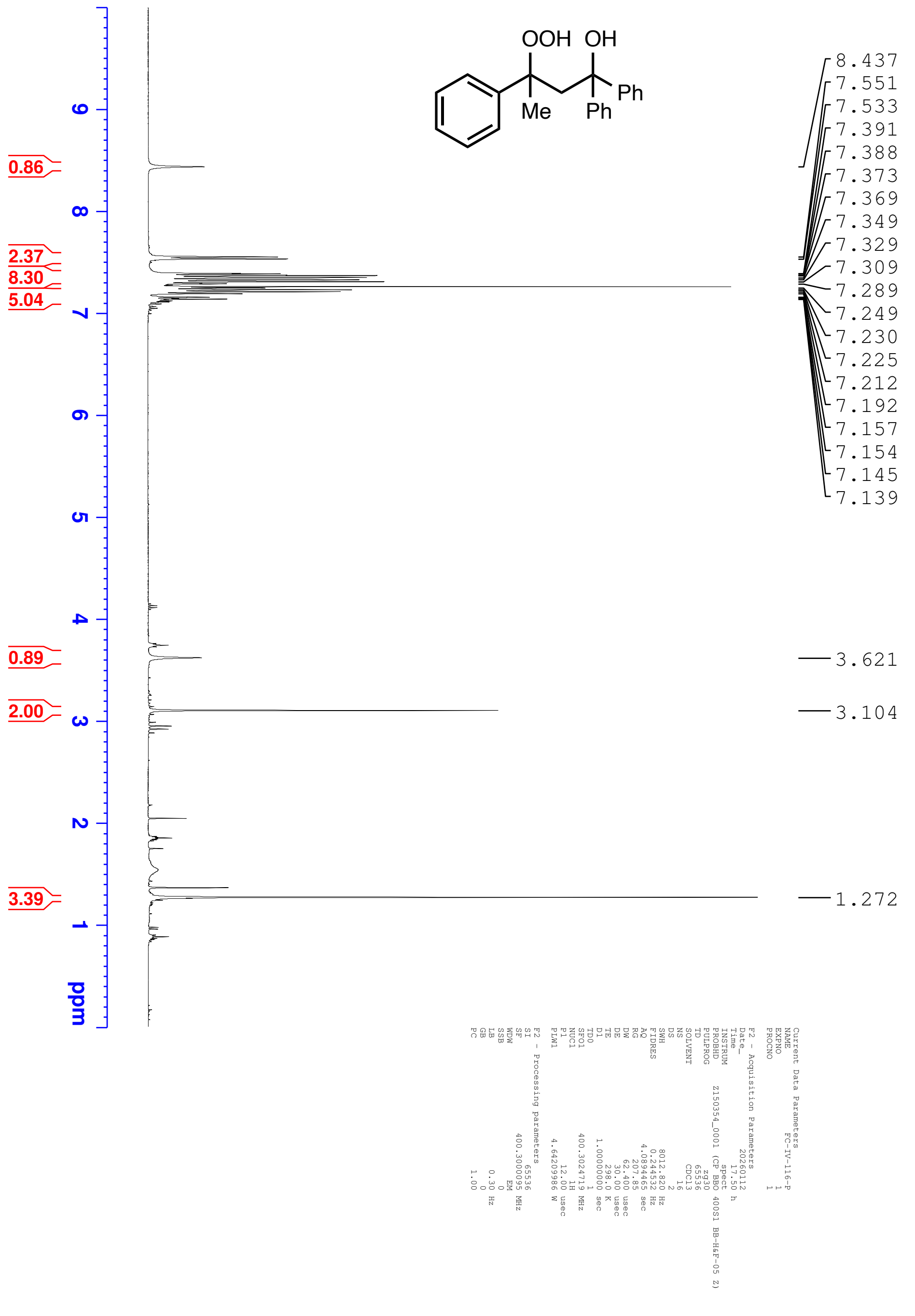

3-Hydroperoxy-1,1,3-triphenylbutan-1-ol (S17).

Solvent: $CDCl_3$
100 MHz
$^{13}C\{^1H\}$ NMR

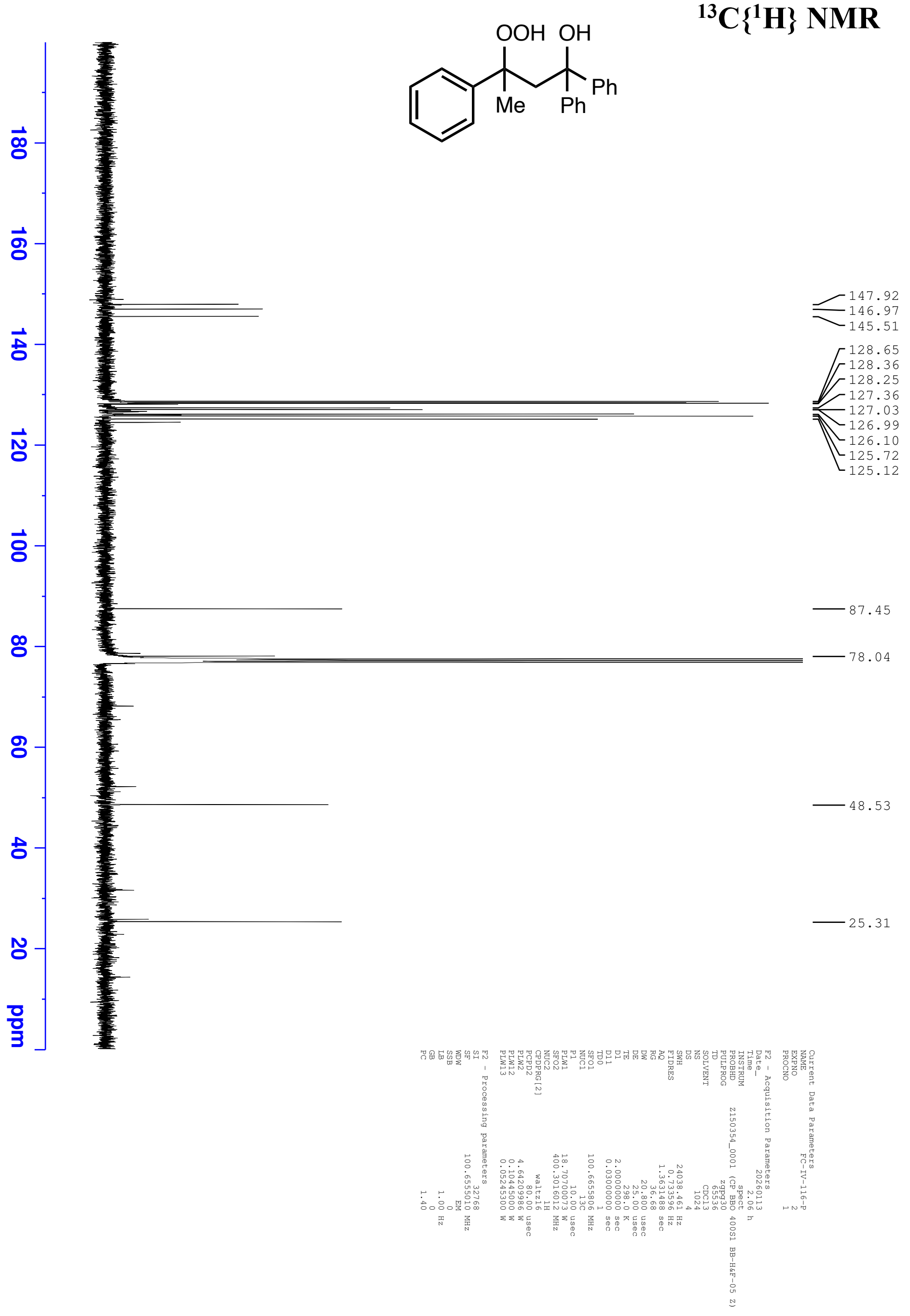

6-Hydroperoxy-6-methyl-4-phenylhept-1-en-4-ol (S18).

Solvent: $CDCl_3$
400 MHz
^{1}H NMR

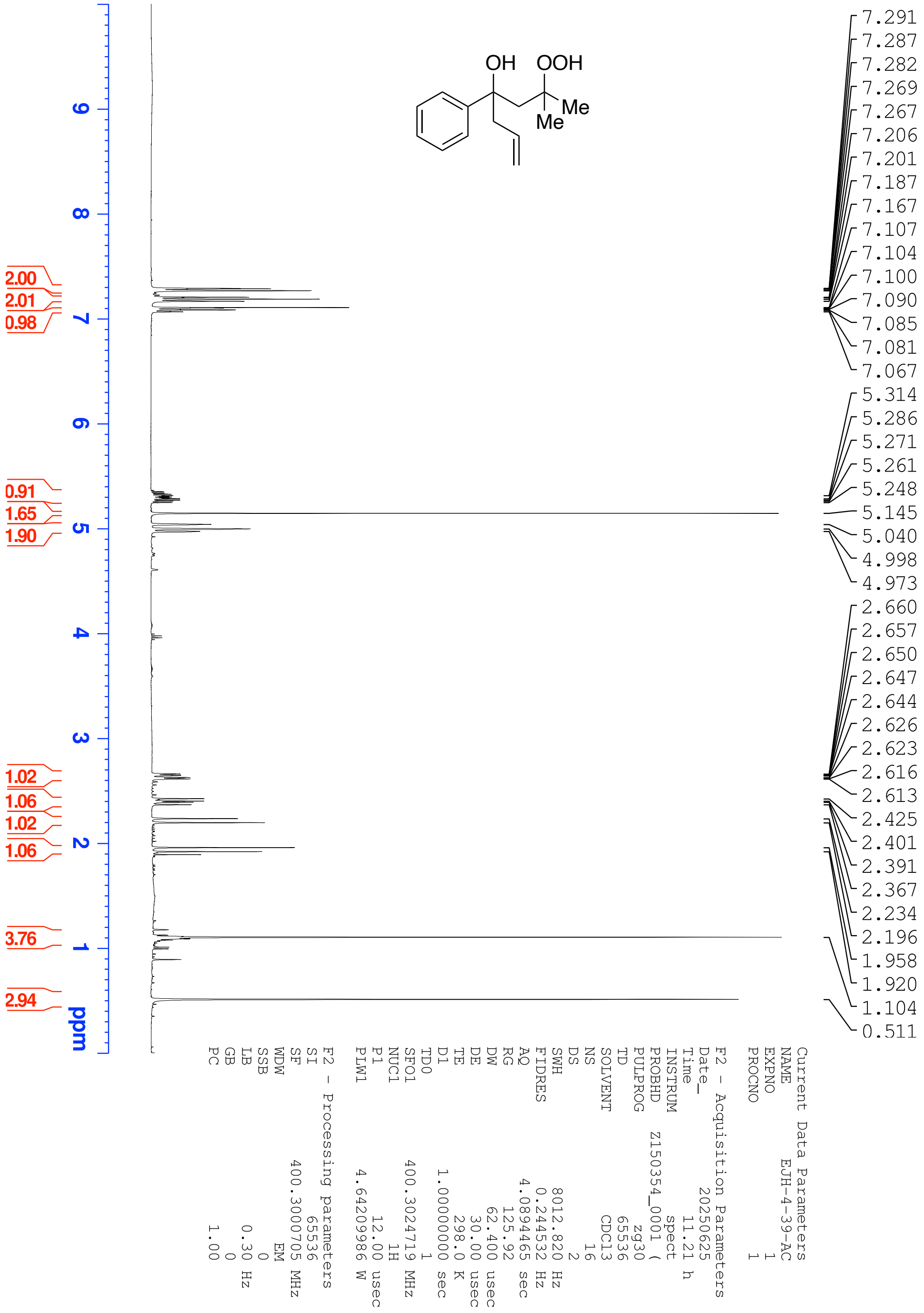

6-Hydroperoxy-6-methyl-4-phenylhept-1-en-4-ol (S18).

Solvent: $CDCl_3$
100 MHz
$^{13}C\{^1H\}$ NMR

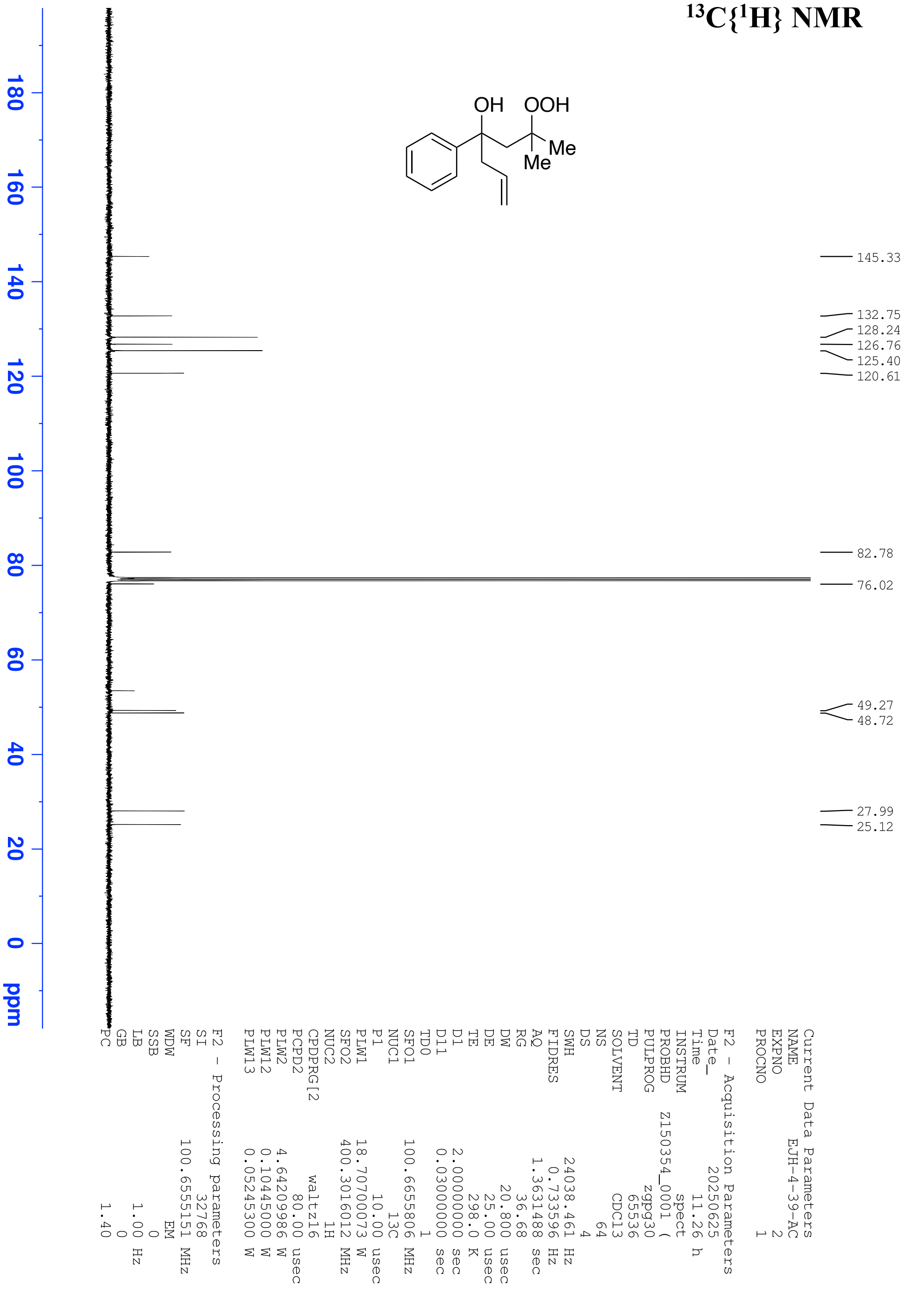

2-Hydroperoxy-2,4-dimethylpentadecan-4-ol (S19).

Solvent: $CDCl_3$
400 MHz
^{1}H NMR

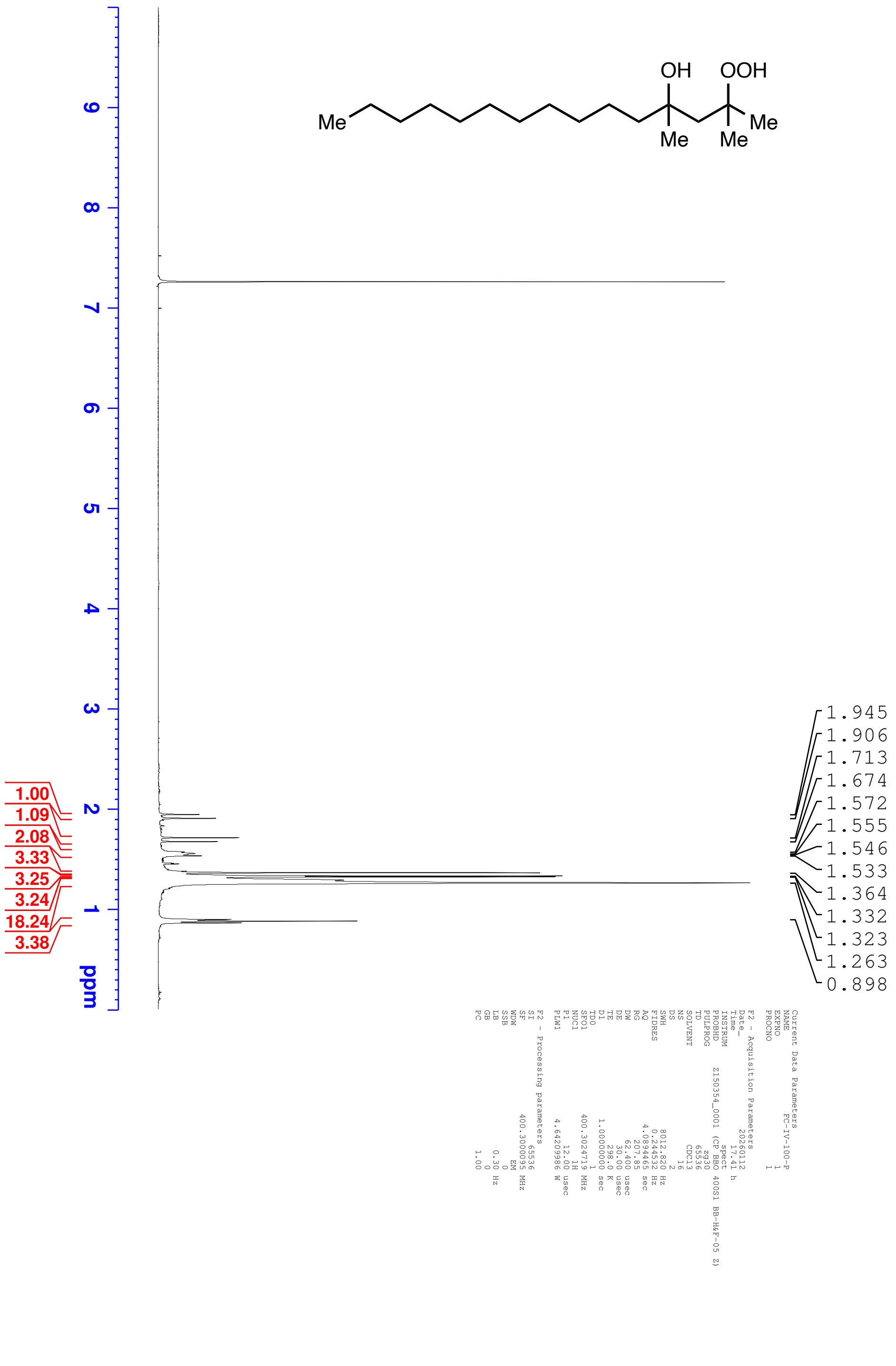

Solvent: $CDCl_3$
100 MHz
$^{13}C\{^1H\}$ NMR

2-Hydroperoxy-2,4-dimethylpentadecan-4-ol (S19).

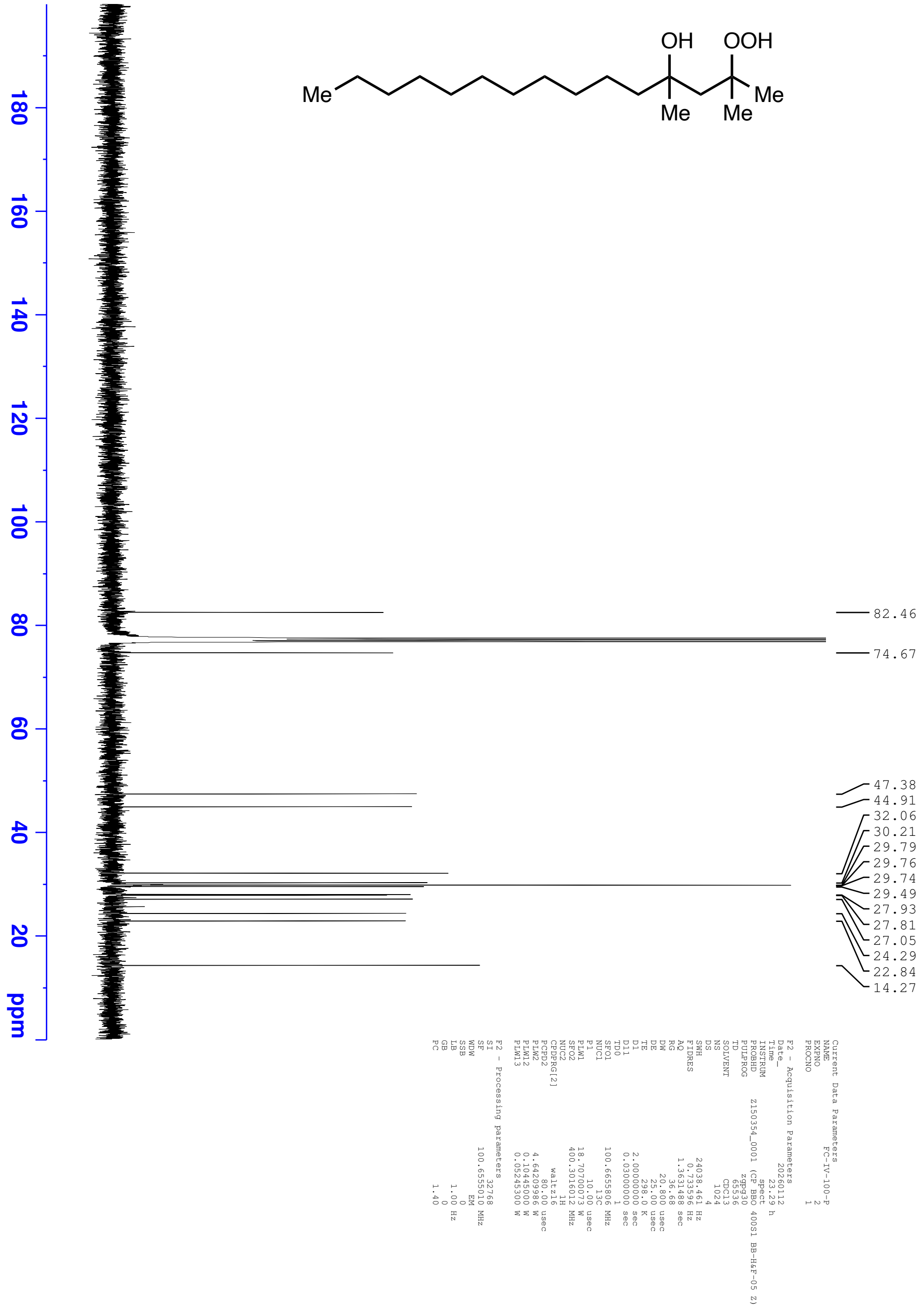

3-Hydroperoxy-3-methyl-1-phenylbutan-1-ol (S20).

Solvent: $CDCl_3$
400 MHz
^{1}H NMR

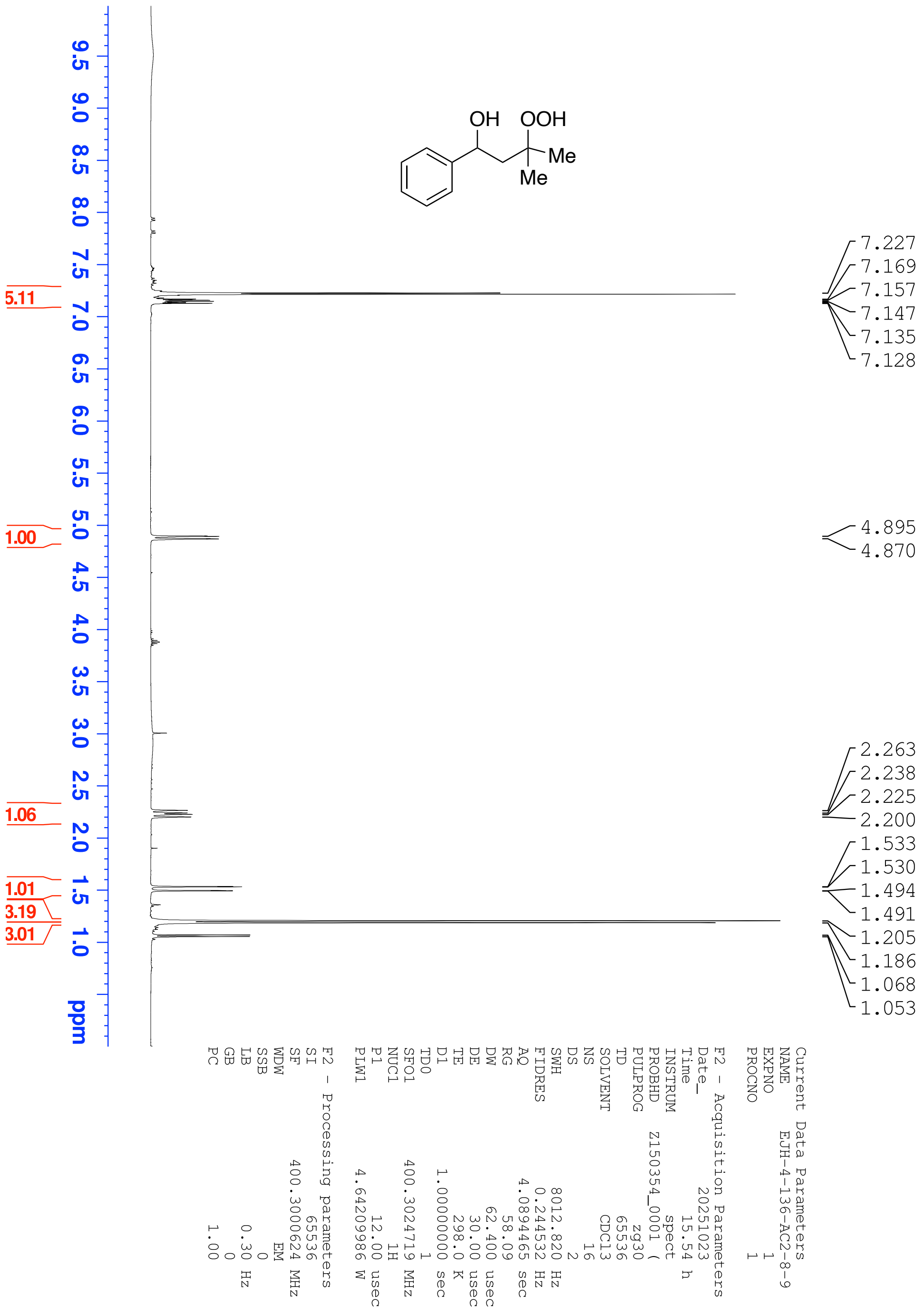

3-Hydroperoxy-3-methyl-1-phenylbutan-1-ol (S20).

Solvent: $CDCl_3$
100 MHz
$^{13}C\{^1H\}$ NMR

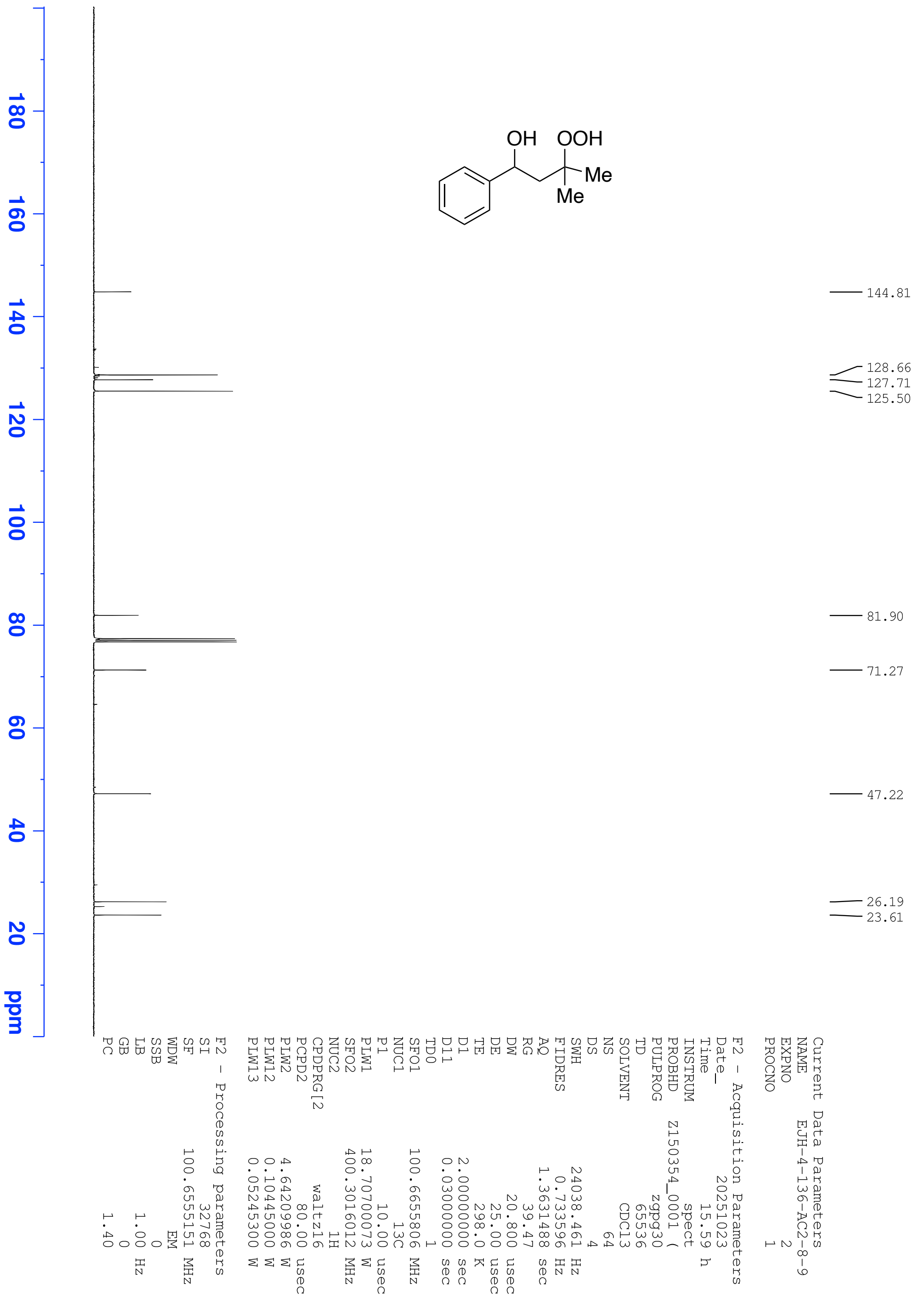

1-(4-Bromophenyl)-3-hydroperoxy-3-methylbutan-1-ol (S21).

Solvent: $CDCl_3$
400 MHz
^{1}H NMR

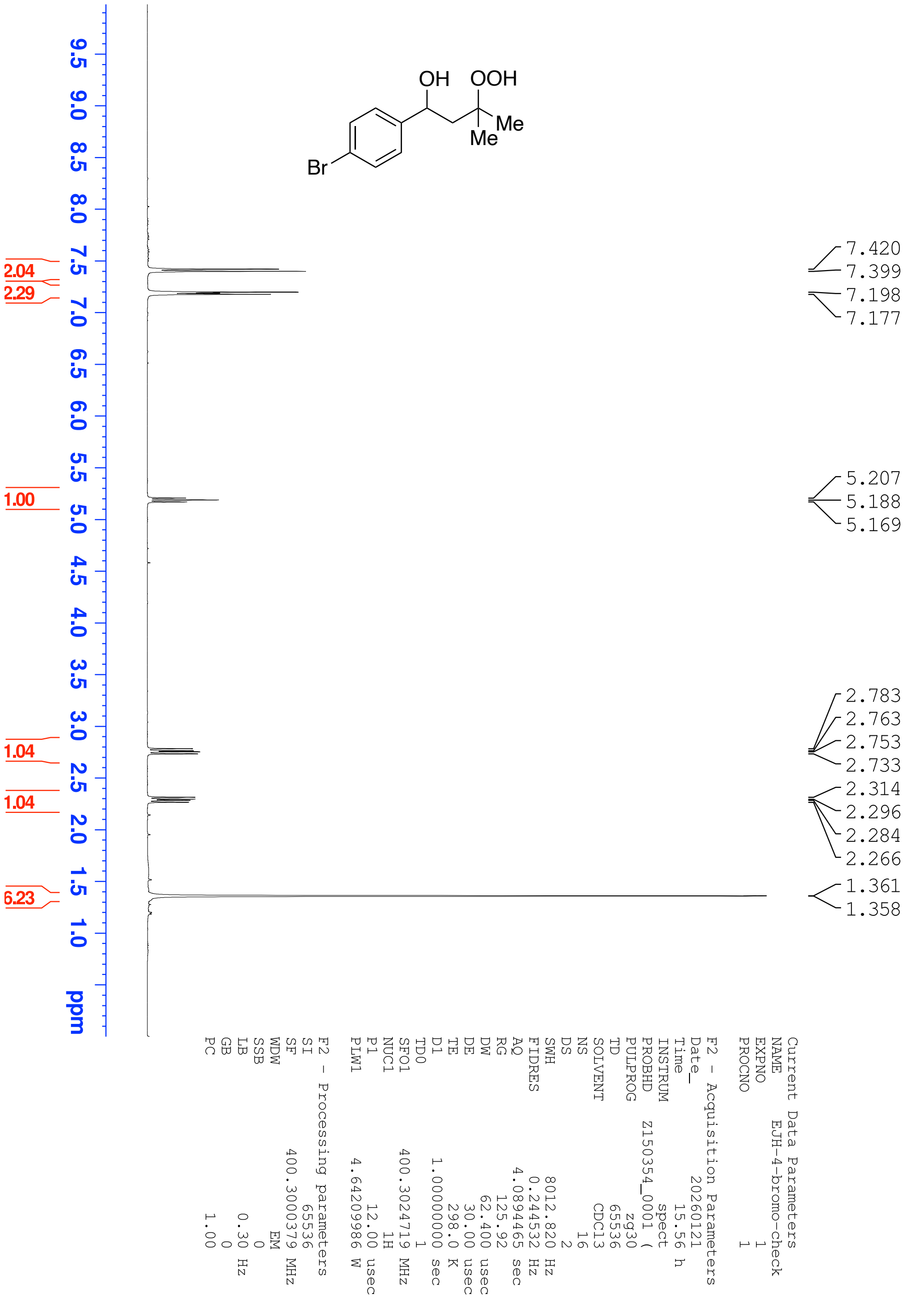

1-(4-Bromophenyl)-3-hydroperoxy-3-methylbutan-1-ol (S21).

Solvent: $CDCl_3$
100 MHz
$^{13}C\{^1H\}$ NMR

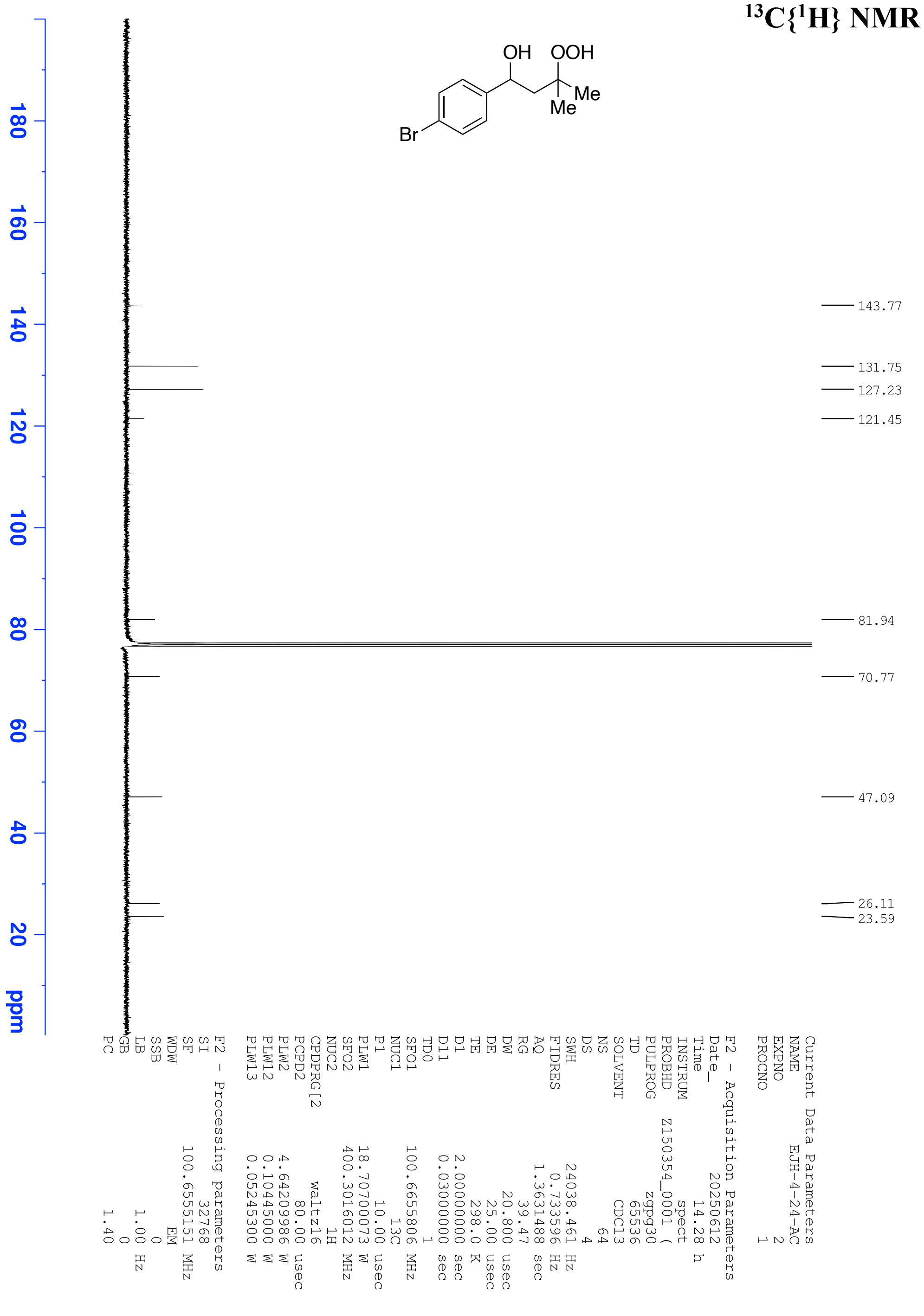

1-(4-(3-Hydroperoxy-1-hydroxy-3-methylbutyl)phenyl)ethan-1-one (S22).

Solvent: $CDCl_3$
400 MHz
^{1}H NMR

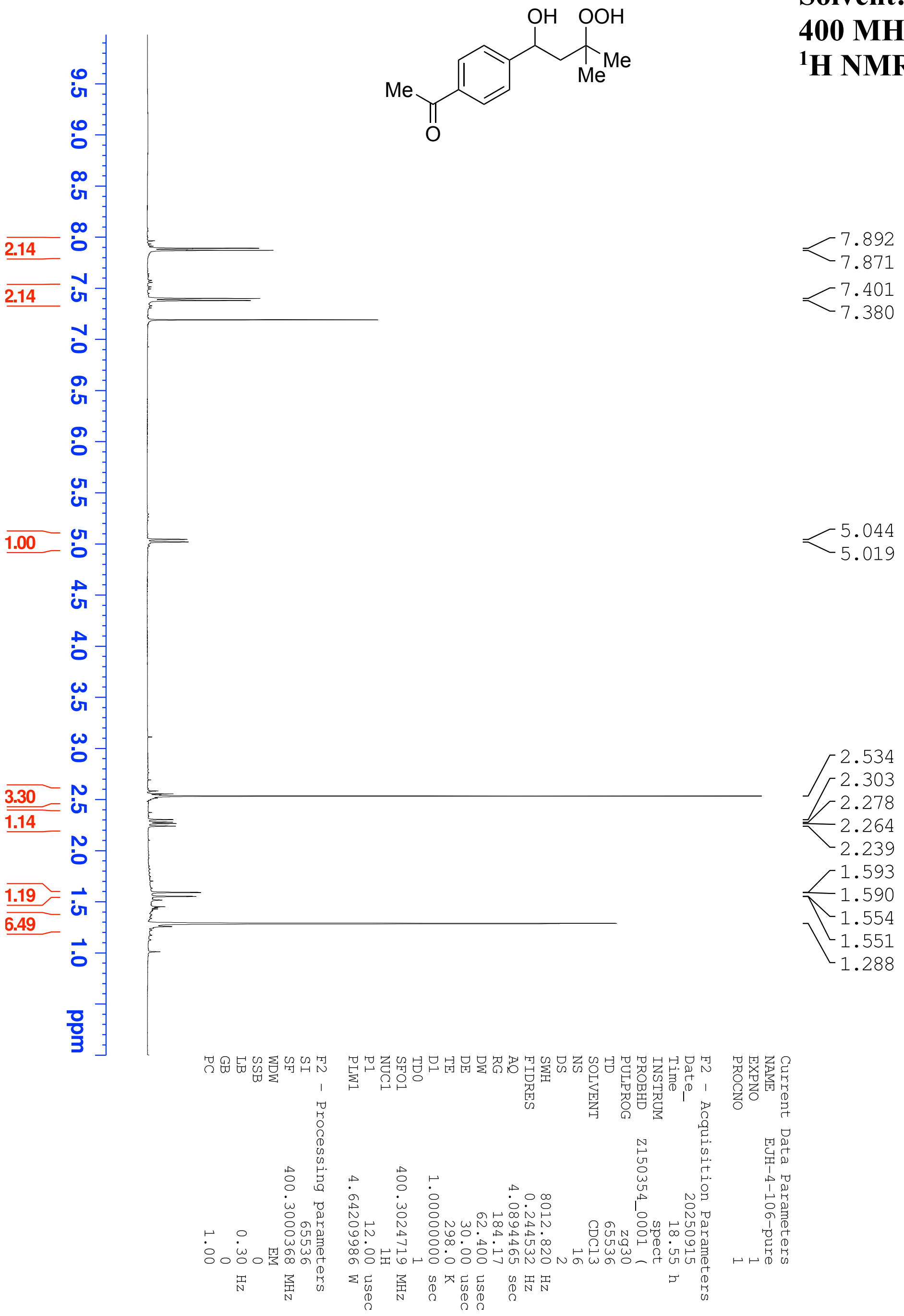

1-(4-(3-Hydroperoxy-1-hydroxy-3-methylbutyl)phenyl)ethan-1-one (S22).

Solvent: $CDCl_3$
100 MHz
$^{13}C\{^1H\}$ NMR

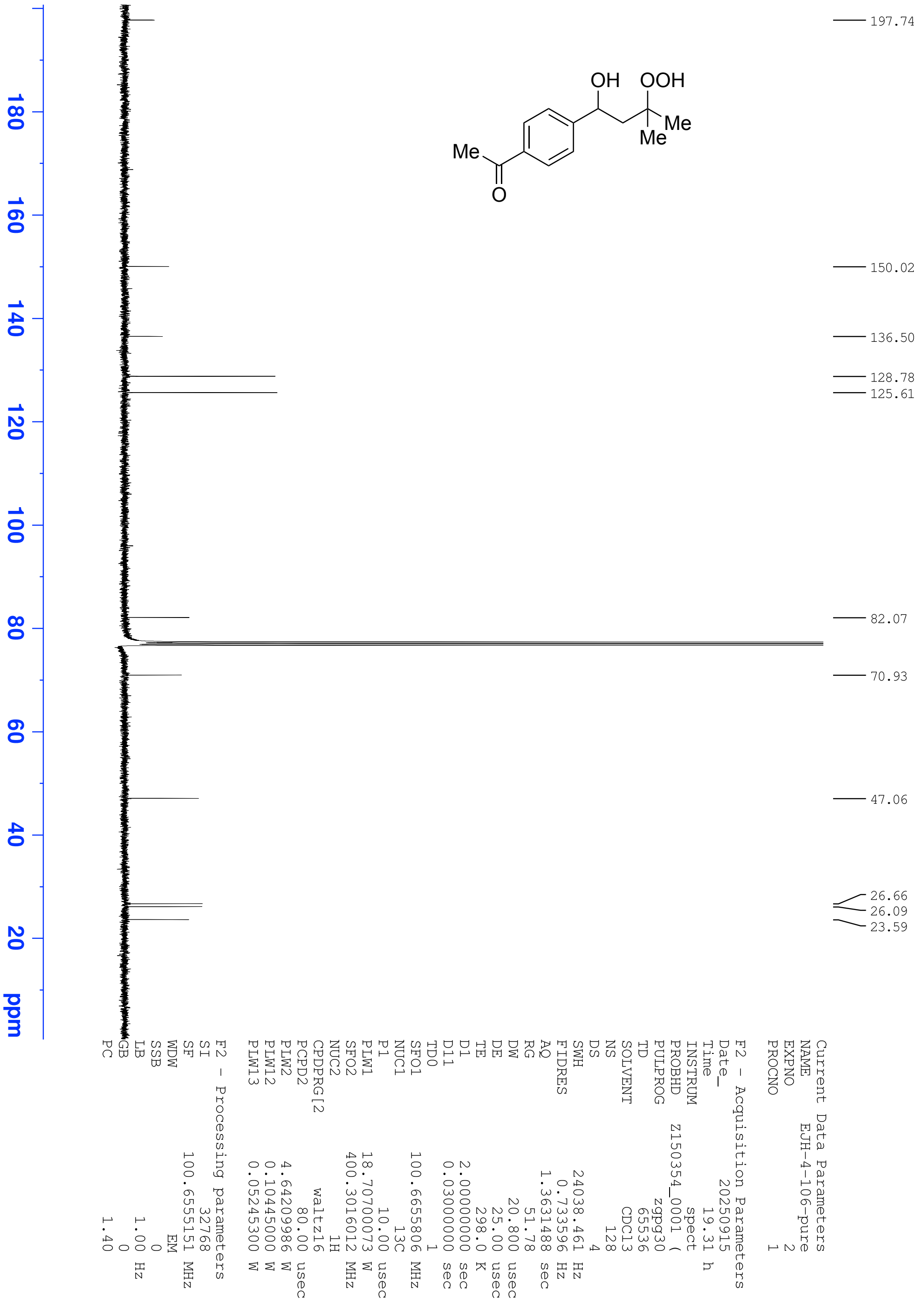

3-Hydroperoxy-1,1-diphenylbutan-1-ol (S23).

Solvent: $CDCl_3$
400 MHz
^{1}H NMR

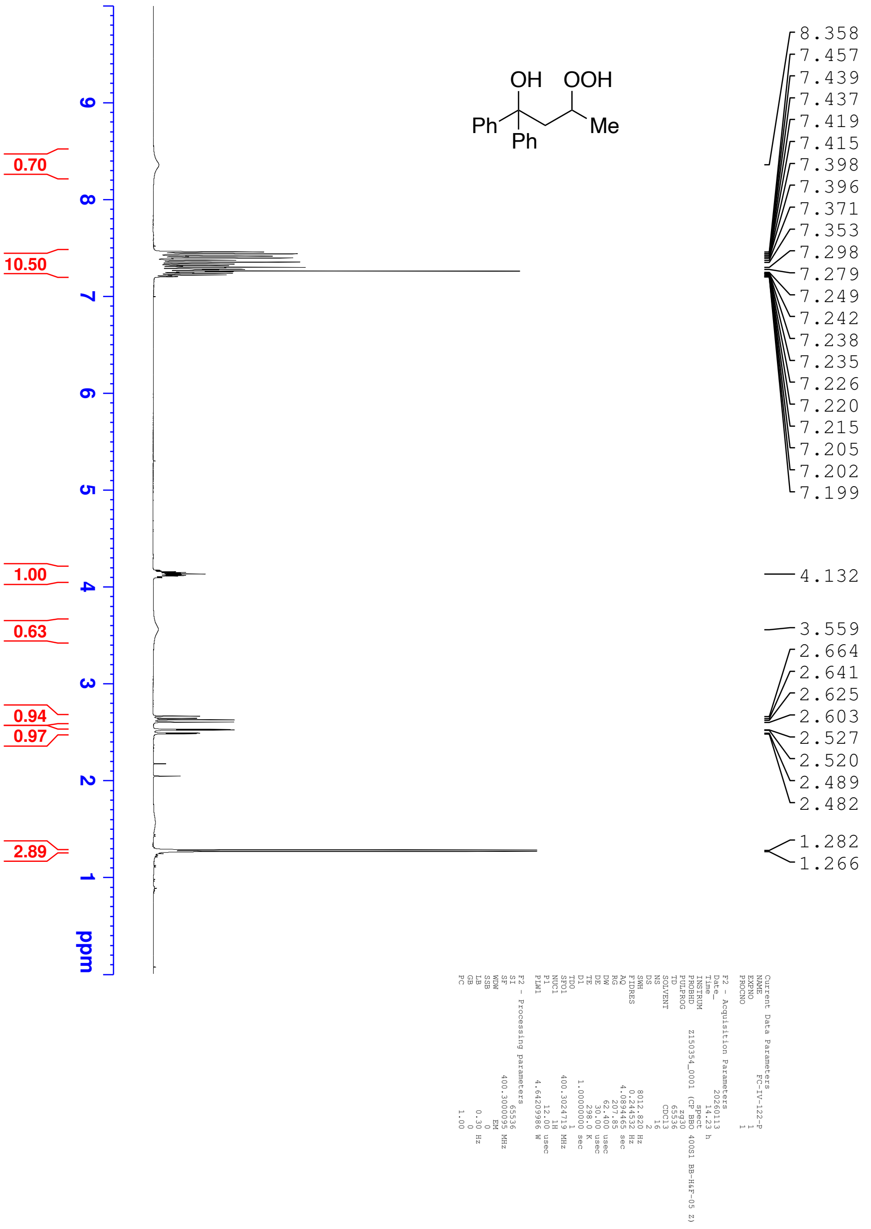

3-Hydroperoxy-1,1-diphenylbutan-1-ol (S23).

Solvent: $CDCl_3$
100 MHz
$^{13}C\{^1H\}$ NMR

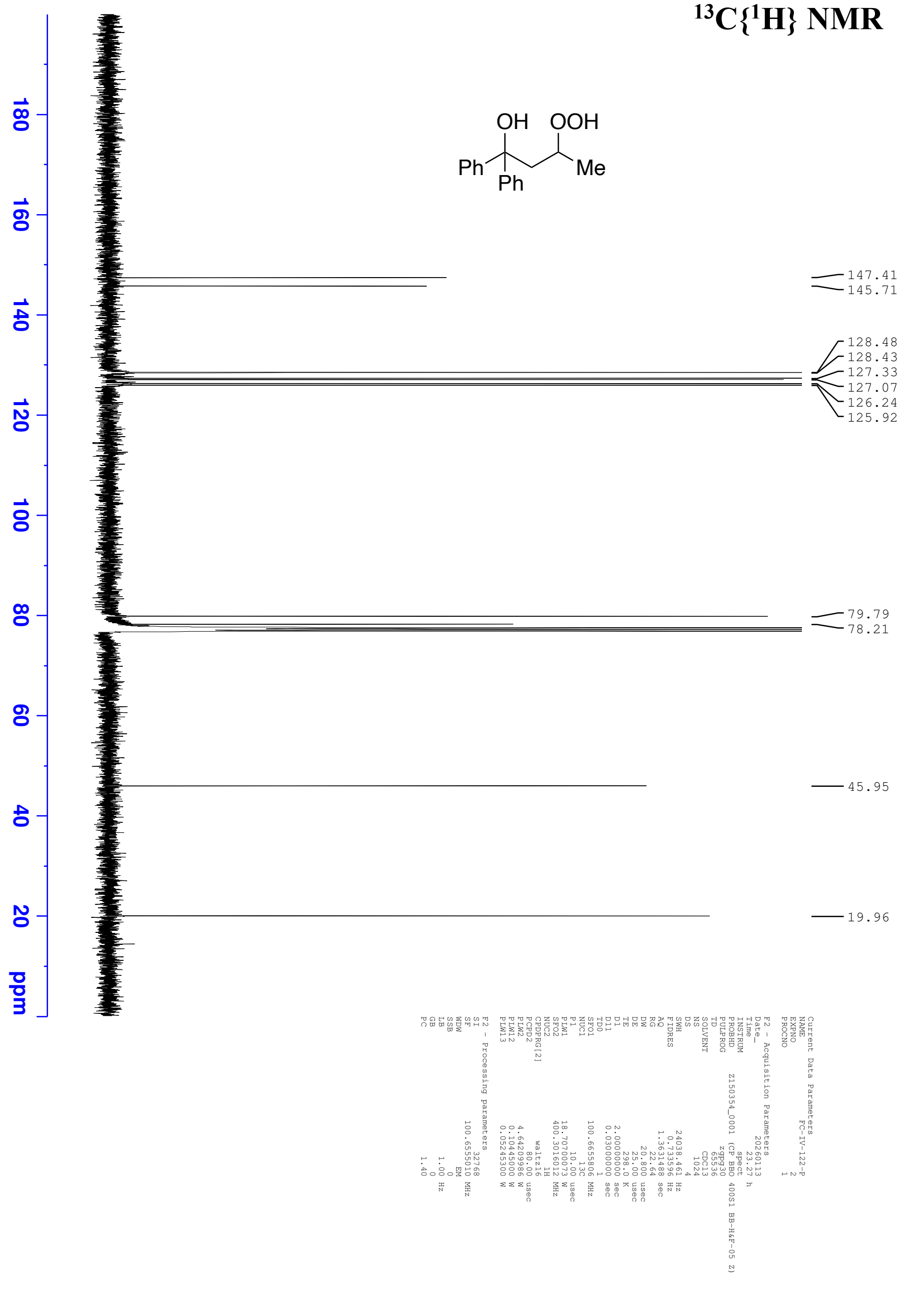

(1S,4S)-1-(2-Hydroperoxy-2-methylpropyl)-4-phenylcyclohexan-1-ol (S24).

Solvent: $CDCl_3$
400 MHz
^{1}H NMR

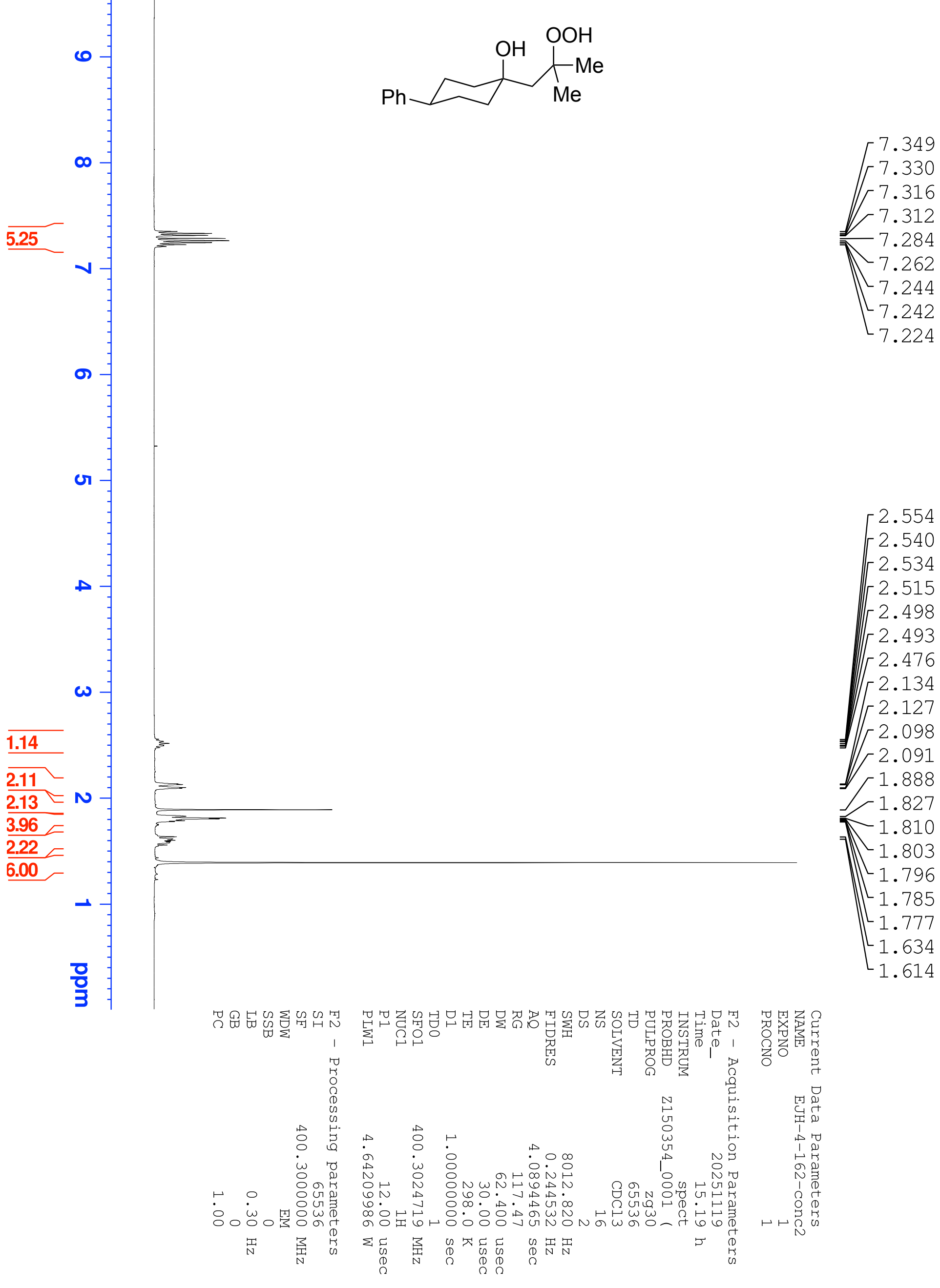

(1S,4S)-1-(2-Hydroperoxy-2-methylpropyl)-4-phenylcyclohexan-1-ol (S24).

Solvent: $CDCl_3$
100 MHz
$^{13}C\{^1H\}$ NMR

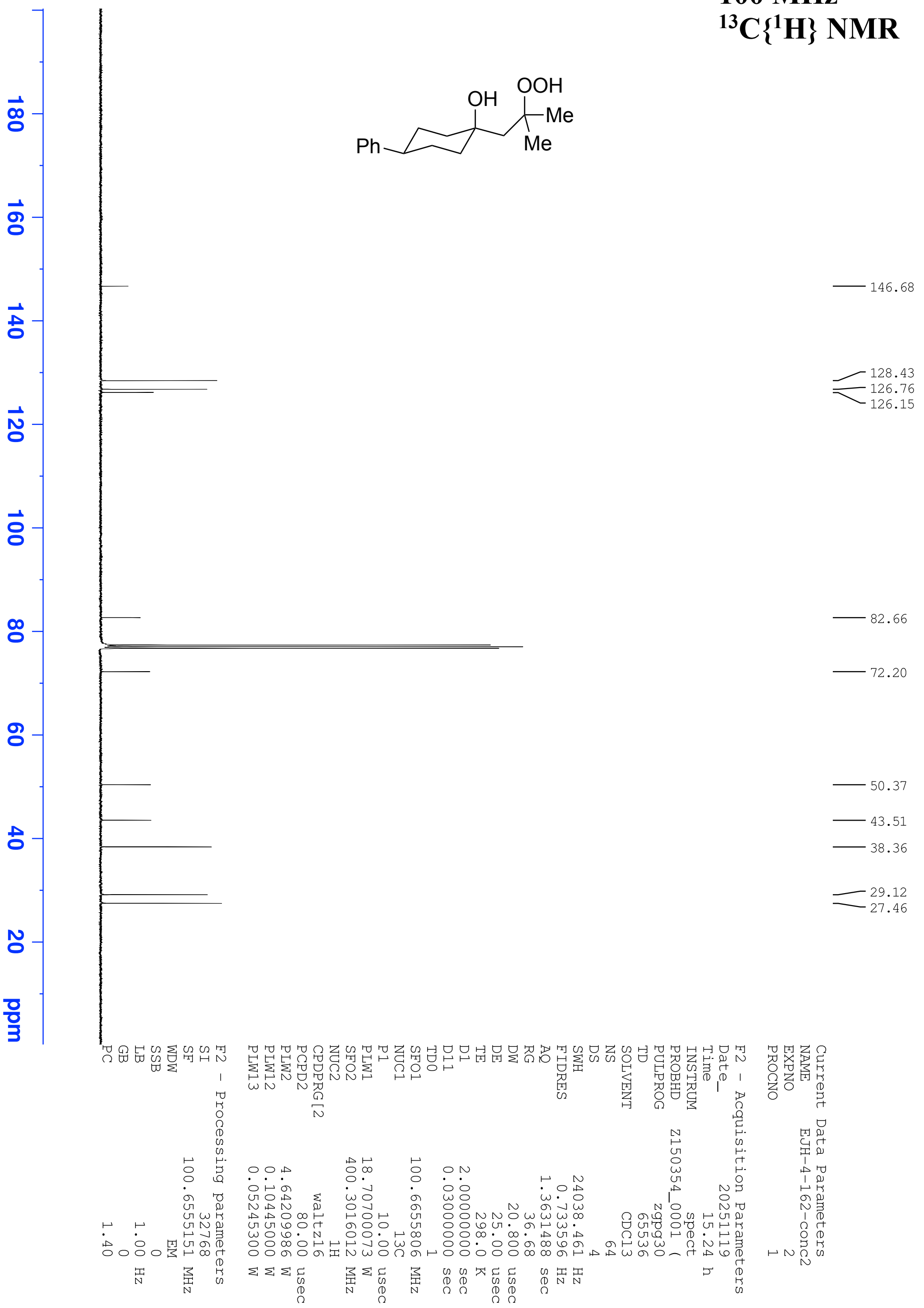

3-Hydroperoxy-3-methyl-2-phenylbutan-1-ol (S25).*

Solvent: $CDCl_3$
400 MHz
^{1}H NMR

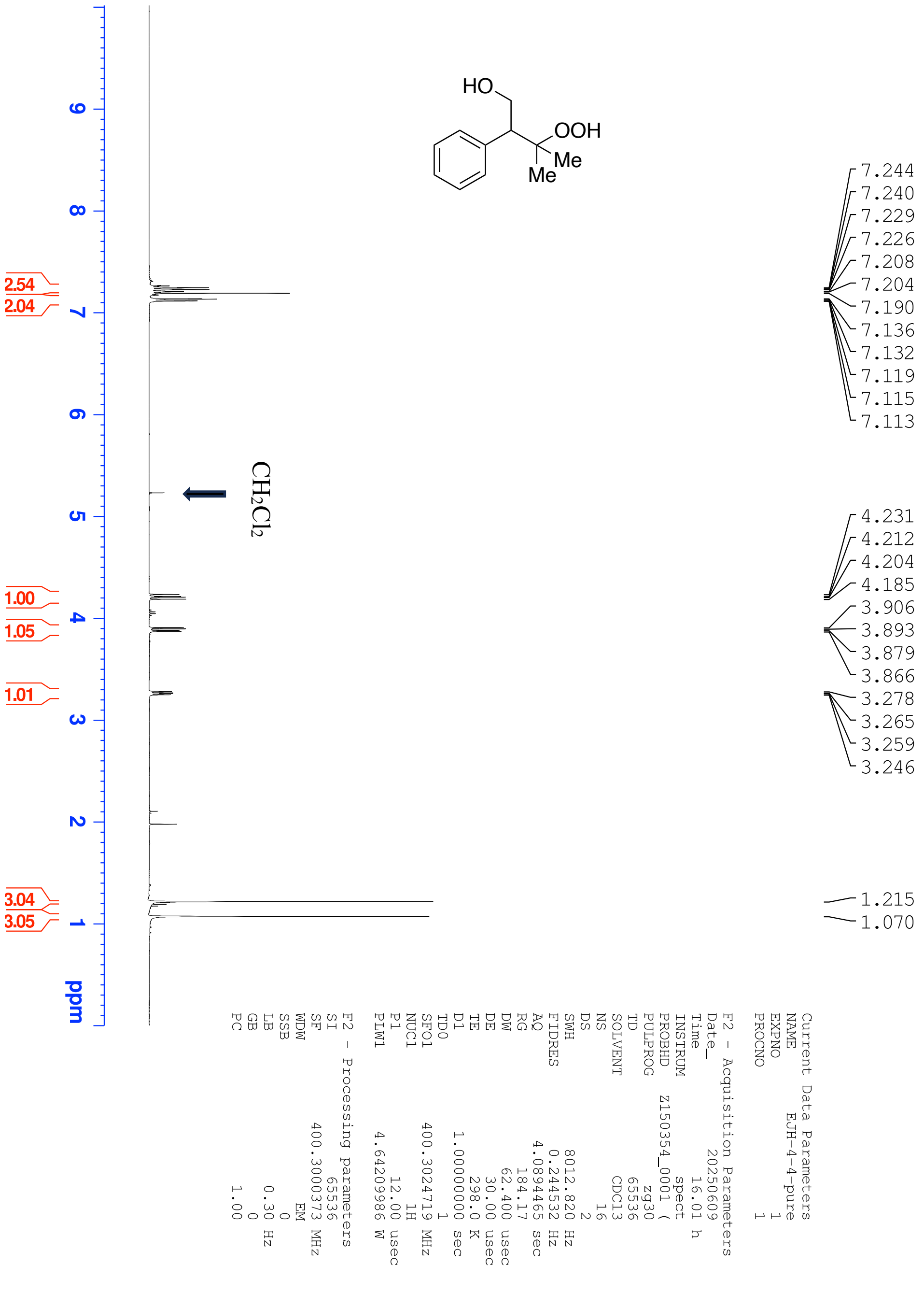

***S25** was brought forward to the next reaction as a crude mixture.

3-Hydroperoxy-3-methyl-2-phenylbutan-1-ol (S25). *

Solvent: $CDCl_3$
100 MHz
$^{13}C\{^1H\}$ NMR

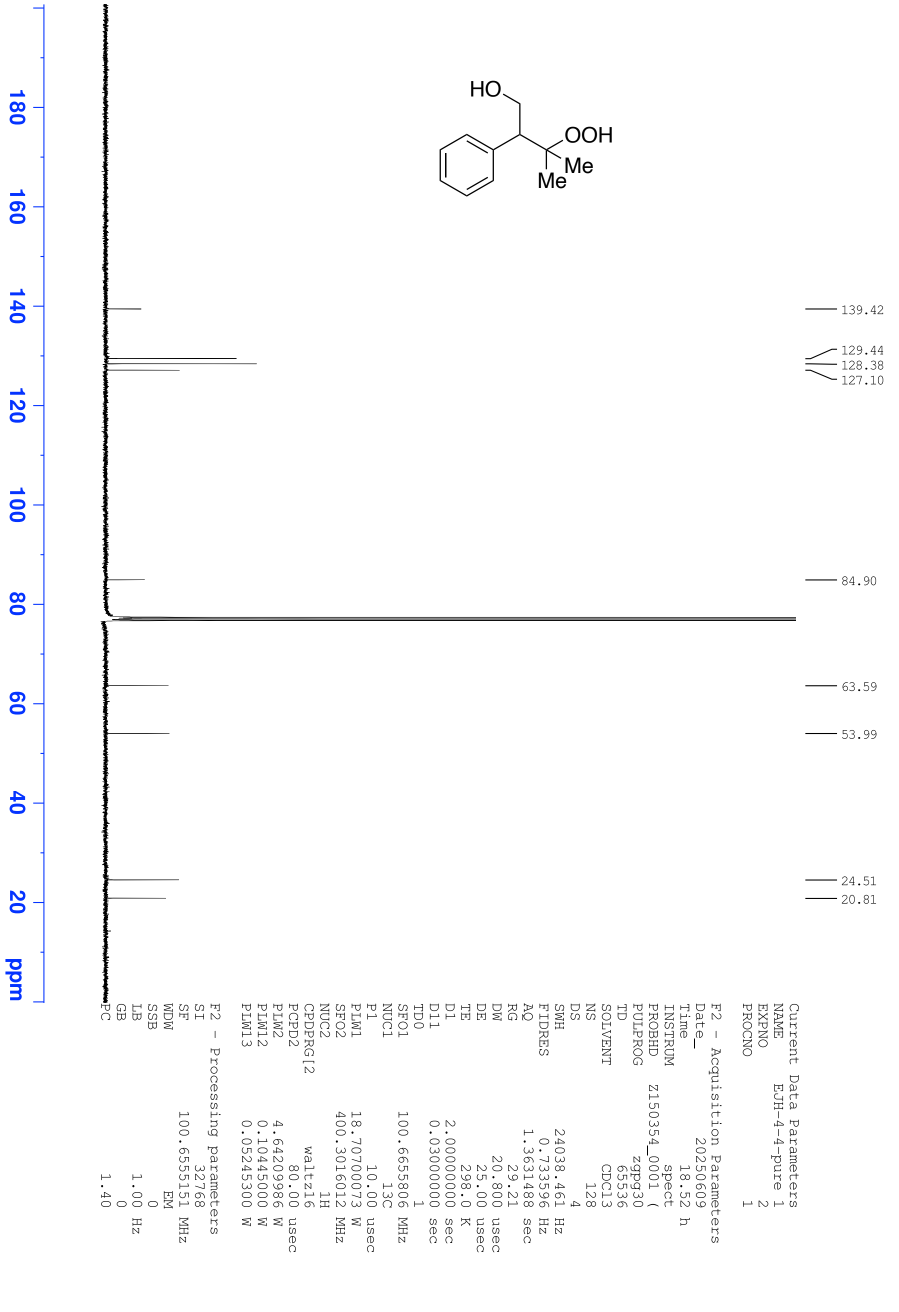

* **S25** was brought forward to the next reaction as a crude mixture.

(1R,2R)-2-(2-Hydroperoxypropan-2-yl)cyclohexan-1-ol (S26).

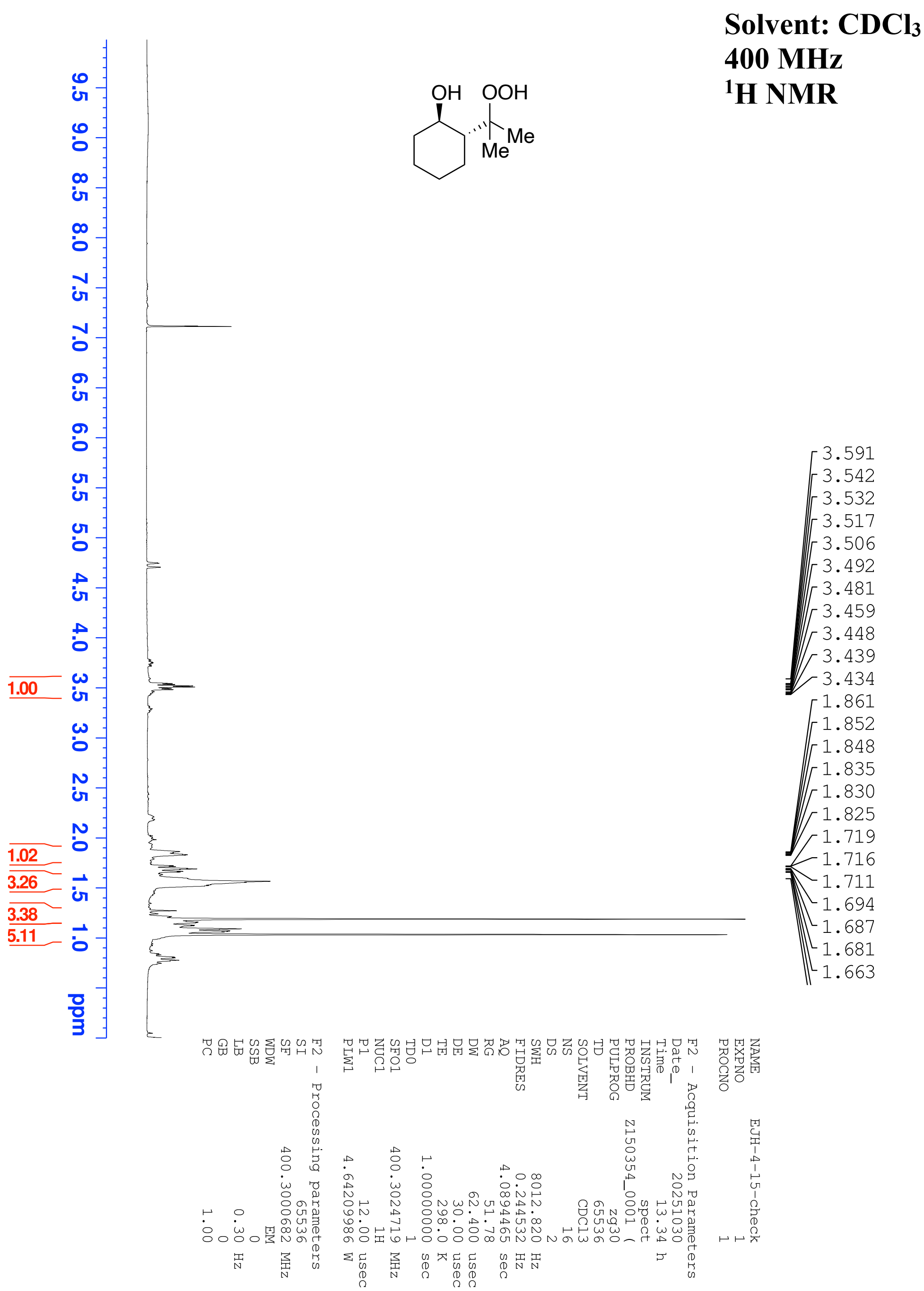

(1R,2R)-2-(2-Hydroperoxypropan-2-yl)cyclohexan-1-ol (S26).

Solvent: $CDCl_3$
100 MHz
$^{13}C\{^1H\}$ NMR

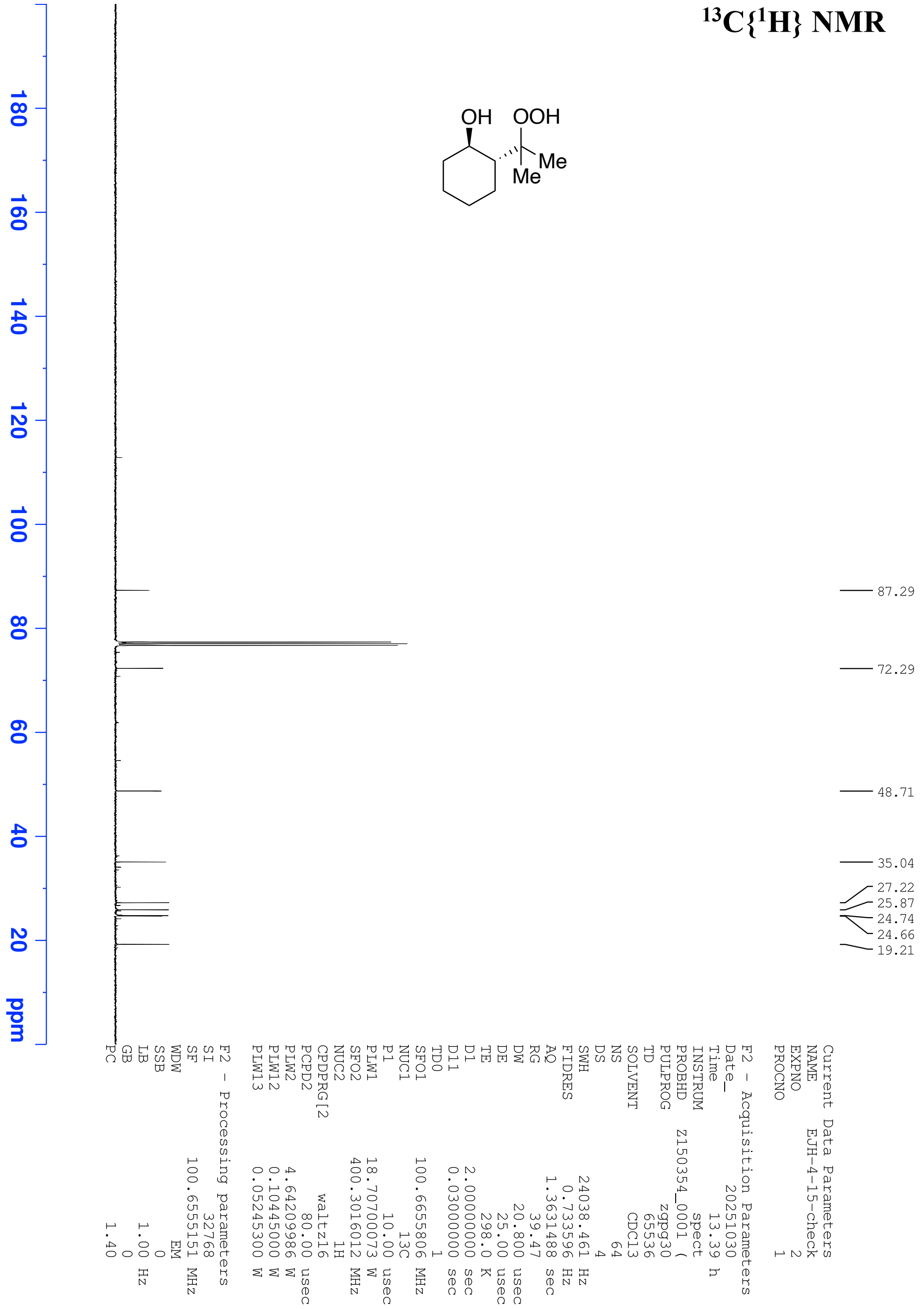

4-Hydroperoxy-4-methyl-1,1-diphenylpentan-1-ol (S27).*

Solvent: $CDCl_3$
400 MHz
^{1}H NMR

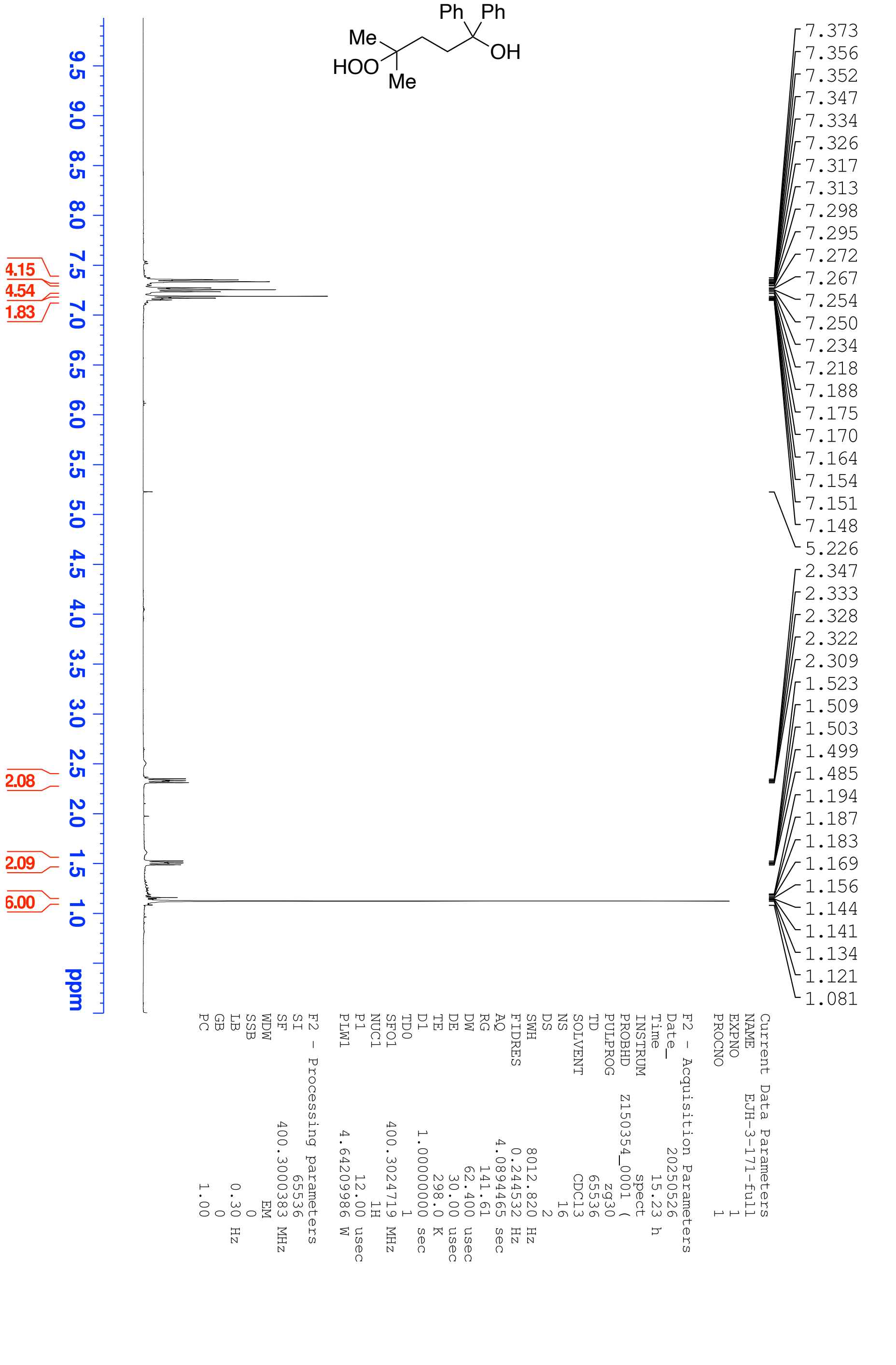

*[*]**S27** was brought forward to the next reaction as a crude mixture.

4-Hydroperoxy-4-methyl-1,1-diphenylpentan-1-ol (S27).*

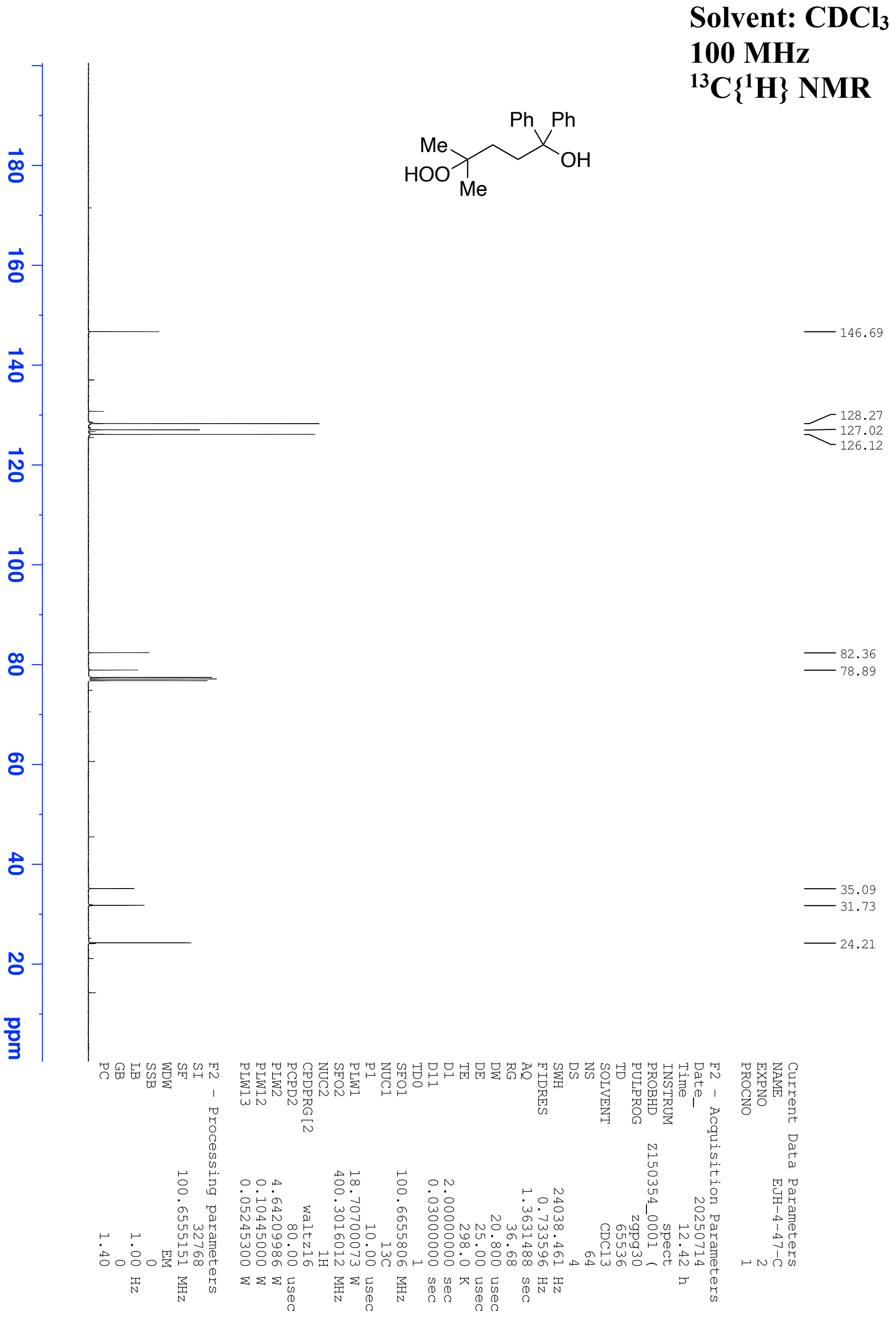

*__S27__ was brought forward to the next reaction as a crude mixture.

(E)-4-(3,5-Bis(trifluoromethyl)phenyl)pent-3-en-2-one (S28).

Solvent: $CDCl_3$
400 MHz
^{1}H NMR

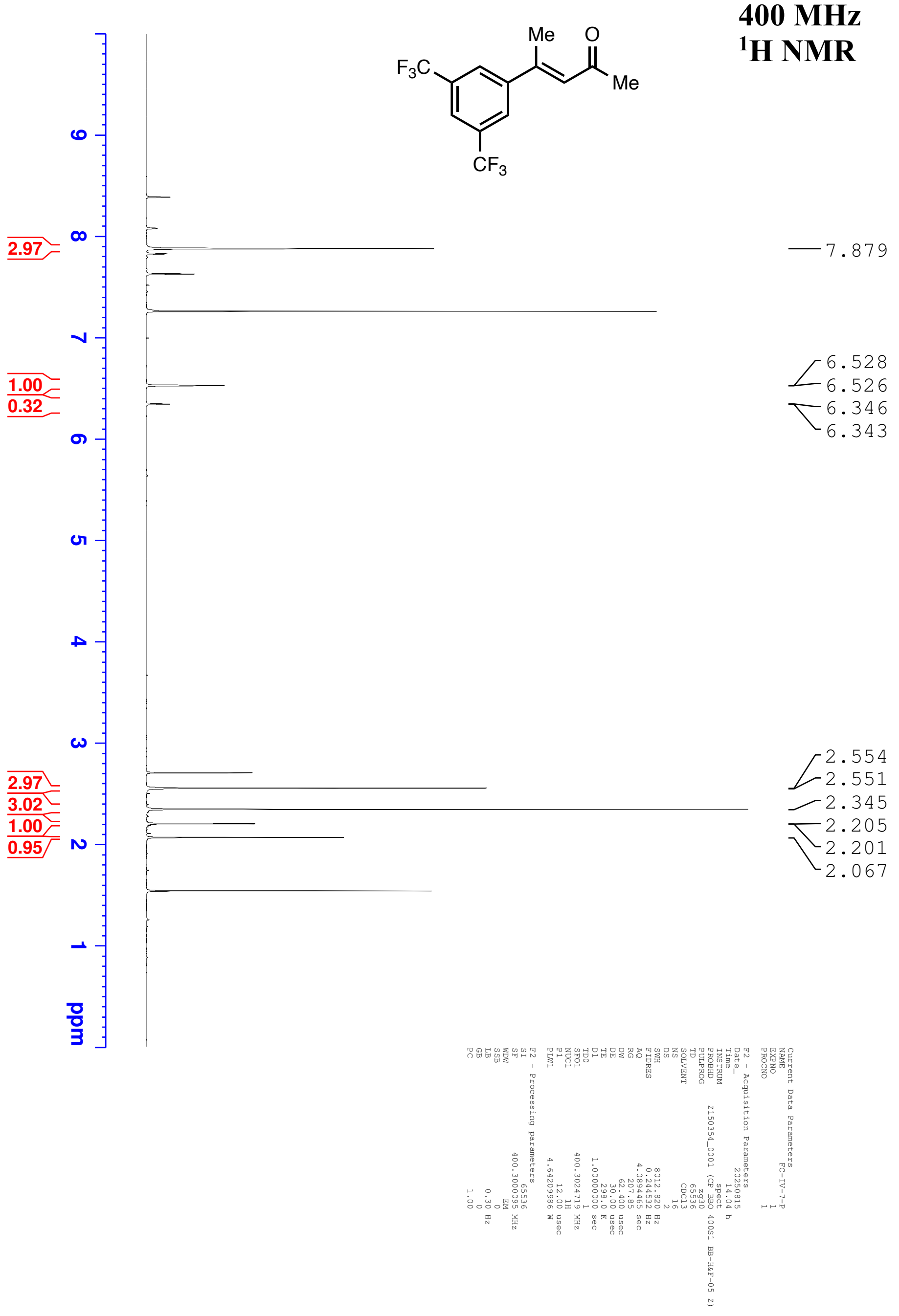

Solvent: $CDCl_3$
100 MHz
$^{13}C\{^1H\}$ NMR

(E)-4-(3,5-Bis(trifluoromethyl)phenyl)pent-3-en-2-one (S28).

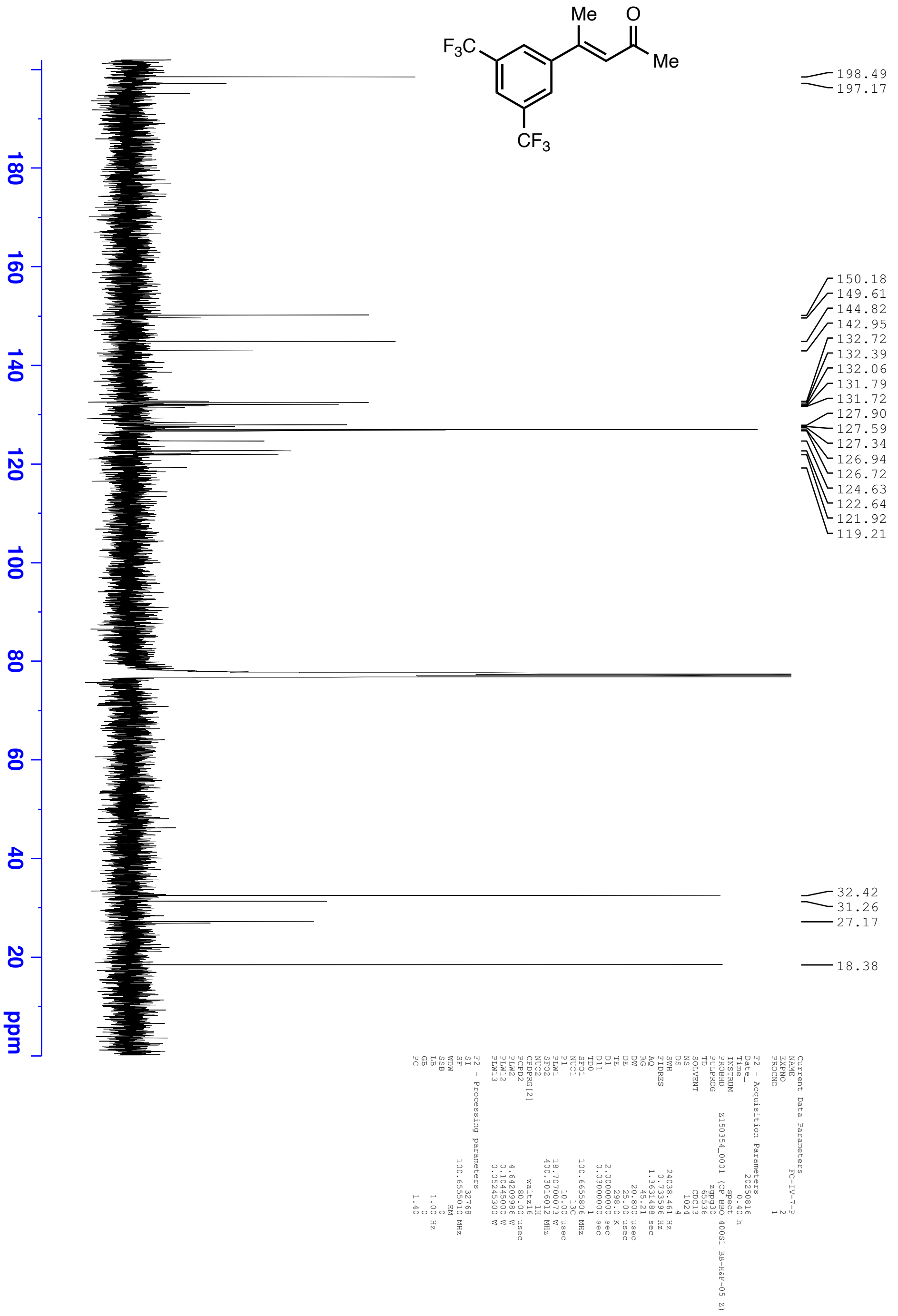

(E)-4-(3,5-Bis(trifluoromethyl)phenyl)pent-3-en-2-one (S28).

Solvent: $CDCl_3$
377 MHz
$^{19}F\{^1H\}$ NMR

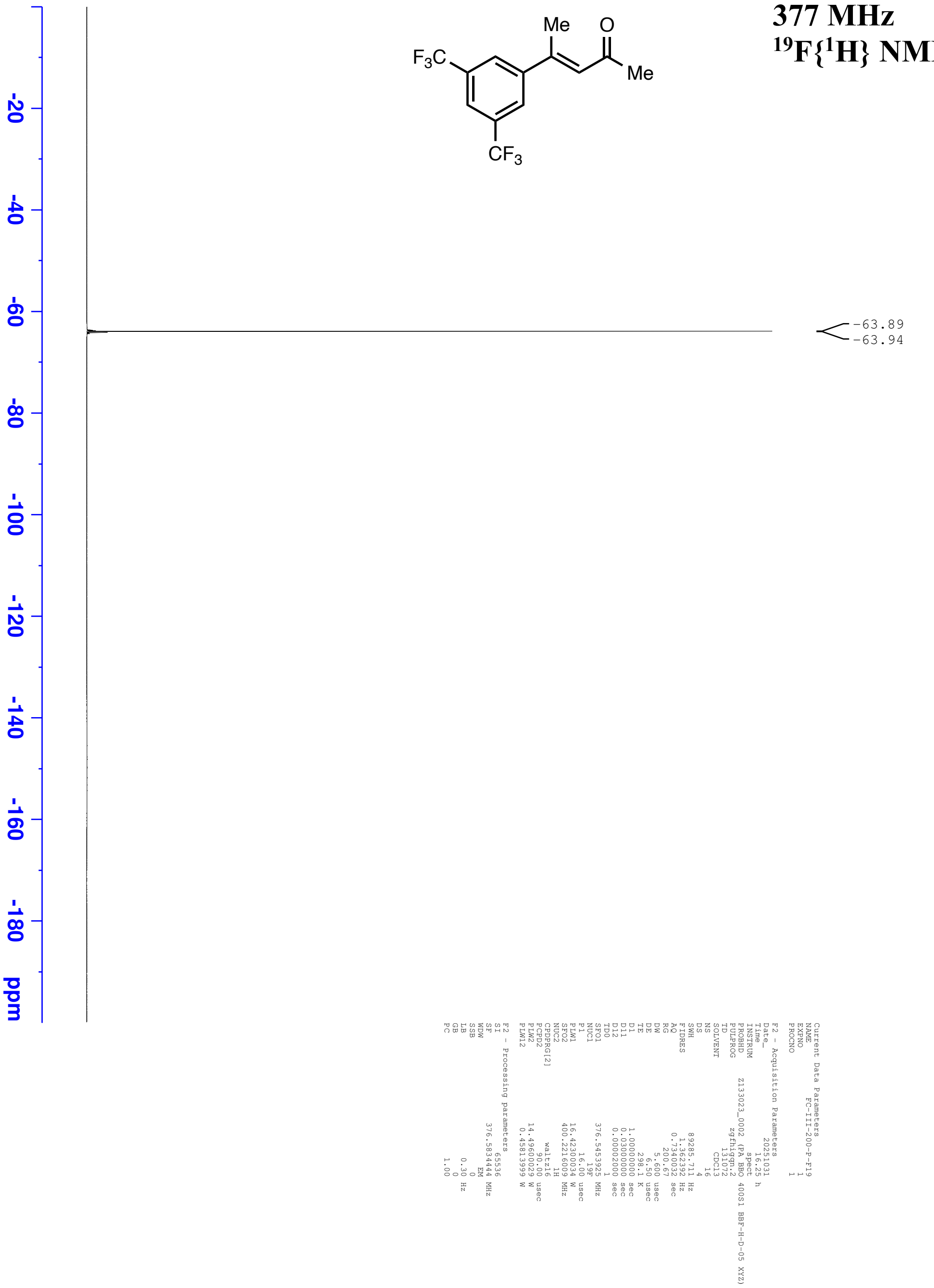

Solvent: $CDCl_3$
400 MHz
^{1}H NMR

(E)-4-(3,5-Bis(trifluoromethyl)phenyl)-2-phenylpent-3-en-2-ol (S29).

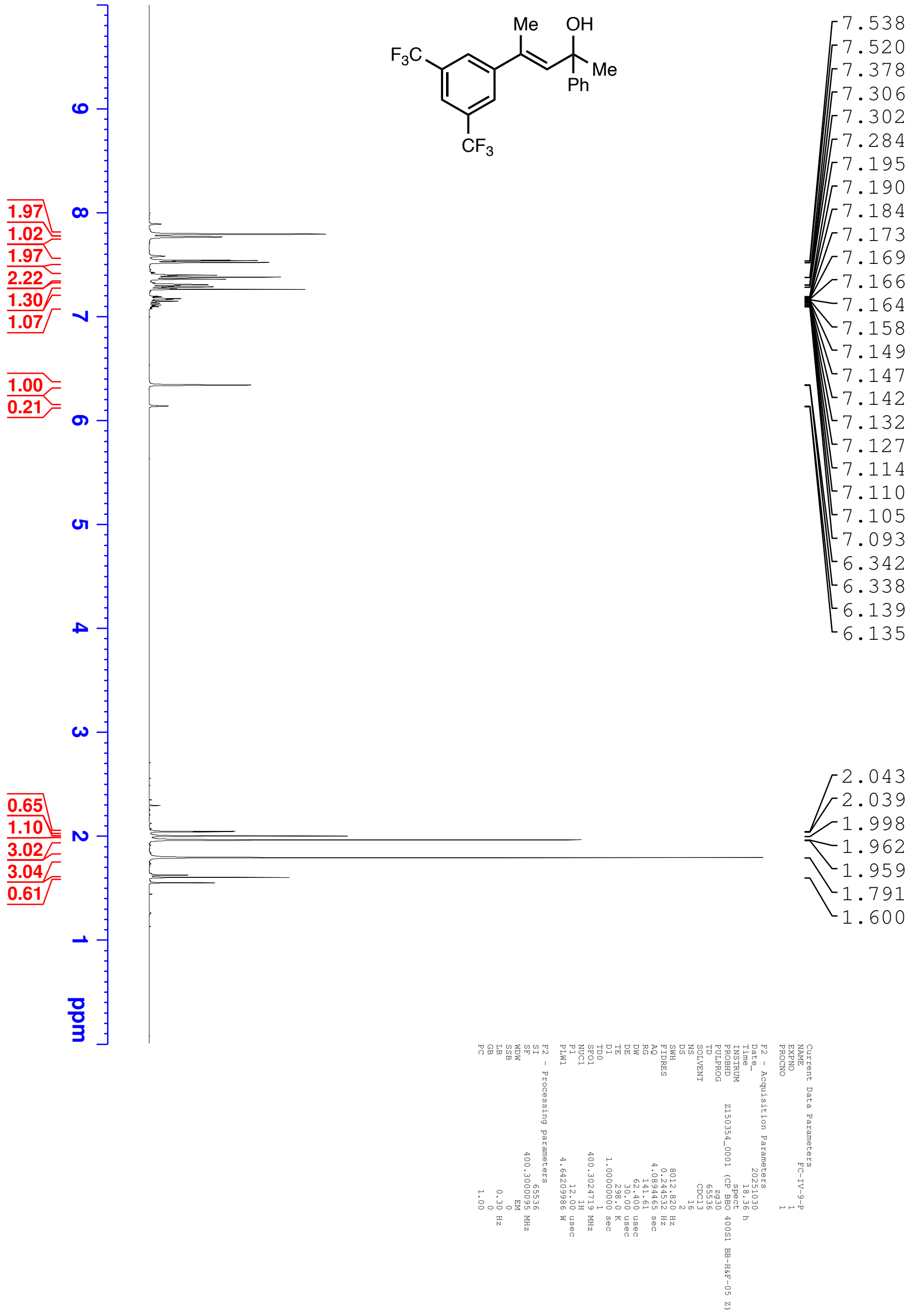

***(E)*-4-(3,5-Bis(trifluoromethyl)phenyl)-2-phenylpent-3-en-2-ol (S29).**

Solvent: $CDCl_3$
100 MHz
$^{13}C\{^1H\}$ NMR

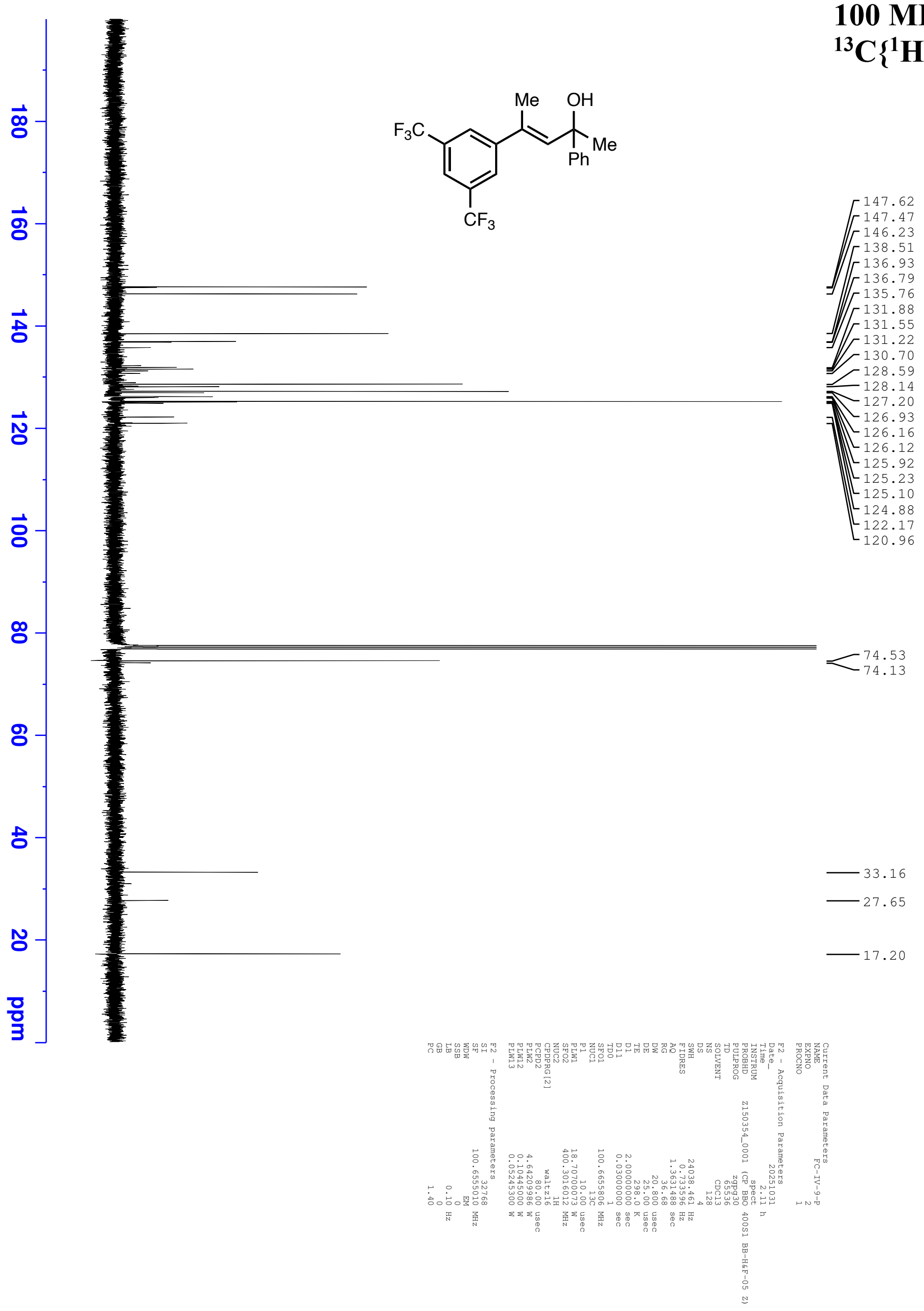

(E)-4-(3,5-Bis(trifluoromethyl)phenyl)-2-phenylpent-3-en-2-ol (S29).

Solvent: $CDCl_3$
377 MHz
$^{19}F\{^1H\}$ NMR

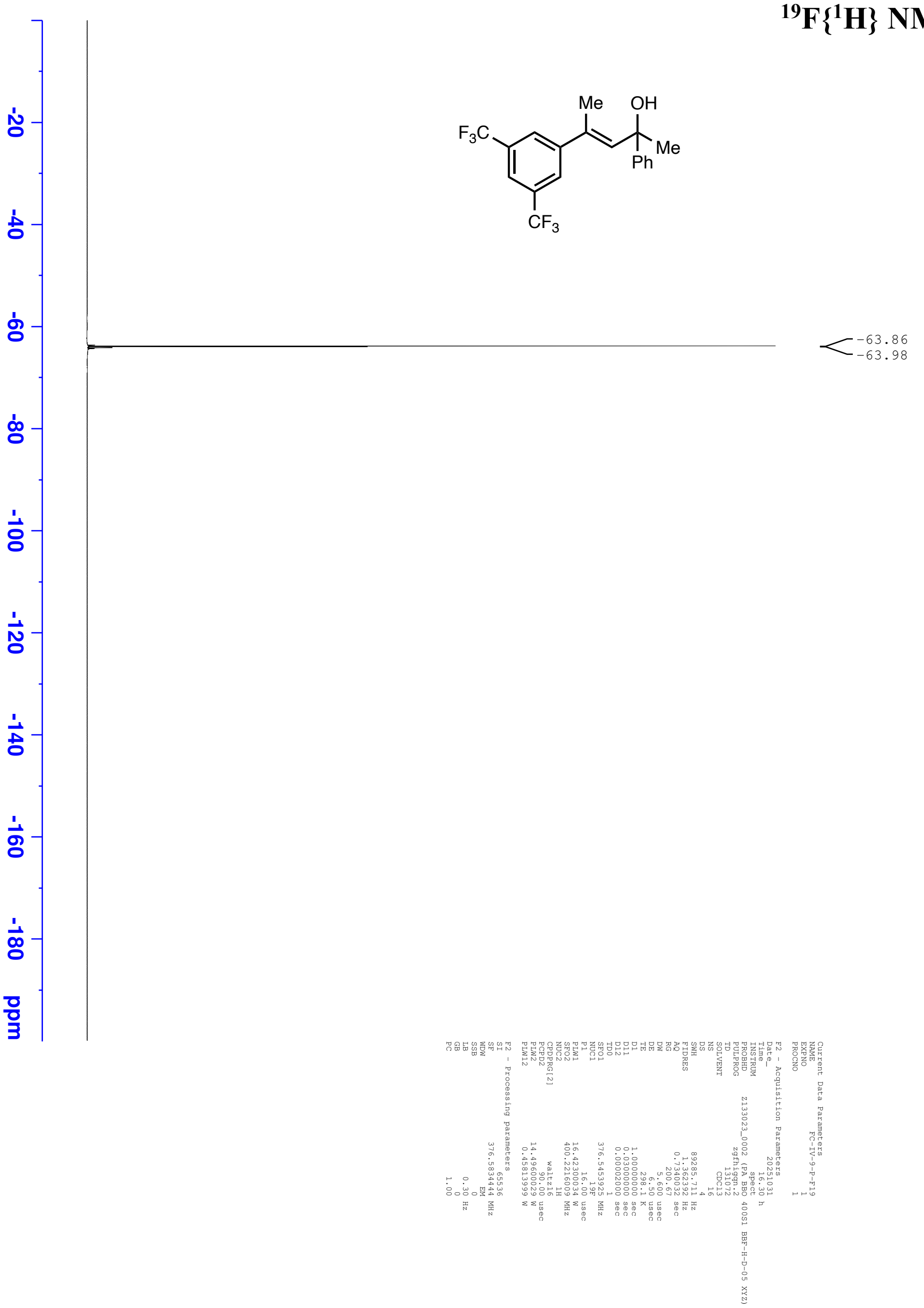

2-(4-Bromophenyl)-4-methylpent-4-en-2-yl benzoate (S30).

Solvent: $CDCl_3$
400 MHz
^{1}H NMR

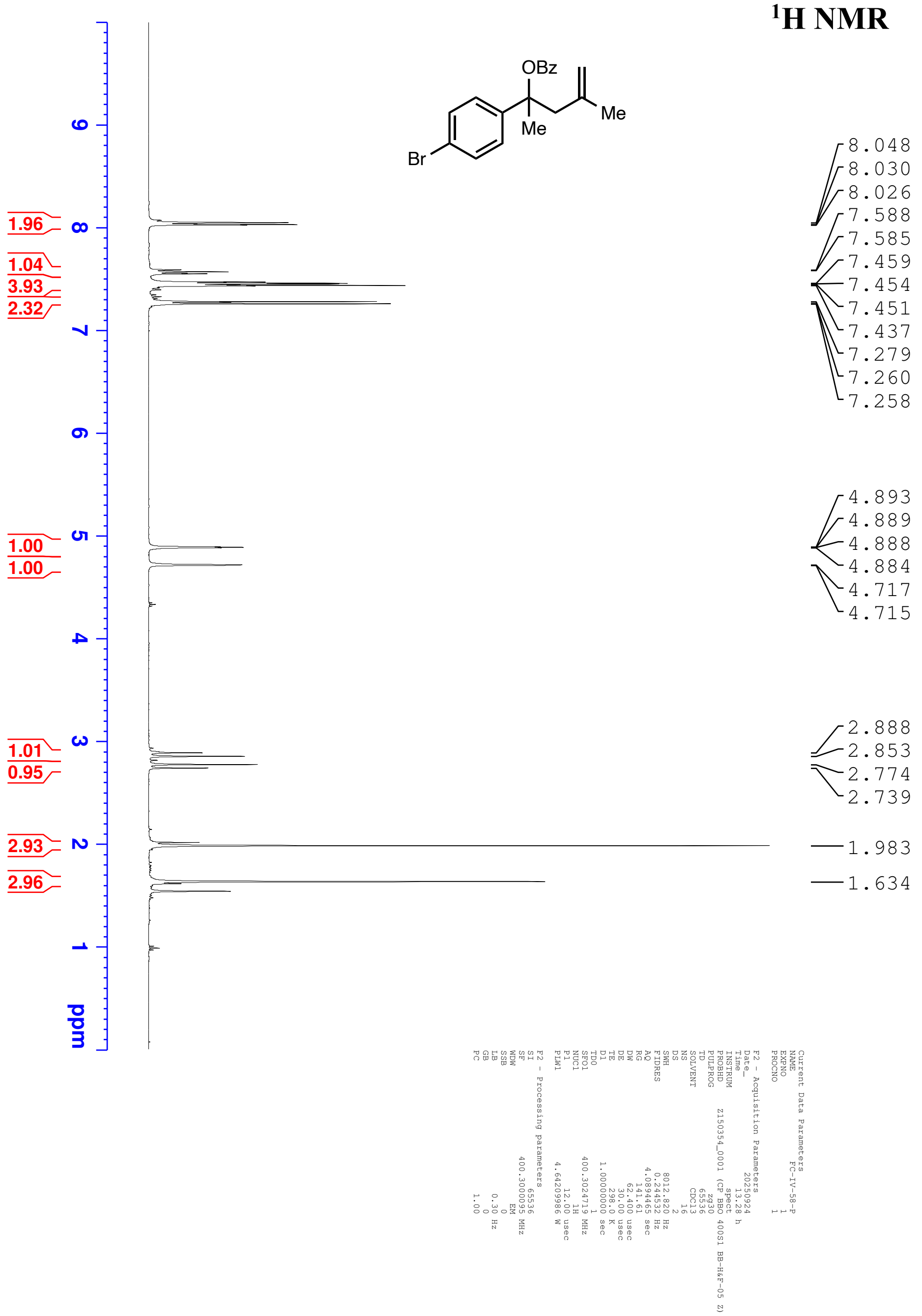

2-(4-Bromophenyl)-4-methylpent-4-en-2-yl benzoate (S30).

Solvent: $CDCl_3$
100 MHz
$^{13}C\{^1H\}$ NMR

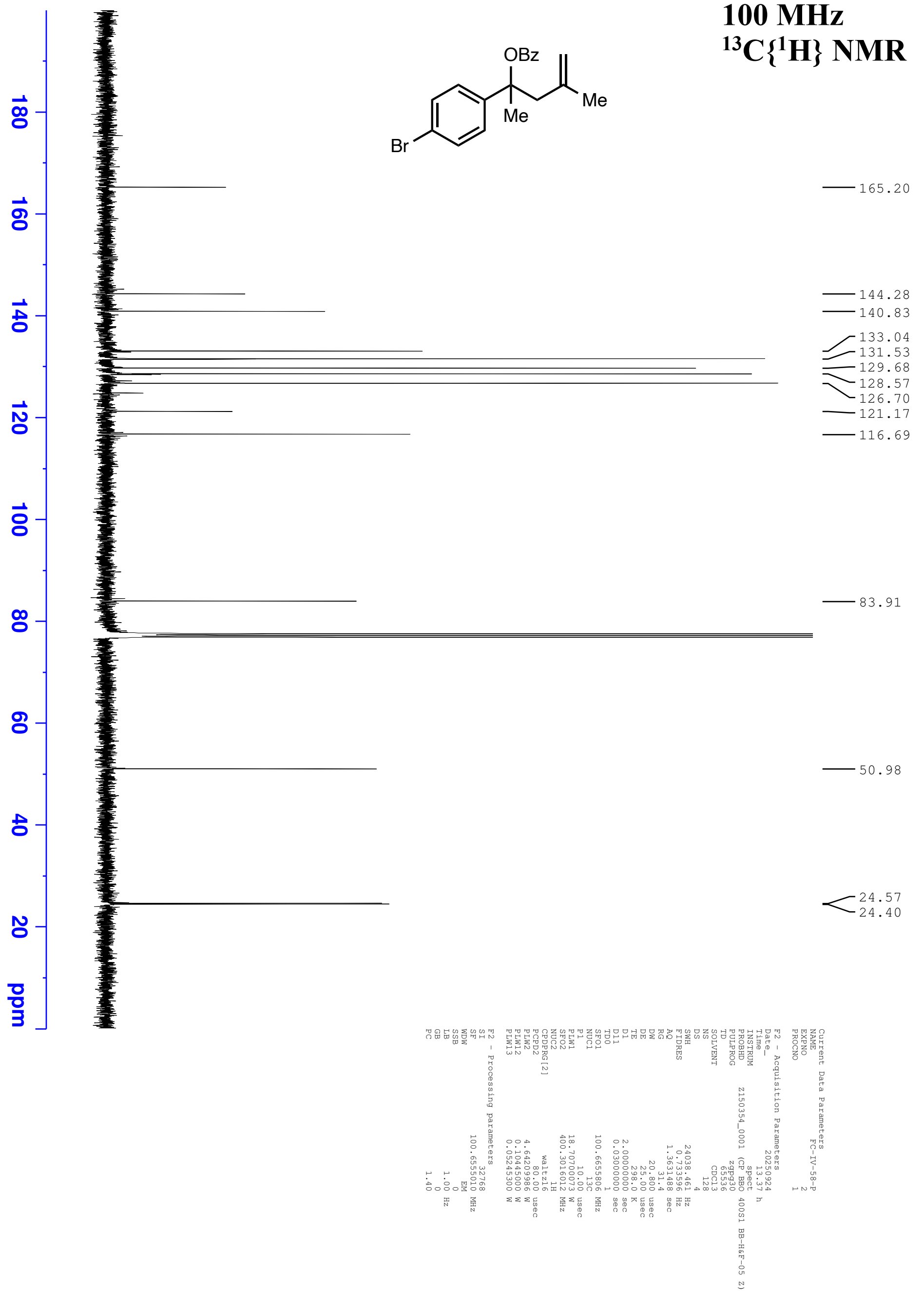

2-(4-Bromophenyl)-4-hydroperoxy-4-methylpentan-2-yl benzoate (S31).

Solvent: C_6D_6
400 MHz
^{1}H NMR

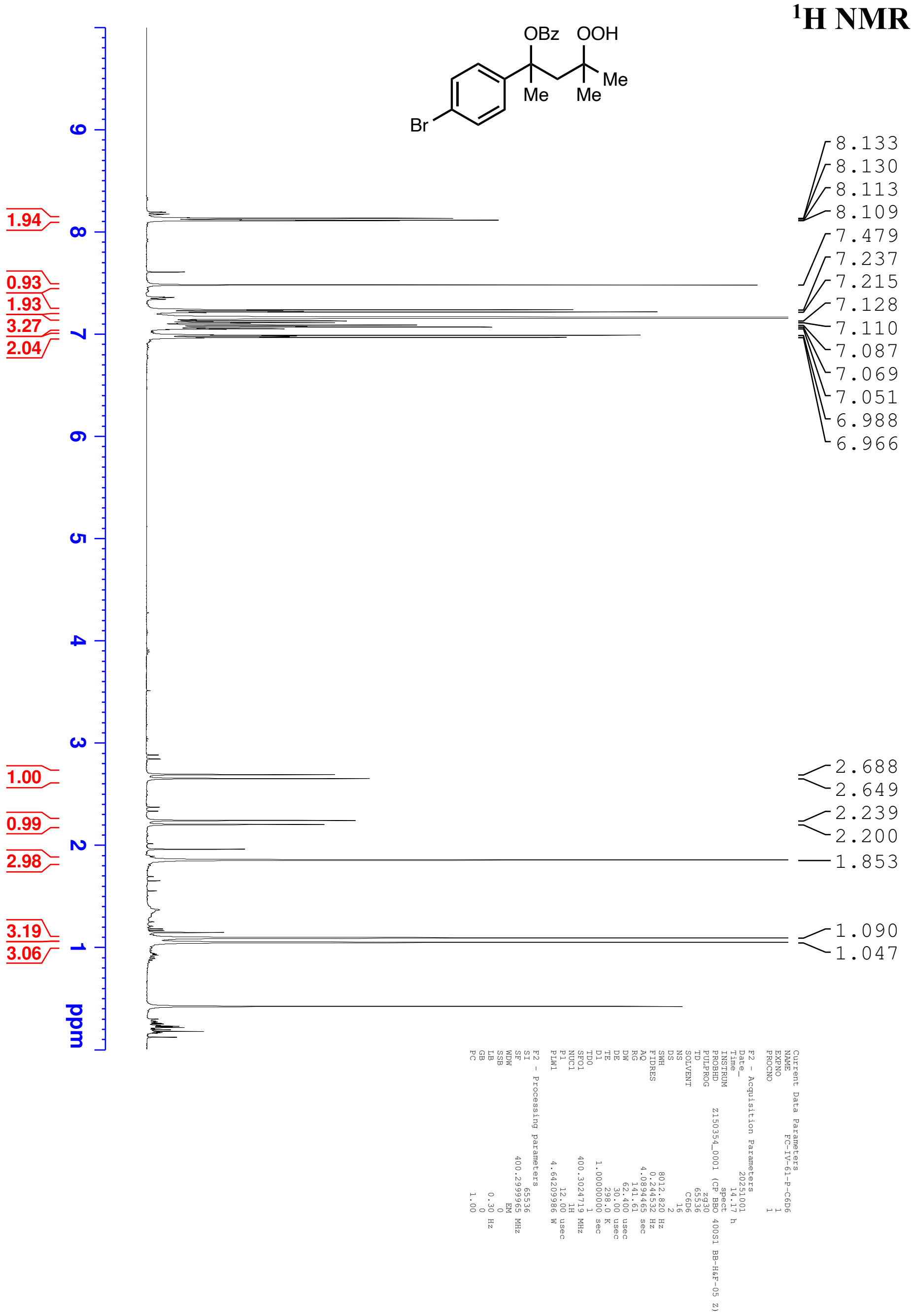

2-(4-Bromophenyl)-4-hydroperoxy-4-methylpentan-2-yl benzoate (S31).

Solvent: C_6D_6
100 MHz
$^{13}C\{^1H\}$ NMR

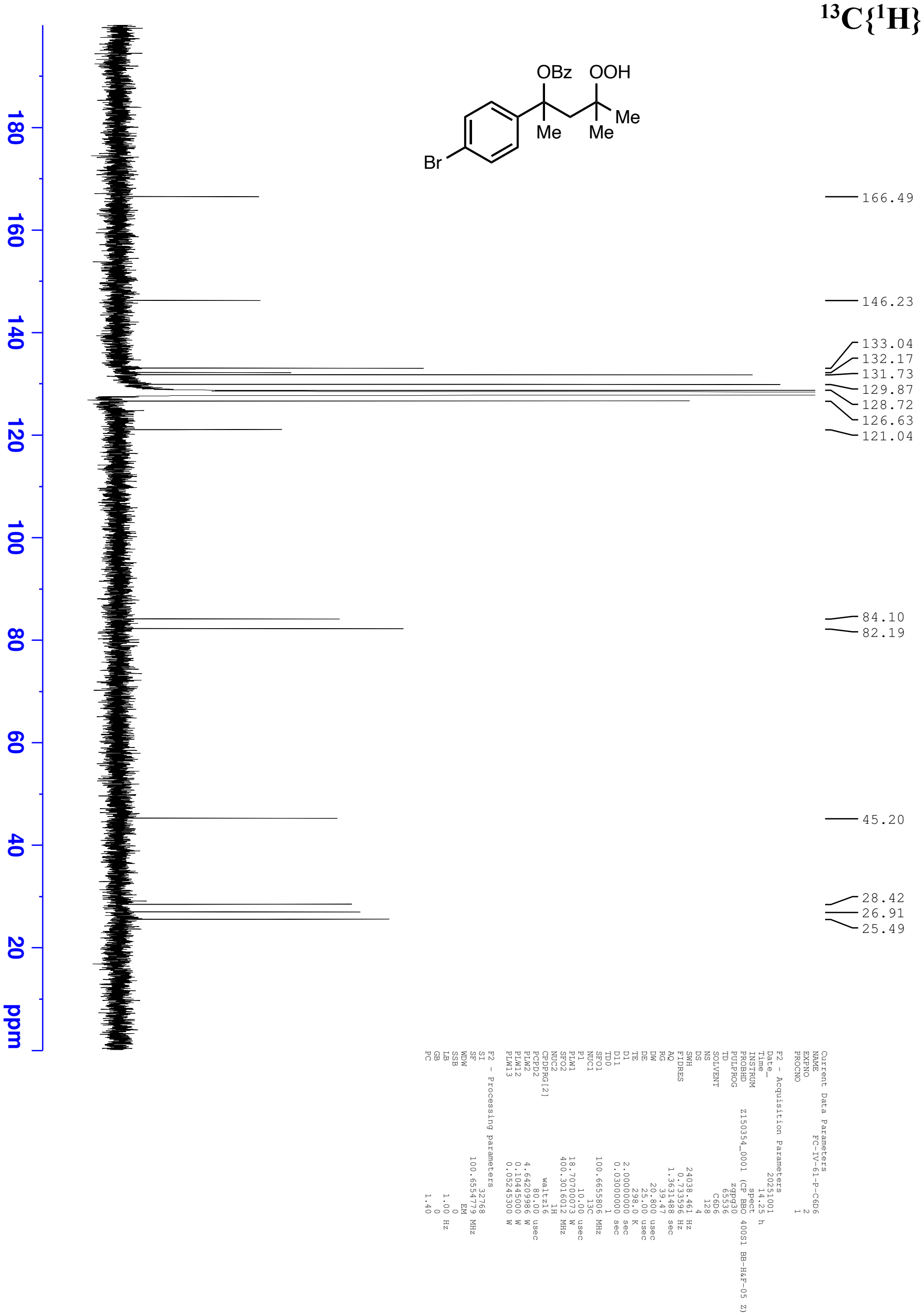

1-(4-Bromophenyl)ethan-1-one-^{17}O (S33).

Solvent: $CDCl_3$
400 MHz
^{1}H NMR

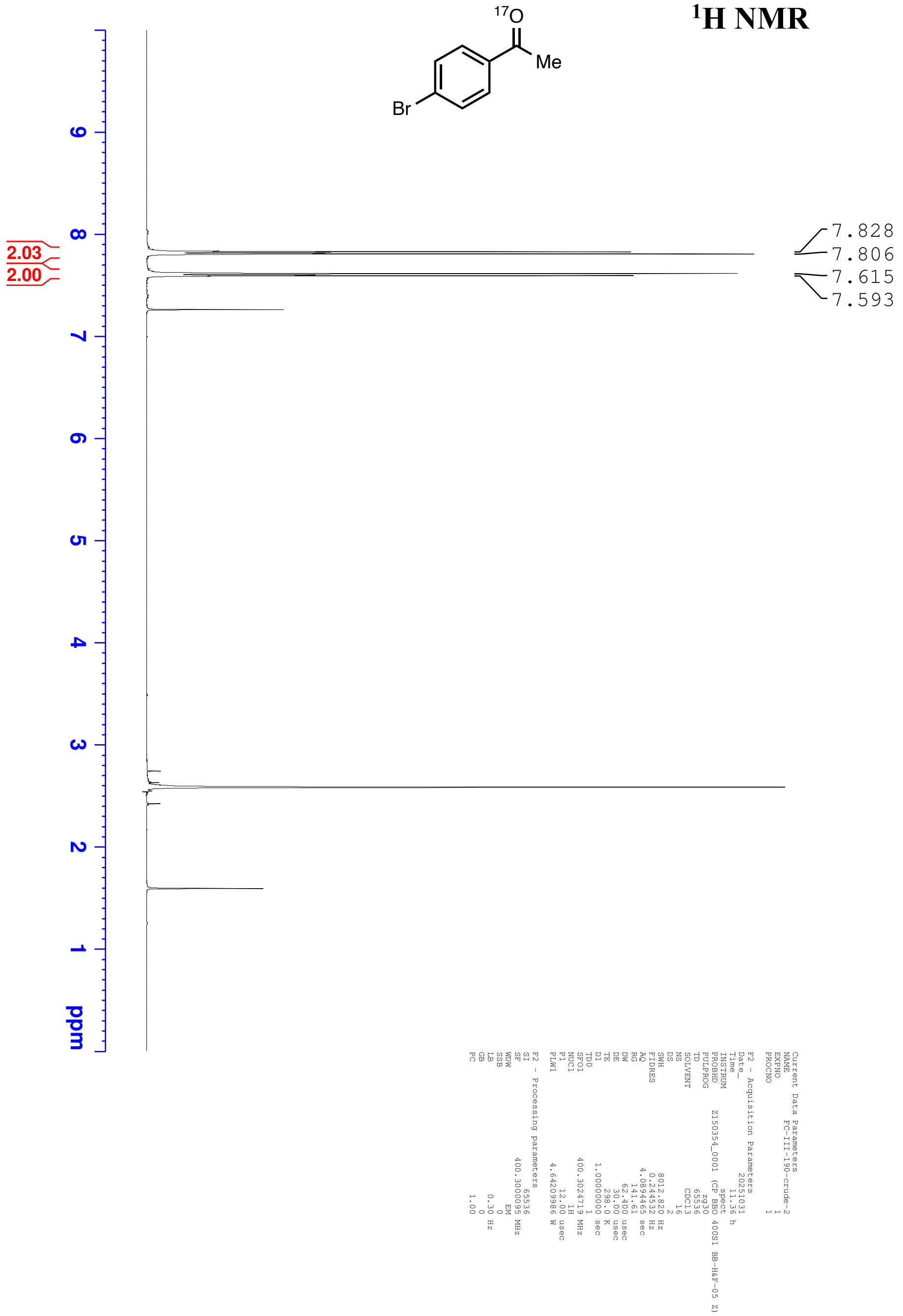

1-(4-Bromophenyl)ethan-1-one-^{17}O (S33).

Solvent: $CDCl_3$
100 MHz
$^{13}C\{^1H\}$ NMR

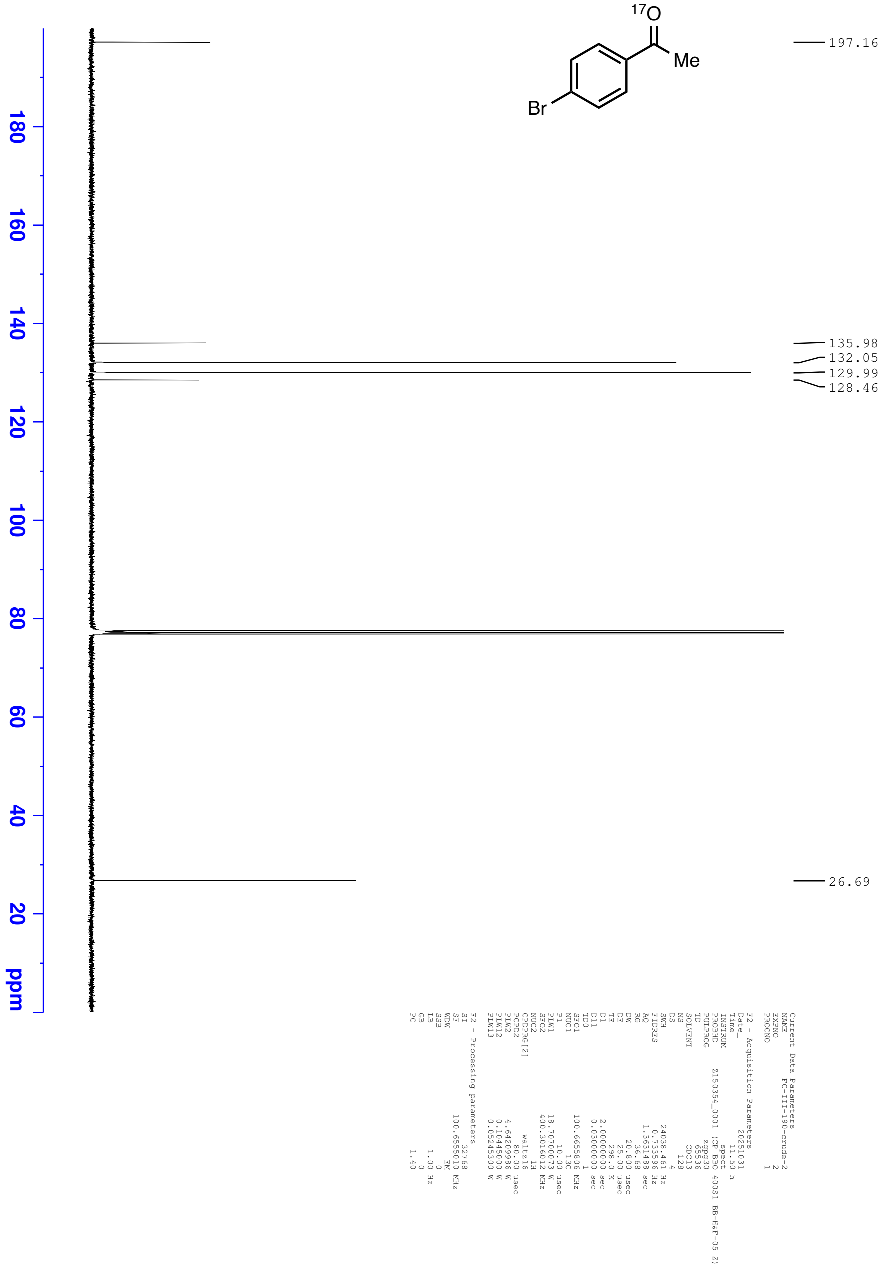

1-(4-Bromophenyl)ethan-1-one-^{17}O (S33).

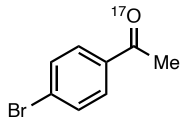

Solvent: $CDCl_3$
54 MHz
^{17}O NMR

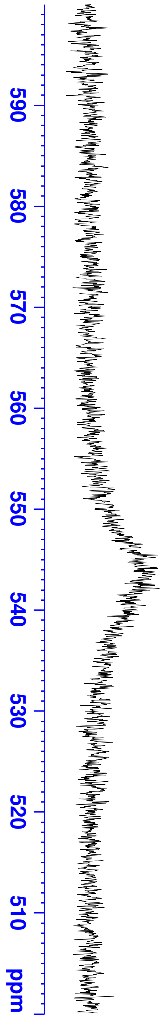

Supplement: Supplementary file 1 [file ja6c04588_si_001.pdf]
